# Stereospecific Insertion of Cyclic Amidines into Aryl-Substituted Cyclopropanones: Access to Complex Spirocyclic Aminals

**DOI:** 10.1021/acs.orglett.5c02501

**Published:** 2025-07-21

**Authors:** Richard Herzog, Ishika Agrawal, Heinrich F. von Köller, Konstantin Kloiber, Daniel B. Werz

**Affiliations:** † Institute of Organic Chemistry, Albert-Ludwigs-Universität Freiburg, 79104 Freiburg im Breisgau, Germany; ‡ Institute of Inorganic and Analytical Chemistry, Albert-Ludwigs-Universität Freiburg, 79104 Freiburg im Breisgau, Germany

## Abstract

A facile, catalyst-free protocol to access structurally complex spirocyclic aminals from cyclopropanone surrogates and amidines is reported. The two-component reaction is described as a stereospecific insertion of the amidine C=N bond into the C1–C2 bond of the cyclopropanone via the addition of N to the carbonyl group, followed by ring enlargement. The products display similarities to numerous biologically active oligocyclic systems. Further structural diversification is demonstrated, and a proposed mechanism based on experimental and computational evidence is provided.

## Graphical abstract

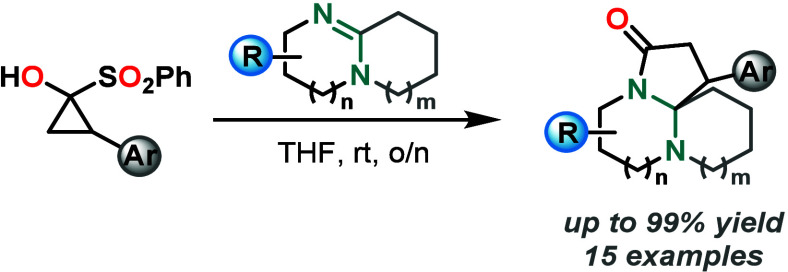

Structurally complex fused nitrogen heterocycles continue to be of high interest to the medicinal and pharmaceutical chemistry community.^1,2^ These compounds show strong tendencies to exhibit antimicrobial,^3^ antitumor,^4^ or antifungal activity^5^ and are therefore commonly found in a plethora of agrochemicals and drugs.^1,6^ For instance, natural products bearing the pyrrolizidine skeleton, such as pyrrolams A–D or spiro­[pyrrolidine-azlactones], exhibit considerable biological activity, rendering them highly desirable synthetic targets (Scheme 1A).^7–9^

**Scheme 1:**
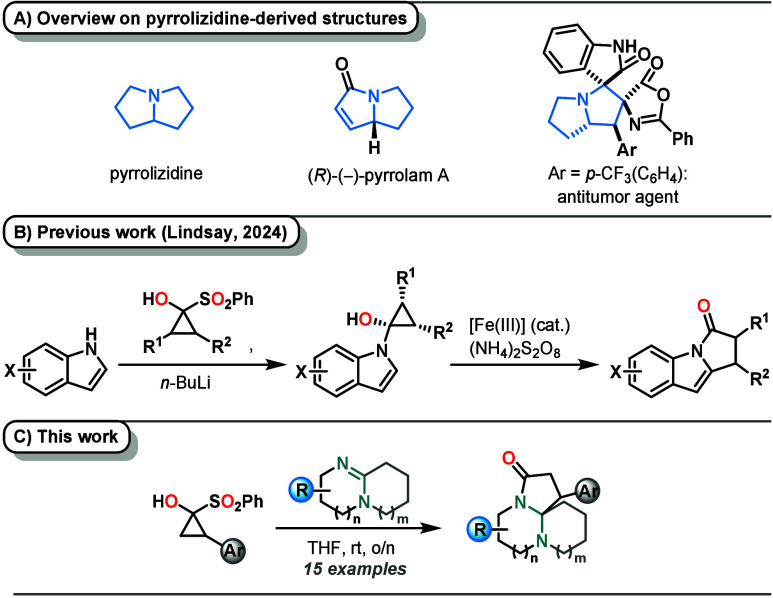
(A) Pyrrolizidine Derivatives, (B) Recent Work on the Synthesis of Pyrroloindoles from Cyclopropanones, and (C) Synthesis of Spirocyclic Aminals (this work)

A myriad of C–C bond-forming reactions such as pericyclic,^10,11^ photochemical,^12^ and transition-metal-catalyzed reactions^13,14^ have been employed to obtain these immensely valuable compounds and related structures.^15^ However, access to these relatively complex molecular scaffolds often requires elaborate multistep syntheses and the use of bespoke transition-metal catalysts, resulting in increased time and cost of synthesis.^16,17^ Therefore, more direct, easily applicable synthetic routes toward these compounds from readily available starting materials are highly desirable.

Recent works by Lindsay and co-workers have already shown how cyclopropanones, generated in situ from stable precursors, may serve as powerful synthetic three-carbon building blocks in organic synthesis^18–23^ as well as for the synthesis of complex N-containing fused heterocyclic frameworks.^24^ In particular, the authors investigated an Fe-catalyzed oxidative rearrangement reaction of indole-derived cyclopropanone hemiaminals to form medicinally relevant pyrroloindoles (Scheme 1B).^24^ Due to their immense ring strain of about 50 kcal mol^–1^, cyclopropanones are prone to undergoing a variety of nucleophilic addition reactions, followed by ring opening, ring expansion, and rearrangement reactions,^19,25^ as was also shown recently by our group.^26,27^ However, to the best of our knowledge, direct incorporation of the cyclopropanone unit to generate an aza-polyquinane-type framework has not yet been reported.

Herein, a one-step, catalyst-free, and stereospecific insertion of cyclic amidines into the C1–C2 bond of cyclopropanones accessed from stable α-sulfonylcyclopropanols (SCPs), furnishing congested, spirocyclic aminals, is reported (Scheme 1C). The amidine used in this two-component reaction thus serves both as the liberating agent of the cyclopropanone surrogate and as a reactant.

SCP **1a** and amidine **2a** (1,5-diazabicyclo[4.3.0]­non-5-ene (DBN)) were chosen as model substrates for the optimization of the insertion reaction. First, the influence of the solvent on the reaction outcome was examined. Beginning with a 0.05 m reaction solution of SCP **1a** (0.1 mmol) and excess **2a** in toluene at −78 °C, a 16% yield of **3aa** was observed (Table 1, entry 1). CH_2_Cl_2_ and MeCN both delivered **3aa** in reduced yields (entries 2 and 3, respectively), while DMF afforded the product in 25% yield (entry 4). At −78 °C, THF furnished **3aa** in 31% yield, which was chosen as the suitable reaction solvent for further optimization.

**Table 1:**
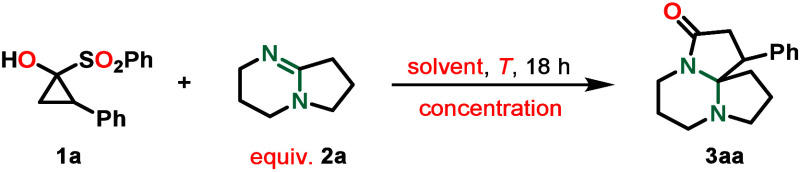
Optimization of the Reaction Conditions^a^

entry	solvent	equiv of **2a**	m (mol L^–1^)	*T* (°C)	yield (%)^b^
1	PhMe	8.0	0.050	–78	16
2	CH_2_Cl_2_	8.0	0.050	–78	11
3	MeCN	8.0	0.050	–78	13
4	DMF	8.0	0.050	–78	25
5	THF	8.0	0.050	–78	31
6^c^	THF	3.0	0.025	0	74
7	THF	6.0	0.025	0	78
8	THF	6.0	0.010	0	84
9	THF	3.0	0.010	0	89
10	THF	6.0	0.010	rt	92
11	THF	2.0	0.010	rt	89

a Reaction conditions: **1a** (0.1 mmol), 18 h.

b Yields refer to ^1^H NMR yields of the major diastereomer, determined from integration with respect to 1,3,5-trimethoxybenzene as the internal standard.

c Highest-yielding condition from optimization using the Design of Experiments method, further details of which can be found in the Supporting Information.

With this in mind, further optimization was carried out using the Design of Experiments (DoE) method (details in the Supporting Information). A clear trend could be identified, in which a large number of equivalents of DBN **2a** and a low initial concentration of SCP **1a** both were deemed beneficial, while the choice of temperature did not seem to impact the yield to a large extent. The highest-yielding condition from the experimental design (74% (Table 1, entry 6)) thus served as the starting point for further subsequent screening. The temperature was mostly kept constant, first at 0 °C and then at room temperature. The number of equivalents of **2a** was varied between 3.0 (entries 6 and 9) and 6.0 (entries 7, 8, and 10), not resulting in noticeably increased yields. Decreasing the initial concentration of **1a**, from 0.025 m (entries 6 and 7) to 0.010 m (entries 8–11), resulted in ideal reaction conditions (entry 11, 89%). It was reasoned that a low initial concentration of the cyclopropanone precursor may prohibit the formation of cyclopropanone/ol oligomers, side products of which are well-known to occur in reactions involving those species.^25,28^ Moreover, an excess of amidine seemed to be crucial for the reaction to work, as the first equivalent is likely to be consumed during the deprotection of the SCP. The amidinium ion formed this way then serves as a counterion for the sulfinate leaving group and is therefore not available for the actual insertion reaction. In fact, the conditions involving only 1.0 equiv of **2a** from the experimental design showed no conversion of the starting materials to the desired product at all.

Having found suitable reaction conditions, a range of aryl-substituted cyclopropanone precursors **1** were subjected to them, in order to investigate the substrate scope (Scheme 2). A suite of *para*-substituted SCPs were examined, with arenes displaying positive mesomeric effects, delivering nearly quantitative yields of the desired products (**3ca** and **3fa**). Accordingly, methyl substitution at the *ortho* position gave a 99% yield, despite the additional steric hindrance. In contrast to this observation, cyclopropanones bearing strongly electron-withdrawing substituents such as a *p*-CF_3_ (**3da**) or a *p*-fluoro (**3ba**) moiety furnished the respective products in only 83% or 72% yield, respectively. Except for **3da**, which was obtained as a 1:1 mixture of both diastereomers, all substrates displayed a tendency to favor the diastereomer depicted in Scheme 2 as a *cis* configuration with respect to the aryl ring and five-membered ring from DBN **2a**. With regard to the diastereomeric ratios, no clear trend is identifiable. While the highest selectivity is observed for *p*-fluoro and *o*-methyl substitution (8:1 and 7:1 dr, respectively), dr values range from 1:1 (**3da**) to 4:1 (**3aa** and **3fa**) and thus do not seem to result from obvious differences in the electronic structure of the substrate or steric influences. The reaction was scaled up to a 1.0 mmol scale using model substrates **1a** and **2a**; only a minor decrease in yield from 82% to 80% was observed. X-ray crystallographic analysis of crystals obtained from **3ba** and **3ea** unequivocally confirmed their respective structures.

**Scheme 2:**
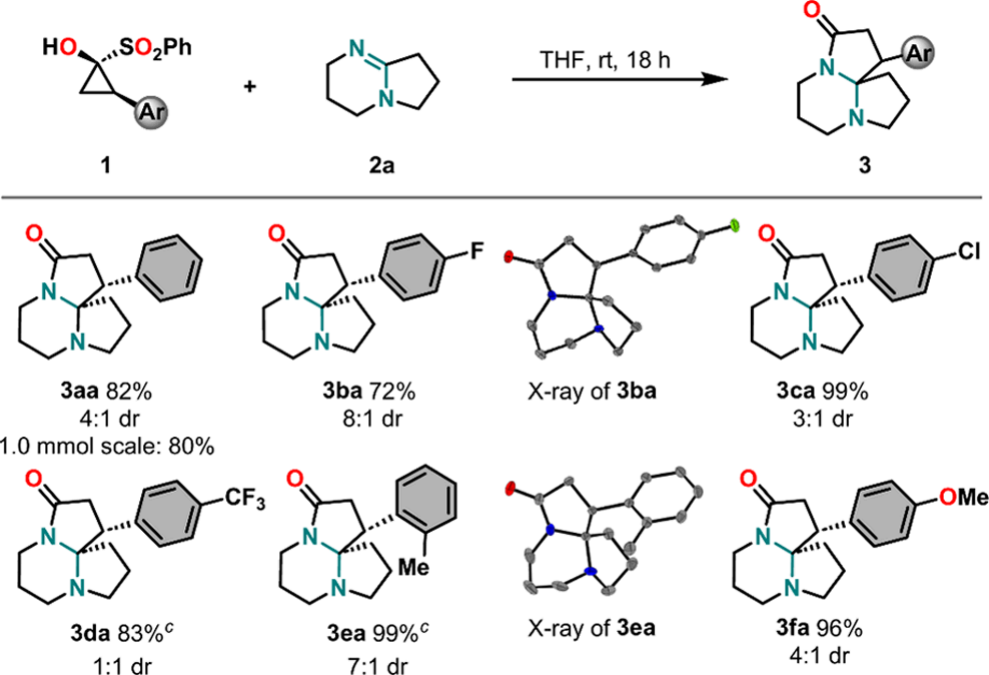
Scope of the Insertion Reaction with Respect to Cyclopropanones^a^,^b^

a Reaction conditions: **1** (0.3 mmol), **2** (0.6 mmol), THF (30 mL), rt, 18 h.
b Diastereomeric ratios were determined by ^1^H NMR spectroscopic analysis of the crude reaction mixtures.
c Obtained from 0.1 mmol scale reactions.

Having explored the variability of SCPs, the reaction was further investigated with regard to the variation of amidines **2** (Scheme 3). Yields varied more drastically here depending on the substrate’s ring-size combinations and/or additional substituents. Different variations of the model substrate DBN were employed, with yields ranging from excellent (**3ae**, six- and seven-membered rings) to good (**3ab** and **3ac**, five/five and five/six, respectively) to moderate (**3ad**, six/six). Analogous products with different substitution patterns, namely, diphenylethane diamine-derived **3af**, benzene-fused tetracycle **3ag**, and *gem*-dimethyl-substituted DBN-derived **3ah**, were obtained in 57%, 43%, and 44% yields, respectively. The decreased yields may be explained with the additional strain and steric hindrance introduced into the systems in the cases of **3af** and **3ah** and with the loss of conjugation between the amidine double bond and the benzene ring during the formation of **3ag**. A product derived from antihelmintic tetrahydropyrimidine derivative pyrantel^29^ (**3aj**) was obtained in 88% yield, with a thiophene unit connected to the amidine-bearing ring via a conjugated double bond. Structurally similar product **3ai** with only a proton instead of the (thienyl)­vinyl group was furnished in only 54% yield. It was noticed that diastereoselectivity was generally lower than that for the cyclopropanone scope, with dr values ranging only between 5:1 (**3af**) and 1:1 (**3ab**). This allows the tentative explanation to be given, that steric effects might impact diastereoselectivity to a larger extent than electronic effects. Again, no clear trend across the amidine scope was noticeable. However, it deserves mentioning that the largest dr value of 5:1 was observed for **3af**, which is derived from enantiopure amidine **2f**. Therefore, it is reasoned that diastereoselectivity might have been induced to some extent by the amidine’s substituents onto the emerging product. In most cases, the aforementioned *cis* configuration was found to be present in the major species, except for **3aj** for which the inversely configured isomer was obtained to a larger extent.

**Scheme 3:**
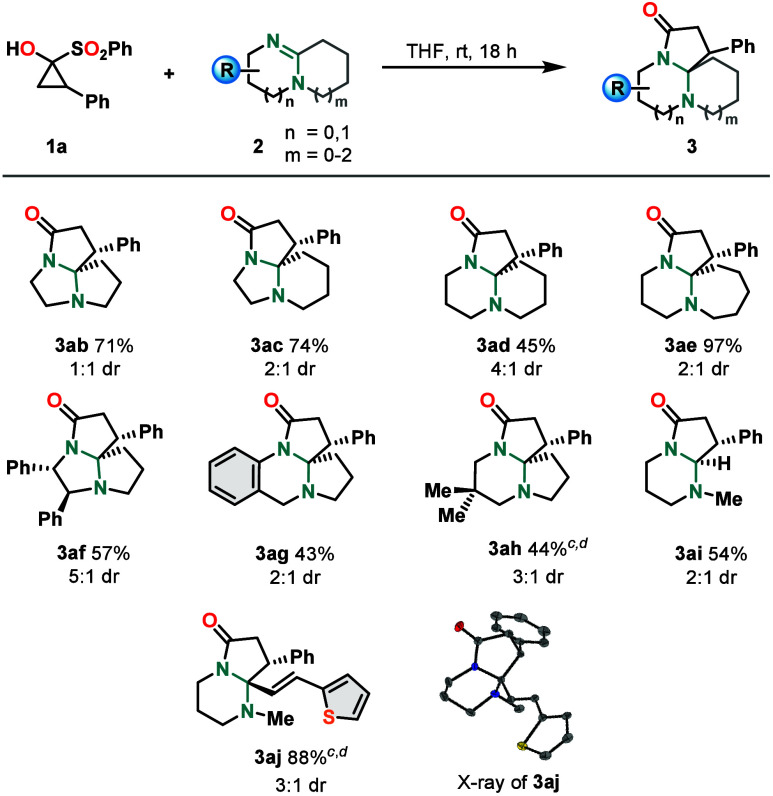
Scope of the Insertion Reaction with Respect to Amidines^a^,^b^

a Reaction conditions: **1** (0.3 mmol), **2** (0.6 mmol), THF (30 mL), rt, 18 h.
b Diastereomeric ratios were determined by ^1^H NMR spectroscopic analysis of the crude reaction mixtures.
c Obtained from 0.1 mmol scale reactions.
d CH_2_Cl_2_ was used as the solvent to account for solubility issues of the amidines.

When enantioenriched (1*S*,2*R*)-**1a** (94% ee) was subjected to standard reaction conditions with amidine **2a**, chiral HPLC analysis of **3aa** and its diastereomer **3aa′** indicated partial retention of stereoinformation at the cyclopropanone unit during the reaction (88% and 80% ee, respectively (Scheme 4A)). These findings are backed by the X-ray crystallographic analysis of crystals obtained from **3ba** and **3ea**, showing the absolute configuration of products obtained via a stereospecific pathway. When methyl-substituted or unsubstituted SCP was subjected to the standard reaction conditions, intended respective product **3ga** or **3ha** was not obtained.

**Scheme 4:**
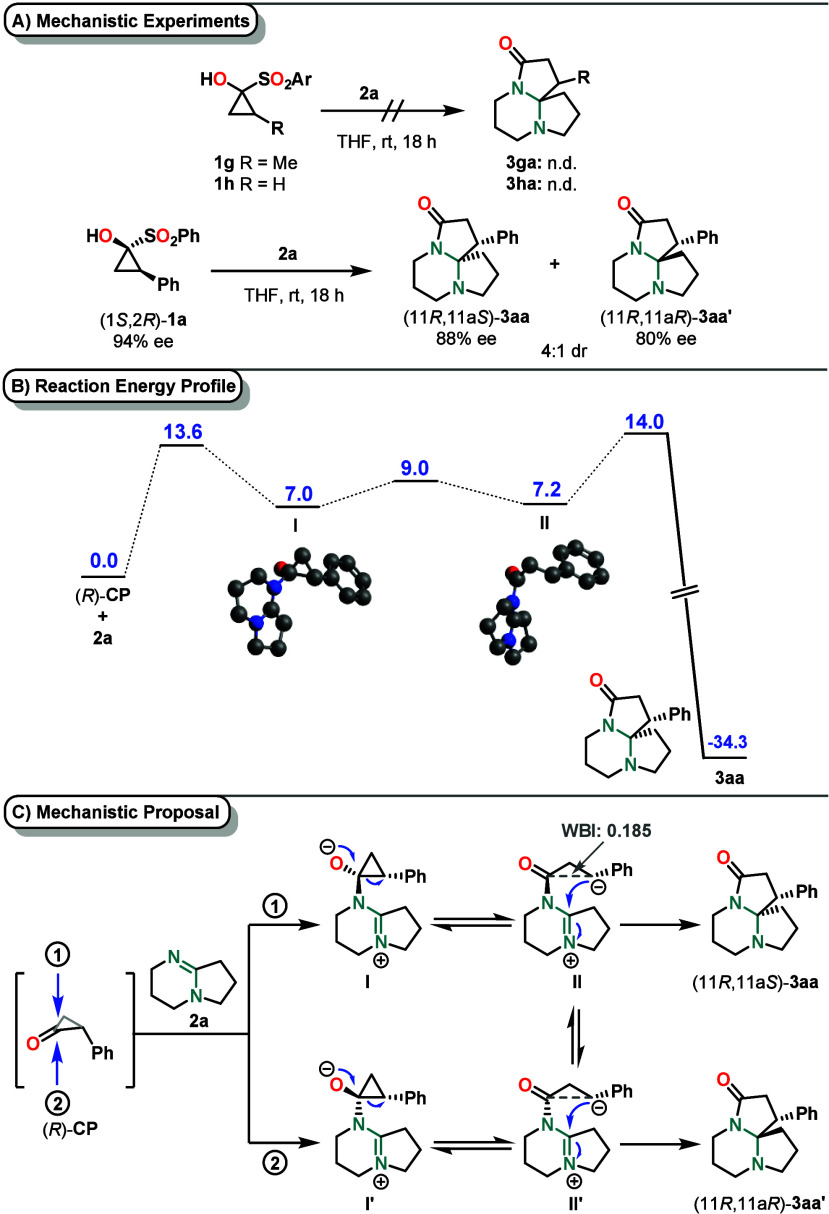
(A) Mechanistic Experiments,^a^ (B) Reaction Profile with Intermediates and Transition State Energies (PW6B95/def2-TZVP-D4//r^2^scan-3c; SMD­(THF)) in kcal/mol, and (C) Proposed Mechanism of the Insertion Reaction

a Reaction conditions: SCP (0.3 mmol), **2a** (0.6 mmol), THF (30 mL), rt, 18 h. WBI is the Wiberg bond index.

To gain deeper mechanistic insights, density functional theory (DFT) calculations were performed.^30–34^ The studies revealed that the amidine attacks cyclopropanone **CP** to form intermediate **I** with a reaction barrier of 14 kcal mol^–1^. Notably, no significant difference in the activation barrier was observed, regardless of which face of the cyclopropanone the amidine approaches. Structurally, the bond length between the two substituted carbon atoms of the cyclopropanone moiety increased by 0.12 Å. Subsequently, this bond is broken to form ring-opened zwitterionic intermediate **II** (Δ*G* ^⧧^ = 2 kcal mol^–1^). The benzylic anion then attacks the amidinium moiety to afford product **3aa**. Notably, intermediates **II** and **II′** are interconvertible via a low-energy rotational barrier of 5 kcal mol^–1^. Accordingly, the subtle difference of 0.5 kcal mol^–1^ in the activation barriers for the subsequent ring-closing steps accounts for the experimentally observed diastereomeric ratio. Identified ring-opened intermediate **II** also rationalizes why cyclopropanones bearing aliphatic instead of aromatic substituents were not tolerated in this transformation. Although the involvement of a ring-opened intermediate, in which one stereocenter is formally lost, may appear to contradict the experimentally observed stereospecificity, stereochemical information is retained in the spatial arrangement of the intermediate. Moreover, the Wiberg bond index between the carbonyl carbon and the benzylic carbon was calculated to be 0.185, indicating that the original C1–C2 bond is not yet fully cleaved (for additional computational details, see the Supporting Information; Scheme 4B,C).

Finally, aminal **3aa** was subjected to a selection of diversification reactions (Scheme 5). Reduction with lithium aluminum hydride in ether at 0 °C gave **4** in 80% yield.^35^ Another modification reaction of the lactam motif employed Lawesson’s reagent, a well-known thioketonation reagent,^36^ in THF at rt, furnishing thioamide **5** in 42% yield.^37^ Deprotonation of **3aa** adjacent to the amide moiety with lithium diisopropylamide (LDA) and subsequent methylation of the respective enolate species using methyl iodide gave a single diastereomer **6** in 31% yield (>99:1 dr).^38^

**Scheme 5:**
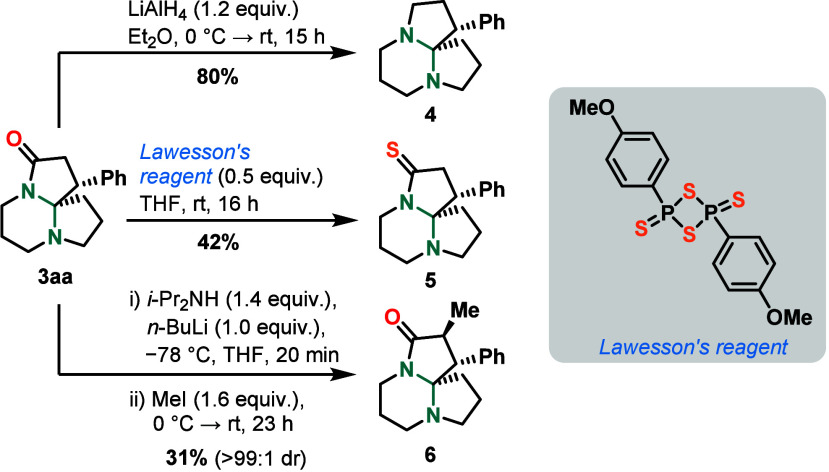
Follow-up Chemistry Reactions^a^

a All reactions were performed on a 0.1 mmol scale with a single diastereomer of **3aa**.

In summary, a facile method for the insertion of cyclic amidines into the C1–C2 bond of cyclopropanones, generated in situ from bench-stable precursors, was developed. The transformation was shown to be applicable to a broad scope of aryl-substituted cyclopropanones as well as amidines with different ring-size combinations and substituents. DFT calculations, backed by a mechanistic experiment showing partial retention of stereoinformation at the cyclopropanone, allowed for the proposal of a not fully ring-opened carbanion species during the ring enlargement step. Finally, the model compound was subjected to a variety of modifications next to or directly involving the amide motif.

## Supplementary Material



## Data Availability

The data underlying this study are available in the published article and its Supporting Information.

## Notes

Experimental procedures, details of optimization and elucidation of the mechanism, ^1^H and ^13^C NMR spectra of all new compounds, analytical data, and X-ray crystallographic data (PDF)
