# Supplementary material for: Stereospecific Insertion of Cyclic Amidines into Aryl-Substituted Cyclopropanones: Access to Complex Spirocyclic Aminals
Source: Org Lett. 2025 Jul 21;27(30):8299–303. doi: 10.1021/acs.orglett.5c02501 (PMC12322962; doi:10.1021/acs.orglett.5c02501)
Supplement: Supplementary file 1 [file ol5c02501_si_001.pdf]

SUPPORTING INFORMATION

Stereospecific Insertion of Cyclic Amidines into Aryl-Substituted Cyclopropanones: Access to Complex Spirocyclic Aminals

Richard Herzog, Ishika Agrawal, Heinrich F. von Köller†, Konstantin Kloiber† and Daniel B. Werz*

Albert-Ludwigs-Universität Freiburg,Institute of Organic Chemistry, Albertstr. 21, 79104 Freiburg im Breisgau, Germany

†*Albert-Ludwigs-Universität Freiburg, Institute of Inorganic Chemistry and Analytical Chemistry, Albertstr. 21, 79104 Freiburg im Breisgau, Germany*

(*corresponding author: daniel.werz@chemie.uni-freiburg.de)

Table of Contents

1. General Experimental ***3***

2. Synthesis of Starting Materials ***4***

α-Sulfonylcyclopropanols (SCPs) 1a-1h 4

Amidines 7

3. Synthesis of Cyclic Aminals ***9***

Specific Procedures and Characterization Data: Cyclopropanone Scope 10

Specific Procedures and Characterization Data: Amidine Scope 17

4. Follow-Up Procedures ***28***

5. Supplementary Data for Screening and Design of Experiments ***31***

6. Mechanistic Investigations ***35***

Stereospecificity Experiments 35

Supplementary Data for DFT Calculations 39

Further Mechanistic Experiments 48

7. NMR Spectra ***49***

8. X-Ray Data ***82***

9. References ***99***

1. General Experimental

Commercially available chemicals were used without further purification unless stated otherwise. Dry solvents were obtained from an M.Braun purification system (MB-SPS-800) and stored over molecular sieves. *n*-Pentane and ethyl acetate for flash chromatography were purchased in technical grade and distilled before use. All other solvents were purchased and used in analytical or HPLC grade. Air- and/or moisture-sensitive reactions were carried out in oven-dried or flame-dried glassware, septum-capped under argon atmosphere (Argon 5.0 from Sauerstoffwerke Friedrichshafen), using standard inert-atmosphere techniques. 1,2-dimethoxyethane (DME) was degassed by purging with anhydrous dinitrogen for one hour and then placing it under an argon atmosphere. Flash column chromatography (fcc) was carried out using silica gel Silica 60 (grain size 40-63 µm pore size, 230-400 mesh) from Machery-Nagel, unless stated otherwise. Thin layer chromatography analysis was carried out using silica gel-coated aluminum plates or glass plates with a fluorescence indicator (Merck 60 F254). Detection was carried out by using UV light (254 nm) and/or staining with cerium ammonium molybdate (CAM) stain. Proton (^{1}H), carbon (^{13}C) and fluorine (^{19}F) NMR spectra were recorded on a 700 MHz Bruker Advance Neo 700, 500 MHz Bruker DRX 500, 400 MHz Bruker Advance II 400, or 300 MHz Bruker AVIII 300 instrument in deuterated solvent solutions at 298 K. If samples were measured in $CDCl_3$, ^{1}H-shifts are given relative to tetramethyl silane (δ_{TMS} = 0 ppm) and ^{13}C{^{1}H}-shifts relative to the solvent peak (δ_{CDCl3} = 77.16 ppm). If measured in C_6D_6, ^{1}H- and ^{13}C{^{1}H}-shifts are given relative to the solvent peak ($\delta_{C6D6,1H}$ = 7.15 ppm and $\delta_{C6D6,13C}$ = 128.62 ppm). The chemical shift δ is given in ppm. Signals are described as following: s = singlet, d = doublet, dd = doublet of doublets, ddd = doublet of doublet of doublets, t = triplet, m = multiplet. APCI- and ESI-HRMS spectra were measured on a Thermo Fisher Scientific Exactive spectrometer with an orbitrap analyzer. IR spectra were recorded on an FT-IR Paragon 1000 spectrometer with an ATR diamond cell from Perkin Elmer over the range of 4000-650 cm^{-1} neat or as thin films. Melting points of solid products were recorded on a Schorpp MPM-HV2 Melting Point Apparatus using the open capillary method. Determination of diastereomer ratios was performed by integration of base-line separated signals in the ^{1}H NMR spectrum from mixtures of the isomers, unless stated otherwise.

Exact reaction conditions are given in the following synthetic manipulations.

2. Synthesis of Starting Materials

α-Sulfonylcyclopropanols (SCPs) 1a-1h

Synthesis of Peroxybis(triethylsilane)

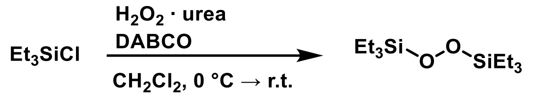

The reaction was carried out following a literature procedure. All analytical data were consistent with those reported in the literature.[1]

Synthesis of (*R*)-Ru(II)-Pheox[1] catalyst

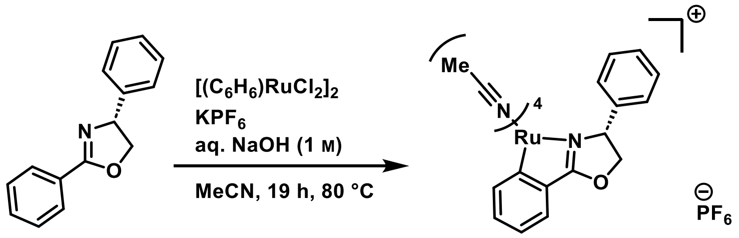

The reaction was carried out following a literature procedure. All analytical data were consistent with those reported in the literature.[2]

Synthesis of sulfonylcyclopropane precursor 1-diazo-1-(phenylsulfonyl)propan-2-one

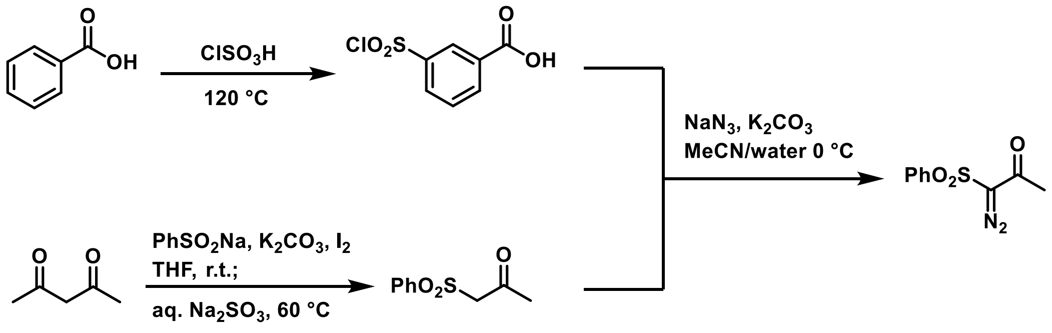

[1] Tetrakis(acetonitrile)[2-[(4*R*)-4,5-dihydro-4-phenyl-2-oxazolyl-*N*]phenyl]ruthenium(II) Hexafluorophosphate

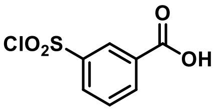

3-(Chlorosulfonyl)benzoic acid. The reaction was carried out following a literature procedure. All analytical data were consistent with those reported in the literature.[3]

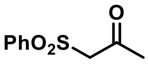

1-(Phenylsulfonyl)propan-2-one. The reaction was carried out following a literature procedure. All analytical data were consistent with those reported in the literature.[4]

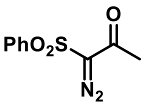

1-Diazo-1-(phenylsulfonyl)propan-2-one. The reaction was carried out following a literature procedure. All analytical data were consistent with those reported in the literature.[5]

Synthesis of enantioenriched, aryl-substituted SCPs 1a-1f

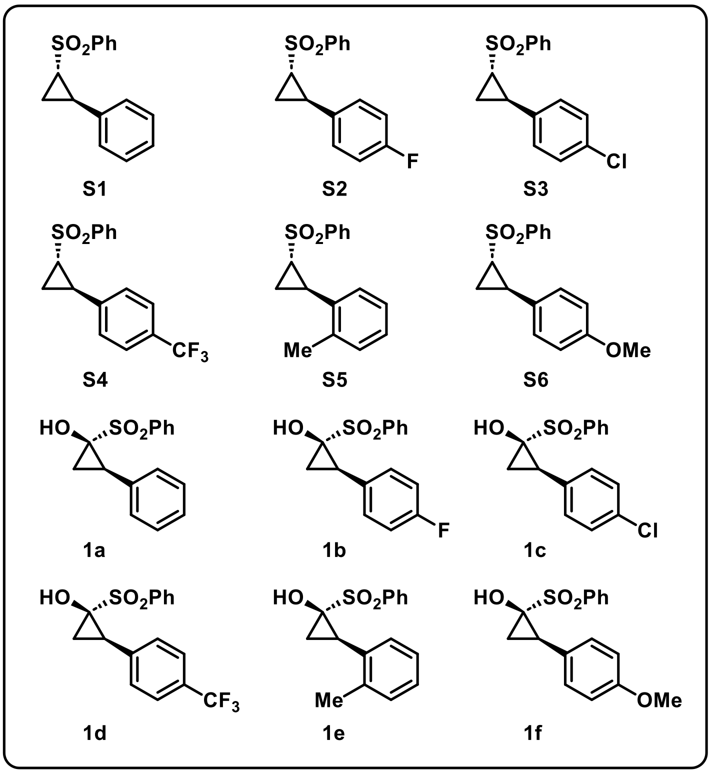

Figure 1: Overview on the synthesized sulfonylcyclopropanes S1-S6 and α-sulfonylcyclopropanols 1a-1f.

Synthesis of sulfonylcyclopropanes S1-S6 from styrene derivatives.

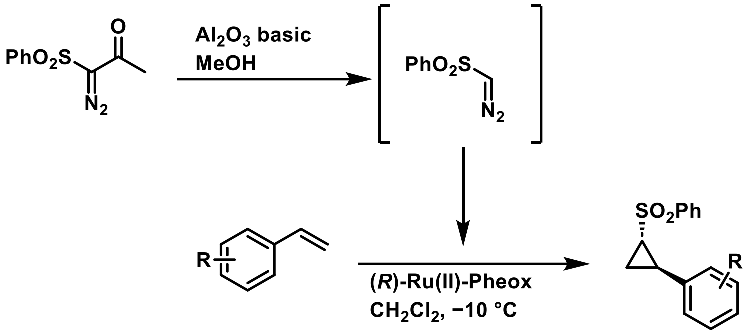

The reactions were carried out following literature procedures. All analytical data were consistent with those reported in the literature.[1,6]

Synthesis of α-sulfonylcyclopropanols 1a-1f *via* α-hydroxylation.

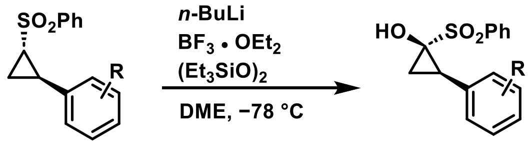

The reactions were carried out following a modified literature procedure. All analytical data were consistent with those reported in the literature.[1]

Synthesis of racemic phenyl-substituted SCP (*rac*)-1a and methyl-substituted SCP 1g

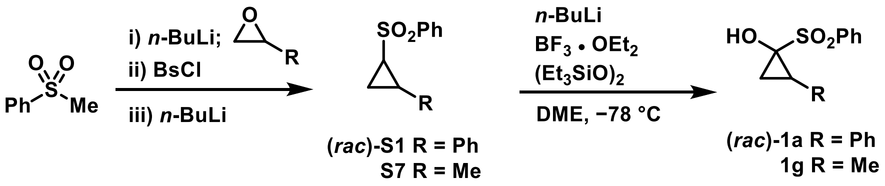

The reactions were carried out following literature procedures. All analytical data were consistent with those reported in the literature.[1]

Synthesis of 1-Tosylcyclopropan-1-ol (1h)

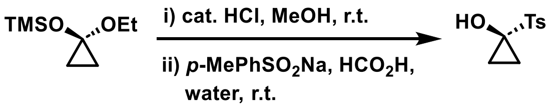

The reaction was performed following a literature procedure. All analytical data were consistent with those reported in the literature.[1]

Amidines

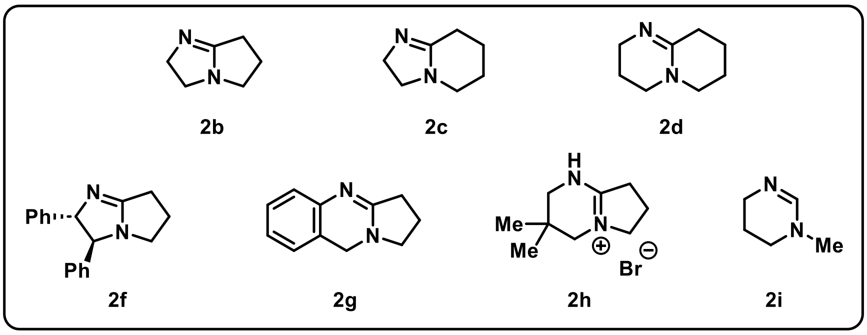

Figure 2: Overview on the synthesized cyclic amidines 2b-2d and 2f-2i.

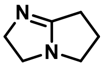

2,5,6,7-Tetrahydro-3H-pyrrolo[1,2-a]imidazole (2b). The reaction was carried out following a literature procedure. All analytical data were consistent with those reported in the literature.[7]

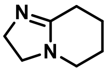

2,3,5,6,7,8-Hexahydroimidazo[1,2-a]pyridine (2c). The reaction was carried out following a literature procedure. All analytical data were consistent with those reported in the literature.[7]

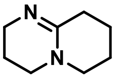

3,4,6,7,8,9-Hexahydro-2H-pyrido[1,2-a]pyrimidine (2d). The reaction was carried out following a literature procedure. All analytical data were consistent with those reported in the literature.[7]

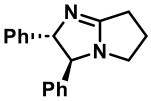

(2*S*,3*S*)-2,3-Diphenyl-2,5,6,7-tetrahydro-3H-pyrrolo[1,2-a]imidazole (2f). The reaction was carried out following a literature procedure. All analytical data were consistent with those reported in the literature.[8]

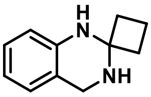

3',4'-Dihydro-1'H-spiro[cyclobutane-1,2'-quinazoline] (2g'). The reaction was carried out following a literature procedure. All analytical data were consistent with those reported in the literature.[8]

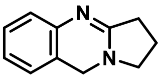

1,2,3,9-Tetrahydro-pyrrolo[2,1-b]quinazoline (2g). The reaction was carried out following a literature procedure. All analytical data were consistent with those reported in the literature.[8]

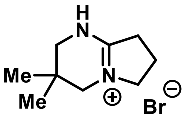

3,3-Dimethyl-2,3,4,6,7,8-hexahydropyrrolo[1,2-a]pyridinium bromide (2h). The reaction was carried out analogous to a literature procedure.[8] In an oven-dried round-bottomed flask under argon, cyclobutanone (120 µL, 1.64 mmol, 1.03 equiv.) and 2,2-dimethylpropane-1,3-diamine (162 mg, 1.59 mmol, 1.00 equiv.) were dissolved in dry CH_2Cl_2 (15 mL). The reaction mixture was stirred for 18 h at room temperature. The solvent was removed *in vacuo* and the residue was dissolved in dry CH_2Cl_2 (15 mL). The solution was cooled to 0 °C and NBS (292 mg, 1.64 mmol, 1.04 equiv.) was added. The mixture was stirred at 0 °C for 30 min and was then stirred for another 4 h, while being allowed to warm to room temperature. After the reaction went to completion (as judged by TLC analysis of the crude reaction mixture, EtOAc + 0.5 Vol% NEt_3), sat. aq. $Na_2S_2O_3$ (10 mL) and 10% aq. NaOH (10 mL) were added and the mixture was extracted with CH_2Cl_2 (2 × 10 mL). The combined organic layers were dried over Na_2SO_4 and the solvent was removed *in vacuo*. Purification of the crude product *via* fcc (alox stationary phase, EtOAc to EtOAc/MeOH 5:1) and drying *in vacuo* yielded **2h** in the form of its hydrobromide salt[2] (255 mg, 1.09 mmol, 69%) as a brown waxy solid.

^{1}H NMR (500 MHz, $CDCl_3$): δ = 11.82 (br. s, 1H), 3.69 – 3.63 (m, 2H), 3.25 – 3.14 (m, 4H), 3.09 (s, 2H), 2.28 – 2.17 (m, 2H), 1.10 – 1.05 (m, 6H).

^{13}C{^{1}H} NMR (126 MHz, $CDCl_3$): δ = 164.0, 54.4, 53.5, 49.7, 29.9, 27.3, 24.6, 19.3.

HRMS (ESI): *m/z* calcd. for $C_9H_{17}N_2^+$ 153.1386 $[M]^+$, found 153.1389 $[M]^+$.

IR: $\tilde{\nu}$ (cm^{-1}) = 3387, 3113, 2957, 2878, 2167, 1680, 1592, 1402, 1373, 1293, 1159.

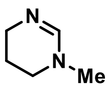

1-Methyl-1,4,5,6-tetrahydropyrimidine (2i). The reaction was carried out following a literature procedure.[9] All analytical data were consistent with those reported in the literature.[9]

The amidines **2e** (DBU) and **2j** (as its pamoate salt) were commercially available and were used without further purification.

[2] Bromide was assumed to be the counterion present in this species, as residual Br^+ from NBS must have been reduced during the quenching step.

3. Synthesis of Cyclic Aminals

GENERAL PROCEDURE 1 (GP1): Syntheses of aminals 3aa-3fa (cyclopropanone scope) and 3ab-3aj (amidine scope).[3]

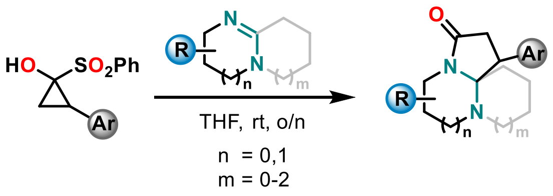

<u>0.3 mmol scale:</u>

An oven-dried round-bottomed flask was charged with SCP (0.3 mmol, 1.0 equiv.) and amidine (0.6 mmol, 2.0 equiv.), if the latter was a solid. It was evacuated and backfilled with argon and capped with a septum. Dry THF (0.01 M with respect to the SCP; 30 mL) was added *via* syringe. If the amidine was a liquid, the SCP was dissolved in dry THF (28.5 mL). In another oven-dried round-bottomed flask under argon the amidine (0.6 mmol, 2.0 equiv.) was dissolved in dry THF (0.4 M; 1.5 mL). This solution was then added to the stirred solution of SCP in dry THF under argon (0.01 M with respect to the SCP). The mixture was stirred at room temperature for the required amount of time. The flask was opened, and the mixture was transferred to a separatory funnel with CH_2Cl_2 washes (2 × 5 mL). The mixture was washed with 10% aq. NaCl (15 mL) and the aq. layers were extracted with CH_2Cl_2 (2 × 15 mL). The combined organic layers were dried over $MgSO_4$, and the solvent was removed *in vacuo*. The residue was adsorbed to celite and purified *via* fcc to yield products **3aa-3fa** and **3ab-3aj**.

<u>0.1 mmol and 1.0 mmol scale:</u>

The reactions were carried out analogous to the 0.3 mmol scale.

[3] For substrates **3ah** and **3aj** a slightly modified version of this procedure was followed, as can be found in the specific procedures for these compounds.

Specific Procedures and Characterization Data: Cyclopropanone Scope

11-Phenylhexahydro-1*H*,5*H*,9*H*-dipyrrolo[1,2-*a*:2',1'-*b*]pyrimidin-9-one (3aa/3aa')

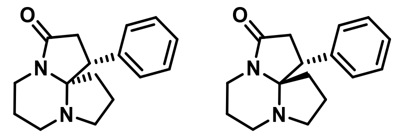

3aa **3aa'**

GP1 was followed, starting with (*rac*)-**1a** (274 mg, 1.00 mmol, 1.00 equiv.) and 1,5-diazabicyclo[4.3.0]non-5-ene (DBN) for 21.5 h. Purification by fcc (CH_2Cl_2, containing 0.5 Vol% NEt_3) afforded the title compound as a faint-brown oil and as a mixture of diastereomers **3aa** and **3aa'** (205 mg, 0.80 mmol, 80%; 4:1 dr), which were separated by another fcc (EtOAc, containing 0.5 Vol% *i*-$PrNH_2$).

0.3 mmol scale: 82% yield.

Analytical data of **3aa**:

^{1}H NMR (700 MHz, $CDCl_3$): δ = 7.37 – 7.35 (m, 2H), 7.33 – 7.31 (m, 2H), 7.29 – 7.27 (m, 1H), 4.22 – 4.17 (m, 1H), 3.50 (dd, *J* = 11.7, 8.1 Hz, 1H), 3.10 – 3.03 (m, 1H), 2.92 – 2.82 (m, 4H), 2.75 (dd, *J* = 16.2, 11.8, 1H), 2.60 (dd, *J* = 16.2, 8.1 Hz, 1H), 2.00 – 1.94 (m, 1H), 1.78 – 1.73 (m, 1H), 1.72 – 1.63 (m, 2H), 1.55 – 1.50 (m, 1H), 0.84 – 0.77 (m, 1H).

^{13}C{^{1}H} NMR (176 MHz, $CDCl_3$): δ = 171.5, 137.7, 128.7, 128.1, 127.3, 86.4, 50.4, 49.6, 41.6, 34.9, 32.3, 29.0, 22.2, 18.7.

HRMS (ESI): *m/z* calcd. for $C_{16}H_{21}ON_2^+$ 257.1648 [M+H]$^+$, found 257.1645 [M+H]$^+$.

IR: ṽ (cm^{-1}) = 2948, 2871, 1680, 1451, 1435, 1416, 1317, 1282, 1188, 1178, 1160, 1062.

R*f* (EtOAc, containing 0.5 Vol% *i*-$PrNH_2$) = 0.12.

Analytical data of **3aa'**:

^{1}H NMR (700 MHz, C_6D_6): δ = 7.16 – 7.14 (m, 1H), 7.12 – 7.09 (m, 3H), 7.05 – 7.02 (m, 1H), 4.22 – 4.17 (m, 1H), 2.60 (dd, *J* = 8.4, 2.0 Hz, 1H), 2.52 (dd, *J* = 16.7, 8.4 Hz, 1H), 2.41 (dd, *J* = 16.7, 1.9 Hz, 1H), 2.41 – 2.34 (m, 2H), 2.08 – 2.03 (m, 2H), 1.87 – 1.81 (m, 1H), 1.54 – 1.43 (m, 2H), 1.28 – 1.19 (m, 3H), 1.08 – 1.02 (m, 1H).

^{13}C{^{1}H} NMR (176 MHz, C_6D_6): δ = 171.6, 142.1, 128.7, 126.7, 85.8, 50.7, 50.4, 47.1, 38.2, 34.7, 34.7, 30.4, 21.5, 21.1.

HRMS (ESI): *m/z* calcd. for $C_{16}H_{21}ON_2^+$ 257.1648 [M+H]$^+$, found 257.1648 [M+H]$^+$.

IR: ṽ (cm^{-1}) = 2953, 2953, 2929, 2929, 2877, 2877, 1688, 1455, 1417, 1136, 1115, 1104, 1049.

mp: 114 – 126 °C.

R*f* (EtOAc, containing 0.5 Vol% *i*-PrNH_2) = 0.58.

11-(4-Fluorophenyl)hexahydro-1*H*,5*H*,9*H*-dipyrrolo[1,2-*a*:2',1'-*b*]pyrimidin-9-one (3ba/3ba')

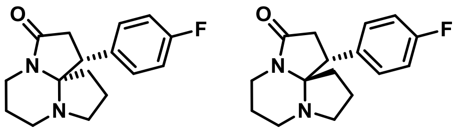

3ba 3ba'

GP1 was followed, starting with **1b** (87.7 mg, 0.30 mmol, 1.00 equiv.) and 1,5-diazabicyclo[4.3.0]non-5-ene (DBN) for 18 h. Purification by fcc (CH_2Cl_2, containing 0.5 Vol% *i*-PrNH_2) afforded the title compound as a faint-green solid and as a mixture of diastereomers **3ba** and **3ba'** (58.9 mg, 215 µmol, 72%; 8:1 dr), which were separated by another fcc (EtOAc, containing 0.5 Vol% *i*-PrNH_2).

Analytical data of **3ba**:

^{1}H NMR (700 MHz, $CDCl_3$): δ = 7.36 – 7.32 (m, 2H), 7.04 – 6.99 (m, 2H), 4.21 – 4.15 (m, 1H), 3.48 (dd, *J* = 12.0, 8.0 Hz, 1H), 3.10 – 3.03 (m, 1H), 2.94 – 2.81 (m, 4H), 2.69 (dd, *J* = 16.1, 12.0 Hz, 1H), 2.58 (dd, *J* = 16.0, 8.0 Hz, 1H), 2.03 – 1.95 (m, 1H), 1.74 – 1.61 (m, 3H), 1.57 – 1.50 (m, 1H), 0.85 – 0.77 (m, 1H).

^{13}C{^{1}H} NMR (176 MHz, $CDCl_3$): δ = 171.1, 162.2 (d, 2C), 133.2 (d, 2C), 130.0 (d, 1C), 115.0 (d, 1C), 86.2, 50.3, 48.7, 41.1, 34.8, 32.1, 29.2, 22.3, 18.3.

^{19}F NMR (659 MHz, $CDCl_3$): δ = –115.5.

HRMS (ESI): *m/z* calcd. for $C_{16}H_{20}FN_2O^+$ 275.1554 [M+H]$^+$, found 275.1553 [M+H]$^+$.

IR: ṽ (cm^{-1}) = 2934, 2868, 1673, 1511, 1418, 1274, 1217, 1181, 115, 11361102, 1058.

mp: 110 – 120 °C.

R_f (EtOAc, containing 0.5 Vol% *i*-$PrNH_2$) = 0.14.

<u>Analytical data of **3ba’**:</u>

^{1}H NMR (700 MHz, C_6D_6): δ = 6.91 – 6.87 (m, 2H), 6.76 – 6.72 (m, 2H), 4.26 – 4.19 (m, 1H), 3.08 (dd, J=11.5, 8.5, 1H), 2.62 – 2.55 (m, 1H), 2.49 – 2.44 (m, 1H), 2.41 – 2.28 (m, 5H), 1.40 – 1.34 (m, 1H), 1.34 – 1.29 (m, 1H), 1.24 – 1.17 (m, 1H), 1.17 – 1.10 (m, 1H), 1.06 – 1.00 (m, 1H), 0.60 – 0.53 (m, 1H).

^{13}C{^{1}H} NMR (176 MHz, C_6D_6): δ = 169.7, 162.4 (d, 2C), 133.9 (d, 2C), 130.3 (d, 1C), 114.8 (d, 1C), 85.4, 50.0, 48.7, 40.6, 34.7, 31.6, 29.3, 22.4, 18.1.

^{19}F NMR (659 MHz, C_6D_6): δ = –115.7.

HRMS (APCI): *m*/*z* calcd. for $C_{16}H_{20}FN_2O^+$ 275.1554 $[M+H]^+$, found 275.1554 $[M+H]^+$.

IR: ṽ (cm^{-1}) = 2947, 2873, 1690, 1511, 1455, 1421, 1282, 1222, 1159, 1064.

R_f (EtOAc, containing 0.5 Vol% *i*-$PrNH_2$) = 0.32.

11-(4-Chlorophenyl)hexahydro-1*H*,5*H*,9*H*-dipyrrolo[1,2-*a*:2',1'-*b*]pyrimidin-9-one (3ca/3ca’)

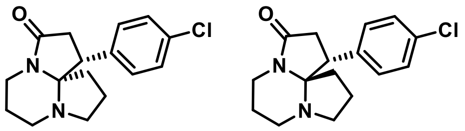

3ca 3ca'

GP1 was followed, starting with **1c** (93.0 mg, 0.30 mmol, 1.00 equiv.) and 1,5-diazabicyclo[4.3.0]non-5-ene (DBN) for 18 h. Purification by fcc (CH_2Cl_2, containing 0.5 Vol% *i*-$PrNH_2$) afforded the title compound as a light-brown, viscous oil and as a mixture of diastereomers **3ca** and **3ca’** (87.1 mg, 0.30 mmol, 99%; 3:1 dr), which were not further separated.

Analytical data of **3ca** (major diastereomer):

^{1}H NMR (500 MHz, $CDCl_3$): δ = 7.32 – 7.29 (m, 3H), 7.25 – 7.21 (m, 1H), 7.12 – 7.08 (m, 1H), 4.21 – 4.14 (m, 1H), 3.50 – 3.44 (m, 1H), 3.10 – 3.01 (m, 1H), 2.98 – 2.77 (m, 4H), 2.74 – 2.66 (m, 1H), 2.66 – 2.53 (m, 1H), 2.52 – 2.38 (m, 1H), 2.09 – 1.94 (m, 1H), 1.73 – 1.60 (m, 3H), 1.60 – 1.50 (m, 1H), 0.90 – 0.76 (m, 1H).

^{13}C{^{1}H} NMR (126 MHz, $CDCl_3$): δ = 171.0, 136.0, 133.2, 129.9, 128.3, 86.2, 50.3, 48.8, 41.0, 34.6, 32.0, 29.3, 22.4, 18.3.

HRMS (ESI): *m/z* calcd. for $C_{16}H_{20}ON_2Cl^+$ 291.1259 $[M+H]^+$, found 291.1260 $[M+H]^+$.

IR: ṽ (cm^{-1}) = 2948, 1681, 1492, 1454, 1417, 1316, 1281, 1160, 1090, 1063, 1014.

R_f (CH_2Cl_2, containing 0.5 Vol% *i*-$PrNH_2$) = 0.20[4].

11-(4-(Trifluoromethyl)phenyl)hexahydro-1H,5H,9H-dipyrrolo[1,2-a:2',1'b]pyrimidin-9-one (3da/3da')

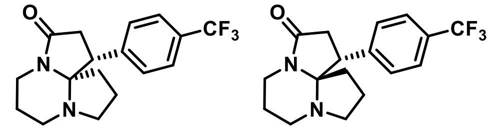

3da 3da'

GP1 was followed, starting with **1d** (28.0 mg, 0.08 mmol, 1.00 equiv.) and 1,5-diazabicyclo[4.3.0]non-5-ene (DBN) for 22 h. Purification by fcc (EtOAc, containing 0.5 Vol% *i*-$PrNH_2$) afforded **3da** (11.3 mg, 34.8 µmol, 43%) and **3da'** (10.8 mg, 33.3 µmol, 41%) as a faint-yellow and a light-green oil, respectively (total yield: 22.1 mg, 68.1 µmol, 83%; 1:1 dr).

Analytical data of **3da**:

^{1}H NMR (300 MHz, C_6D_6): δ = 7.30 (d, J=8.2, 2H), 6.96 (d, J=8.0, 2H), 4.27 – 4.15 (m, 1H), 3.12 – 3.00 (m, 1H), 2.65 – 2.49 (m, 1H), 2.49 – 2.22 (m, 6H), 1.43 – 1.03 (m, 3H), 1.02 – 0.88 (m, 1H), 0.61 – 0.34 (m, 2H).

[4] This R_f value was only obtained, if the TLC plate was "developed" twice.

^{13}C{^{1}H} NMR (176 MHz, $CDCl_3$): δ = 170.8, 141.7, 129.7 (q, 2C), 129.0, 126.7 – 121.8 (q, 2C), 125.0 (q, 1C), 86.2, 50.3, 49.2, 40.8, 34.5, 32.0, 29.5, 22.4, 18.2.

^{19}F NMR (659 MHz, $CDCl_3$): δ = –62.5.

HRMS (APCI): *m/z* calcd. for $C_{17}H_{20}F_3N_2O^+$ 325.1522 [M+H]$^+$, found 325.1527 [M+H]$^+$.

IR: ṽ (cm^{-1}) = 2951, 1692, 1619, 1421, 1327, 1283, 1164, 1120, 1069, 1018.

R$_f$ (EtOAc, containing 0.5 Vol% *i*-PrNH$_2$) = 0.12.

Analytical data of **3da'**:

^{1}H NMR (300 MHz, C_6D_6): δ = 7.30 (d, *J* = 8.1 Hz, 2H), 6.94 (d, *J* = 8.1 Hz, 2H), 4.22 – 4.09 (m, 1H), 2.50 – 2.13 (m, 5H), 2.05 – 1.89 (m, 2H), 1.83 – 1.69 (m, 1H), 1.49 – 1.38 (m, 1H), 1.24 – 0.95 (m, 4H), 0.53 – 0.41 (m, 1H).

^{13}C{^{1}H} NMR (176 MHz, C_6D_6): δ = 170.9, 146.1, 129.0 (q, 2C), 129.0, 125.8, 124.7 (q, 2C), 85.6, 50.6, 49.9, 47.0, 37.8, 34.7, 30.2, 21.4, 20.9.

^{19}F NMR (659 MHz, C_6D_6): δ = –62.1.

HRMS (APCI): *m/z* calcd. for $C_{17}H_{20}F_3N_2O^+$ 325.1522 [M+H]$^+$, found 325.1528 [M+H]$^+$.

IR (neat): ṽ (cm^{-1}) = 2945, 1691, 1619, 1418, 1326, 1290, 1163, 1116, 1069, 1050.

R$_f$ (EtOAc, containing 0.5 Vol% *i*-PrNH$_2$) = 0.41.

11-(*o*-Tolyl)hexahydro-1H,5H,9H-dipyrrolo[1,2-a:2',1'-b]pyrimidin-9-one (3ea/3ea')

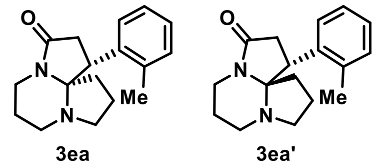

GP1 was followed, starting with **1e** (28.8 mg, 99.9 µmol, 1.00 equiv.) and 1,5-diazabicyclo[4.3.0]non-5-ene (DBN) for 17 h. Purification by fcc (EtOAc, containing 0.5 Vol% *i*-PrNH$_2$) afforded **3ea** (23.5 mg, 86.9 µmol, 87%) and **3ea'** (3.2 mg, 11.8 µmol, 12%) as a colorless solid and a colorless oil, respectively (total yield: 26.7 mg, 98.8 µmol, 99%; 7:1 dr).

Analytical data of **3ea**:

^{1}H NMR (700 MHz, $CDCl_3$): δ = 7.29 – 7.27 (m, 1H), 7.21 – 7.17 (m, 1H), 7.17 – 7.13 (m, 2H), 4.19 (dd, *J* = 13.9, 9.8 Hz, 1H), 3.89 (dd, *J* = 11.4, 8.2 Hz, 1H), 3.06 (dd, *J* = 13.9, 10.0 Hz, 1H), 2.86 (dd, *J* = 8.3, 1.5 Hz, 1H), 2.82 (dd, *J* = 9.4, 6.1 Hz, 1H), 2.80 – 2.75 (m, 3H), 2.71 (ddd, *J* = 16.3, 11.3, 1.4 Hz, 1H), 2.57 (dd, *J* = 16.4, 8.3 Hz, 1H), 2.46 (s, 3H), 2.01 – 1.96 (m, 2H), 1.96 – 1.91 (m, 1H), 1.73 – 1.65 (m, 2H), 1.56 – 1.50 (m, 1H), 0.75 – 0.66 (m, 1H)..

^{13}C{^{1}H} NMR (176 MHz, $CDCl_3$): δ = 172.1, 138.5, 136.1, 130.8, 127.4, 126.9, 125.7, 86.9, 50.1, 45.3, 41.6, 36.3, 32.5, 28.7, 22.1, 20.3, 18.7.

HRMS (ESI): *m/z* calcd. for $C_{17}H_{23}ON_2^+$ 271.1805 $[M+H]^+$, found 271.1801 $[M+H]^+$.

IR: ṽ (cm^{-1}) = 1691, 1418, 2942, 1063, 1284, 2870, 1492, 1186, 1322, 1167.

mp: 111 – 122 °C.

R*f* (EtOAc, containing 0.5 Vol% *i*-$PrNH_2$) = 0.09.

Analytical data of **3ea'**:

^{1}H NMR (700 MHz, $CDCl_3$): δ = 7.22 – 7.19 (m, 1H), 7.14 – 7.12 (m, 1H), 7.12 – 7.10 (m, 2H), 4.14 – 4.09 (m, 1H), 3.47 (dd, *J* = 8.3, 1.3 Hz, 1H), 2.91 – 2.87 (m, 1H), 2.85 (dd, *J* = 16.9, 8.3 Hz, 1H), 2.76 (dd, *J* = 10.2, 8.1 Hz, 1H), 2.68 (dd, *J* = 10.3, 7.9 Hz, 1H), 2.46 (dd, *J* = 16.7, 1.4 Hz, 1H), 2.34 (s, 3H), 2.35 – 2.31 (m, 1H), 2.23 – 2.19 (m, 1H), 2.07 – 2.01 (m, 1H), 2.00 – 1.97 (m, 1H), 1.97 – 1.93 (m, 1H), 1.85 – 1.74 (m, 2H), 1.55 – 1.48 (m, 1H).

^{13}C{^{1}H} NMR (176 MHz, $CDCl_3$): δ = 172.8, 139.5, 136.8, 130.0, 127.2, 126.6, 125.8, 86.1, 51.1, 45.8, 44.4, 37.9, 33.8, 33.6, 21.8, 20.5, 18.7.

HRMS (ESI): *m/z* calcd. for $C_{17}H_{23}ON_2^+$ 271.1805 $[M+H]^+$, found 271.1805 $[M+H]^+$.

IR: ṽ (cm^{-1}) = 3373, 2948, 2875, 1685, 1492, 1460, 1418, 1316, 1288, 1149, 1060.

R*f* (EtOAc, containing 0.5 Vol% *i*-$PrNH_2$) = 0.33.

11-(4-Methoxyphenyl)hexahydro-1*H*,5*H*,9*H*-dipyrrolo[1,2-*a*:2',1'-*b*]pyrimidin-9-one (3fa/3fa')

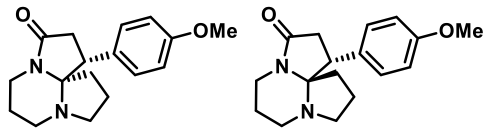

3fa 3fa'

GP1 was followed, starting with **1f** (91.5 mg, 0.30 mmol, 1.00 equiv.) and 1,5-diazabicyclo[4.3.0]non-5-ene (DBN) for 21 h. Purification by fcc (CH_2Cl_2, containing 0.5 Vol% *i*-$PrNH_2$) afforded the title compound as a light-brown, viscous oil and as a mixture of diastereomers **3fa** and **3fa'** (82.1 mg, 0.29 mmol, 96%; 4:1 dr), which were not further separated.

Analytical data of **3fa** (major diastereomer):

^{1}H NMR (500 MHz, $CDCl_3$): δ = 7.38 – 7.27 (m, 2H), 6.88 – 6.84 (m, 2H), 4.22 – 4.14 (m, 1H), 3.81 (s, 3H), 3.44 (dd, *J* = 11.9, 8.1 Hz, 1H), 3.09 – 3.02 (m, 1H), 2.91 – 2.80 (m, 4H), 2.69 (dd, *J* = 16.2, 11.8 Hz, 1H), 2.57 (dd, *J* = 16.2, 8.1 Hz, 1H), 2.05 – 1.92 (m, 1H), 1.78 – 1.59 (m, 3H), 1.58 – 1.49 (m, 1H), 0.89 – 0.77 (m, 1H).

^{13}C{^{1}H} NMR (126 MHz, $CDCl_3$): δ = 171.5, 158.9, 129.6, 128.1, 113.5, 86.4, 55.3, 50.5, 48.9, 41.7, 35.1, 32.3, 28.8, 22.2, 18.7.

HRMS (ESI): *m/z* calcd. for $C_{17}H_{23}O_2N_2^+$ 287.1754 [M+H]$^+$, found 287.1755 [M+H]$^+$.

IR: ṽ (cm^{-1}) = 2945, 2871, 1678, 1512, 1454, 1437, 1418, 1282, 1247, 1180, 1062, 1034.

R*f* (CH_2Cl_2, containing 0.5 Vol% *i*-$PrNH_2$) = 0.06.

Specific Procedures and Characterization Data: Amidine Scope

10-Phenylhexahydro-1H,8H-dipyrrolo[1,2-a:2',1'-b]imidazol-8-one (3ab/3ab')

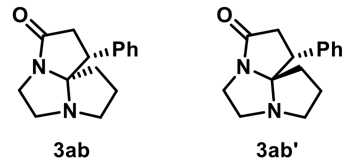

GP1 was followed, starting with (*rac*)-**1a** (82.3 mg, 0.30 mmol, 1.00 equiv.) and **2b** for 19 h. Purification by fcc (CH_2Cl_2, containing 0.5 Vol% *i*-$PrNH_2$) afforded the title compound as a colorless oil and as a mixture of diastereomers **3ab** and **3ab'** (51.7 mg, 0.21 mmol, 71%; 1:1 dr), which were separated by another fcc (EtOAc, containing 0.5 Vol% *i*-$PrNH_2$).

<u>Analytical data of **3ab**:</u>

^{1}H NMR (300 MHz, C_6D_6): δ = 7.17 (m, 2H), 7.13 – 7.04 (m, 3H), 3.81 – 3.73 (m, 1H), 3.14 (m, 1H), 2.81 – 2.68 (m, 2H), 2.60 – 2.38 (m, 2H), 2.34 – 2.24 (m, 2H), 1.99 (m, 1H), 1.69 – 1.58 (m, 1H), 1.14 – 0.95 (m, 2H), 0.92 – 0.74 (m, 1H).

A clean ^{13}C NMR spectrum of this compound could not be obtained, due to rapid decomposition upon isolation.

HRMS (ESI): *m/z* calcd. for $C_{15}H_{19}N_2O^+$ 243.1492 [M+H]$^+$, found 243.1492 [M+H]$^+$.

IR: ṽ (cm^{-1}) = 2933, 1703, 1453, 1386, 1310, 1123, 771.

R*f* (EtOAc, containing 0.5 Vol% *i*-$PrNH_2$) = 0.12.

<u>Analytical data of **3ab'**:</u>

^{1}H NMR (700 MHz, C_6D_6): δ = 7.12 – 7.09 (m, 3H), 7.09 – 7.07 (m, 1H), 7.04 – 7.01 (m, 1H), 3.66 (ddd, *J* = 11.3, 6.5, 1.0 Hz, 1H), 2.84 (dd, *J* = 7.5, 1.6 Hz, 1H), 2.74 (ddd, *J* = 16.3, 7.5, 1.2 Hz, 1H), 2.60 – 2.55 (m, 1H), 2.53 (dd, *J* = 16.3, 1.6 Hz, 1H), 2.35 (ddd, *J* = 11.3, 6.4, 1.2 Hz, 1H), 1.99 – 1.92 (m, 2H), 1.84 (dd, *J* = 6.4, 1.0 Hz, 1H), 1.68 – 1.63 (m, 1H), 1.35 – 1.26 (m, 3H).

^{13}C{^{1}H} NMR (176 MHz, C_6D_6): δ = 175.1, 140.9, 128.7, 128.3, 126.8, 95.7, 54.8, 53.3, 53.2, 40.5, 40.1, 39.2, 24.9.

HRMS (APCI): *m/z* calcd. for $C_{15}H_{19}N_2O^+$ 243.1492 [M+H]$^+$, found 243.1490 [M+H]$^+$.

IR: ṽ (cm^{-1}) = 2985, 2006, 1737, 1446, 1373, 1301, 1234, 1098, 1044, 938.

R_f (EtOAc, containing 0.5 Vol% *i*-$PrNH_2$) = 0.44.

1-Phenyloctahydro-3H-pyrrolo[2',1':2,3]imidazo[1,2-a]pyridin-3-one (3ac/3ac')

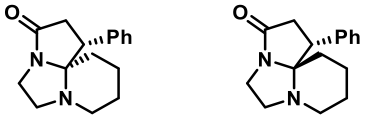

3ac **3ac'**

GP1 was followed, starting with (*rac*)-**1a** (82.6 mg, 0.30 mmol, 1.00 equiv.) and **2c** for 18 h. Purification by fcc (*n*-pentane/EtOAc 1:4 to EtOAc, containing 0.5 Vol% *i*-$PrNH_2$) afforded **3ac** (37.2 mg, 145 µmol, 48%) and **3ac'** (20.2 mg, 78.9 µmol, 26%) as a colorless oil and a colorless solid, respectively (total yield: 57.4 mg, 224 µmol, 74%; 2:1 dr[5]).

<u>Analytical data of **3ac**:</u>

^{1}H NMR (500 MHz, $CDCl_3$): δ = 7.39 – 7.32 (m, 4H), 7.31 – 7.27 (m, 1H), 3.91 – 3.82 (m, 1H), 3.59 (app. t, *J* = 9.0 Hz, 1H), 3.39 – 3.31 (m, 1H), 3.26 – 3.16 (m, 2H), 3.15 – 3.08 (m, 1H), 3.02 – 2.94 (m, 2H), 2.86 (dd, *J* = 16.9, 9.2 Hz, 1H), 1.77 – 1.69 (m, 1H), 1.62 – 1.47 (m, 2H), 1.29 – 1.21 (m, 1H), 1.03 – 0.96 (m, 1H), 0.55 – 0.42 (m, 1H).

^{13}C{^{1}H} NMR (126 MHz, $CDCl_3$): δ = 176.5, 140.0, 129.1, 128.7, 127.5, 83.9, 50.7, 48.8, 45.1, 40.9, 40.0, 24.8, 20.3, 16.8.

HRMS (APCI): *m/z* calcd. for $C_{16}H_{21}N_2O^+$ 257.1648 $[M+H]^+$, found 257.1646 $[M+H]^+$.

IR: ṽ (cm^{-1}) = 3029, 2937, 2863, 1702, 1453, 1405, 1357, 1334, 1216, 1191, 1175.

R_f (*n*-pentane/EtOAc 1:4, containing 0.5 Vol% *i*-$PrNH_2$) = 0.09.

<u>Analytical data of **3ac'**:</u>

^{1}H NMR (500 MHz, $CDCl_3$): δ = 7.29 – 7.26 (m, 1H), 7.26 – 7.25 (m, 1H), 7.25 – 7.21 (m, 1H), 7.19 – 7.16 (m, 2H), 3.69 (ddd, *J* = 10.8, 8.5, 1.1 Hz, 1H), 3.50 (dd, *J* = 8.9, 1.6 Hz, 1H), 3.41 (dd, *J* = 17.4, 9.4 Hz, 1H), 3.32 (ddd, *J* = 17.1, 8.9, 1.4 Hz, 1H), 3.24 (ddd, *J* = 10.8, 8.6, 1.3 Hz, 1H),

[5] dr value determined from the ratios of isolated products.

3.16 (dd, *J* = 8.6, 2.1 Hz, 1H), 2.51 – 2.45 (m, 2H), 2.18 – 2.10 (m, 1H), 1.90 – 1.84 (m, 1H), 1.81 – 1.69 (m, 2H), 1.68 – 1.61 (m, 1H), 1.61 – 1.48 (m, 1H), 1.11 – 1.04 (m, 1H).

$^{13}C\{^1H\}$ NMR (126 MHz, $CDCl_3$): δ = 174.5, 141.3, 128.6, 127.8, 126.8, 83.9, 50.1, 47.5, 44.6, 43.1, 39.3, 30.5, 22.2, 17.7.

HRMS (APCI): *m/z* calcd. for $C_{16}H_{21}N_2O^+$ 257.1648 $[M+H]^+$, found 257.1646 $[M+H]^+$.

IR: ṽ (cm^{-1}) = 2930, 2857, 1694, 1456, 1446, 1408, 1341, 1244, 1191, 1126.

mp: 92 – 103 °C.

R*f* (*n*-pentane/EtOAc 1:4, containing 0.5 Vol% *i*-$PrNH_2$) = 0.45.

1-Phenyloctahydro-3H,5H-pyrido[1,2-a]pyrrolo[2,1-b]pyrimidin-3-one (3ad/3ad')

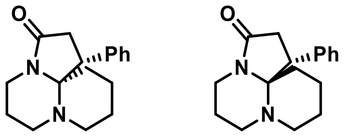

3ad **3ad'**

GP1 was followed, starting with (*rac*)-**1a** (82.3 mg, 0.30 mmol, 1.00 equiv.) and **2d** for 18 h. Purification by fcc (CH_2Cl_2, containing 0.5 Vol% *i*-$PrNH_2$) afforded the title compound as a colorless oil and as a mixture of diastereomers **3ad** and **3ad'** (36.2 mg, 0.13 mmol, 45%; 4:1 dr), which were separated by another fcc (EtOAc, containing 0.5 Vol% *i*-$PrNH_2$).

Analytical data of **3ad**:

^1H NMR (500 MHz, C_6D_6): δ = 7.12 – 7.07 (m, 2H), 7.05 – 6.97 (m, 3H), 4.30 – 4.22 (m, 1H), 3.40 (dd, *J* = 9.1, 1.9 Hz, 1H), 3.13 (app. td, *J* = 14.1, 3.3 Hz, 1H), 3.03 (dd, *J* = 16.9, 9.4 Hz, 1H), 2.76 (app. td, *J* = 12.2, 3.3 Hz, 1H), 2.48 – 2.28 (m, 3H), 2.26 – 2.19 (m, 1H), 1.83 – 1.63 (m, 2H), 1.39 – 1.26 (m, 1H), 1.21 – 1.10 (m, 1H), 1.07 – 0.98 (m, 3H), 0.71 – 0.62 (m, 1H).

$^{13}C\{^1H\}$ NMR (126 MHz, C_6D_6): δ = 176.6, 143.3, 128.5, 128.3, 127.0, 80.3, 50.6, 46.5, 44.7, 39.7, 37.3, 25.2, 21.4, 21.2, 16.9.

HRMS (APCI): *m/z* calcd. for $C_{17}H_{23}ON_2^+$ 271.1805 $[M+H]^+$, found 271.1805 $[M+H]^+$.

IR: ṽ (cm^{-1}) = 2942, 2869, 1696, 1452, 1385, 1346, 1310, 1273, 1163, 1101.

R$_f$ (EtOAc, containing 0.5 Vol% *i*-PrNH$_2$) = 0.06.

<u>Analytical data of **3ad'**:</u>

^{1}H NMR (700 MHz, C_6D_6): δ = 7.26 – 7.22 (m, 2H), 7.13 – 7.10 (m, 2H), 7.07 – 7.02 (m, 1H), 4.34 – 4.28 (m, 1H), 3.18 – 3.14 (m, 1H), 2.58 – 2.50 (m, 2H), 2.47 – 2.38 (m, 2H), 2.30 – 2.26 (m, 1H), 1.98 – 1.93 (m, 1H), 1.90 – 1.85 (m, 1H), 1.67 – 1.60 (m, 1H), 1.55 – 1.45 (m, 1H), 1.43 – 1.38 (m, 1H), 1.38 – 1.32 (m, 1H), 1.20 – 1.11 (m, 1H), 1.10 – 1.05 (m, 1H), 0.73 – 0.67 (m, 1H), 0.61 – 0.56 (m, 1H).

^{13}C{^{1}H} NMR (101 MHz, C_6D_6): δ = 170.3, 143.0, 128.9, 128.3, 126.7, 78.1, 49.1, 46.2, 45.3, 39.9, 35.7, 24.8, 23.8, 23.0, 16.9.

HRMS (APCI): *m/z* calcd. for $C_{17}H_{23}ON_2^+$ 271.1805 [M+H]$^+$, found 271.1801 [M+H]$^+$.

IR: ṽ (cm^{-1}) = 2933, 2861, 1688, 1432, 1418, 1274, 1167, 1121, 1066, 1016.

R$_f$ (EtOAc, containing 0.5 Vol% *i*-PrNH$_2$) = 0.39.

1-Phenyloctahydro-3*H*,5*H*,9*H*-pyrrolo[2',1':2,3]pyrimido[1,2-*a*]azepin-3-one (3ae/3ae')

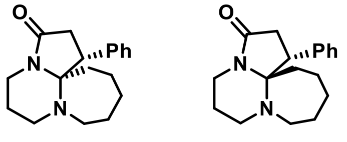

3ae **3ae'**

GP1 was followed, starting with (*rac*)-**1a** (82.5 mg, 0.30 mmol, 1.00 equiv.) and **2e** for 18 h. Purification by fcc (CH_2Cl_2, containing 0.5 Vol% *i*-PrNH$_2$) afforded the title compound as a faint-brown oil and as a mixture of diastereomers **3ae** and **3ae'** (83.0 mg, 0.29 mmol, 97%; 2:1 dr), which were separated by another fcc (EtOAc, containing 0.5 Vol% *i*-PrNH$_2$).

Analytical data of **3ae**:

^{1}H NMR (500 MHz, $CDCl_3$): δ = 7.37 – 7.32 (m, 2H), 7.32 – 7.28 (m, 2H), 7.27 – 7.15 (m, 1H), 4.21 – 4.13 (m, 1H), 3.71 – 3.64 (m, 1H), 3.37 – 3.24 (m, 1H), 3.12 – 3.03 (m, 1H), 3.01 – 2.87 (m, 3H), 2.87 – 2.79 (m, 1H), 2.59 – 2.47 (m, 1H), 2.23 – 2.13 (m, 1H), 1.96 – 1.83 (m, 1H), 1.82 – 1.70 (m, 2H), 1.70 – 1.52 (m, 2H), 1.44 – 1.32 (m, 2H), 1.21 – 1.10 (m, 1H), 0.85 – 0.74 (m, 1H).

^{13}C{^{1}H} NMR (126 MHz, $CDCl_3$): δ = 173.9, 141.4, 129.0, 128.5, 127.2, 83.5, 48.5, 47.8, 45.6, 39.0, 36.1, 31.4, 26.9, 25.5, 24.6, 21.3.

HRMS (APCI): *m/z* calcd. for $C_{18}H_{25}ON_2^+$ 285.1961 $[M+H]^+$, found 285.1964 $[M+H]^+$.

IR: ṽ (cm^{-1}) = 2924, 2859, 1679, 1454, 1438, 1419, 1386, 1352, 1273, 1146, 1085.

R$_f$ (EtOAc, containing 0.5 Vol% *i*-$PrNH_2$) = 0.14.

Analytical data of **3ae'**:

^{1}H NMR (700 MHz, C_6D_6): δ = 7.26 – 7.23 (m, 2H), 7.14 – 7.10 (m, 2H), 7.07 – 7.04 (m, 1H), 4.36 – 4.32 (m, 1H), 3.08 – 3.04 (m, 1H), 2.58 – 2.49 (m, 2H), 2.46 – 2.38 (m, 2H), 2.31 – 2.26 (m, 1H), 2.12 – 2.07 (m, 1H), 1.92 – 1.86 (m, 1H), 1.83 – 1.77 (m, 1H), 1.57 – 1.41 (m, 3H), 1.28 – 1.19 (m, 3H), 1.15 – 1.07 (m, 2H), 0.96 – 0.89 (m, 1H).

^{13}C{^{1}H} NMR (176 MHz, C_6D_6): δ = 170.0, 143.6, 128.9, 128.5, 126.5, 81.3, 46.5, 46.3, 44.8, 40.3, 35.7, 34.5, 26.4, 24.7, 24.1, 21.8.

HRMS (APCI): *m/z* calcd. for $C_{18}H_{25}ON_2^+$ 285.1961 $[M+H]^+$, found 285.1960 $[M+H]^+$.

IR: ṽ (cm^{-1}) = 2925, 2858, 1688, 1494, 1456, 1420, 1274, 1150, 1102, 1059.

R$_f$ (EtOAc, containing 0.5 Vol% *i*-$PrNH_2$) = 0.52.

5,6,10-Triphenylhexahydro-1H,8H-dipyrrolo[1,2-a:2',1'-b]imidazol-8-one (3af/3af')

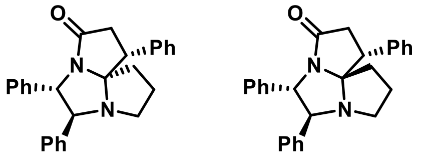

3af 3af'

GP1 was followed, starting with (*rac*)-**1a** (82.3 mg, 0.30 mmol, 1.00 equiv.) and **2f** for 19 h. Purification by fcc (*n*-pentane/EtOAc 5:1, containing 0.5 Vol% *i*-$PrNH_2$) afforded the title compound as a colorless oil and as a mixture of diastereomers **3af** and **3af'** (67.9 mg, 0.17 mmol, 57%; 5:1 dr), which were not further separated.

Analytical data of **3af** (major diastereomer):

^{1}H NMR (700 MHz, C_6D_6): δ = 7.45 – 7.42 (m, 2H), 7.31 – 7.27 (m, 2H), 7.21 – 7.18 (m, 2H), 7.18 – 7.16 (m, 1H), 7.14 – 7.13 (m, 1H), 7.13 – 7.09 (m, 3H), 7.09 – 7.06 (m, 2H), 7.06 – 7.02 (m, 2H), 5.46 (d, *J* = 6.2 Hz, 1H), 4.11 (d, *J* = 6.2 Hz, 1H), 3.66 (dd, *J* = 13.5, 7.4 Hz, 1H), 2.89 (dd, *J* = 15.5, 13.6 Hz, 1H), 2.69 – 2.65 (m, 1H), 2.38 (dd, *J* = 15.4, 7.4 Hz, 1H), 2.31 – 2.26 (m, 1H), 1.85 (m, 1H), 1.66 (m, 1H), 1.24 – 1.18 (m, 1H), 0.64 – 0.56 (m, 1H) ppm.

^{13}C{^{1}H} NMR (176 MHz, C_6D_6): δ = 174.6, 141.9, 141.3, 138.3, 128.8, 128.7, 128.6, 128.5, 128.3, 128.0 (overlaid with solvent signal), 127.4, 127.4, 126.5, 96.6, 79.6, 67.1, 55.7, 52.0, 38.5, 31.5, 24.0 ppm.

HRMS (APCI): *m/z* calcd. for $C_{27}H_{27}N_2O^+$ 395.2118 [M+H]$^+$, found 395.2122 [M+H]$^+$.

IR (neat): ṽ (cm^{-1}) = 3030, 2935, 1708, 1602, 1495, 1452, 1372, 1304, 1148, 1077, 1030.

R$_f$ (*n*-pentane/EtOAc 5:1, containing 0.5 Vol% *i*-$PrNH_2$) = 0.50.

3-Phenyl-2,3,5,6-tetrahydro-1H,4H,8H-dipyrrolo[1,2-a:2',1'-b]quinazolin-1-one (3ag/3ag')

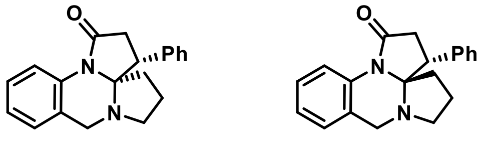

GP1 was followed, starting with (*rac*)-**1a** (82.4 mg, 0.30 mmol, 1.00 equiv.) and **2g** for 20 h. Purification by fcc (*n*-pentane/EtOAc 1:4, containing 0.5 Vol% *i*-PrNH_2) afforded the title compound as a colorless oil and as a mixture of diastereomers **3ag** and **3ag'** (39.0 mg, 0.13 mmol, 43%; 2:1 dr), which were separated by another fcc (*n*-pentane/EtOAc 6:1 to 2:1, containing 0.5 Vol% *i*-PrNH_2).[6]

Analytical data of **3ag**:

^{1}H NMR (500 MHz, C_6D_6): δ = 8.12 – 8.06 (m, 1H), 7.24 – 7.20 (m, 2H), 7.20 – 7.15 (m, 1H), 7.13 – 7.06 (m, 3H), 6.95 – 6.91 (m, 1H), 6.90 – 6.86 (m, 1H), 3.54 (d, *J* = 15.1 Hz, 1H), 3.35 (dd, *J* = 13.1, 7.3 Hz, 1H), 3.20 (d, *J* = 15.1 Hz, 1H), 2.65 (dd, *J* = 15.9, 13.1 Hz, 1H), 2.54 – 2.47 (m, 1H), 2.35 (dd, *J* = 15.9, 7.3 Hz, 1H), 2.25 – 2.16 (m, 1H), 1.73 – 1.65 (m, 1H), 1.08 – 0.98 (m, 1H), 0.78 – 0.68 (m, 1H), 0.48 – 0.37 (m, 1H).

^{13}C{^{1}H} NMR (126 MHz, C_6D_6): δ = 169.1, 136.6, 135.8, 131.7, 129.3, 128.5, 127.5, 127.3, 127.0, 124.6, 124.3, 88.0, 52.0, 50.0, 47.1, 34.0, 33.9, 22.7.

HRMS (APCI): *m/z* calcd. for $C_{20}H_{21}ON_2^+$ 305.1648 $[M+H]^+$, found 305.1648 $[M+H]^+$.

IR: ṽ (cm^{-1}) = 3033, 2938, 2853, 1701, 1602, 1489, 1458, 1387, 1355, 1188, 1134, 1042.

R_f (*n*-pentane/EtOAc 2:1, containing 0.5 Vol% *i*-PrNH_2) = 0.08.

Analytical data of **3ag'**:

^{1}H NMR (500 MHz, C_6D_6): δ = 8.23 (dd, *J* = 8.0, 1.2 Hz, 1H), 7.21 – 7.16 (m, 2H), 7.14 – 7.10 (m, 1H), 7.04 – 7.00 (m, 3H), 6.80 (app. td, *J* = 7.6, 1.4 Hz, 1H), 6.60 (app. d, *J* = 8.0 Hz, 1H), 2.72 (dd, *J* = 5.4, 2.4 Hz, 1H), 2.67 – 2.56 (m, 6H), 2.32 – 2.24 (m, 1H), 1.69 – 1.62 (m, 1H), 1.42 – 1.24 (m, 2H), 1.19 – 1.11 (m, 1H).

[6] **3ag** was further purified by an additional preparative TLC (CH_2Cl_2, containing 0.5 Vol% *i*-PrNH_2).

$^{13}C\{^1H\}$ NMR (126 MHz, C_6D_6): δ = 170.6, 140.5, 135.3, 130.9, 129.4, 128.5, 126.9, 126.8, 124.5, 123.5, 87.8, 53.4, 52.4, 48.3, 39.9, 37.3, 30.2, 22.9.

HRMS (APCI): *m/z* calcd. for $C_{20}H_{21}N_2O^+$ 305.1648 [M+H]$^+$, found 305.1650 [M+H]$^+$.

IR: ṽ (cm^{-1}) = 2961, 2923, 1696, 1490, 1457, 1388, 1357, 1222, 1186, 1146, 1124.

R*f* (*n*-pentane/EtOAc 2:1, containing 0.5 Vol% *i*-PrNH_2) = 0.61.

6,6-Dimethyl-11-phenylhexahydro-1H,5H,9H-dipyrrolo[1,2-a:2',1'-b]pyrimidin-9-one (3ah/3ah')

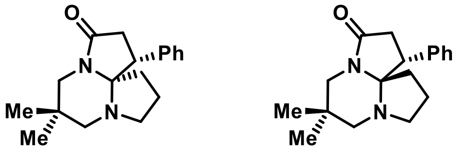

3ah 3ah'

A slightly modified version of **GP1** was followed. **2h** (46.6 mg, 0.20 mmol, 2.00 equiv.) was dissolved in CH_2Cl_2 (20 mL), washed with aq. NaOH (10%, 3 × 10 mL) and the aq. layers were extracted with CH_2Cl_2 (3 ×10 mL). The combined organic layers were dried over $MgSO_4$ and the solvent was removed under reduced pressure. In an oven-dried round-bottomed flask under argon (*rac*)-**1a** (35.1 mg, 0.13 mmol, 1.00 equiv.) was dissolved in CH_2Cl_2 (13 mL). This solution was added to the free amidine **2h**, which was placed in another oven-dried round-bottomed flask under argon. The resulting mixture was stirred for 17 h. Purification by fcc (*n*-pentane/EtOAc 1:1 to EtOAc, containing 0.5 Vol% *i*-PrNH_2) afforded **3ah** (12.1 mg, 42.6 µmol, 33%) and **3ah'** (3.9 mg, 13.7 µmol, 11%) as a faint-brown solid and a colorless oil, respectively (total yield: 16.0 mg, 56.3 µmol, 44%; 3:1 dr).

Analytical data of **3ah**:

^1H NMR (700 MHz, C_6D_6): δ = 7.06 – 7.01 (m, 5H), 4.04 – 3.99 (m, 1H), 3.23 (dd, *J* = 10.3, 8.7 Hz, 1H), 2.75 – 2.70 (m, 1H), 2.58 (dd, *J* = 16.4, 8.7 Hz, 1H), 2.52 (dd, *J* = 16.4, 10.2 Hz, 1H), 2.42 – 2.38 (m, 1H), 2.28 (d, *J* = 12.7 Hz, 2H), 2.14 (d, *J* = 12.7 Hz, 1H), 1.48 – 1.43 (m, 1H), 1.42 – 1.36 (m, 1H), 1.24 – 1.18 (m, 1H), 1.04 (s, 3H), 0.68 (s, 3H), 0.64 – 0.57 (m, 1H).

$^{13}C\{^1H\}$ NMR (176 MHz, C_6D_6): δ = 171.1, 140.0, 129.0, 128.3, 127.1, 86.3, 59.0, 52.1, 49.8, 46.0, 36.8, 32.6, 27.2, 26.5, 25.9, 21.5.

HRMS (APCI): *m/z* calcd. for $C_{18}H_{25}N_2O^+$ 285.1961 $[M+H]^+$, found 285.1962 $[M+H]^+$.

IR: ṽ (cm^{-1}) = 2957, 2927, 2868, 1694, 1453, 1419, 1393, 1319, 1101, 1082.

mp: 105 – 110 °C

$\mathbf{R_f}$ (EtOAc, containing 0.5 Vol% *i*-$PrNH_2$) = 0.28.

Analytical data of **3ah'**:

^{1}H NMR (700 MHz, C_6D_6): δ = 7.15 – 7.13 (m, 2H), 7.10 – 7.07 (m, 2H), 7.03 – 7.00 (m, 1H), 3.93 (d, *J* = 13.2 Hz, 1H), 2.61 – 2.55 (m, 2H), 2.52 – 2.44 (m, 2H), 2.23 (d, *J* = 13.4 Hz, 1H), 2.02 – 1.96 (m, 1H), 1.78 (s, 2H), 1.63 – 1.54 (m, 2H), 1.31 – 1.25 (m, 1H), 1.10 – 1.06 (m, 1H), 0.94 (s, 3H), 0.51 (s, 3H).

^{13}C{^{1}H} NMR (176 MHz, C_6D_6): δ = 171.6, 142.1, 128.7, 128.3, 127.0, 86.3, 61.4, 50.9, 49.6, 46.7, 38.9, 30.9, 27.7, 26.2, 23.7, 20.3.

HRMS (ESI): *m/z* calcd. for $C_{18}H_{25}N_2O^+$ 285.1961 $[M+H]^+$, found 285.1965 $[M+H]^+$.

IR: ṽ (cm^{-1}) = 3468, 2985, 2941, 2908, 1737, 1465, 1447, 1373, 1301, 1233, 1098, 1044.

$\mathbf{R_f}$ (EtOAc, containing 0.5 Vol% *i*-$PrNH_2$) = 0.58.

1-Methyl-8-phenylhexahydropyrrolo[1,2-a]pyrimidin-6(2H)-one (3ai/3ai')

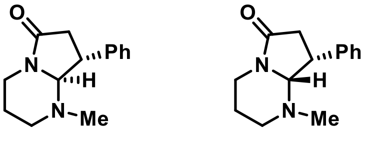

3ai 3ai'

GP1 was followed, starting with (*rac*)-**1a** (82.3 mg, 0.30 mmol, 1.00 equiv.) and **2i** for 20 h. Purification by fcc (CH_2Cl_2, containing 0.5 Vol% *i*-$PrNH_2$) afforded the title compound as a faint-brown oil and as a mixture of diastereomers **3ai** and **3ai'** (37.2 mg, 0.16 mmol, 54%; 2:1 dr), which were separated by another fcc (EtOAc, containing 0.5 Vol% *i*-$PrNH_2$).

Analytical data of **3ai**:

^{1}H NMR (700 MHz, C_6D_6): δ = 7.07 – 7.02 (m, 2H), 7.02 – 6.98 (m, 1H), 6.87 – 6.84 (m, 2H), 4.36 – 4.30 (m, 1H), 3.25 (d, *J* = 3.6 Hz, 1H), 3.01 (ddd, *J* = 9.9, 6.0, 3.6 Hz, 1H), 2.75 (dd,

J = 17.3, 10.2 Hz, 1H), 2.45 – 2.40 (m, 1H), 2.32 – 2.26 (m, 1H), 2.24 (dd, *J* = 17.3, 6.0 Hz, 1H), 1.77 (s, 3H), 1.62 – 1.57 (m, 1H), 1.57 – 1.52 (m, 1H), 0.96 – 0.91 (m, 1H).

^{13}C{^{1}H} NMR (176 MHz, C_6D_6): δ = 171.1, 144.4, 129.0, 127.4, 126.8, 85.5, 55.3, 43.1, 40.9, 39.6, 38.9, 24.5.

HRMS (ESI): *m/z* calcd. for $C_{14}H_{19}N_2O^+$ 231.1492 [M+H]$^+$, found 231.1496 [M+H]$^+$.

IR: ṽ (cm^{-1}) = 2935, 2854, 2789, 2164, 1692, 1497, 1448, 1370, 1281, 1133.

<u>Analytical data of **3ai'**:</u>

^{1}H NMR (700 MHz, C_6D_6): δ = 7.16 – 7.15 (m, 2H), 7.10 – 7.07 (m, 2H), 7.04 – 7.01 (m, 1H), 4.25 – 4.20 (m, 1H), 3.07 (d, *J* = 6.1 Hz, 1H), 2.86 (ddd, *J* = 9.0, 6.1, 2.0 Hz, 1H), 2.41 (dd, *J* = 16.9, 9.0 Hz, 1H), 2.30 (dd, *J* = 16.9, 2.0 Hz, 2H), 2.27 – 2.22 (m, 1H), 1.56 – 1.51 (m, 1H), 1.50 (s, 3H), 1.49 – 1.41 (m, 1H), 0.99 – 0.95 (m, 1H).

^{13}C{^{1}H} NMR (176 MHz, C_6D_6): δ = 171.8, 141.8, 129.0, 128.5, 126.8, 80.6, 54.7, 41.9, 40.1, 39.8, 39.1, 23.6.

HRMS (ESI): *m/z* calcd. for $C_{14}H_{19}N_2O^+$ 231.1492 [M+H]$^+$, found 231.1494 [M+H]$^+$.

IR: ṽ (cm^{-1}) = 3325, 2942, 2853, 2783 1692, 1455, 1438, 1418, 1369, 1288, 1141.

R$_f$ (EtOAc, containing 0.5 Vol% *i*-$PrNH_2$) = 0.38.

1-Methyl-8-phenyl-8a-((E)-2-(thienyl)vinyl)hexahydropyrrolo[1,2-a]pyrimidin-6(2H)-one (3aj/3aj')

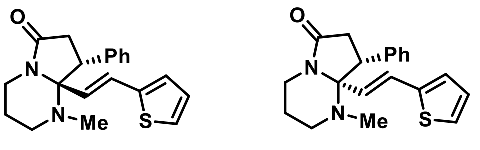

3aj 3aj'

A slightly modified version of **GP1** was followed. Pyrantel pamoate (168 mg, 0.28 mmol, 1.99 equiv.) was dissolved in CH_2Cl_2 (20 mL), washed with aq. NaOH (10%, 3 × 10 mL) and the aq. layers were extracted with CH_2Cl_2 (3 ×10 mL). The combined organic layers were dried over $MgSO_4$ and the solvent was removed under reduced pressure. In an oven-dried round-bottomed flask under argon (*rac*)-**1a** (38.6 mg, 0.14 mmol, 1.00 equiv.) was dissolved in CH_2Cl_2 (15 mL). This solution was added to the free pyrantel, which was placed in another oven-dried round-bottomed flask under argon. The resulting mixture was stirred for 17 h.

Purification by fcc (*n*-pentane/EtOAc 1:2 to EtOAc, containing 0.5 Vol% *i*-PrNH_2) afforded **3aj** (30.5 mg, 90.1 µmol, 64%) and **3aj'** (11.6 mg, 34.3 µmol, 24%) as a colorless solid and a faint-brown oil, respectively (total yield: 42.1 mg, 12.4 µmol, 88%; 3:1 dr).

Analytical data of **3aj**:

^{1}H NMR (500 MHz, $CDCl_3$): δ = 7.31 – 7.26 (m, 4H), 7.26 – 7.22 (m, 1H), 7.22 – 7.20 (m, 1H), 7.03 – 6.97 (m, 2H), 6.54 (d, *J* = 16.1 Hz, 1H), 6.33 (d, *J* = 16.0 Hz, 1H), 4.29 – 4.20 (m, 1H), 3.49 – 3.43 (m, 1H), 2.99 – 2.88 (m, 2H), 2.64 – 2.54 (m, 2H), 2.40 – 2.33 (m, 1H), 2.03 (s, 3H), 1.88 – 1.74 (m, 1H), 1.65 – 1.56 (m, 1H).

^{13}C{^{1}H} NMR (126 MHz, $CDCl_3$): δ = 173.5, 141.3, 141.1, 128.7, 128.0, 127.7, 126.9, 126.8, 125.6, 124.9, 123.8, 82.7, 49.5, 48.4, 38.8, 37.9, 37.1, 24.1.

HRMS (ESI): *m/z* calcd. for $C_{20}H_{23}N_2OS^+$ 339.1526 [M+H]$^+$, found 339.1530 [M+H]$^+$.

IR: ṽ (cm^{-1}) = 2939, 2797, 1689, 1455, 1427, 1410, 1368, 1288, 1243, 1147, 1053.

mp: Decomposition at 170 °C.

R*f* (EtOAc, containing 0.5 Vol% *i*-PrNH_2) = 0.73.

Analytical data of **3aj'**:

^{1}H NMR (700 MHz, C_6D_6): δ = 7.05 – 7.03 (m, 2H), 7.02 – 6.99 (m, 2H), 6.98 – 6.95 (m, 1H), 6.68 – 6.66 (m, 1H), 6.63 – 6.60 (m, 1H), 6.59 – 6.57 (m, 1H), 6.44 (d, *J* = 16.2 Hz, 1H), 5.75 (d, *J* = 16.2 Hz, 1H), 4.34 – 4.29 (m, 1H), 3.53 – 3.49 (m, 1H), 2.74 – 2.69 (m, 1H), 2.64 – 2.59 (m, 1H), 2.54 – 2.48 (m, 1H), 2.39 – 2.33 (m, 1H), 2.21 – 2.17 (m, 1H), 2.10 (s, 3H), 1.64 – 1.56 (m, 1H), 0.90 – 0.85 (m, 1H).

^{13}C{^{1}H} NMR (176 MHz, C_6D_6): δ = 171.7, 141.7, 139.3, 129.8, 128.4, 128.3, 127.7, 127.2, 126.6, 126.3, 125.3, 124.9, 82.8, 50.4, 37.7, 37.7, 37.1, 22.6.

HRMS (ESI): *m/z* calcd. for $C_{20}H_{23}N_2OS^+$ 339.1526 [M+H]$^+$, found 339.1531 [M+H]$^+$.

IR: ṽ (cm^{-1}) = 3367, 3066, 3031, 2952, 2926, 2854, 2802, 1689, 1454, 1413, 1371, 1289, 1209, 1145.

R*f* (EtOAc, containing 0.5 Vol% *i*-PrNH_2) = 0.24.

4. Follow-Up Procedures

1-Phenylhexahydro-1H,5H,9H-dipyrrolo[1,2-a:2',1'-b]pyrimidine (4)

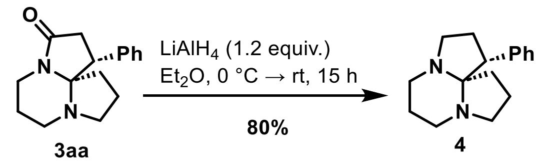

The reaction was carried out following a modified literature procedure.[10]

In an oven-dried round-bottomed flask under an argon atmosphere, **3aa** (25.6 mg, 0.10 mmol, 1.00 equiv.; single diastereomer) was dissolved in dry Et_2O (1.0 mL). The solution was cooled to 0 °C and $LiAlH_4$ (0.12 mL, 0.12 mmol, 1.20 equiv.; 1 M solution in THF) was added. The reaction mixture was stirred for 15 h while being allowed to warm to room temperature. Then, the mixture was again cooled to 0 °C and water (0.2 mL), aq. NaOH (10%; 0.2 mL) and water (0.6 mL) were added sequentially. The mixture was extracted with Et_2O (2 × 5 mL) and the combined organic layers were dried over $MgSO_4$. The mixture was filtered through a pad of celite, while eluting with Et_2O (20 mL). The solvent was removed under reduced pressure, yielding **4** (19.5 mg, 80.5 µmol, 80%) as a colorless oil.

^{1}H NMR (500 MHz, C_6D_6): δ = 7.41 – 7.36 (m, 2H), 7.27 – 7.22 (m, 2H), 7.17 – 7.12 (m, 1H), 3.45 – 3.39 (m, 1H), 3.08 – 3.00 (m, 1H), 2.95 – 2.83 (m, 3H), 2.82 – 2.75 (m, 1H), 2.75 – 2.68 (m, 1H), 2.65 – 2.55 (m, 2H), 2.24 – 2.14 (m, 1H), 1.96 – 1.85 (m, 1H), 1.71 – 1.53 (m, 3H), 1.53 – 1.47 (m, 1H), 1.36 – 1.28 (m, 1H), 1.21 – 1.11 (m, 1H).

^{13}C{^{1}H} NMR (126 MHz, C_6D_6): δ = 142.8, 129.1, 128.3, 126.2, 88.4, 52.1, 50.5, 47.5, 46.3, 44.9, 31.3, 27.4, 21.9, 19.6.

HRMS (ESI): *m/z* calcd. for $C_{16}H_{23}N_2^+$ 243.1856 $[M+H]^+$, found 243.1861 $[M+H]^+$.

IR: ṽ (cm^{-1}) = 3058, 3026, 2938, 2884, 1451, 1276, 1161, 1090, 1056, 1034, 760, 698.

R_f (EtOAc, containing 0.5 Vol% *i*-$PrNH_2$) = 0.23.

11-Phenylhexahydro-1H,5H,9H-dipyrrolo[1,2-a:2',1'-b]pyrimidine-9-thione (5)

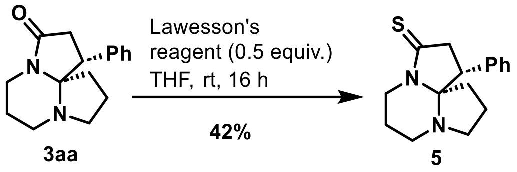

The reaction was carried out following a modified literature procedure.[11]

In an oven-dried round-bottomed flask under an argon atmosphere, Lawesson's reagent[7] (21.7 mg, 53.7 mmol, 0.54 equiv.) was dissolved in dry THF (4.0 mL). A solution of **3aa** (25.6 mg, 0.10 mmol, 1.00 equiv.; single diastereomer) in dry THF (1.8 mL) was added at room temperature and the resulting mixture was stirred at that temperature for 16 h. The mixture was treated with water (5 mL) and the aqueous layer was extracted with Et_2O (2 × 5 mL). The combined organic layers were dried over $MgSO_4$ and the solvent was removed under reduced pressure. The crude product was adsorbed to celite and purified *via* fcc (*n*-pentane/EtOAc 5:1, containing 0.5 Vol% *i*-$PrNH_2$) to yield **5** (11.3 mg, 41.5 µmol, 42%) as a colorless oil.

^{1}H NMR (500 MHz, C_6D_6): δ = 7.09 – 7.04 (m, 5H), 5.02 – 4.92 (m, 1H), 3.21 (dd, *J* = 12.7, 7.2 Hz, 1H), 3.08 (dd, *J* = 16.4, 7.3 Hz, 1H), 2.99 (dd, *J* = 16.4, 12.7 Hz, 1H), 2.59 – 2.49 (m, 2H), 2.44 – 2.29 (m, 3H), 1.55 – 1.44 (m, 2H), 1.37 – 1.27 (m, 1H), 1.07 – 0.99 (m, 1H), 0.96 – 0.89 (m, 1H), 0.63 – 0.52 (m, 1H).

^{13}C{^{1}H} NMR (126 MHz, C_6D_6): δ = 197.2, 136.8, 128.9, 128.1 (overlaid with solvent signal), 127.3, 91.6, 50.1, 49.6, 45.6, 38.8, 36.2, 29.8, 22.8, 18.1.

HRMS (APCI): *m/z* calcd. for $C_{16}H_{21}N_2S^+$ 273.1420 $[M+H]^+$, found 273.1420 $[M+H]^+$.

IR: ṽ (cm^{-1}) = 2942, 2870, 1489, 1451, 1425, 1312, 1287, 1199, 1157, 1058, 1037.

R*f* (EtOAc, containing 0.5 Vol% *i*-$PrNH_2$) = 0.83.

[7] 2,4-Bis(4-methoxyphenyl)-1,3,2,4-dithiadiphosphetane-2,4-dithione

10-Methyl-11-phenylhexahydro-1H,5H,9H-dipyrrolo[1,2-a:2',1'-b]pyrimidin-9-one (6)

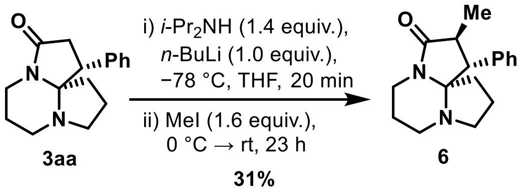

The reaction was carried out following a modified literature procedure.[12]

In an oven-dried round-bottomed flask under an argon atmosphere, *n*-BuLi (0.04 mL, 0.10 mmol, 1.01 equiv.; 2.5 M solution in hexanes) was added towards a stirred solution of *i*-Pr_2NH (0.02 mL, 0.14 mmol, 1.44 equiv.) in dry THF (0.16 mL) at −78 °C. The mixture was allowed to warm to 0 °C and was stirred at that temperature for 15 min. It was then again cooled to −78 °C and a solution of **3aa** (25.3 mg, 98.7 µmol, 1.00 equiv.; single diastereomer) in dry THF (0.25 mL) was added dropwise over 5 min. The resulting mixture was stirred at −78 °C for 20 min. The mixture was allowed to warm to 0 °C and MeI (0.01 mL, 0.16 mmol, 1.63 equiv.) was added dropwise. The mixture was stirred at 0 °C for 2 h and was then stirred for another 23 h at room temperature. The reaction was quenched by adding aq. sat. NH_4Cl (1 mL). The aqueous layer was extracted with EtOAc (3 × 5 mL). The combined organic layers were washed with brine (10 mL), dried over Na_2SO_4 and the solvent was removed under reduced pressure. Purification *via* fcc (*n*-pentane/EtOAc 5:1 to 2:1, containing 0.5 Vol% *i*-$PrNH_2$) yielded **6** (8.4 mg, 31 µmol, 31%; >99:1 dr) as a colorless oil.

^{1}H NMR (700 MHz, C_6D_6): δ = 7.18 – 7.15 (m, 2H), 7.13 – 7.10 (m, 2H), 7.09 – 7.06 (m, 1H), 4.28 – 4.24 (m, 1H), 3.03 – 2.99 (m, 1H), 2.74 – 2.68 (m, 1H), 2.64 – 2.58 (m, 1H), 2.50 (dd, *J* = 7.1 Hz, 1H), 2.45 – 2.35 (m, 4H), 1.57 – 1.51 (m, 1H), 1.45 – 1.38 (m, 1H), 1.28 (d, *J* = 7.0 Hz, 3H), 1.24 – 1.16 (m, 2H), 1.05 – 1.00 (m, 1H), 0.62 – 0.54 (m, 1H).

^{13}C{^{1}H} NMR (176 MHz, C_6D_6): δ = 172.3, 137.3, 129.3, 128.5, 127.2, 83.7, 58.5, 50.0, 40.3, 38.7, 31.3, 29.7, 22.7, 18.0, 13.9.

HRMS (ESI): *m/z* calcd. for $C_{17}H_{23}N_2O^+$ 271.1805 $[M+H]^+$, found 271.1806 $[M+H]^+$.

IR: ṽ (cm^{-1}) = 3434, 3030, 2943, 2871, 1691, 1453, 1424, 1281, 1173, 1139, 1073, 1044.

R_f (*n*-pentane/EtOAc 2:1, containing 0.5 Vol% *i*-$PrNH_2$) = 0.45.

5. Supplementary Data for Screening and Design of Experiments

Solvent screening for the reaction of SCP 1a with amidine 2a

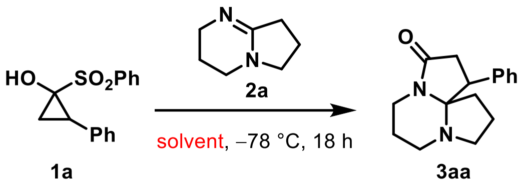

A microwave vial was equipped with a magnetic stir bar and **1a** (27 mg, 0.1 mmol, 1.0 equiv.) and was capped, evacuated and backfilled with argon. Dry solvent (2.0 mL, 0.05 M with respect to **1a**) was added *via* syringe and the solution was cooled to –78 °C. To this stirred solution, **2a** (0.1 mL, 0.8 mmol, 8.1 equiv) was added *via* syringe and the resulting mixture was stirred for 18 h, while being allowed to warm to room temperature. The vial was opened, and the mixture was diluted with CH_2Cl_2 (5 mL), washed with 10% aq. NaCl (10 mL) and the aq. layers were extracted with CH_2Cl_2 (2 × 10 mL). The combined organic layers were dried over $MgSO_4$, and the solvent was removed *in vacuo*. The residue was transferred with $CDCl_3$-washes (1.0 mL) to an NMR tube charged with 1,3,5-trimethoxybenzene (40 µmol). The extent of conversion was determined by integration of the product multiplet at 0.71-0.91 ppm with respect to the aromatic singlet at 6.07 ppm of the internal standard (see Figure 4 for an exemplary ^{1}H NMR spectrum).

Table 1: Results of the solvent screening.

Entry	Solvent	NMR yield / %
1	DMF	25
2	PhMe	16
**3**	**THF**	**31**
4	1,4-Dioxane	26
5	Et_2O	16
6	CH_2Cl_2	11
7	MeCN	13
8	DMSO	13

Design of Experiments (DoE) for the reaction of SCP 1a with amidine 2a

Design of Experiments was carried out using MODDE Pro 13.1 by Sartorius. Using a quadratic CCF model (central composite factorial design), three factors were defined, which were assumed to be affecting the outcome of the reaction the most: The initial concentration of SCP, defined as $[SCP]_0$, the equivalents of DBN with respect to the SCP and the temperature *T*. Each factor was assigned a minimum- (= –1), a center- (= 0) and a maximum- (= +1) value:

Table 2: Overview on the selected values for the factors in the experimental design.

Value / a.u.	Initial conc. 1a / mol L^{-1}	Equiv. 2a	*T* / °C
–1	0.025	1.0	–78
0	0.050	2.0	–40
+1	0.075	3.0	0

The experimental conditions generated by the software were then carried out according to the following procedure:

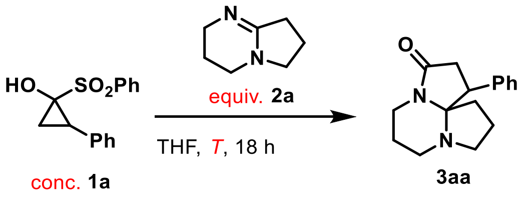

A microwave vial was equipped with a magnetic stir bar and **1a** (27 mg, 0.1 mmol, 1.0 equiv) and was capped, evacuated and backfilled with argon. Dry THF was added *via* syringe to reach the required initial concentration of **1a**, and the solution was cooled to the respective temperature. To this solution was added a stock solution of **2a** in dry THF (0.4 M solution) *via* syringe and the resulting solution was stirred at 320 rpm for 18 h, while being allowed to warm to room temperature. The vial was opened, and the mixture was diluted with CH_2Cl_2 (5 mL), washed with 10% aq. NaCl (10 mL) and the aq. layers were extracted with CH_2Cl_2 (2 × 10 mL). The combined organic layers were dried over $MgSO_4$, and the solvent was removed *in vacuo*. The residue was dissolved in a stock solution of 1,3,5-trimethoxybenzene in $CDCl_3$ (7.1 g L^{-1} or 0.63 g L^{-1}; 0.8 mL) and the resulting solution was transferred to an NMR tube. The extent of conversion was determined by integration of the product multiplet at 0.71-0.91 ppm with respect to the aromatic singlet at 6.07 ppm of the internal standard (see Figure 4 for an exemplary ^{1}H NMR spectrum).

Table 3: Results of the screening conditions generated by the DoE software.

Entry	Initial conc. 1a / mol L^{-1}	Equiv. 2a	*T* / °C	NMR yield / %
1	0.050	2.0	–40	32
2	0.075	3.0	–78	32
3	0.050	2.0	0	38
4	0.075	1.0	0	0
5	0.050	3.0	–40	37
6	0.050	1.0	–40	0
7	0.025	3.0	–78	50
**8**	**0.025**	**3.0**	**0**	**74**
9	0.050	2.0	–78	24
10	0.075	1.0	–78	0
11	0.025	1.0	–78	0
12	0.075	3.0	0	28
13	0.075	2.0	–40	17
14	0.050	2.0	–40	20
15	0.050	2.0	–40	29
16	0.025	2.0	–40	46
17	0.025	1.0	0	0

Table 4: Results of further consecutive screening.

Entry	Initial conc. 1a / mol L^{-1}	Equiv 2a	*T* / °C	NMR yield / %
1	0.025	6.0	0 °C	78
2	0.010	3.0	0 °C	89
3	0.025	3.0	rt	76
4	0.010	6.0	0 °C	84
5	0.025	6.0	rt	84
6	0.010	6.0	rt	92
7	0.010	3.0	rt	81
8	0.010	10.0	rt	77
9	0.005	6.0	rt	84
10	0.010	2.0	rt	89

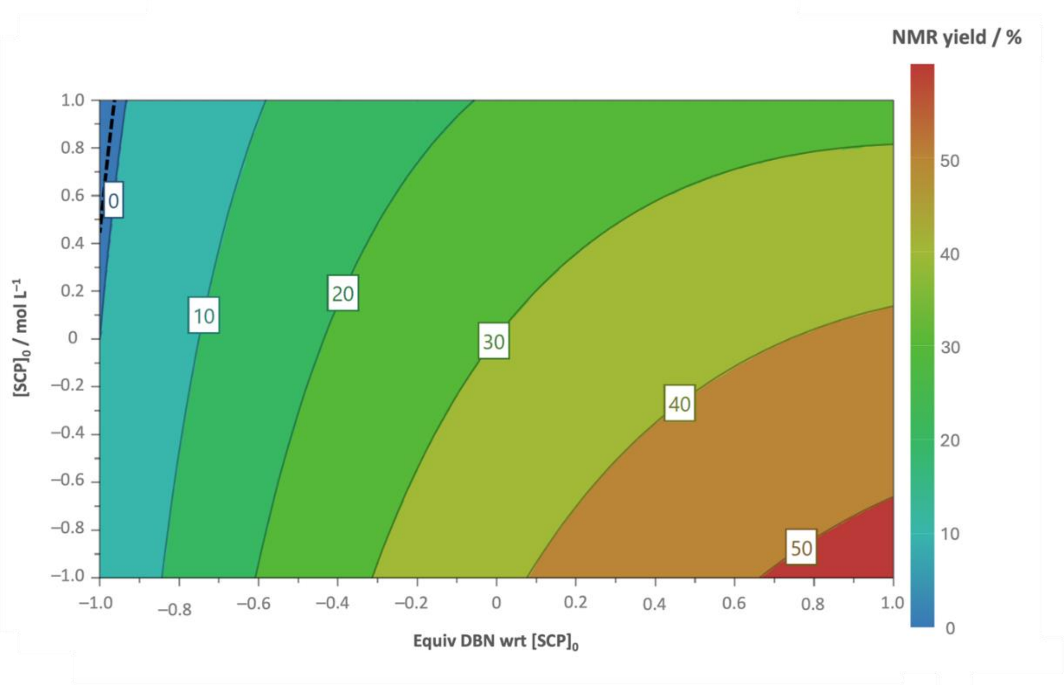

Figure 3: 2D response contour plot of the results of the Design of Experiments screening. On the y-axis is shown the initial concentration of 1a, $[SCP]_0$, the x-axis represents the equivalents of 2a. The response, NMR yield, is displayed as a color gradient. Created with MODDE Pro 13.1.

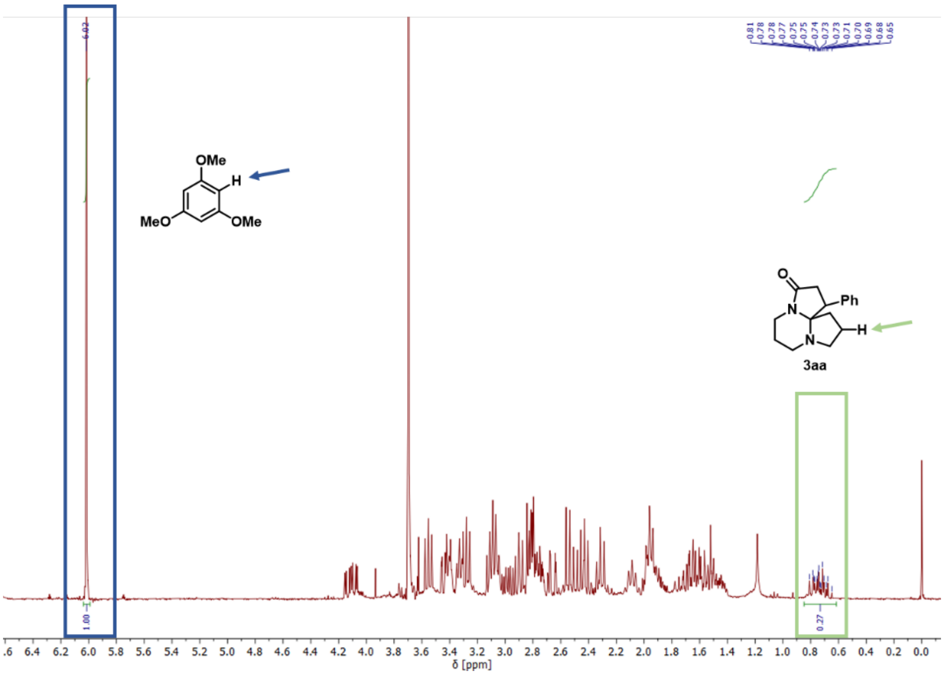

Figure 4: Exemplary ^{1}H NMR spectrum of a crude mixture from a screening condition with highlighted integrals of the internal standard signal (blue) and product signal (green).

6. Mechanistic Investigations

Stereospecificity Experiments

Separation of SCPs *rac*-1a and (1*S*,2*R*)-1a:

Enantiomeric excess was determined using an Agilent 1260 Infinity column with a G1311B-1260 quaternary pump. A Chiralpak AD-3 column (3 µm; 150 × 4.6 mm) was used with *n*-heptane/EtOH (85/15) eluent mixtures. Detection was conducted at 230 nm using a 1290 Infinity II DAD detector. Flow rate: 0.5 mL min^{-1}. Column temperature: Hold at 22 °C.

Separation of products 3aa and 3aa', obtained from *rac*-1a *or* (1*S*,2*R*)-1a:

Enantiomeric excess was determined using an Agilent 1260 Infinity column with a G1311B-1260 quaternary pump. A Chiralcel OD-3 column (3 µm; 150 × 4.6 mm) was used with *n*-heptane/EtOH (85/15) eluent mixtures. Detection was conducted at 220 or 212 nm using a 1290 Infinity II DAD detector. Flow rate: 0.5 mL min^{-1}. Column temperature: Hold at 22 °C.

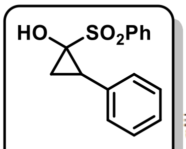

(*rac*)-**1a**

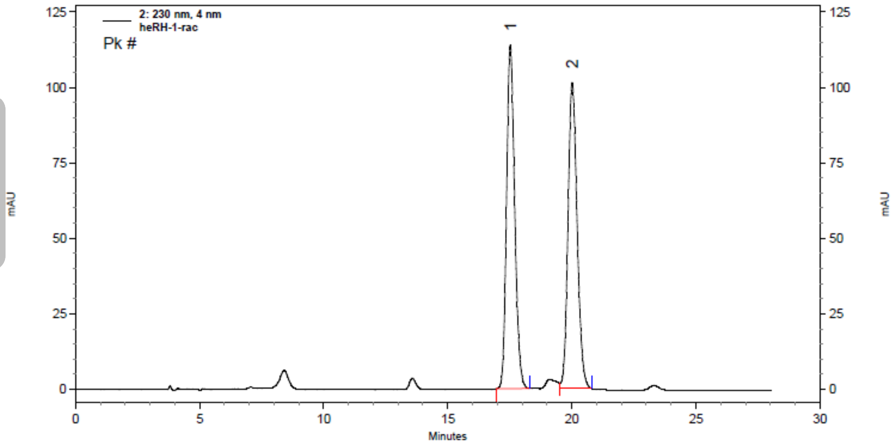

2: 230 nm, 4 nm Results

Pk #	Retention Time	Area Percent	Lambda Max
1	17,520	50,060	204
2	20,020	49,940	204

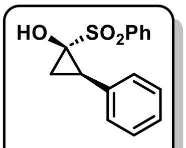

(1*S*,2*R*)-**1a**

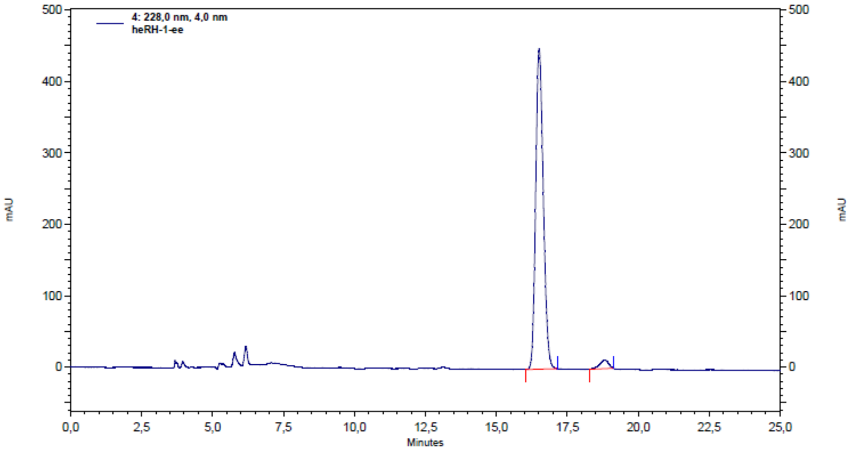

4: 228,0 nm, 4,0 nm
Results

Peak Number	Retention Time	Area Percent	Area
1	16,512	97,043	1128893214
2	18,818	2,957	34398691
Totals		100,000	1163291905

Chromatograms of product 3aa, obtained from (*rac*)-1a (top) and (1*S*,2*R*)-1a (bottom):

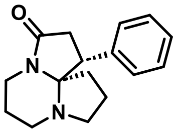

3aa
(from (*rac*)-**1a**)

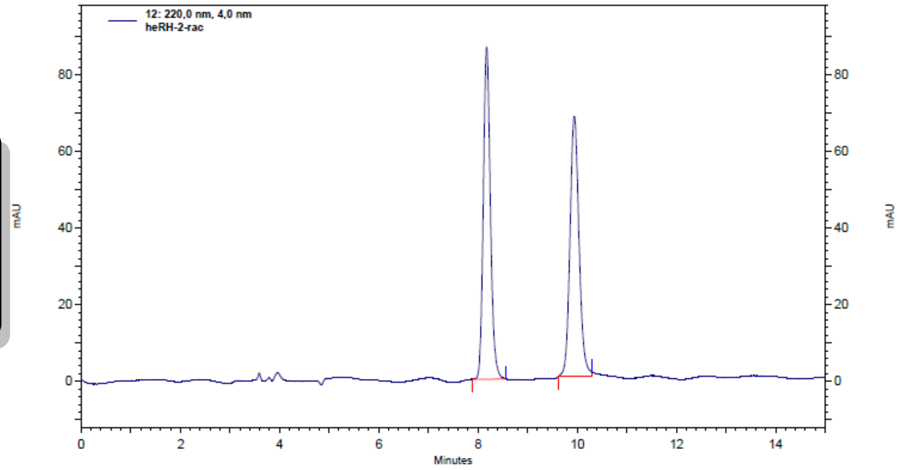

12: 220,0 nm, 4,0 nm
Results

Peak Number	Retention Time	Area Percent	Area
1	8,173	50,099	115832751
2	9,940	49,901	115374758
Totals		100,000	231207509

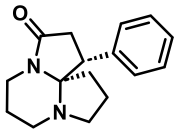

3aa
(from (1*S*,2*R*)-**1a**)

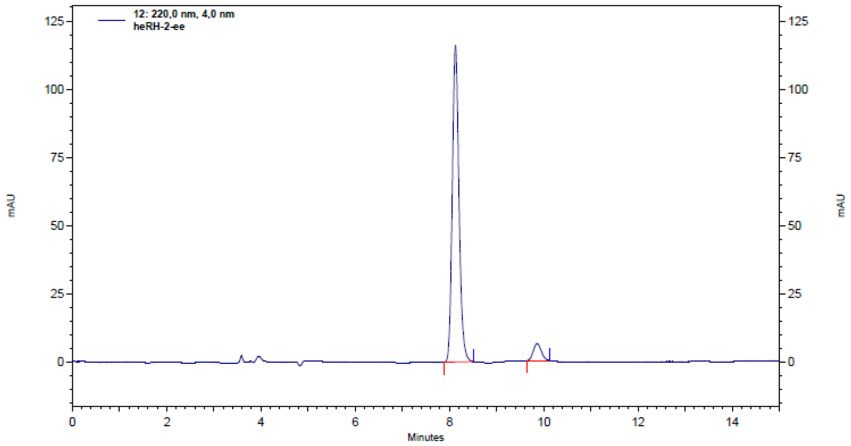

12: 220,0 nm, 4,0 nm
Results

Peak Number	Retention Time	Area Percent	Area
1	8,127	93,991	154360620
2	9,867	6,009	9868617
Totals		100,000	164229237

Chromatograms of product 3aa', obtained from (*rac*)-1a (top) and (1*S*,2*R*)-1a (bottom):

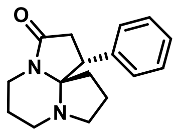

3aa'
(from (*rac*)-**1a**)

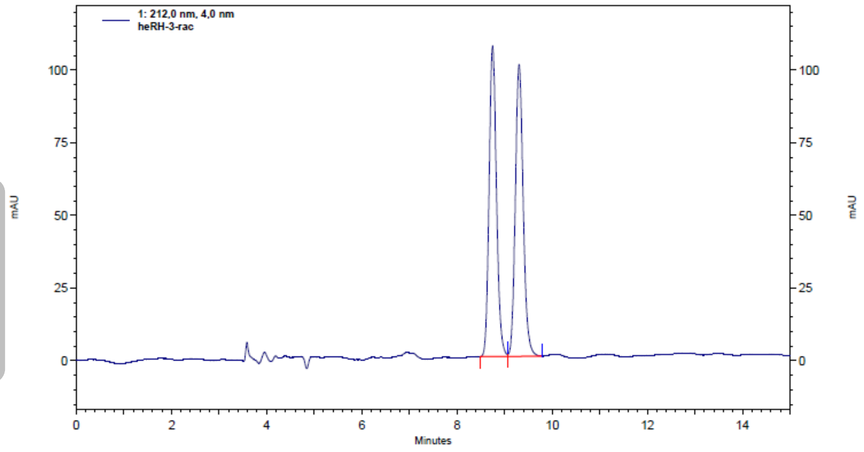


```
1: 212,0 nm, 4,0 nm
Results
```

Peak Number	Retention Time	Area Percent	Area
1	8,753	49,707	153789812
2	9,307	50,293	155603333
Totals		100,000	309393145

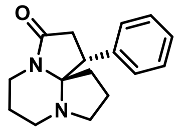

3aa'
(from (1*S*,2*R*)-**1a**)

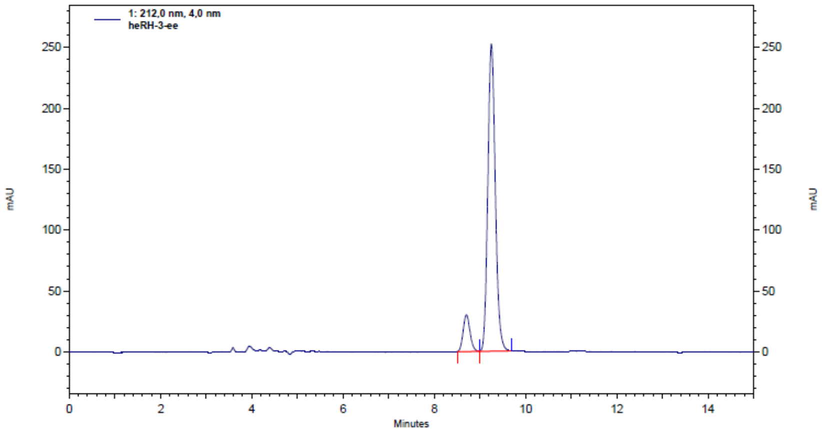


```
1: 212,0 nm, 4,0 nm
Results
```

Peak Number	Retention Time	Area Percent	Area
1	8,707	9,874	42245994
2	9,253	90,126	385586238
Totals		100,000	427832232

Supplementary Data for DFT Calculations

Methods

All calculations were performed with the ORCA program (versions 6.0.1).[13–15] Structures were pre-optimized by Grimme's tight-binding method XTB as implemented in the ORCA program suite.[16,17] In order to identify the global minimum, the pre-optimized structures were subjected to the GOAT module of ORCA. The global minimum geometry was further optimized by r^2scan-3c.[18,19] For the calculation of free energy contributions of translation, rotations and harmonic vibrations (G^{RRHO}, computed with r^2scan-3c) the temperature was set to 298°K and the quasi-RRHO approach for low-energy frequencies was chosen.[20] Single point calculations were performed with the hybrid functional PW6B95, def2-TZVP as basis set[21,22] and D4 as dispersion correction.[23–25] Solvent effects were taken into account by applying the SMD solvation model.[26] Wiberg bond indices were calculated by performing a natural bond orbital analysis (Keyword: NBO).[27]

The SCF convergence criteria were set to tight (Keyword: TightSCF) and DefGrid3 was used as integration grid in ORCA.

Proposed Mechanism

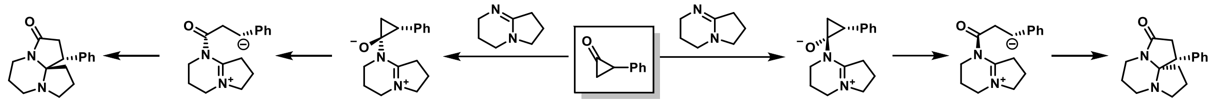

3aa' II' I' CP I II 3aa

Species	E(PW6B95) [E_h]	G^{RRHO}_{298} [E_h]	ΔG [kcal/mol]	ΔΔG [kcal/mol]
CP	-423.66934011	0.10851553	0.0	13.7
DBN	-384.12784302	0.15409168	0.0	
TS1’	-807.79850601	0.28577510	13.7	
I’	-807.81481056	0.29063509	6.5	1.8
TS2’	-807.81097341	0.28958511	8.3	
II’	-807.81360183	0.28951899	6.6	7.3
TS3aa’	-807.80236214	0.28989747	13.9	
3aa’	-807.87909314	0.29504752	-31.0	
TS1	-807.79863085	0.28602672	13.6	13.6
I	-807.81319321	0.28973704	7.0	2.1
TS2	-807.80886885	0.28870253	9.0	
II	-807.81204929	0.28890856	7.2	6.8
TS3aa	-807.80283678	0.29049744	14.0	
3aa	-807.88387811	0.29465863	-34.3	

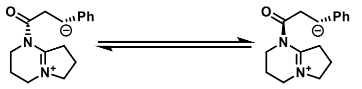

II' II

Species	E(PW6B95) [E_h]	G^{RRHO}_{195} [E_h]	ΔG [kcal/mol]
II’	-807.81360183	0.28951899	0.0
TS4	-807.80402518	0.28884224	5.6
II	-807.81204929	0.28890856	0.6

Intermediate II’ can be converted into intermediate **II** by rotation.

Extract of the Wiberg bond index matrix of **II**:

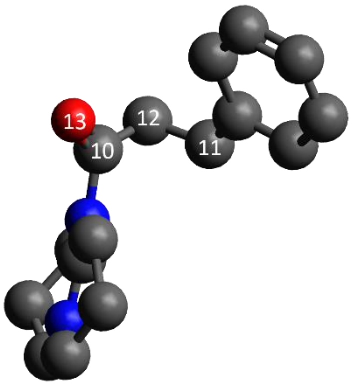

	**10**	**11**	**12**	**13**
**10**	0.0000	0.1849	0.9835	1.6733
**11**	0.1849	0.0000	1.0075	0.0872
**12**	0.9835	1.0075	0.0000	0.0524
**13**	1.6733	0.0872	0.0524	0.0000

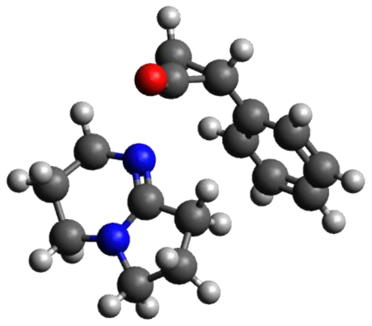

TS1’

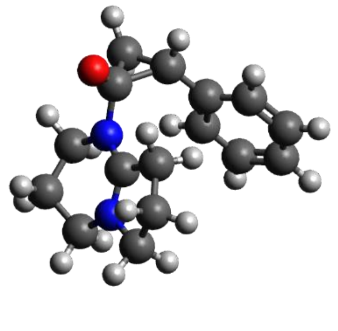

I’

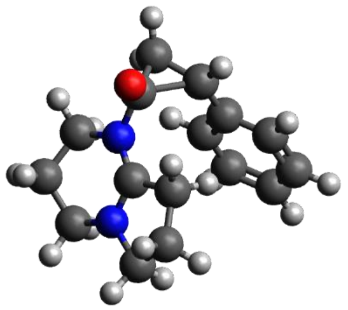

TS2’

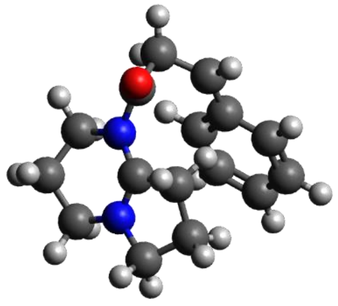

II’

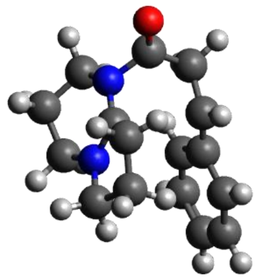

TS3aa’

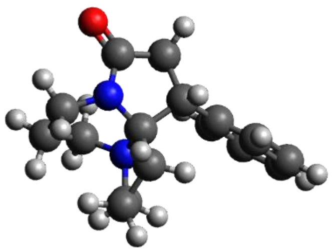

3aa’

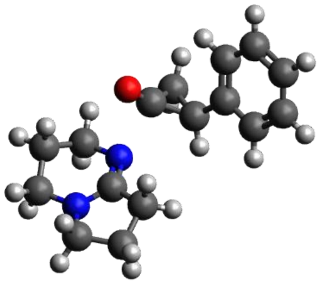

TS1

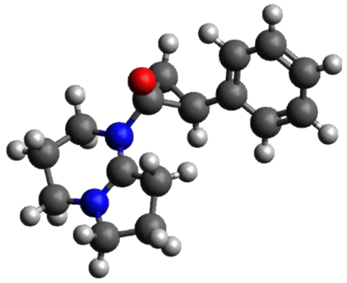

I

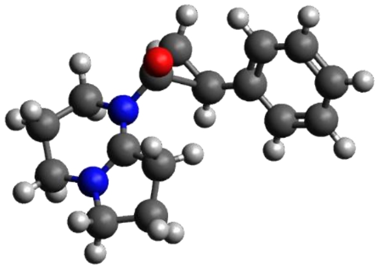

TS2

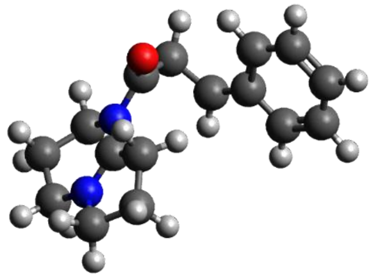

II

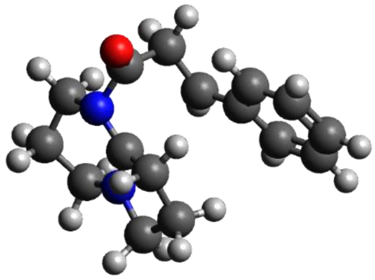

TS3aa

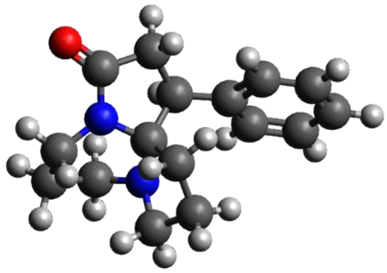

3aa

Figure 5: DFT-optimized (r^2scan-3c) intermediates and transition state structures.

DFT optimized (r^2scan-3c) cartesian coordinates

CP

E(r^2scan-3c) = -422.84104755 (conv)
Lowest Freq. = 39.13 cm^{-1}

C	-2.1642	-0.1825	-0.1042
C	-2.1751	-1.5731	-0.0307
C	-0.9698	-2.2665	0.0069
C	0.2370	-1.5752	-0.0230
C	0.2577	-0.1794	-0.0904
C	-0.9601	0.5093	-0.1384
C	1.5550	0.5420	-0.1100
C	1.6786	1.8953	-0.6930
C	1.7877	1.8397	0.7598
O	1.5900	2.5713	-1.6780
H	-3.1000	0.3675	-0.1451
H	-3.1175	-2.1119	-0.0074
H	-0.9666	-3.3514	0.0617
H	1.1753	-2.1232	0.0109
H	-0.9708	1.5940	-0.2191
H	2.4377	-0.0929	-0.1811
H	2.7604	1.9301	1.2382
H	0.9467	2.2080	1.3468

DBN

E(r^2scan-3c) = -383.36461767 (conv)
Lowest Freq. = 36.65 cm^{-1}

H	1.4257	2.2418	-1.2398
H	0.5400	3.2708	-0.1264
C	1.5174	0.0386	0.6039
C	1.4493	1.5500	0.8160
C	0.7489	2.2209	-0.3705
H	0.8857	1.7496	1.7359
H	2.4580	1.9575	0.9497
H	1.8988	-0.4695	1.5002
H	2.2012	-0.2046	-0.2298
C	-0.1727	-1.8342	0.2396
C	-1.6829	-1.7808	-0.0352
C	-1.8473	-0.4897	-0.8528
H	0.3685	-2.3346	-0.5851
H	0.0758	-2.3585	1.1713
H	-2.2211	-1.6977	0.9149
H	-2.0427	-2.6719	-0.5558
H	-1.7630	-0.6839	-1.9294
H	-2.7956	0.0267	-0.6870
C	-0.6695	0.3661	-0.4359
N	-0.5084	1.5868	-0.7790
N	0.1729	-0.4244	0.3272

TS1’

E(r^2scan-3c) = -806.24321746 (conv)
Lowest Freq. = -61.44 cm^{-1}

C	-1.0232	-2.6622	0.2852
C	-0.2509	-3.5414	1.2715
C	1.2299	-3.5742	0.9040
N	1.6827	-2.2018	0.7726
C	0.8426	-1.2343	0.2934
N	-0.4177	-1.3457	0.0549
C	3.0882	-1.8234	0.6797
C	3.0131	-0.2910	0.7054
C	1.6603	0.0126	0.0422
C	-2.1276	0.4321	0.4651
C	-2.1071	1.2068	-0.8164
C	-3.0382	-0.0267	-0.5831
O	-1.9986	0.5729	1.6578
C	-1.0577	1.2614	-1.8637
C	-0.2790	2.4237	-1.9643
C	0.7365	2.5256	-2.9090
C	0.9898	1.4693	-3.7827
C	0.2099	0.3175	-3.7029
C	-0.8051	0.2143	-2.7551
H	-2.0466	-2.5098	0.6551
H	-1.1189	-3.1851	-0.6805
H	-0.3581	-3.1370	2.2861
H	-0.6584	-4.5582	1.2741
H	1.8195	-4.0724	1.6840
H	1.3853	-4.1286	-0.0370
H	3.6582	-2.2418	1.5177
H	3.5355	-2.1926	-0.2591
H	3.8564	0.1744	0.1896
H	3.0085	0.0561	1.7442
H	1.1675	0.9072	0.4314
H	1.7664	0.1419	-1.0431
H	-2.5947	2.1716	-0.6545
H	-4.1072	0.1460	-0.4593
H	-2.7738	-0.9224	-1.1354
H	-0.4717	3.2530	-1.2870
H	1.3300	3.4347	-2.9649
H	1.7815	1.5463	-4.5231
H	0.3902	-0.5093	-4.3854
H	-1.3977	-0.6939	-2.7097

I’

E(r^2scan-3c) = -806.26191571 (conv)
Lowest Freq. = 41.55 cm^{-1}

C	-1.2126	-2.5003	0.2346
C	-0.2690	-3.5305	0.8423
C	1.1467	-3.3394	0.3117
N	1.5172	-1.9465	0.5198
C	0.6242	-0.9577	0.5069
N	-0.6667	-1.1359	0.3464
C	2.9003	-1.4576	0.5762
C	2.7226	-0.0395	1.1314
C	1.3122	0.3633	0.6682
C	-1.6286	0.0169	0.5982
C	-1.6787	1.0363	-0.6605
C	-2.7639	0.0029	-0.3815
O	-1.6926	0.4332	1.8073
C	-0.8077	0.9902	-1.8321
C	-0.0407	2.1279	-2.1646
C	0.8613	2.1143	-3.2203
C	1.0346	0.9617	-3.9897
C	0.2744	-0.1673	-3.6881
C	-0.6347	-0.1551	-2.6340
H	-2.1765	-2.5088	0.7535
H	-1.3988	-2.7286	-0.8237
H	-0.2625	-3.4301	1.9340
H	-0.6247	-4.5355	0.5976
H	1.8607	-3.9710	0.8508
H	1.2081	-3.5889	-0.7577
H	3.5001	-2.1080	1.2207

H	3.3387	-1.4593	-0.4327
H	3.4933	0.6447	0.7711
H	2.7623	-0.0661	2.2251
H	0.7660	1.0067	1.3612
H	1.3315	0.8563	-0.3120
H	-1.8891	2.0374	-0.2904
H	-3.7138	0.3798	-0.0086
H	-2.8547	-0.8031	-1.1082
H	-0.1606	3.0328	-1.5716
H	1.4341	3.0106	-3.4473
H	1.7406	0.9489	-4.8154
H	0.3839	-1.0695	-4.2858
H	-1.2171	-1.0500	-2.4351

TS2’

E(r^{2}scan-3c) = -806.26006724 (conv)
Lowest Freq. = -260.21 cm^{-1}

C	-1.2166	-2.5068	0.2058
C	-0.2334	-3.5483	0.7229
C	1.1656	-3.2737	0.1859
N	1.5006	-1.8886	0.4888
C	0.5844	-0.9299	0.5779
N	-0.7085	-1.1394	0.4383
C	2.8713	-1.3613	0.5105
C	2.6836	0.0146	1.1598
C	1.2367	0.3984	0.8056
C	-1.6960	-0.0566	0.7314
C	-1.7147	1.1103	-0.7345
C	-2.7334	0.0777	-0.3126
O	-1.7228	0.4167	1.8921
C	-0.8226	0.9867	-1.8422
C	0.0310	2.0734	-2.1884
C	0.9599	1.9791	-3.2111
C	1.0991	0.7986	-3.9520
C	0.2641	-0.2765	-3.6432
C	-0.6748	-0.1921	-2.6212
H	-2.1776	-2.5931	0.7230
H	-1.3961	-2.6501	-0.8675
H	-0.2126	-3.5303	1.8188
H	-0.5625	-4.5423	0.4069
H	1.9081	-3.9205	0.6651
H	1.2156	-3.4395	-0.9002
H	3.5202	-2.0322	1.0821
H	3.2547	-1.2896	-0.5178
H	3.4100	0.7437	0.7957
H	2.7913	-0.0734	2.2455
H	0.7141	0.9698	1.5749
H	1.1769	0.9631	-0.1337
H	-1.8291	2.0954	-0.2920
H	-3.6849	0.4692	0.0501
H	-2.8719	-0.7529	-1.0068
H	-0.0557	3.0018	-1.6261
H	1.5882	2.8380	-3.4388
H	1.8296	0.7258	-4.7525
H	0.3403	-1.2001	-4.2141
H	-1.3122	-1.0499	-2.4284

II’

E(r^{2}scan-3c) = -806.26331513 (conv)
Lowest Freq. = 34.17 cm^{-1}

C	-1.6191	-2.2510	0.2468
C	-0.8615	-3.4821	0.7264
C	0.5472	-3.4951	0.1472
N	1.1613	-2.1887	0.3678
C	0.4717	-1.0877	0.6203
N	-0.8496	-1.0325	0.5756
C	2.6150	-1.9798	0.4473
C	2.7358	-0.4527	0.3853
C	1.3893	0.0512	0.9338
C	-1.5805	0.1886	0.9504
C	-1.3839	1.4955	-0.8254
C	-2.4664	0.7093	-0.1312
O	-1.3494	0.7119	2.0370
C	-0.5512	1.0137	-1.8528
C	-0.7644	-0.2094	-2.5664
C	0.1079	-0.6458	-3.5538
C	1.2439	0.0895	-3.9079
C	1.4678	1.3055	-3.2420
C	0.6110	1.7542	-2.2546
H	-2.5913	-2.1731	0.7426
H	-1.7842	-2.3008	-0.8362
H	-0.8131	-3.4835	1.8216
H	-1.3967	-4.3814	0.4091
H	1.1696	-4.2516	0.6381
H	0.5343	-3.7095	-0.9301
H	2.9915	-2.3996	1.3907
H	3.1086	-2.4891	-0.3866
H	2.8482	-0.1339	-0.6562
H	3.5872	-0.0824	0.9592
H	1.4079	0.1931	2.0209
H	1.0347	0.9793	0.4791
H	-1.0811	2.4103	-0.3205
H	-3.2601	1.3334	0.2900
H	-2.9111	-0.0728	-0.7541
H	-1.6432	-0.8089	-2.3479
H	-0.1063	-1.5837	-4.0646
H	1.9233	-0.2616	-4.6787
H	2.3368	1.9088	-3.5001
H	0.8197	2.6990	-1.7541

TS3aa’

E(r^{2}scan-3c) = - 806.25264971 (conv)
Lowest Freq. = -108.87 cm^{-1}

C	-1.7342	-2.2852	0.6722
C	-0.7274	-3.4219	0.6214
C	0.4480	-3.0359	-0.2699
N	0.9550	-1.7282	0.1486
C	0.2275	-0.8399	0.8303
N	-1.0650	-1.0575	1.1525
C	2.3901	-1.4168	0.1710
C	2.4240	0.0184	0.7115
C	1.1434	0.1145	1.5509
C	-1.9168	0.1484	1.1547
C	-0.7481	1.1327	-0.7276
C	-2.0978	0.7549	-0.2231
O	-2.3168	0.6110	2.1986
C	-0.1365	0.6230	-1.8947
C	-0.5921	-0.5250	-2.6106
C	0.0643	-0.9947	-3.7397
C	1.2183	-0.3740	-4.2268
C	1.6885	0.7588	-3.5467
C	1.0381	1.2447	-2.4266
H	-2.5505	-2.5077	1.3687
H	-2.1693	-2.1252	-0.3246

H	-0.3695	-3.6496	1.6329
H	-1.2137	-4.3179	0.2245
H	1.2670	-3.7573	-0.1698
H	0.1506	-3.0047	-1.3251
H	2.9045	-2.1248	0.8379
H	2.8145	-1.5130	-0.8349
H	2.3791	0.7283	-0.1188
H	3.3259	0.2148	1.2948
H	1.3058	-0.2930	2.5618
H	0.7476	1.1245	1.6497
H	-0.3138	2.0279	-0.2869
H	-2.7748	1.6095	-0.1014
H	-2.5964	0.0266	-0.8764
H	-1.4695	-1.0624	-2.2598
H	-0.3277	-1.8751	-4.2468
H	1.7307	-0.7529	-5.1060
H	2.5771	1.2744	-3.9071
H	1.4199	2.1371	-1.9330

3aa’

E(r^{2}scan-3c) = -806.32512308 (conv)
Lowest Freq. = 44.97 cm^{-1}

C	-0.8639	-2.0314	2.0204
C	-0.0660	-3.1926	1.3682
C	0.1423	-2.9017	-0.1239
N	0.7139	-1.5747	-0.3909
C	-0.0487	-0.4662	0.2353
N	-1.1596	-1.0113	1.0195
C	2.1360	-1.4254	-0.0224
C	2.1601	-0.6203	1.2815
C	0.9811	0.3279	1.0732
C	-2.3571	-0.9953	0.3549
C	-0.8118	0.4678	-0.7703
C	-2.1792	-0.2001	-0.9232
O	-3.3833	-1.5547	0.7258
C	-0.0488	0.7739	-2.0286
C	0.5678	2.0204	-2.1811
C	1.3139	2.3204	-3.3192
C	1.4537	1.3733	-4.3295
C	0.8381	0.1298	-4.1936
C	0.0948	-0.1675	-3.0551
H	-0.3025	-1.5754	2.8432
H	-1.8230	-2.3745	2.4172
H	0.9062	-3.3078	1.8597
H	-0.6016	-4.1419	1.4860
H	0.8017	-3.6531	-0.5718
H	-0.8180	-2.9839	-0.6515
H	2.6165	-2.4063	0.0589
H	2.6607	-0.8620	-0.8101
H	3.1063	-0.0902	1.4318
H	1.9953	-1.2755	2.1439
H	0.5274	0.7031	1.9954
H	1.3070	1.1945	0.4847
H	-0.9621	1.4107	-0.2284
H	-2.9991	0.5091	-1.0663
H	-2.2116	-0.9150	-1.7551
H	0.4569	2.7678	-1.3983
H	1.7817	3.2966	-3.4166
H	2.0324	1.6030	-5.2202
H	0.9373	-0.6141	-4.9800
H	-0.3746	-1.1421	-2.9659

TS1

E(r^{2}scan-3c) = -806.24554885 (conv)
Lowest Freq. = -82.05 cm^{-1}

C	-1.6367	-2.5073	0.4912
C	-1.0708	-3.0862	1.7883
C	0.2670	-3.7654	1.5150
N	1.0793	-2.8975	0.6699
C	0.5502	-1.8398	0.0015
N	-0.7071	-1.5818	-0.1650
C	2.5376	-2.8536	0.7586
C	2.9141	-1.8876	-0.3718
C	1.6847	-0.9737	-0.4932
C	-1.3465	0.5621	0.3838
C	-1.0115	1.2567	-0.8986
C	-2.4484	0.6985	-0.5687
O	-1.1189	0.6584	1.5747
C	-0.6857	2.6993	-0.8928
C	-1.3690	3.6214	-0.0866
C	-1.0157	4.9669	-0.0907
C	0.0296	5.4220	-0.8930
C	0.7202	4.5144	-1.6932
C	0.3670	3.1683	-1.6916
H	-2.5695	-1.9657	0.6954
H	-1.8901	-3.3273	-0.2007
H	-0.9295	-2.2706	2.5084
H	-1.7675	-3.8079	2.2277
H	0.8105	-3.9490	2.4511
H	0.1194	-4.7377	1.0221
H	2.8480	-2.4731	1.7452
H	2.9657	-3.8548	0.6279
H	3.0537	-2.4481	-1.3024
H	3.8352	-1.3408	-0.1577
H	1.7637	-0.1007	0.1688
H	1.5117	-0.6067	-1.5073
H	-0.5061	0.6740	-1.6614
H	-3.2277	1.4135	-0.3036
H	-2.7604	-0.1286	-1.1973
H	-2.1829	3.2882	0.5521
H	-1.5602	5.6654	0.5396
H	0.3022	6.4737	-0.8943
H	1.5359	4.8559	-2.3255
H	0.9077	2.4661	-2.3227

I

E(r^{2}scan-3c) = -806.25990826 (conv)
Lowest Freq. = 36.65 cm^{-1}

C	-1.7371	-2.2323	0.6952
C	-1.0968	-3.3292	1.5369
C	0.1993	-3.8070	0.8938
N	1.0169	-2.6439	0.5602
C	0.5111	-1.4171	0.4273
N	-0.7738	-1.1516	0.4237
C	2.4847	-2.6521	0.6061
C	2.8465	-1.2805	0.0249
C	1.6162	-0.4095	0.3297
C	-1.2495	0.2992	0.5051
C	-1.1022	0.9840	-0.9295
C	-2.4508	0.5279	-0.3687
O	-1.0153	0.8904	1.6190

C	-0.6831	2.3835	-1.0204
C	-1.1765	3.3869	-0.1669
C	-0.7098	4.6952	-0.2591
C	0.2632	5.0432	-1.1952
C	0.7678	4.0579	-2.0444
C	0.3029	2.7501	-1.9553
H	-2.5859	-1.7848	1.2213
H	-2.1048	-2.6406	-0.2563
H	-0.8869	-2.9419	2.5411
H	-1.7907	-4.1691	1.6353
H	0.7698	-4.4394	1.5834
H	-0.0002	-4.3911	-0.0151
H	2.8188	-2.7683	1.6475
H	2.8766	-3.4894	0.0188
H	2.9833	-1.3625	-1.0581
H	3.7635	-0.8787	0.4601
H	1.6850	0.0988	1.2993
H	1.4119	0.3562	-0.4206
H	-0.7151	0.3505	-1.7261
H	-3.1299	1.3021	-0.0180
H	-2.9280	-0.2882	-0.9098
H	-1.9195	3.1310	0.5808
H	-1.1098	5.4523	0.4117
H	0.6218	6.0667	-1.2636
H	1.5245	4.3105	-2.7835
H	0.7011	1.9920	-2.6274

TS2

E(r^{2}scan-3c) = -806.25748163 (conv)
Lowest Freq. = -294.78 cm^{-1}

C	-1.7063	-2.2717	0.6770
C	-1.0369	-3.4455	1.3822
C	0.2873	-3.7907	0.7130
N	1.0573	-2.5644	0.5268
C	0.5170	-1.3497	0.5505
N	-0.7791	-1.1274	0.5994
C	2.5260	-2.5298	0.4876
C	2.8150	-1.0919	0.0393
C	1.5818	-0.3000	0.5079
C	-1.2969	0.2679	0.7704
C	-1.3194	0.9932	-0.9423
C	-2.4761	0.5668	-0.0669
O	-0.8772	0.9263	1.7526
C	-0.8060	2.3232	-1.0473
C	-1.1710	3.3808	-0.1754
C	-0.6048	4.6449	-0.2980
C	0.3502	4.9208	-1.2777
C	0.7335	3.8889	-2.1424
C	0.1750	2.6254	-2.0302
H	-2.5911	-1.9448	1.2307
H	-2.0220	-2.5562	-0.3363
H	-0.8628	-3.1860	2.4331
H	-1.7002	-4.3146	1.3538
H	0.8754	-4.4737	1.3365
H	0.1282	-4.2733	-0.2610
H	2.9244	-2.7481	1.4889
H	2.8998	-3.2865	-0.2101
H	2.8874	-1.0534	-1.0523
H	3.7454	-0.7085	0.4624
H	1.6991	0.1085	1.5181
H	1.2944	0.5293	-0.1435
H	-1.0139	0.2833	-1.7066
H	-3.1075	1.3620	0.3316
H	-3.0631	-0.2612	-0.4685

H	-1.8972	3.1990	0.6099
H	-0.9142	5.4305	0.3889
H	0.7868	5.9115	-1.3665
H	1.4756	4.0771	-2.9157
H	0.4851	1.8390	-2.7169

II

E(r^{2}scan-3c) = -806.26175522 (conv)
Lowest Freq. = 23.11 cm^{-1}

C	-1.6899	-2.2227	0.3986
C	-1.1170	-3.4949	1.0083
C	0.3132	-3.7148	0.5404
N	1.0548	-2.4640	0.6725
C	0.4934	-1.2726	0.8171
N	-0.8162	-1.0739	0.7245
C	2.5211	-2.4203	0.7799
C	2.8268	-0.9331	0.5797
C	1.5395	-0.2218	1.0340
C	-1.4095	0.2393	0.9962
C	-1.6327	1.0465	-1.1297
C	-2.4883	0.6534	0.0418
O	-0.9012	0.9543	1.8517
C	-0.8962	2.2434	-1.2170
C	-0.9729	3.3097	-0.2626
C	-0.2590	4.4884	-0.4287
C	0.5843	4.6914	-1.5260
C	0.6926	3.6560	-2.4682
C	-0.0100	2.4748	-2.3216
H	-2.6795	-2.0205	0.8136
H	-1.7787	-2.3139	-0.6920
H	-1.1448	-3.4188	2.1015
H	-1.7354	-4.3471	0.7131
H	0.8161	-4.4753	1.1486
H	0.3472	-4.0412	-0.5077
H	2.8221	-2.7883	1.7708
H	2.9664	-3.0654	0.0157
H	3.0049	-0.7319	-0.4812
H	3.7021	-0.6118	1.1469
H	1.5605	0.0431	2.0966
H	1.3125	0.6901	0.4768
H	-1.4122	0.2622	-1.8519
H	-3.0545	1.4783	0.4876
H	-3.1840	-0.1487	-0.2185
H	-1.5993	3.1927	0.6159
H	-0.3574	5.2698	0.3238
H	1.1404	5.6168	-1.6435
H	1.3450	3.7791	-3.3315
H	0.0948	1.6927	-3.0727

TS3aa

E(r^{2}scan-3c) = -806.25336069 (conv)
Lowest Freq. = -116.79 cm^{-1}

C	-1.9086	-2.3261	1.0211
C	-0.9571	-3.5106	0.9960
C	0.1733	-3.2353	0.0105
N	0.7500	-1.9313	0.3068
C	0.0730	-0.9303	0.8800
N	-1.1699	-1.1030	1.4044
C	2.1705	-1.6231	0.1189
C	2.2436	-0.1211	0.4222

C	1.0289	0.1377	1.3258
C	-1.9727	0.1381	1.3373
C	-1.1533	0.5468	-0.9333
C	-2.3812	0.5054	-0.0818
O	-2.1368	0.8081	2.3263
C	-0.3864	1.7246	-1.0999
C	-0.5134	2.8749	-0.2625
C	0.2705	4.0059	-0.4539
C	1.2276	4.0667	-1.4694
C	1.3797	2.9476	-2.3007
C	0.6056	1.8146	-2.1245
H	-2.7112	-2.4697	1.7521
H	-2.3647	-2.2060	0.0266
H	-0.5460	-3.6863	1.9971
H	-1.5054	-4.4069	0.6907
H	0.9667	-3.9856	0.0994
H	-0.1979	-3.2566	-1.0255
H	2.7671	-2.2230	0.8217
H	2.4835	-1.8720	-0.9017
H	2.1359	0.4531	-0.5030
H	3.1865	0.1563	0.8981
H	1.2652	-0.0377	2.3858
H	0.6309	1.1518	1.2290
H	-1.0337	-0.2389	-1.6758
H	-2.9139	1.4617	-0.0192
H	-3.0940	-0.2451	-0.4462
H	-1.2416	2.8730	0.5453
H	0.1329	4.8589	0.2084
H	1.8363	4.9548	-1.6118
H	2.1164	2.9667	-3.1020
H	0.7426	0.9627	-2.7895

3aa

E(r^{2}scan-3c) = -806.32948763 (conv)
Lowest Freq. = 49.89 cm^{-1}

C	-1.1687	-2.4843	1.6049
C	-0.5809	-3.4307	0.5315
C	-0.0298	-2.6261	-0.6640
N	0.6273	-1.3785	-0.2778
C	-0.2687	-0.4527	0.4259
N	-1.4009	-1.1687	1.0380
C	1.8811	-1.5090	0.4771
C	2.0577	-0.1124	1.0639
C	0.6270	0.2310	1.4860
C	-2.5972	-0.5418	0.9249
C	-1.0268	0.5106	-0.5481
C	-2.3487	0.7640	0.1824
O	-3.6728	-0.9448	1.3614
C	-0.2191	1.6906	-1.0168
C	-0.2627	2.9421	-0.3931
C	0.5459	3.9880	-0.8332
C	1.4129	3.8042	-1.9070
C	1.4585	2.5664	-2.5445
C	0.6475	1.5242	-2.1054
H	-0.4733	-2.3909	2.4508
H	-2.1218	-2.8497	1.9977
H	0.2089	-4.0408	0.9824
H	-1.3496	-4.1203	0.1646
H	0.6775	-3.2342	-1.2402
H	-0.8556	-2.3754	-1.3429
H	1.8191	-2.2555	1.2910
H	2.6961	-1.8083	-0.1935
H	2.4116	0.5787	0.2896
H	2.7637	-0.0908	1.8992
H	0.4112	-0.2132	2.4646
H	0.4342	1.3046	1.5570
H	-1.2541	-0.1049	-1.4292
H	-2.2716	1.5620	0.9320
H	-3.1837	1.0025	-0.4814
H	-0.9333	3.1150	0.4437
H	0.4932	4.9525	-0.3348
H	2.0408	4.6219	-2.2507
H	2.1227	2.4131	-3.3912
H	0.6819	0.5627	-2.6119

TS4

E(r^{2}scan-3c) = -806.25025101 (conv)
Lowest Freq. = -37.95 cm^{-1}

C	-0.0821	-2.5109	-0.2229
C	0.2592	-3.7398	0.6041
C	1.6358	-3.5777	1.2211
N	1.7464	-2.2392	1.8020
C	0.9203	-1.2395	1.5809
N	-0.0463	-1.3054	0.6462
C	2.7546	-1.9369	2.8337
C	2.6843	-0.4140	2.9372
C	1.2616	-0.0723	2.4601
C	-0.9924	-0.2296	0.4824
C	-0.5505	0.1855	-1.8946
C	-1.6211	-0.1199	-0.8732
O	-1.1950	0.5323	1.4043
C	-0.8603	0.6935	-3.1563
C	-2.2091	0.9230	-3.6123
C	-2.4806	1.4258	-4.8769
C	-1.4631	1.7357	-5.7872
C	-0.1351	1.5150	-5.3734
C	0.1624	1.0194	-4.1205
H	-1.0838	-2.5996	-0.6412
H	0.6304	-2.3773	-1.0446
H	-0.4946	-3.8819	1.3871
H	0.2474	-4.6216	-0.0420
H	1.8085	-4.3026	2.0244
H	2.4319	-3.6987	0.4753
H	2.4668	-2.4468	3.7628
H	3.7293	-2.3151	2.5107
H	3.4179	0.0388	2.2637
H	2.8760	-0.0602	3.9514
H	0.5424	-0.0344	3.2858
H	1.1917	0.8769	1.9270
H	0.4862	-0.0121	-1.6348
H	-2.3511	0.6960	-0.7787
H	-2.2252	-1.0257	-1.0894
H	-3.0420	0.6905	-2.9524
H	-3.5207	1.5781	-5.1648
H	-1.6879	2.1276	-6.7745
H	0.6836	1.7426	-6.0554
H	1.2041	0.8670	-3.8389

Further Mechanistic Experiments

Attempted Synthesis of 3ga from 2-methyl-substituted SCP 1g:

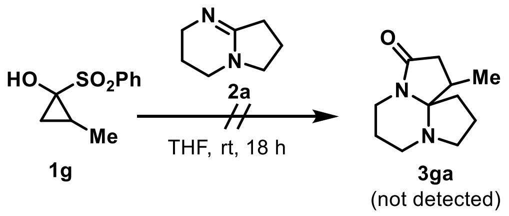

GP1 was followed, starting with **1g** (63.7 mg, 0.30 mmol, 1.00 equiv.) and **2a** (0.07 mL, 0.60 mmol, 2.00 equiv.) for 18 h. The intended product was not obtained.

Attempted Synthesis of 3ha from unsubstituted SCP 1h:

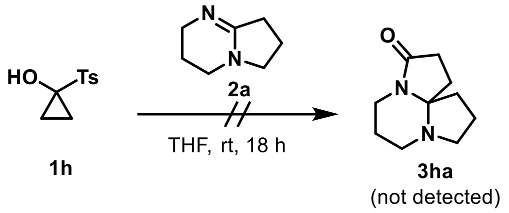

GP1 was followed, starting with **1h** (21.2 mg, 0.10 mmol, 1.00 equiv.) and **2a** (0.12 mL, 1.00 mmol, 10.0 equiv.) for 18 h. The intended product was not obtained.

7. NMR Spectra

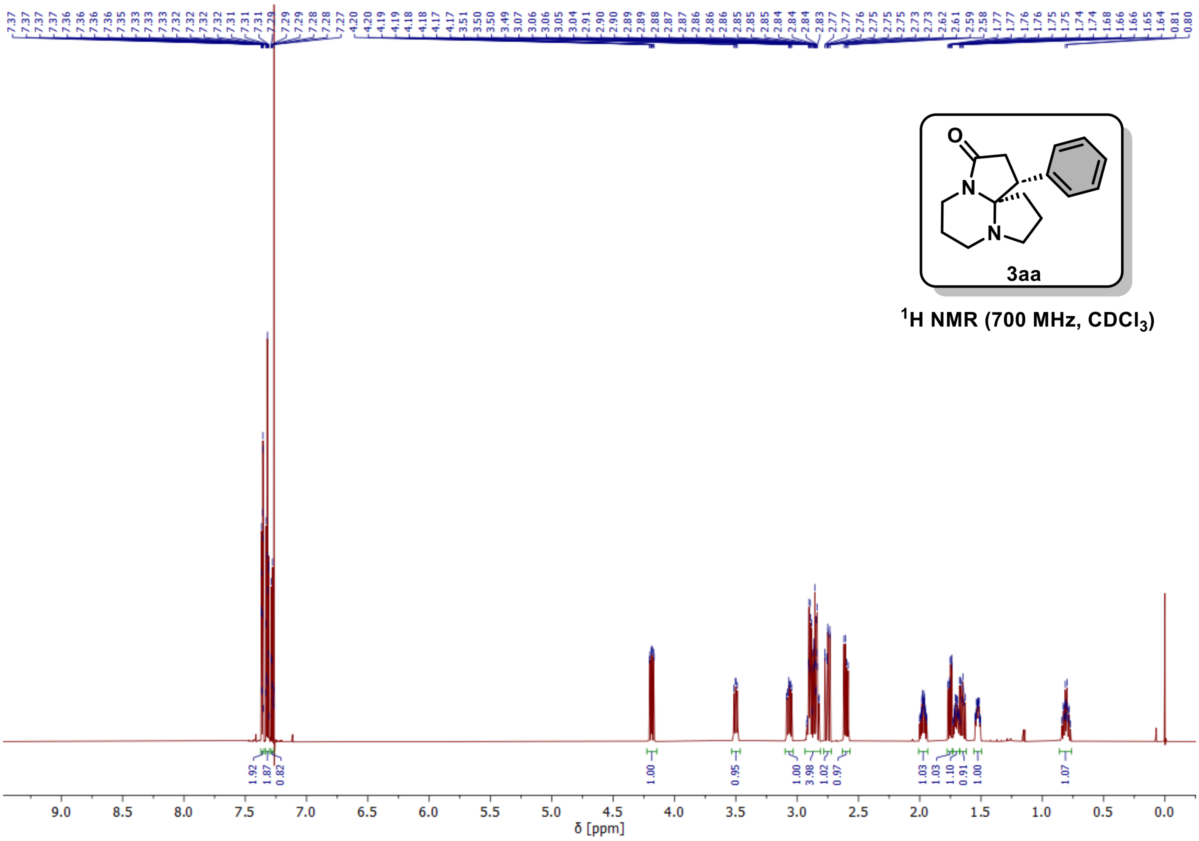

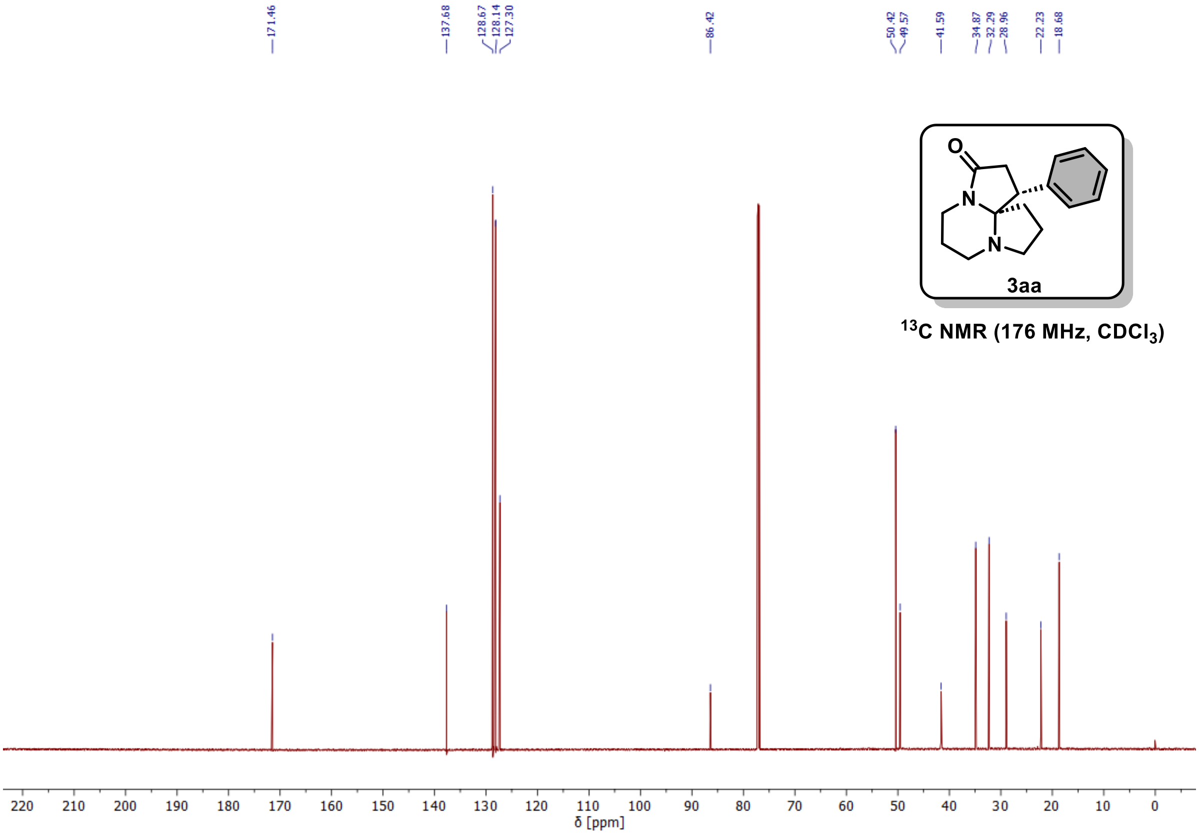

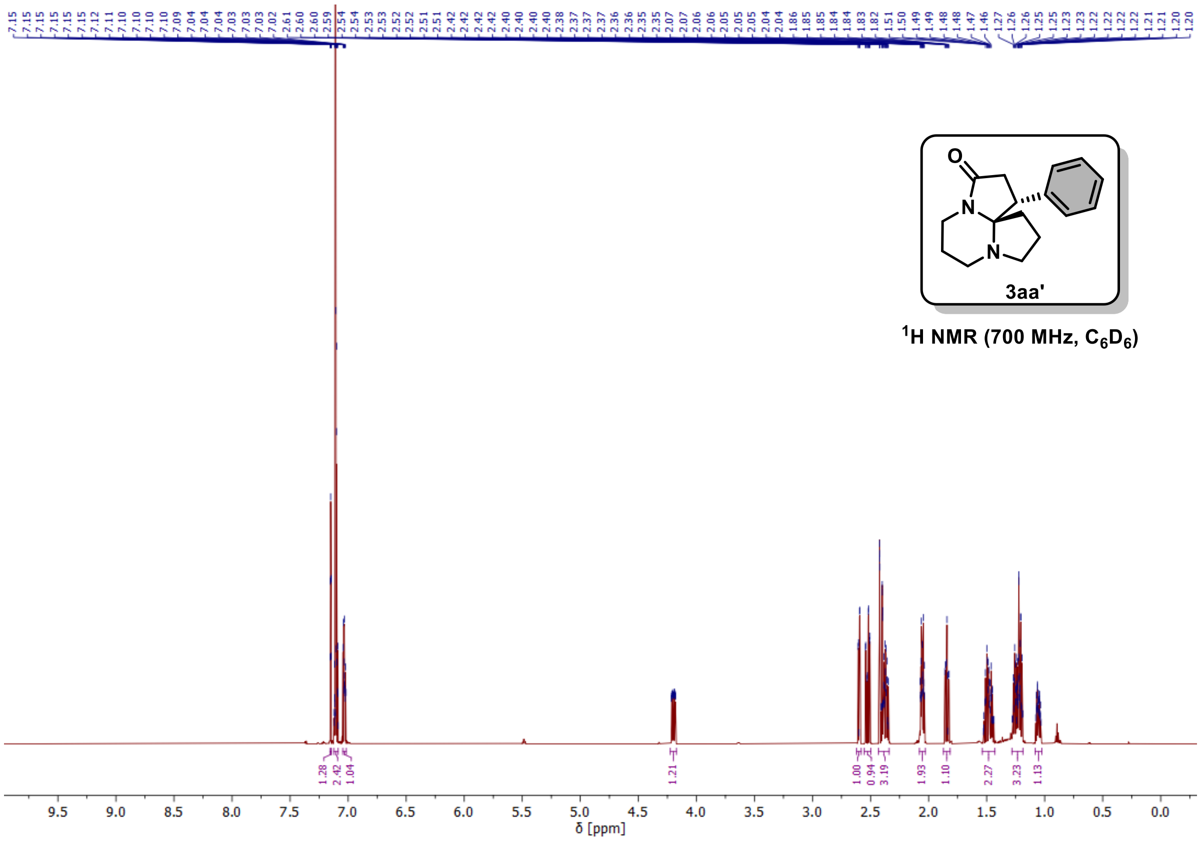
O
N
N
3aa'
1H NMR (700 MHz, C6D6)
1.28
2.42
1.04
1.21
1.00
0.94
3.19
1.93
1.10
2.27
3.23
1.13
δ [ppm]

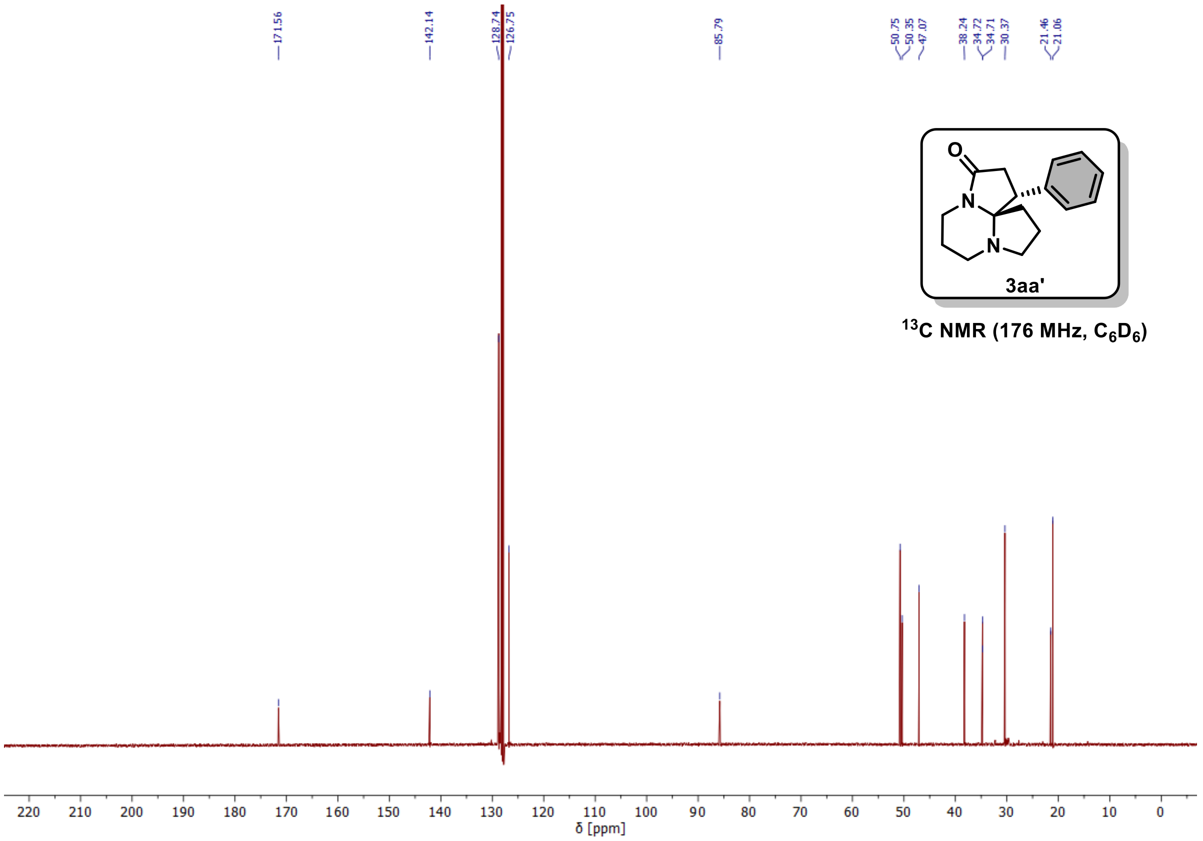
171.56
142.14
128.74
126.75
85.79
50.75
50.35
47.07
38.24
34.72
34.71
30.37
21.46
21.06
O
N
N
3aa'
13C NMR (176 MHz, C6D6)
δ [ppm]

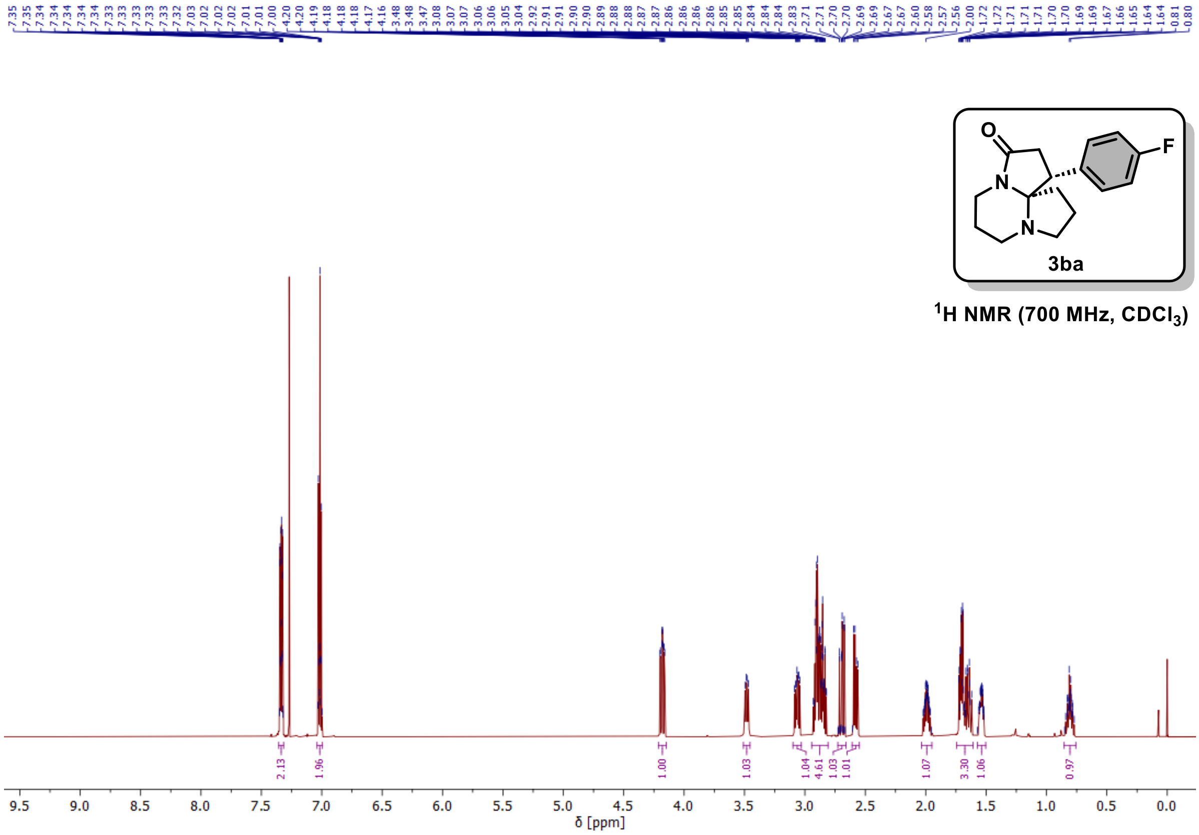
3ba
^{1}H NMR (700 MHz, CDCl$_3$)
2.13
1.96
1.00
1.03
1.04
4.61
1.03
1.01
1.07
3.30
1.06
0.97
δ [ppm]

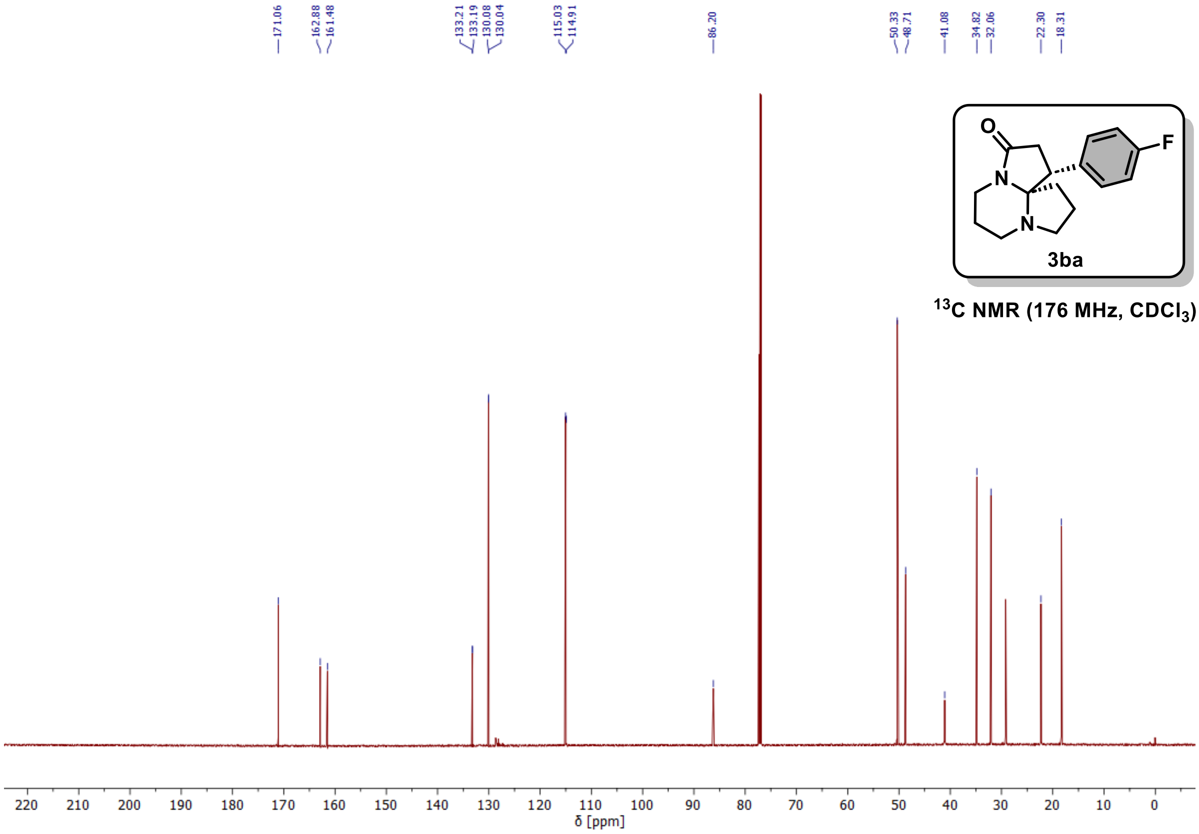
171.06
162.88
161.48
133.21
133.19
130.08
130.04
115.03
114.91
86.20
50.33
48.71
41.08
34.82
32.06
22.30
18.31
3ba
^{13}C NMR (176 MHz, CDCl$_3$)
δ [ppm]

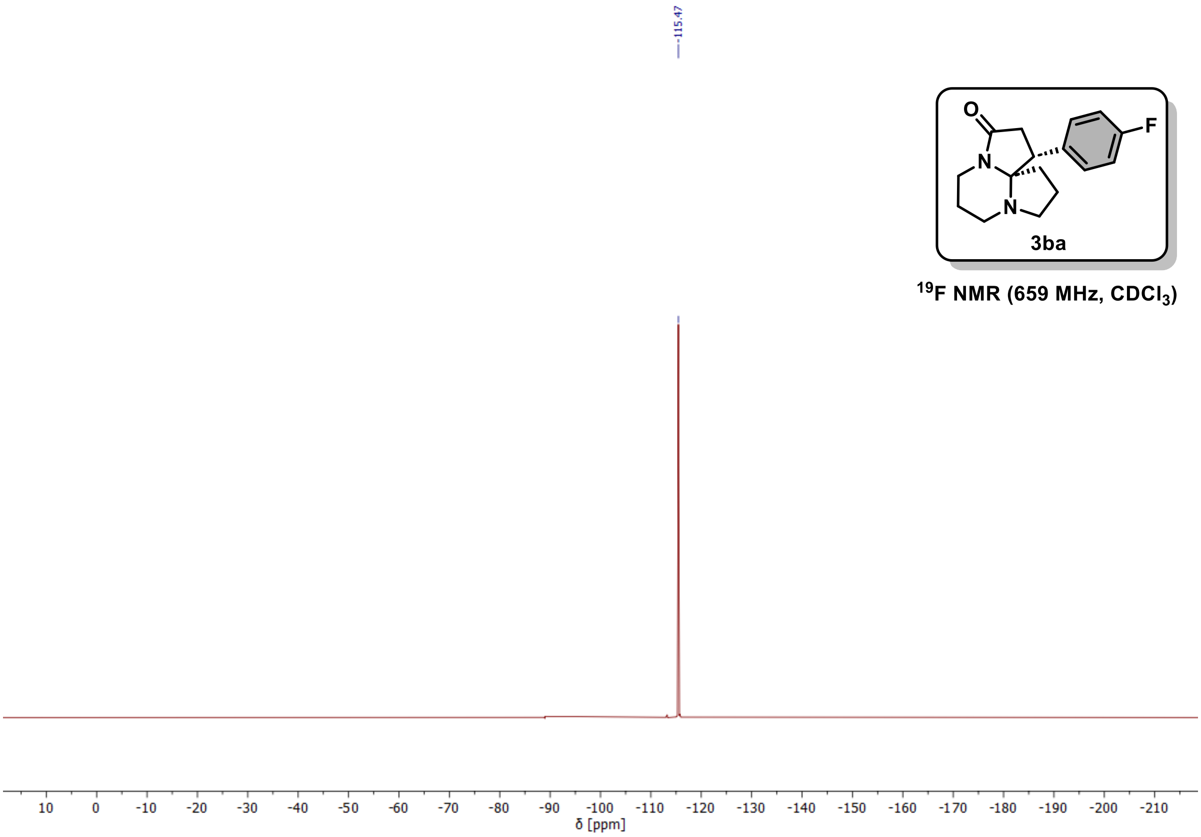
3ba
^{19}F NMR (659 MHz, $CDCl_3$)
δ [ppm]

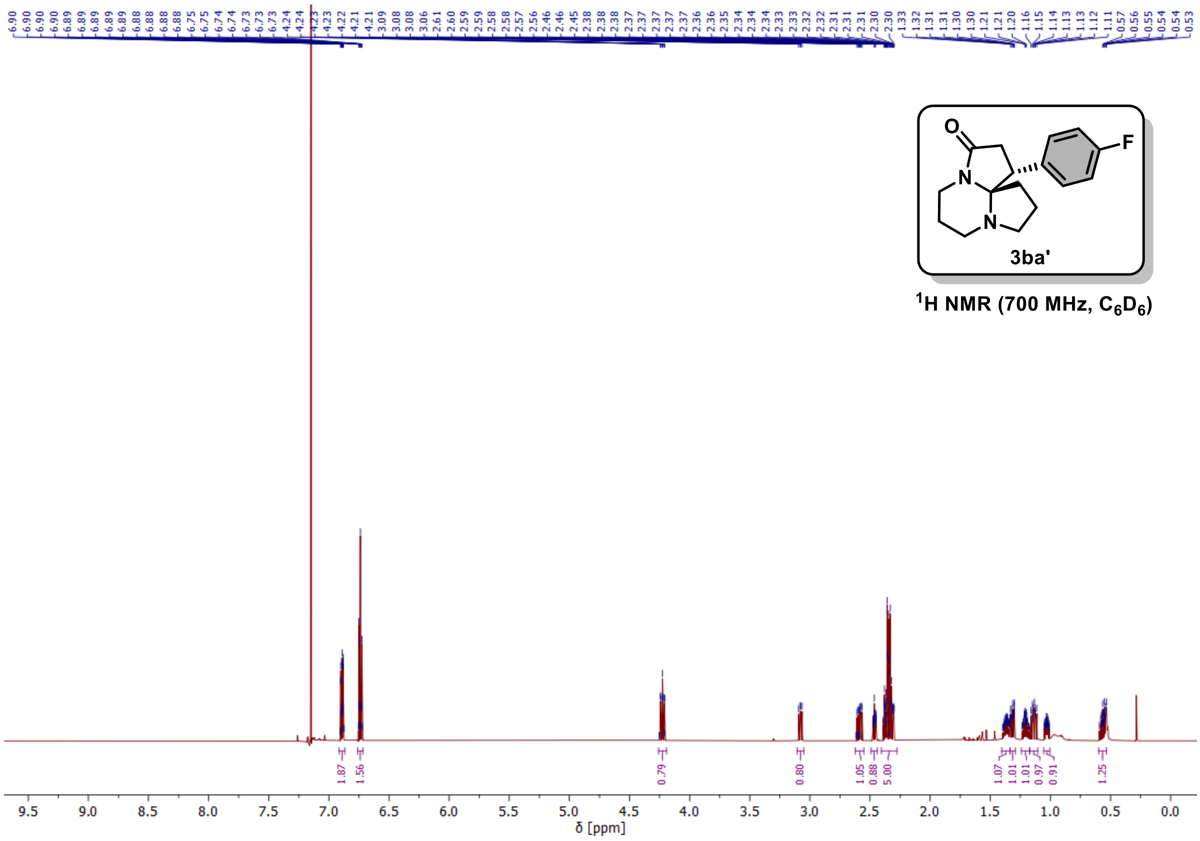
3ba'
^{1}H NMR (700 MHz, C_6D_6)
δ [ppm]

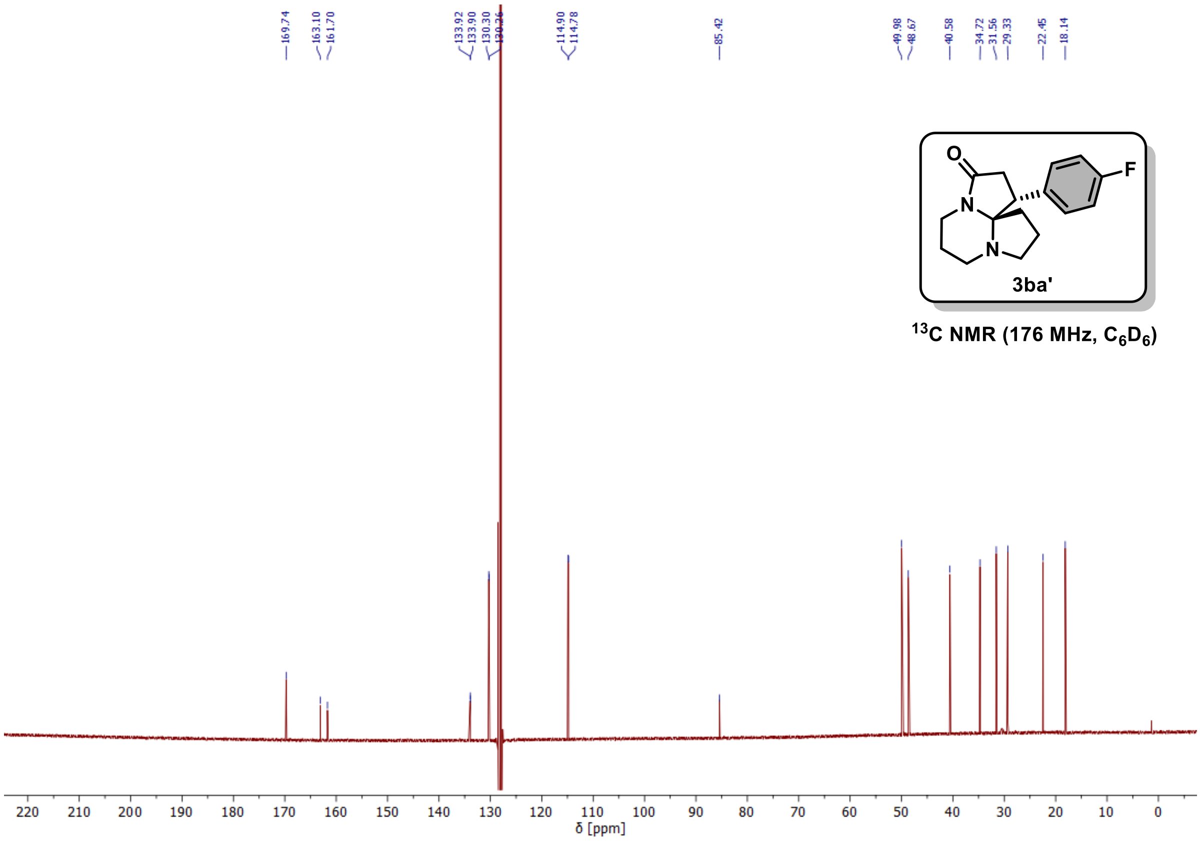

^{13}C NMR (176 MHz, C_6D_6)

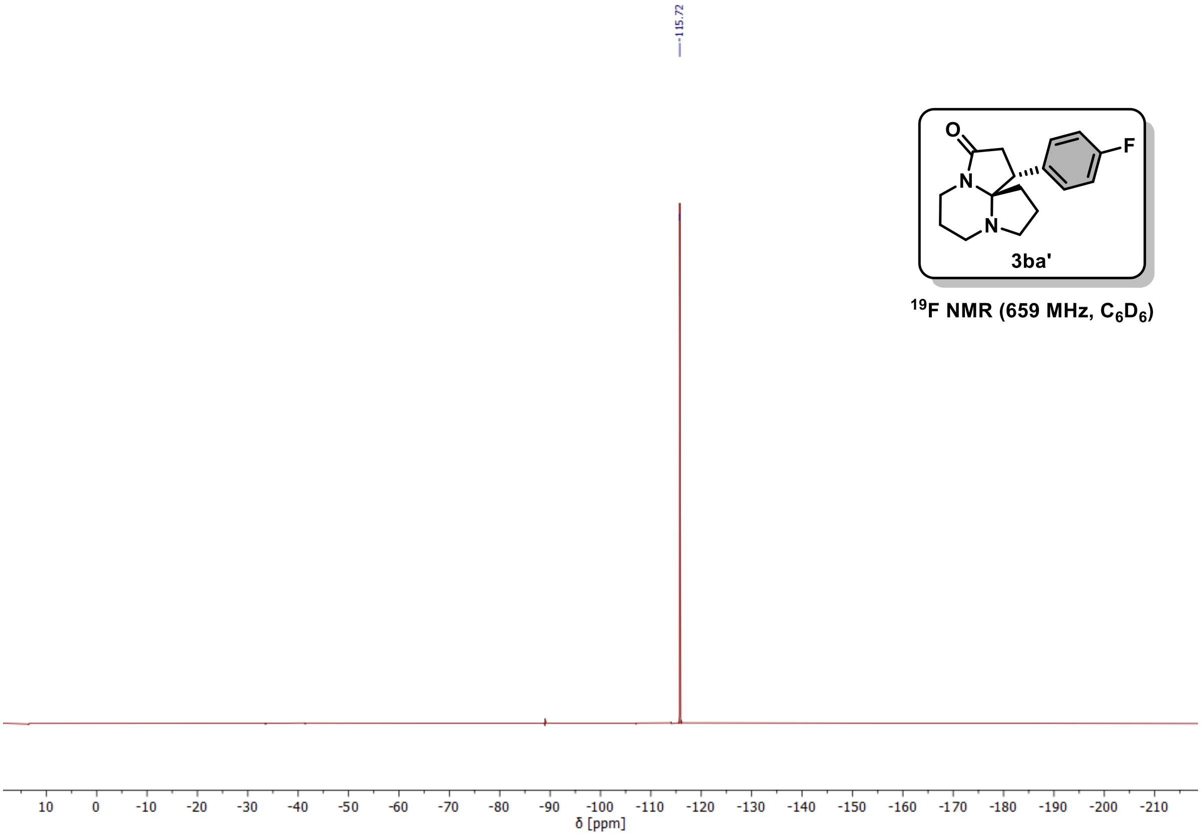

^{19}F NMR (659 MHz, C_6D_6)

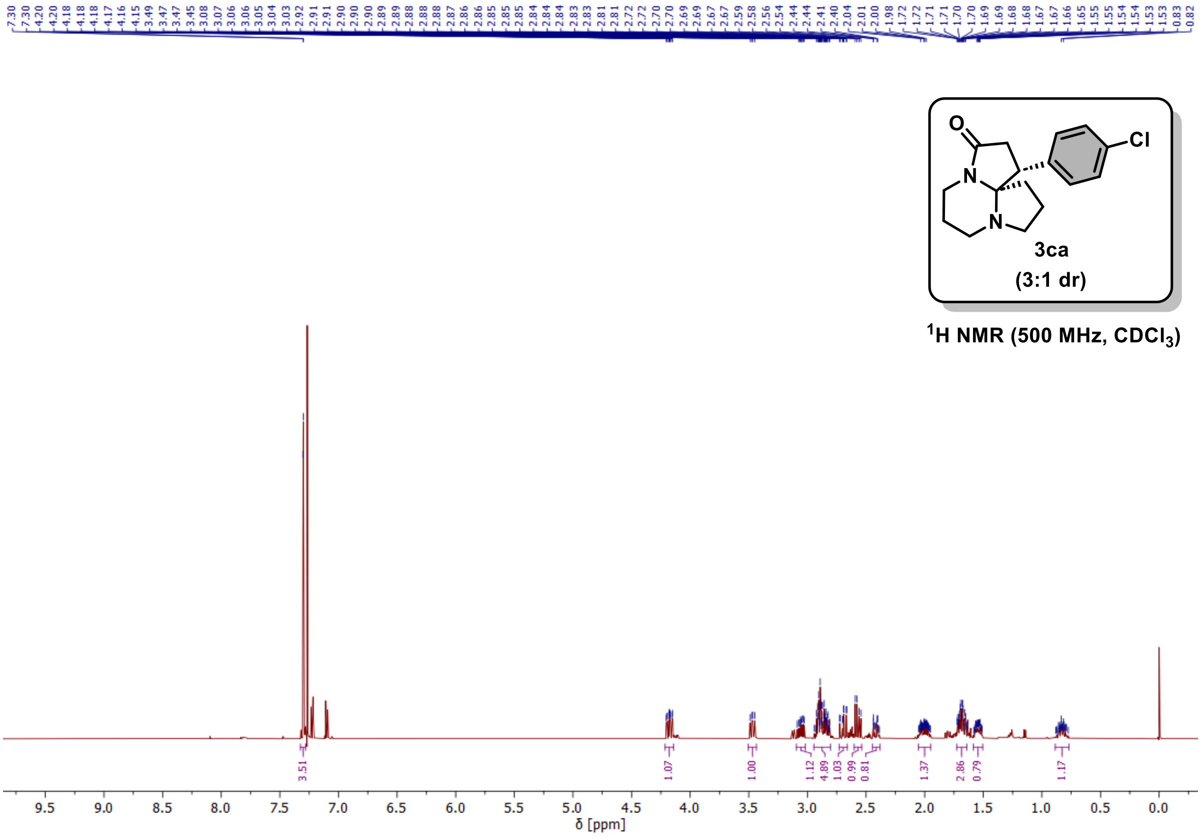
Cl
3ca
(3:1 dr)
1H NMR (500 MHz, CDCl3)
3.51
1.07
1.00
1.12
4.89
1.03
0.99
0.81
1.37
2.86
0.79
1.17
9.5 9.0 8.5 8.0 7.5 7.0 6.5 6.0 5.5 5.0 4.5 4.0 3.5 3.0 2.5 2.0 1.5 1.0 0.5 0.0
δ [ppm]

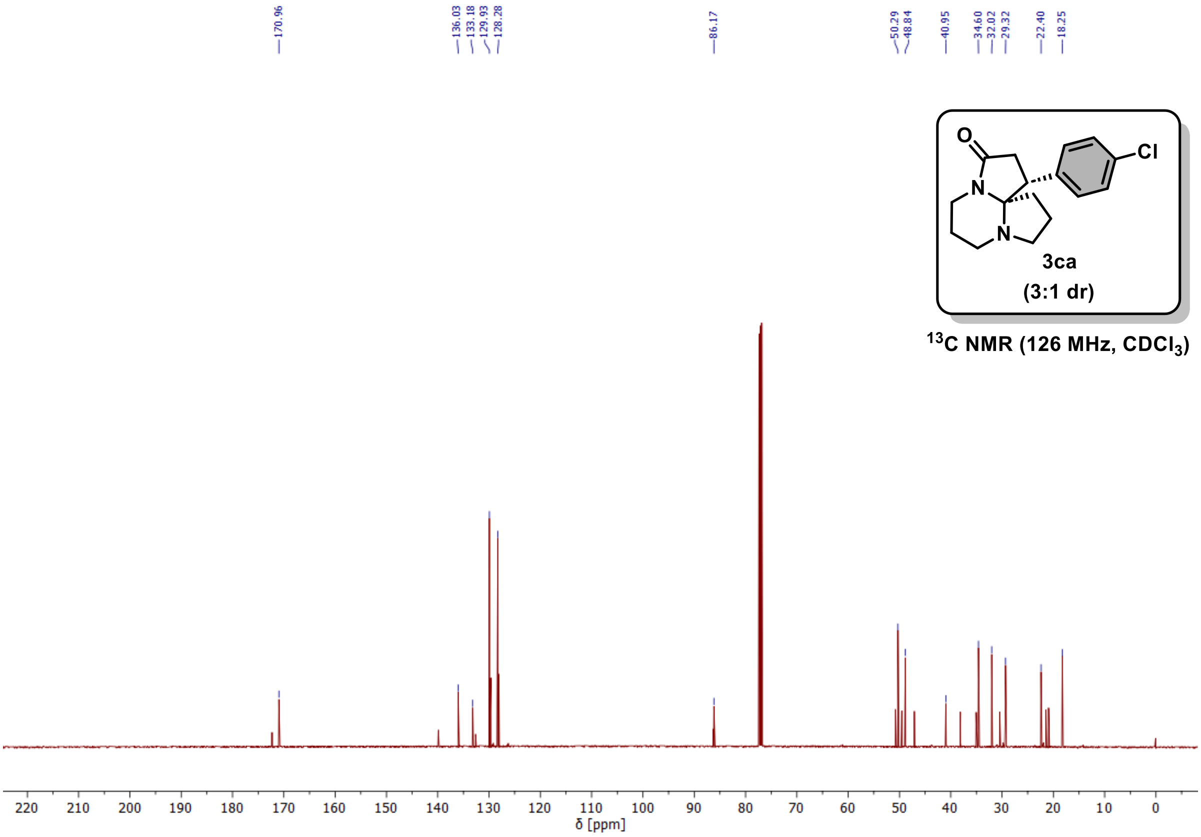
170.96
136.03
133.18
129.93
128.28
86.17
50.29
48.84
40.95
34.60
32.02
29.32
22.40
18.25
Cl
3ca
(3:1 dr)
13C NMR (126 MHz, CDCl3)
220 210 200 190 180 170 160 150 140 130 120 110 100 90 80 70 60 50 40 30 20 10 0
δ [ppm]

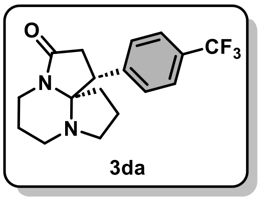

^{1}H NMR (300 MHz, C_6D_6)

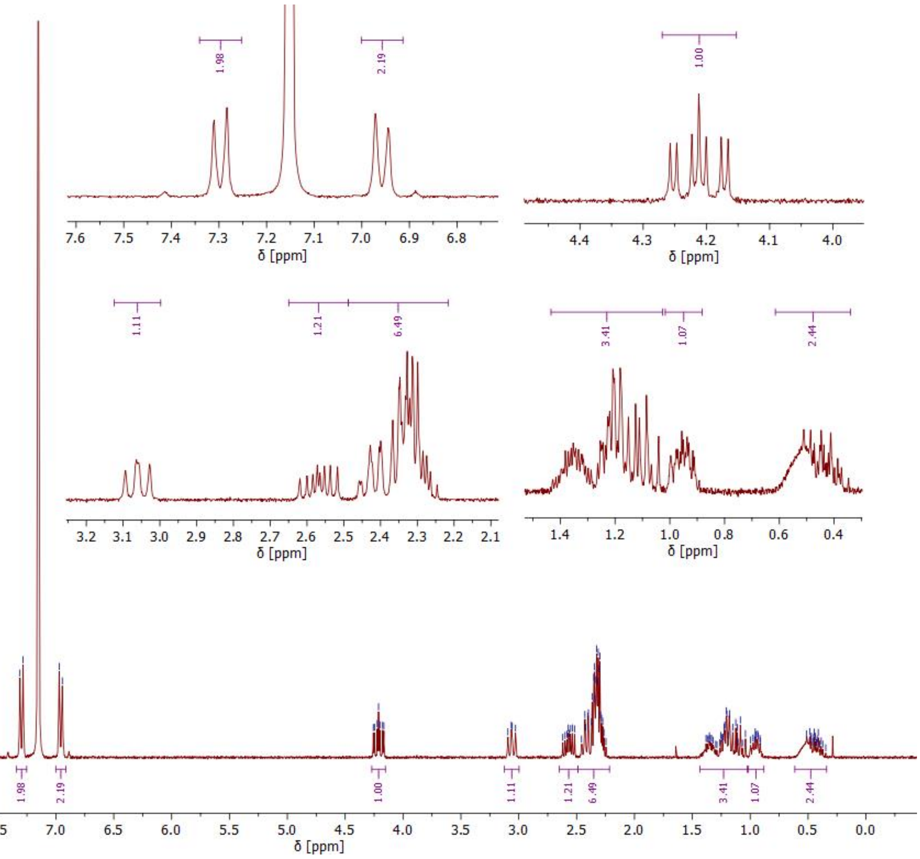

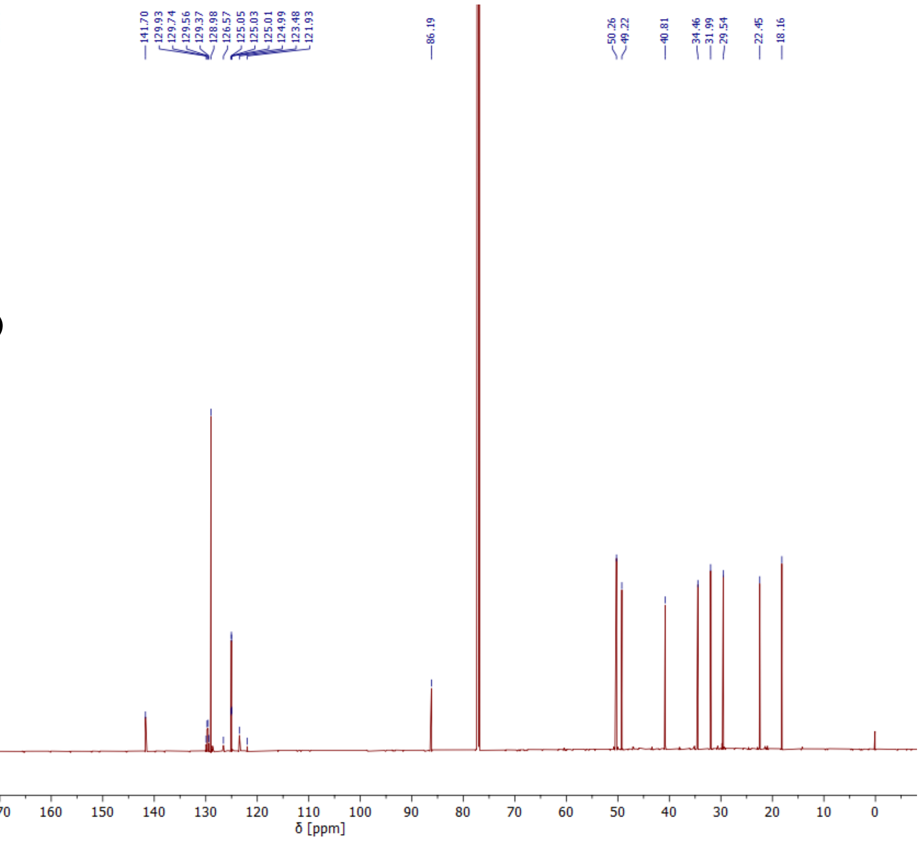

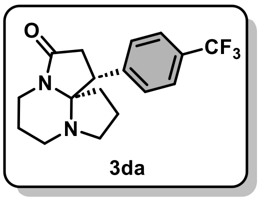

^{13}C NMR (176 MHz, $CDCl_3$)

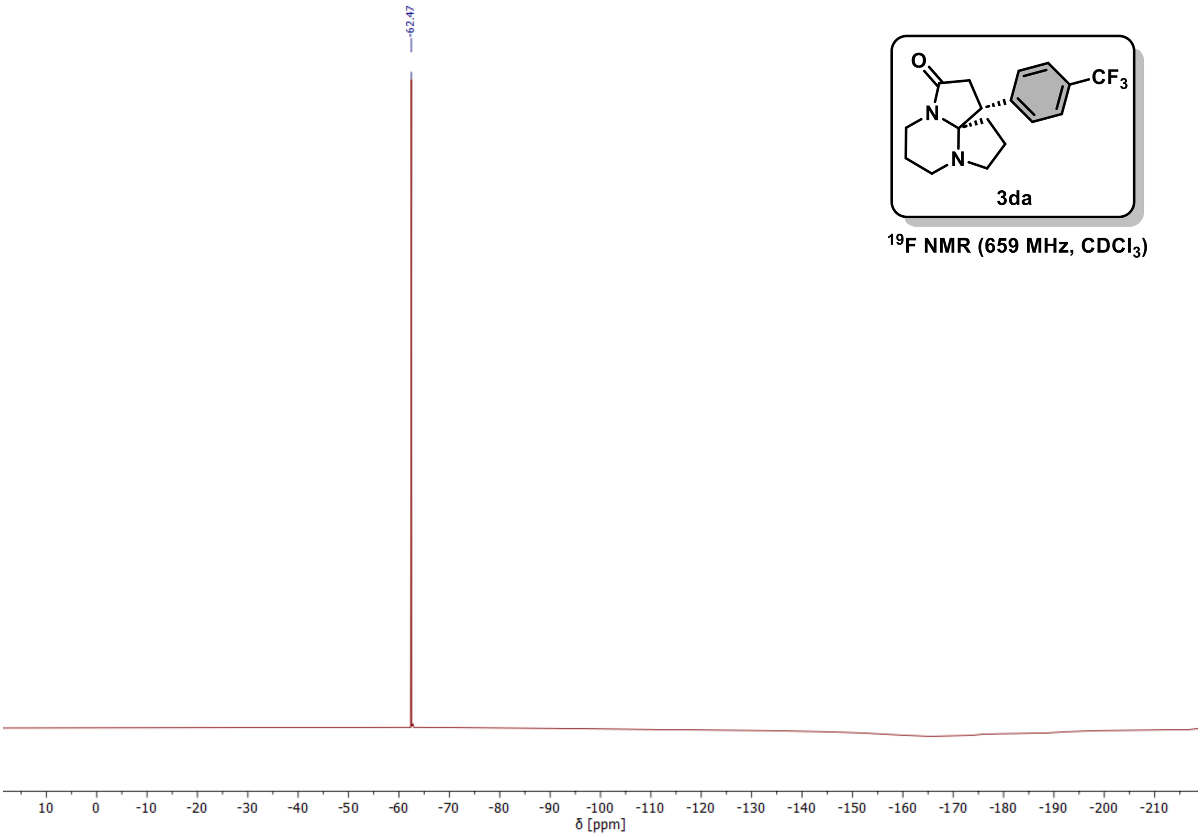
-62.47
3da
19F NMR (659 MHz, CDCl3)
δ [ppm]

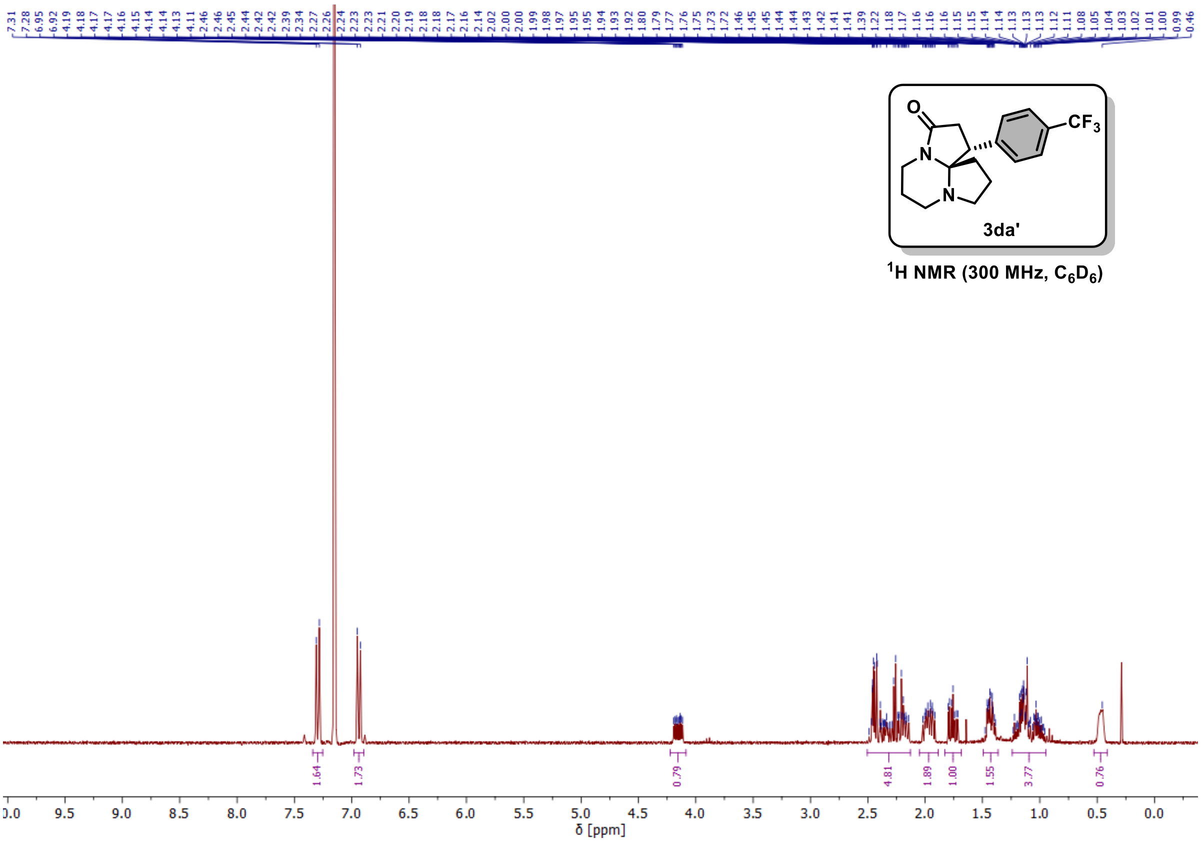
3da'
1H NMR (300 MHz, C6D6)
δ [ppm]

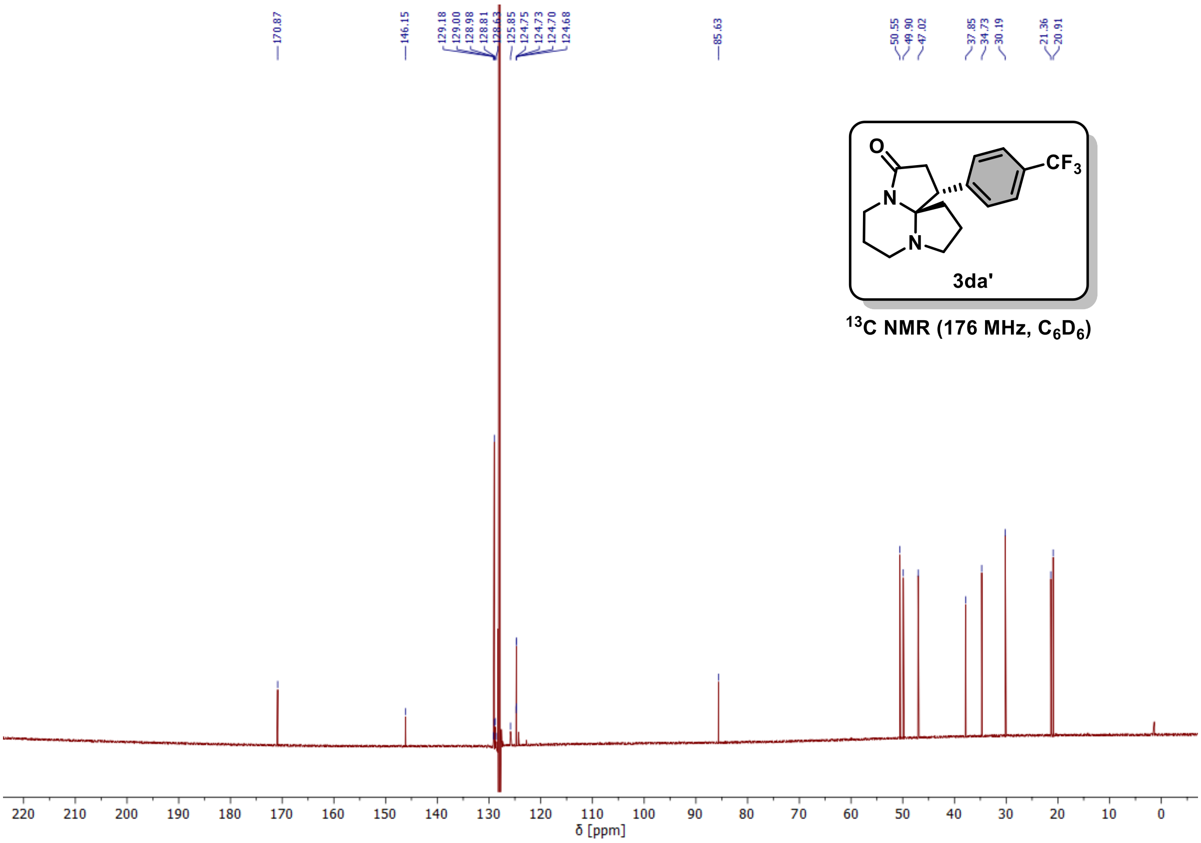
170.87
146.15
129.18
129.00
128.98
128.81
128.63
125.85
124.75
124.73
124.70
124.68
85.63
50.55
49.90
47.02
37.85
34.73
30.19
21.36
20.91
O
N
N
CF3
3da'
13C NMR (176 MHz, C6D6)
δ [ppm]

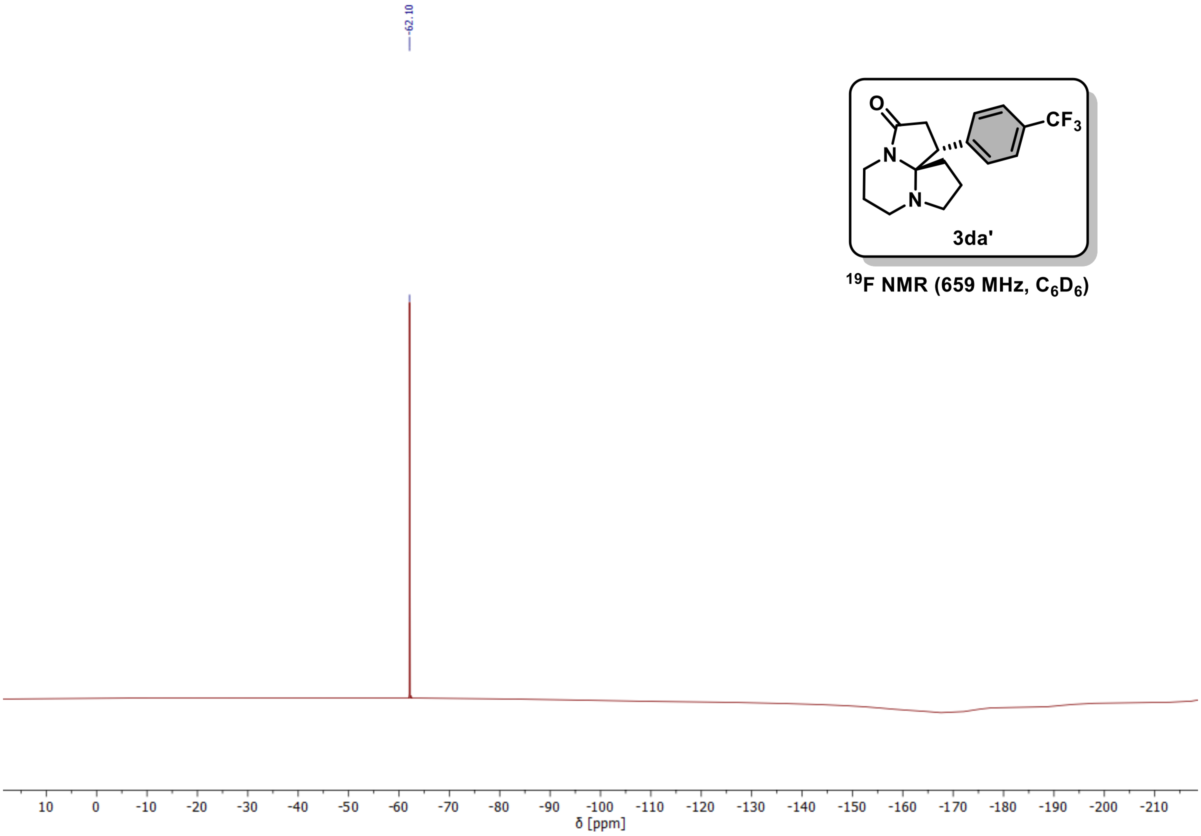
-62.10
O
N
N
CF3
3da'
19F NMR (659 MHz, C6D6)
δ [ppm]

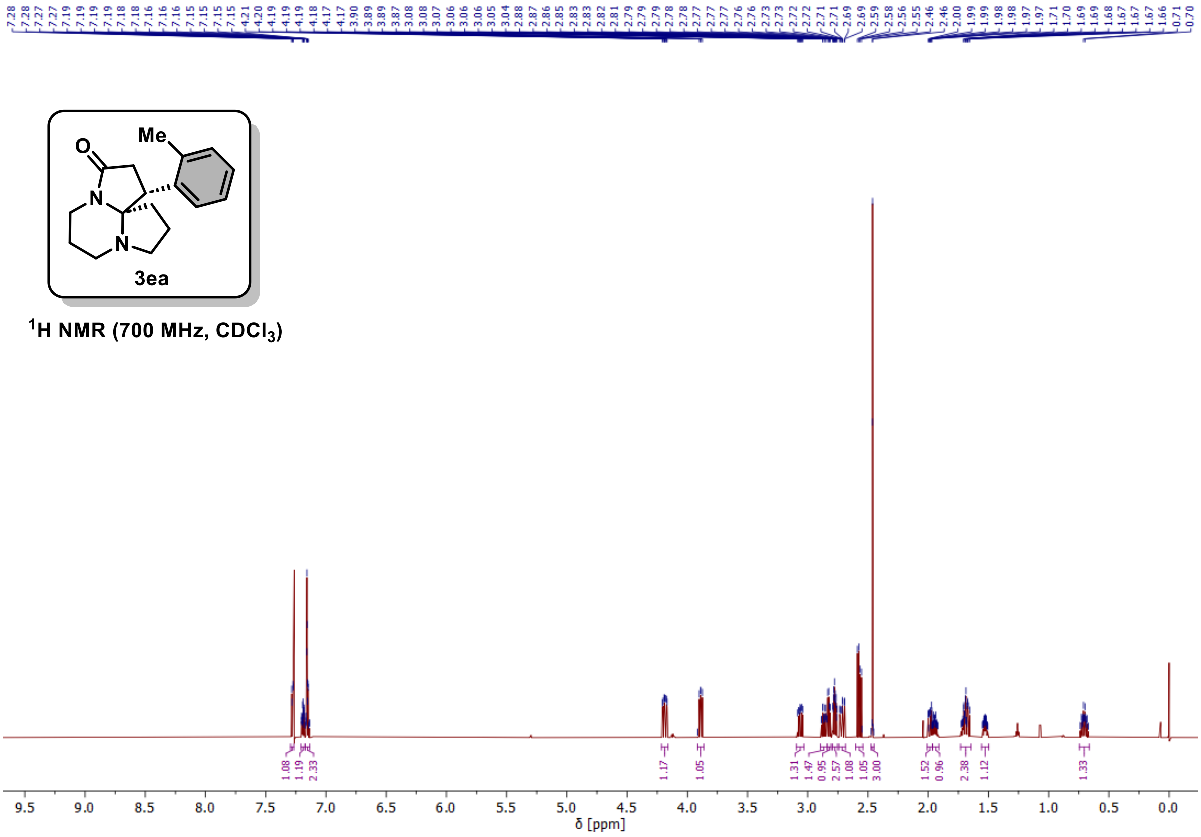
Me
O
N
N
3ea
^{1}H NMR (700 MHz, CDCl$_3$)
1.08
1.19
2.33
1.17
1.05
1.31
1.47
0.95
2.57
1.08
1.05
3.00
1.52
0.96
2.38
1.12
1.33
δ [ppm]

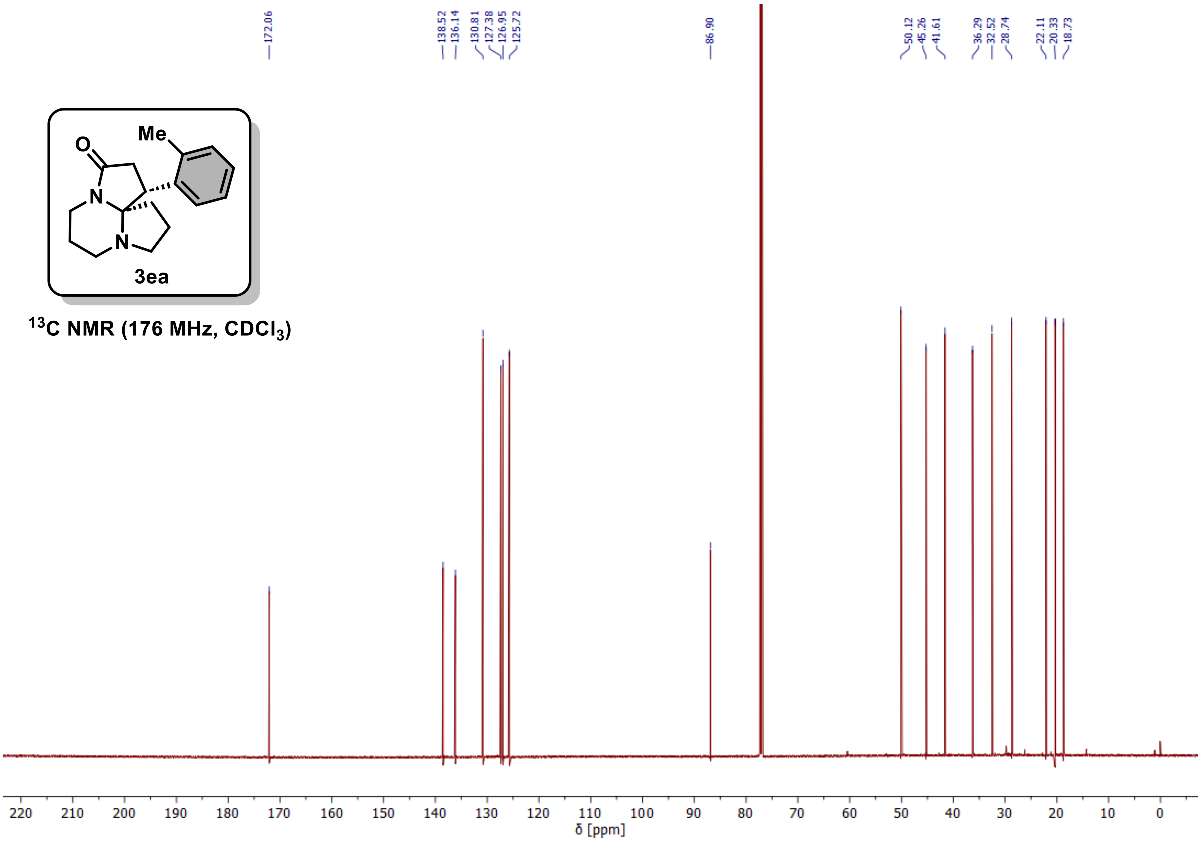
172.06
138.52
136.14
130.81
127.38
126.95
125.72
86.90
50.12
45.26
41.61
36.29
32.52
28.74
22.11
20.33
18.73
Me
O
N
N
3ea
^{13}C NMR (176 MHz, CDCl$_3$)
δ [ppm]

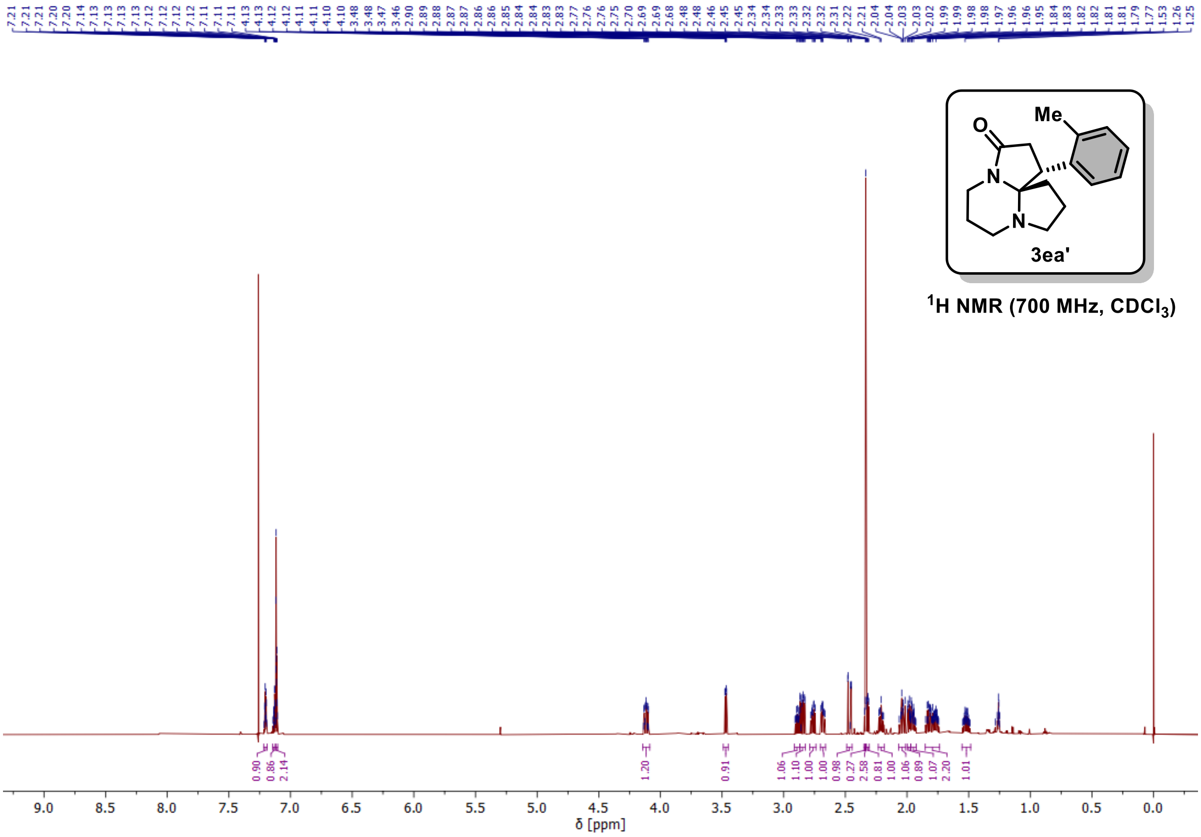

^{1}H NMR (700 MHz, CDCl$_3$)

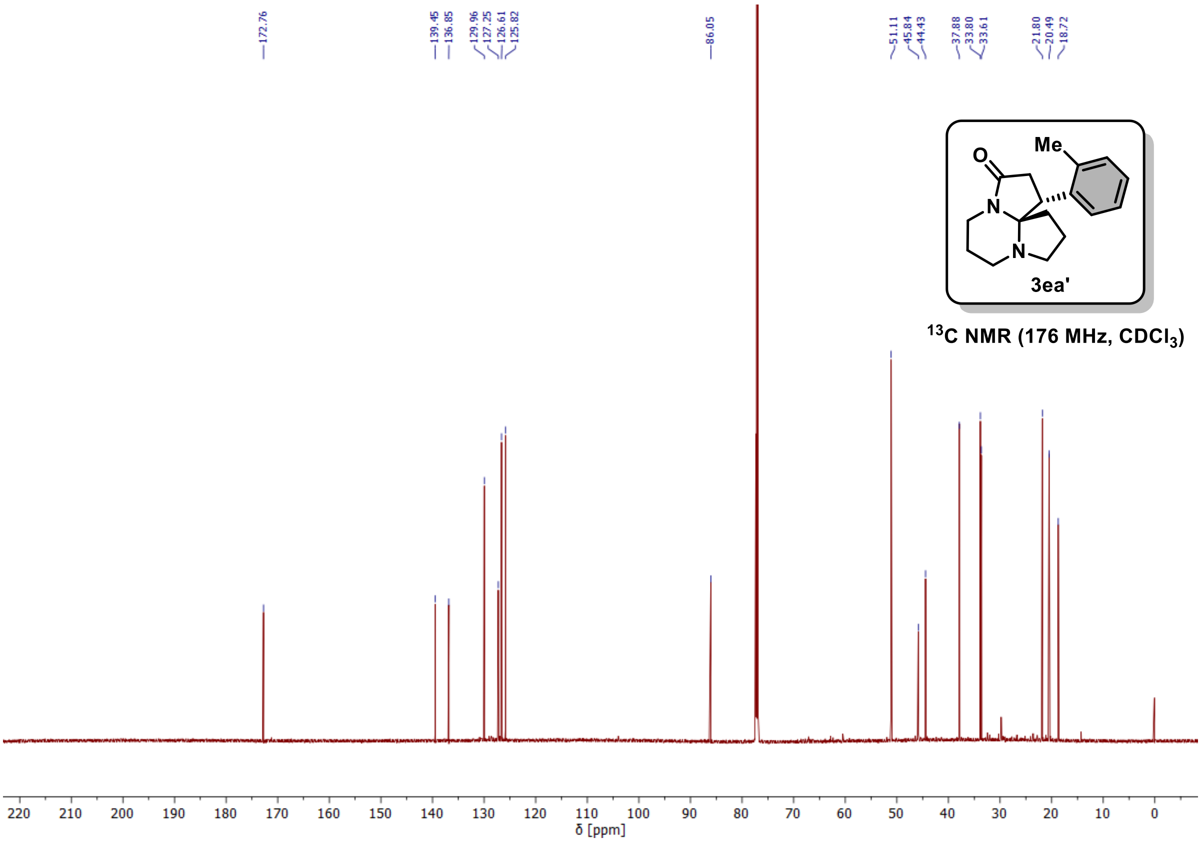

^{13}C NMR (176 MHz, CDCl$_3$)

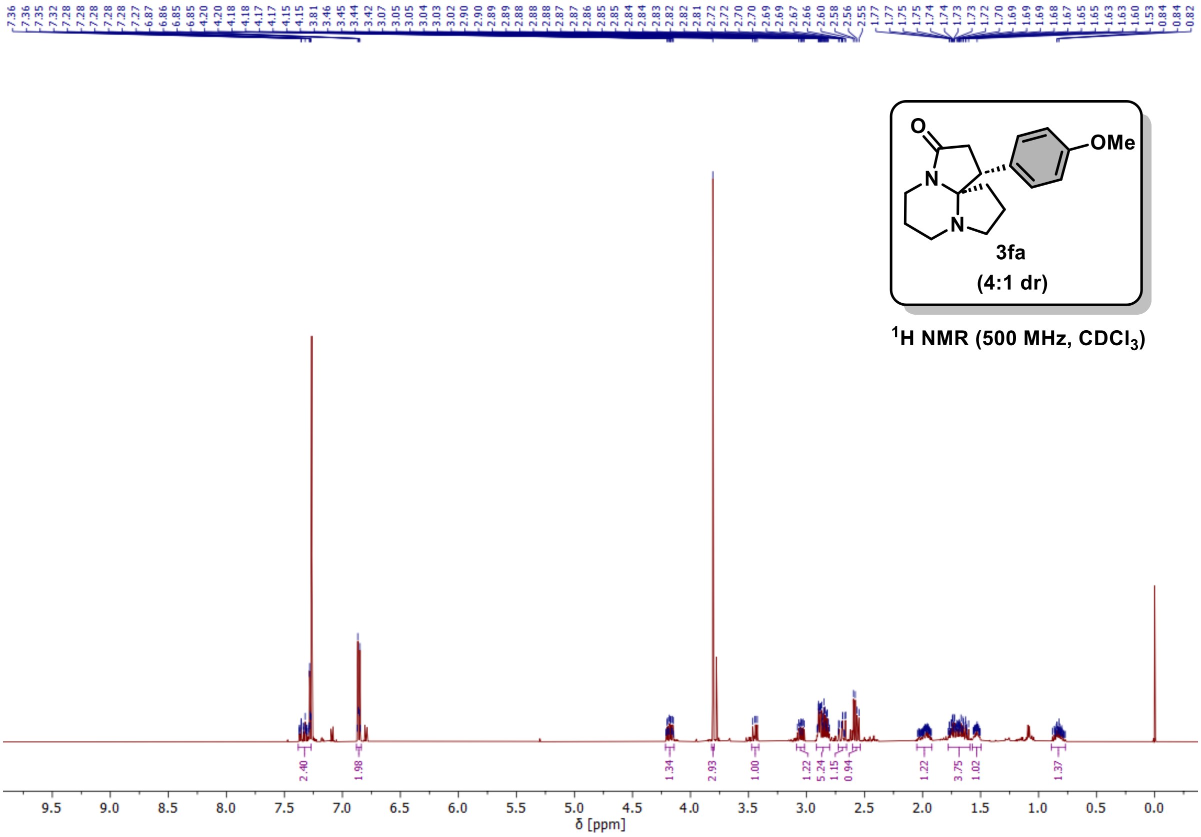
OMe
O
N
N
3fa
(4:1 dr)
1H NMR (500 MHz, CDCl3)
δ [ppm]

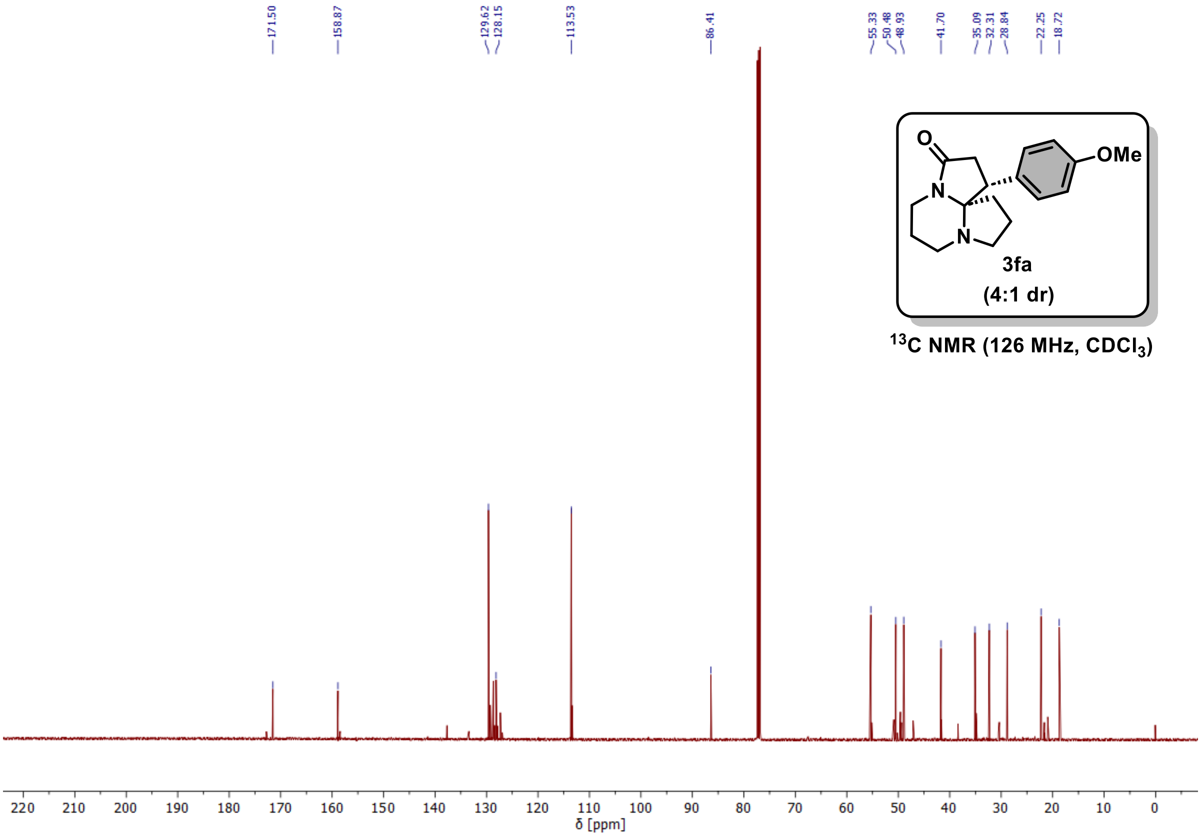
171.50
158.87
129.62
128.15
113.53
86.41
55.33
50.48
48.93
41.70
35.09
32.31
28.84
22.25
18.72
OMe
O
N
N
3fa
(4:1 dr)
13C NMR (126 MHz, CDCl3)
δ [ppm]

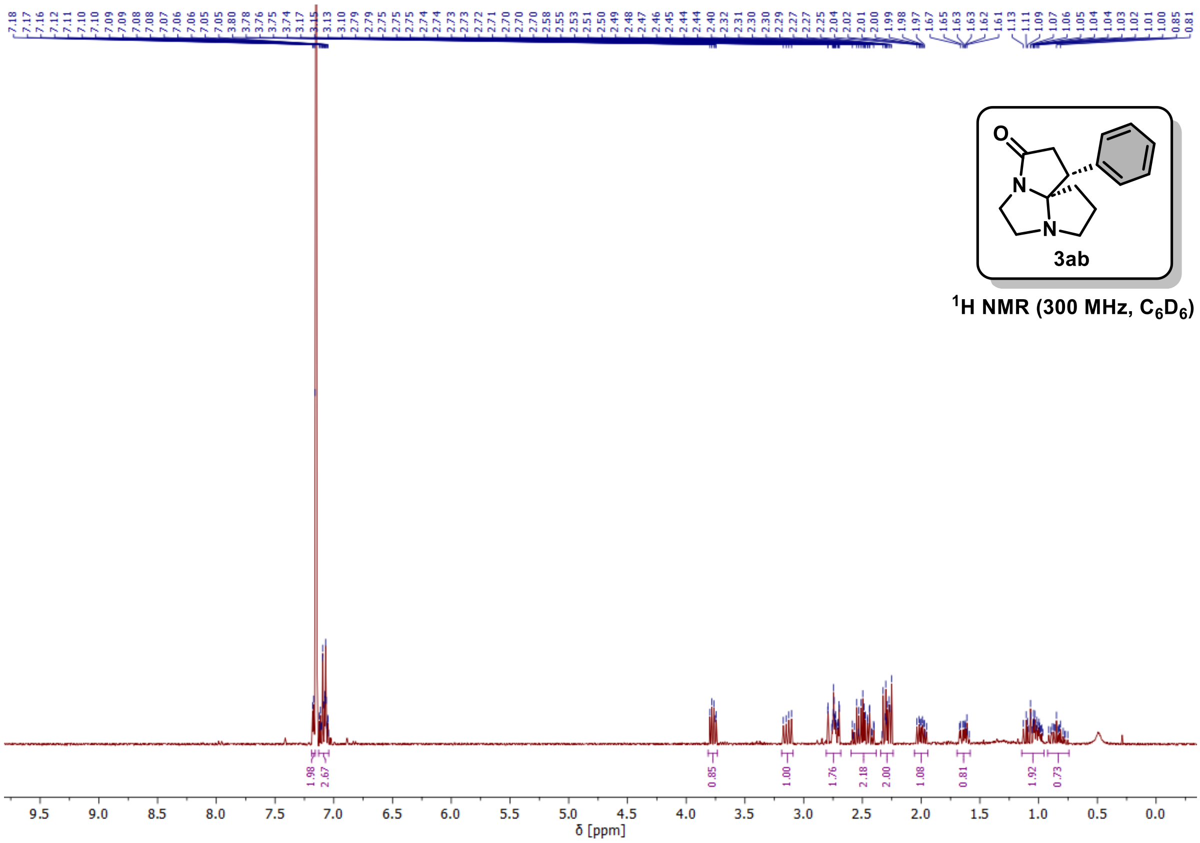

A clean ^{13}C NMR spectrum of this compound could not be obtained, due to rapid decomposition upon isolation.

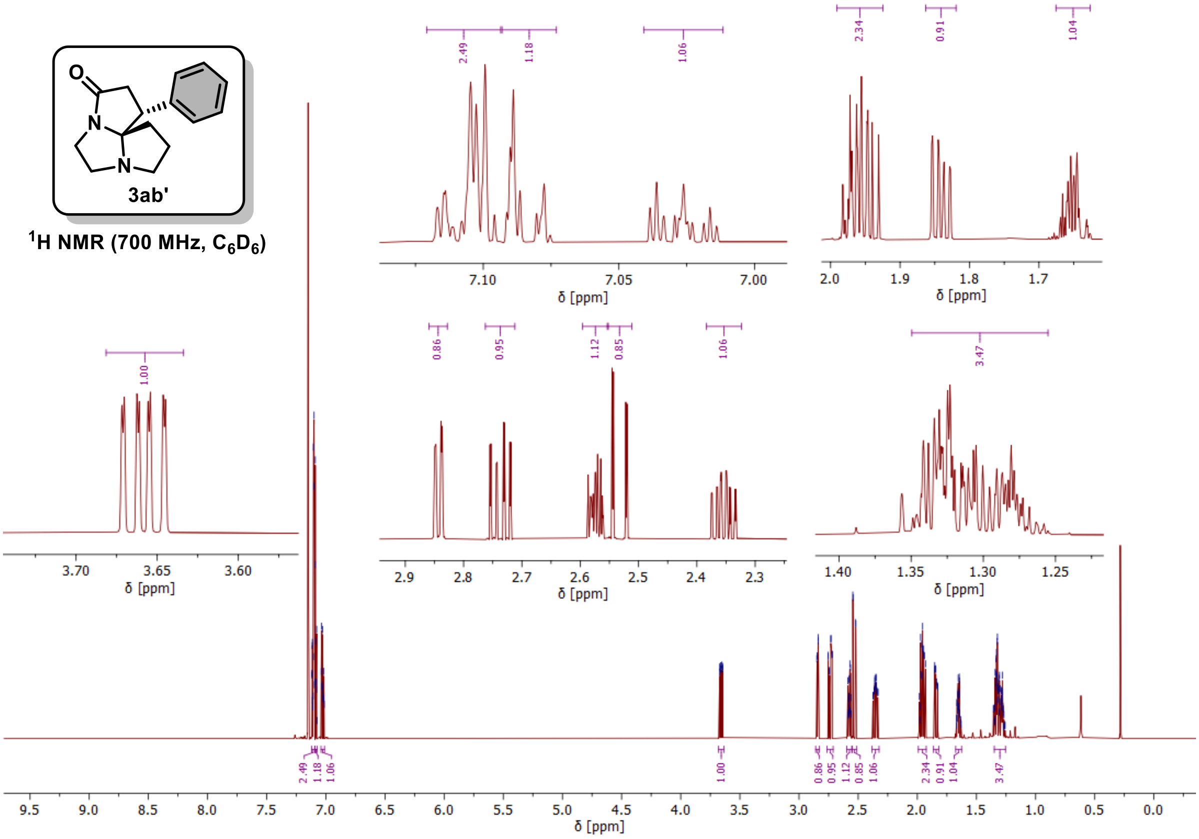
3ab'
1H NMR (700 MHz, C6D6)
δ [ppm]

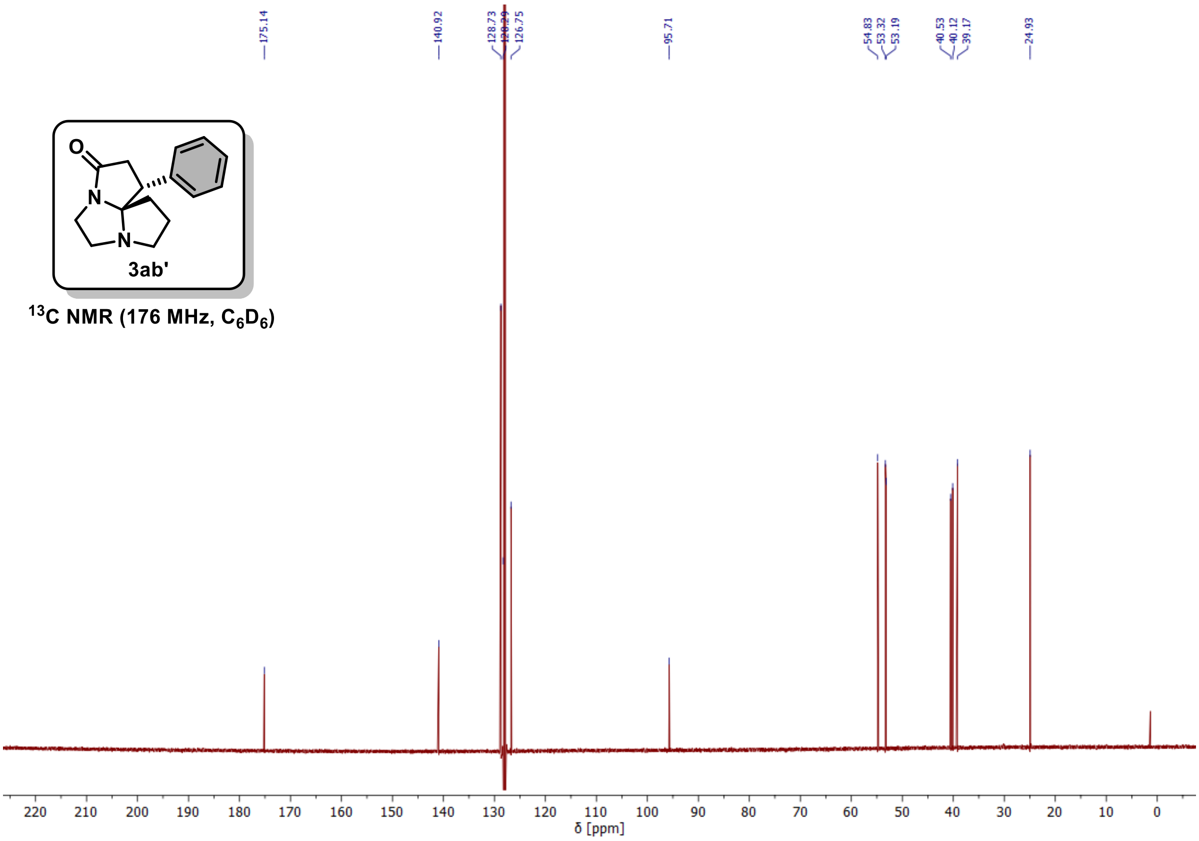
175.14
140.92
128.73
126.75
95.71
54.83
53.32
53.19
40.53
40.12
39.17
24.93
3ab'
13C NMR (176 MHz, C6D6)
δ [ppm]

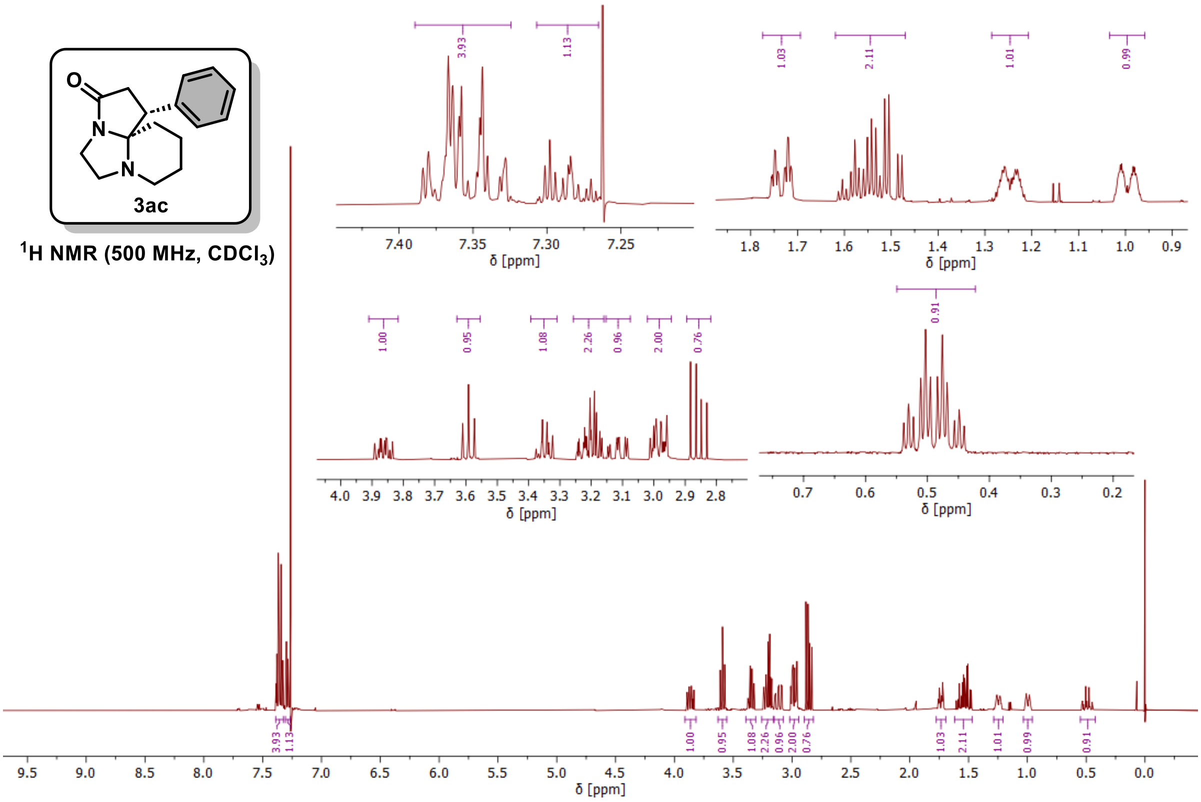
3ac
^{1}H NMR (500 MHz, $CDCl_3$)
δ [ppm]

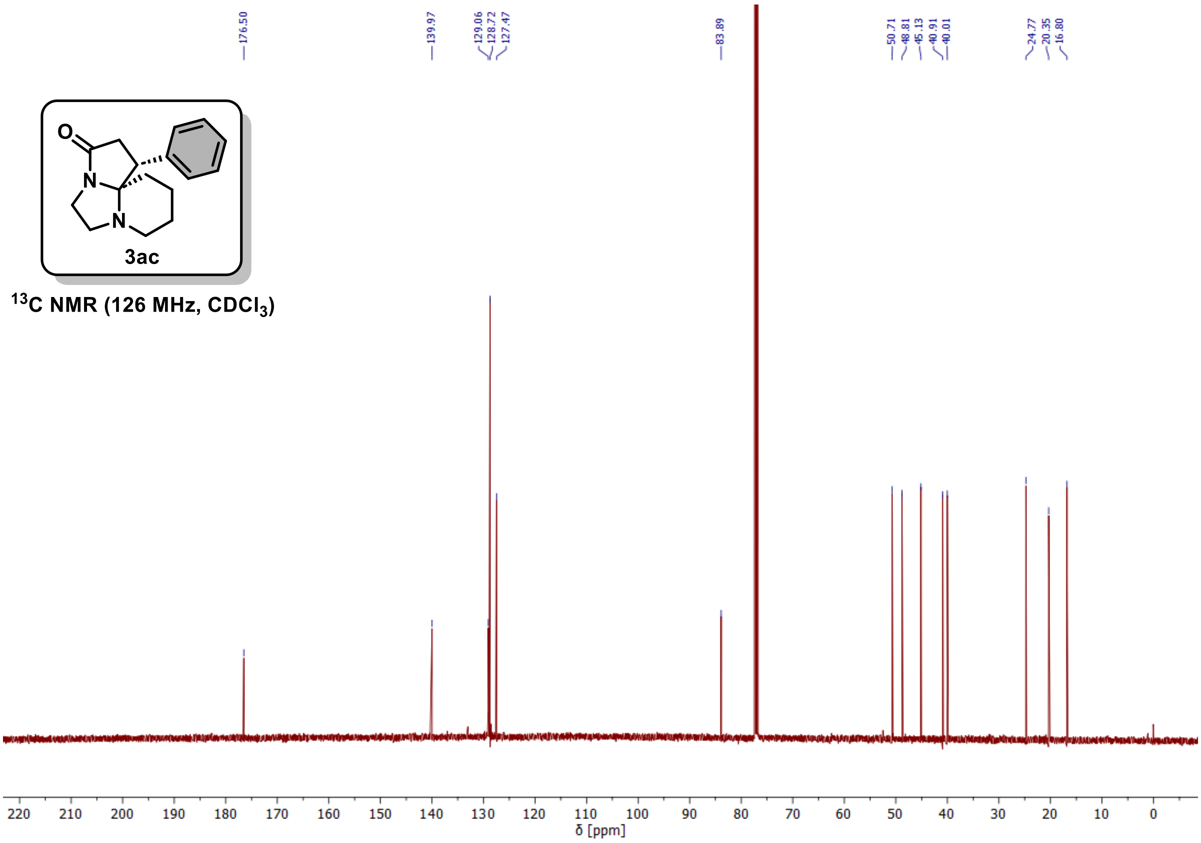
3ac
^{13}C NMR (126 MHz, $CDCl_3$)
176.50
139.97
129.06
128.72
127.47
83.89
50.71
48.81
45.13
40.91
40.01
24.77
20.35
16.80
δ [ppm]

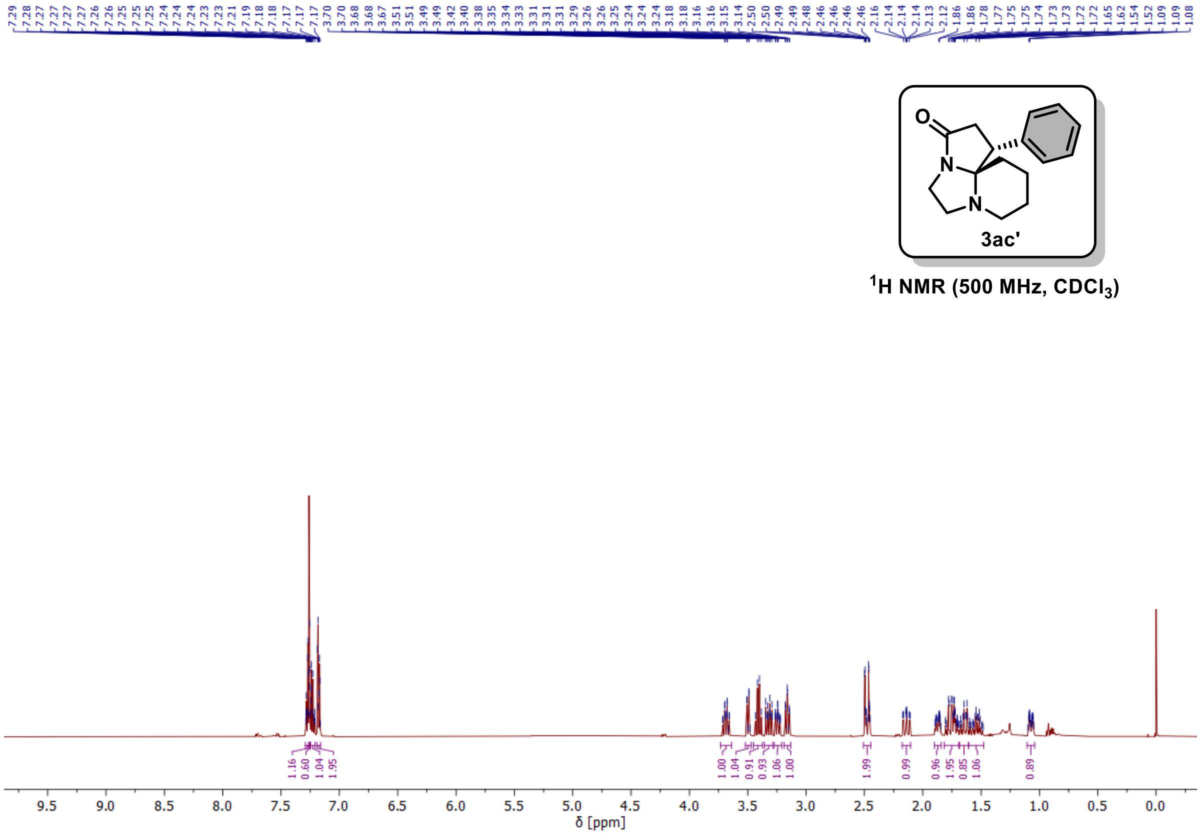

^{1}H NMR (500 MHz, $CDCl_3$)

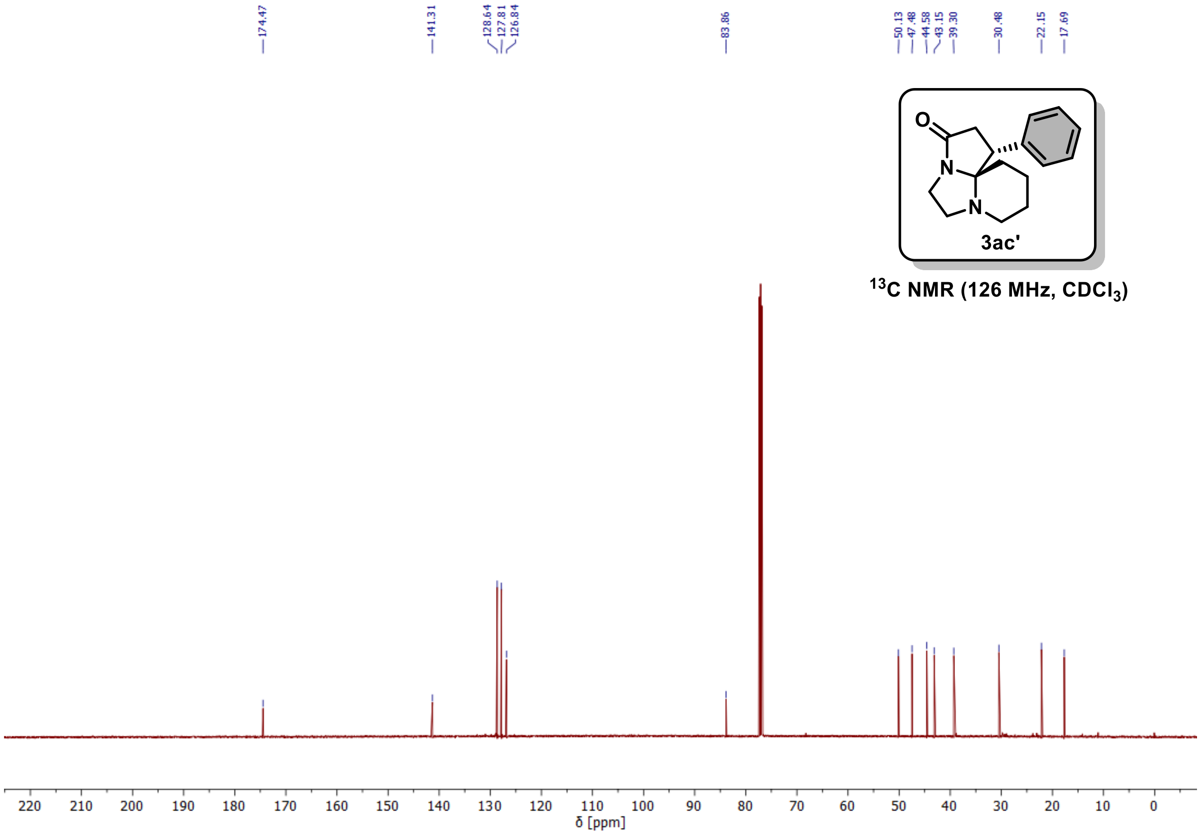

^{13}C NMR (126 MHz, $CDCl_3$)

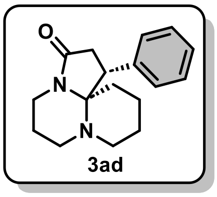

^{1}H NMR (500 MHz, C_6D_6)

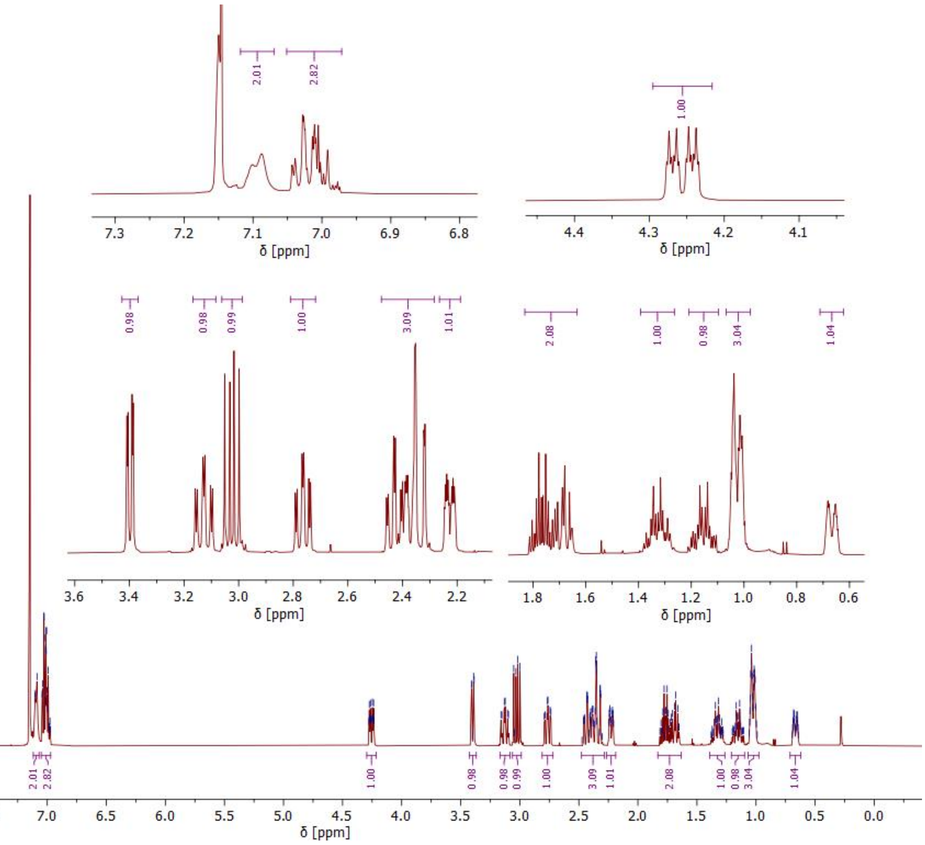

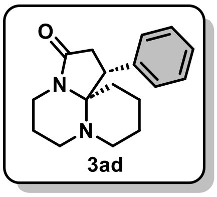

^{13}C NMR (126 MHz, C_6D_6)

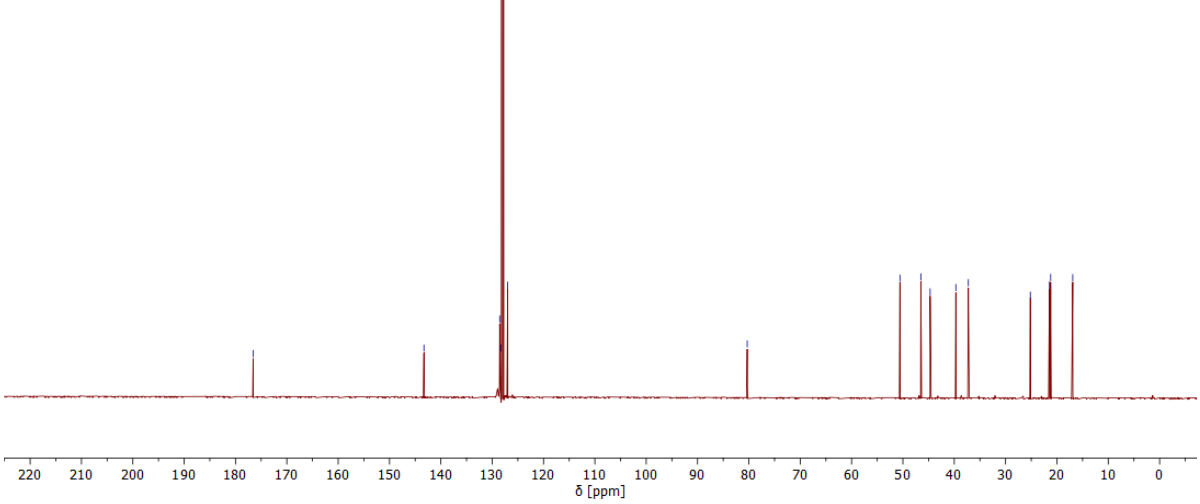

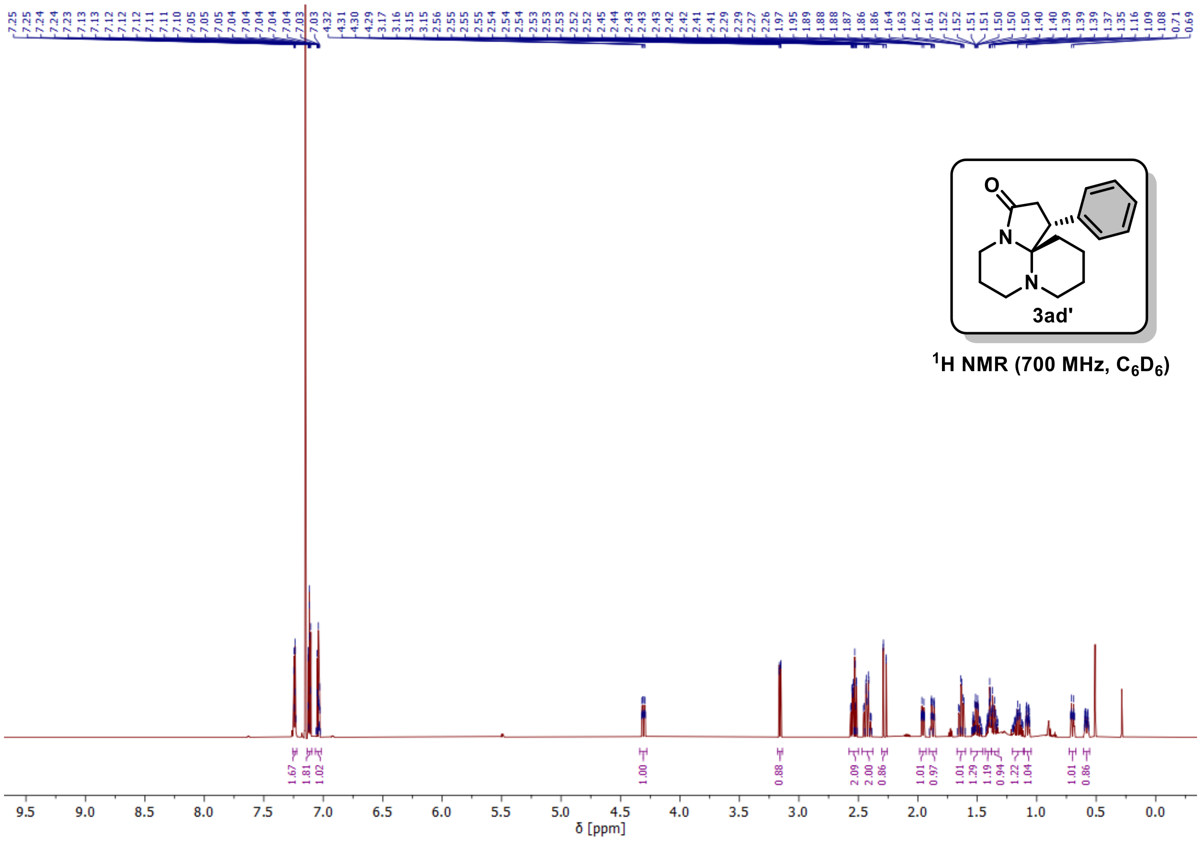

^1H NMR (700 MHz, C_6D_6)

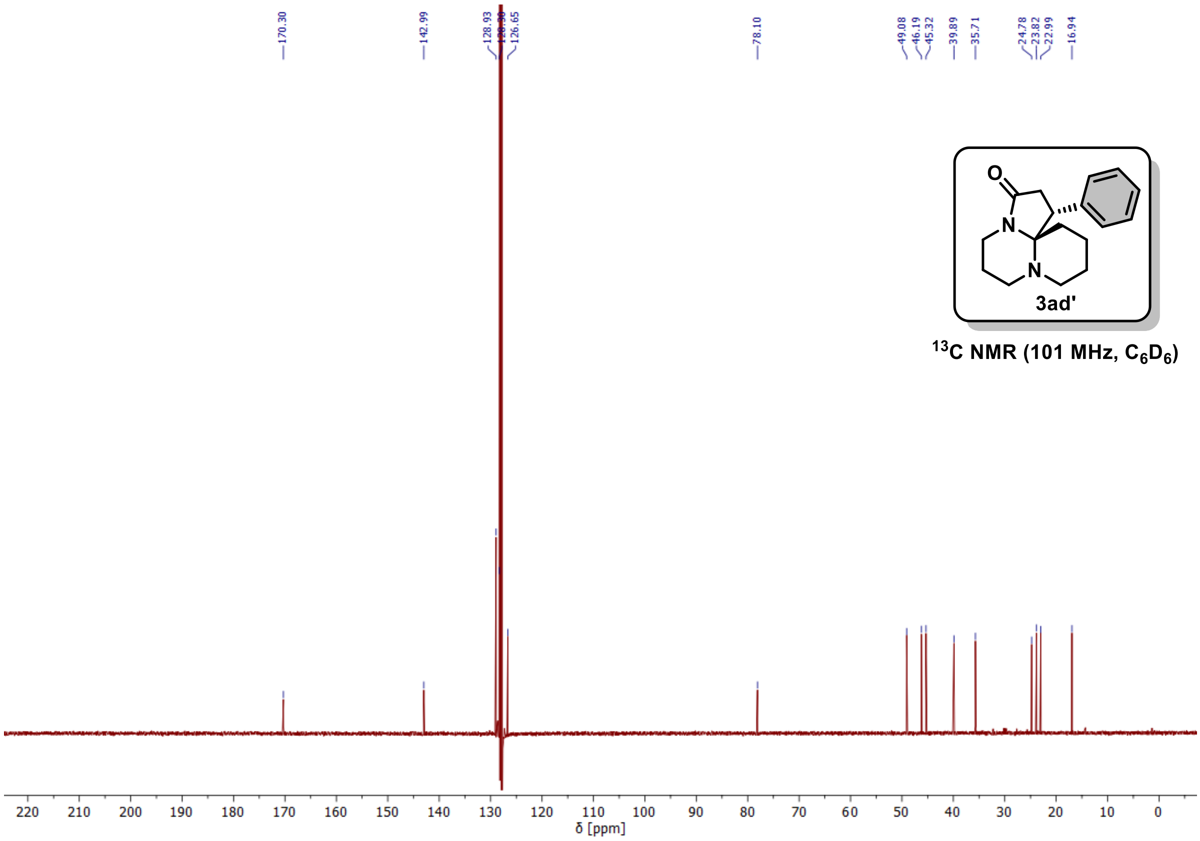

^{13}C NMR (101 MHz, C_6D_6)

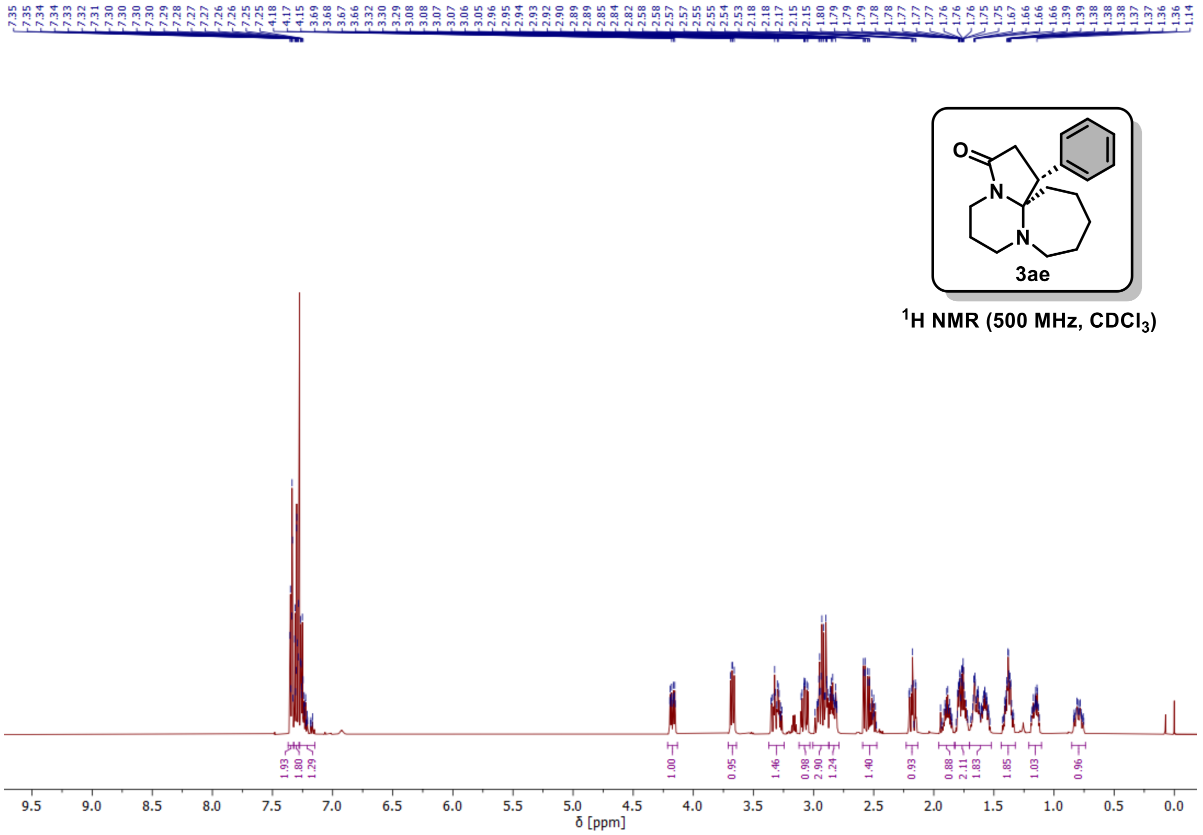
3ae
^{1}H NMR (500 MHz, $CDCl_3$)
δ [ppm]

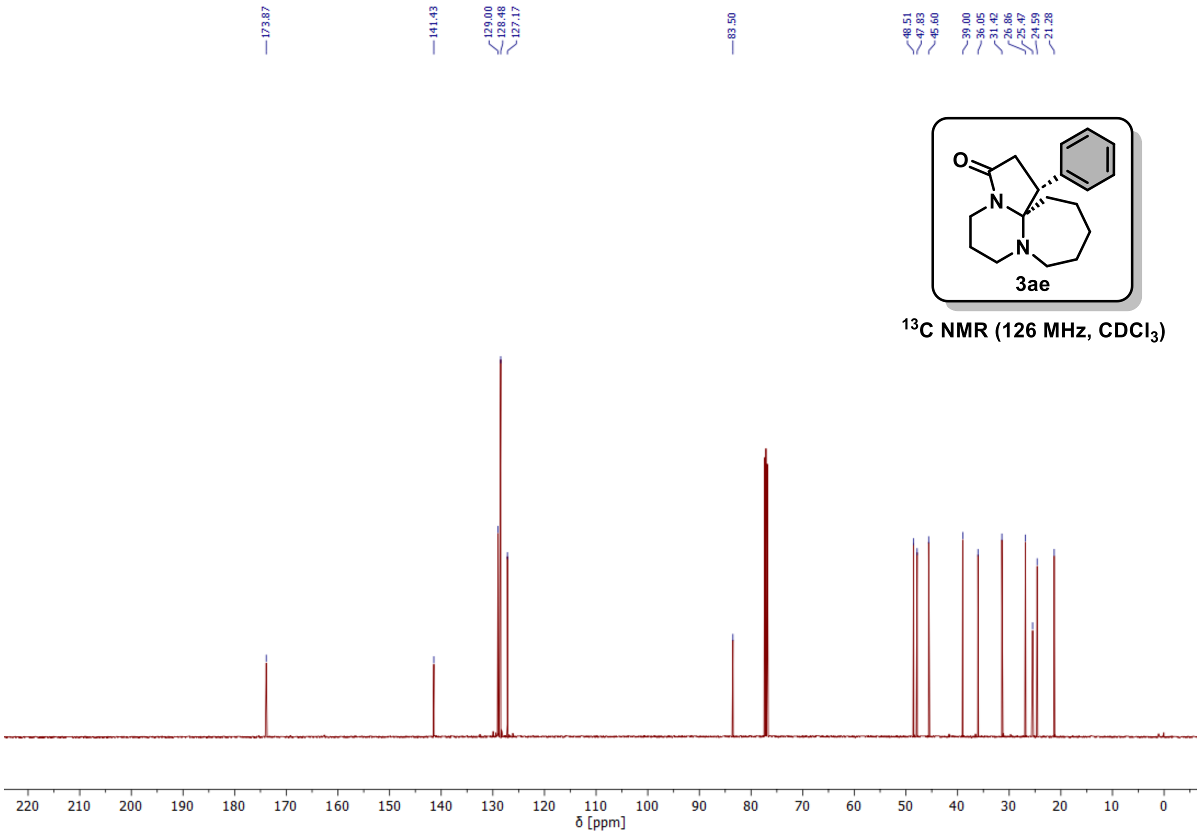
173.87
141.43
129.00
128.48
127.17
83.50
48.51
47.83
45.60
39.00
36.05
31.42
26.86
25.47
24.59
21.28
3ae
^{13}C NMR (126 MHz, $CDCl_3$)
δ [ppm]

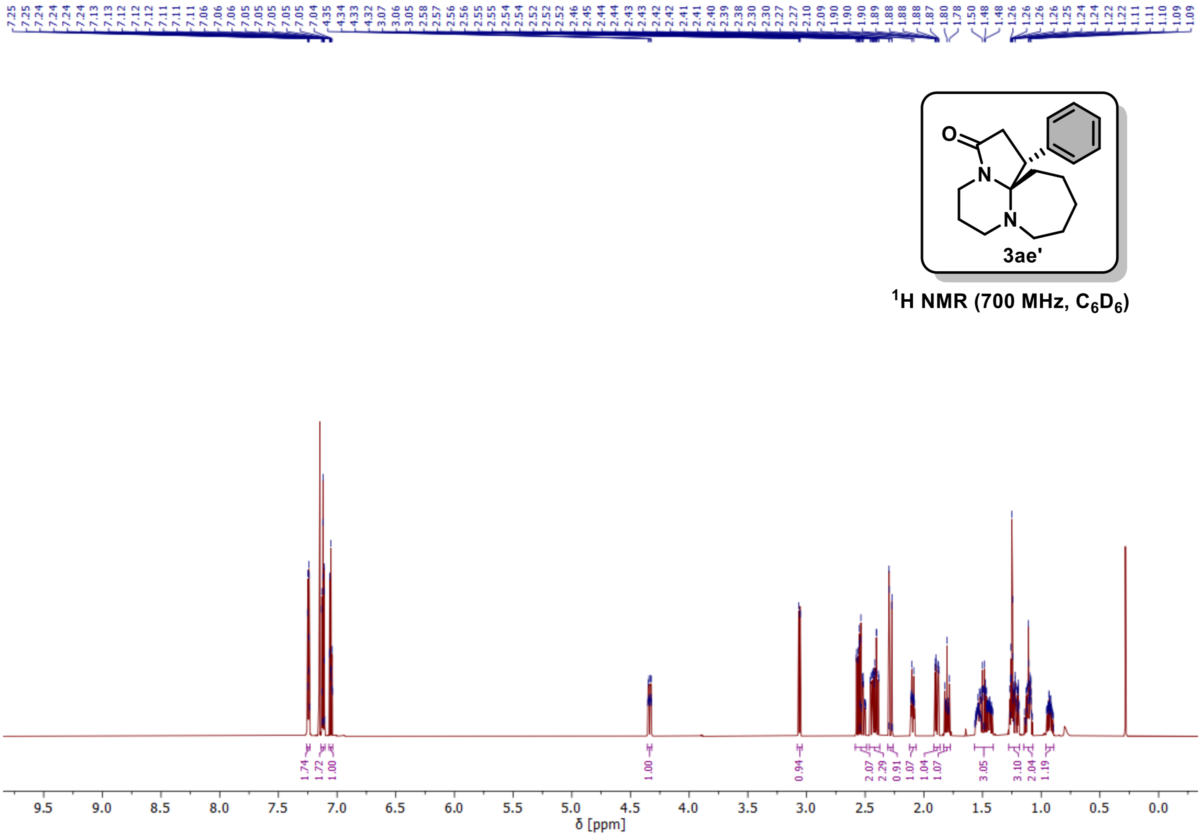

^{1}H NMR (700 MHz, C_6D_6)

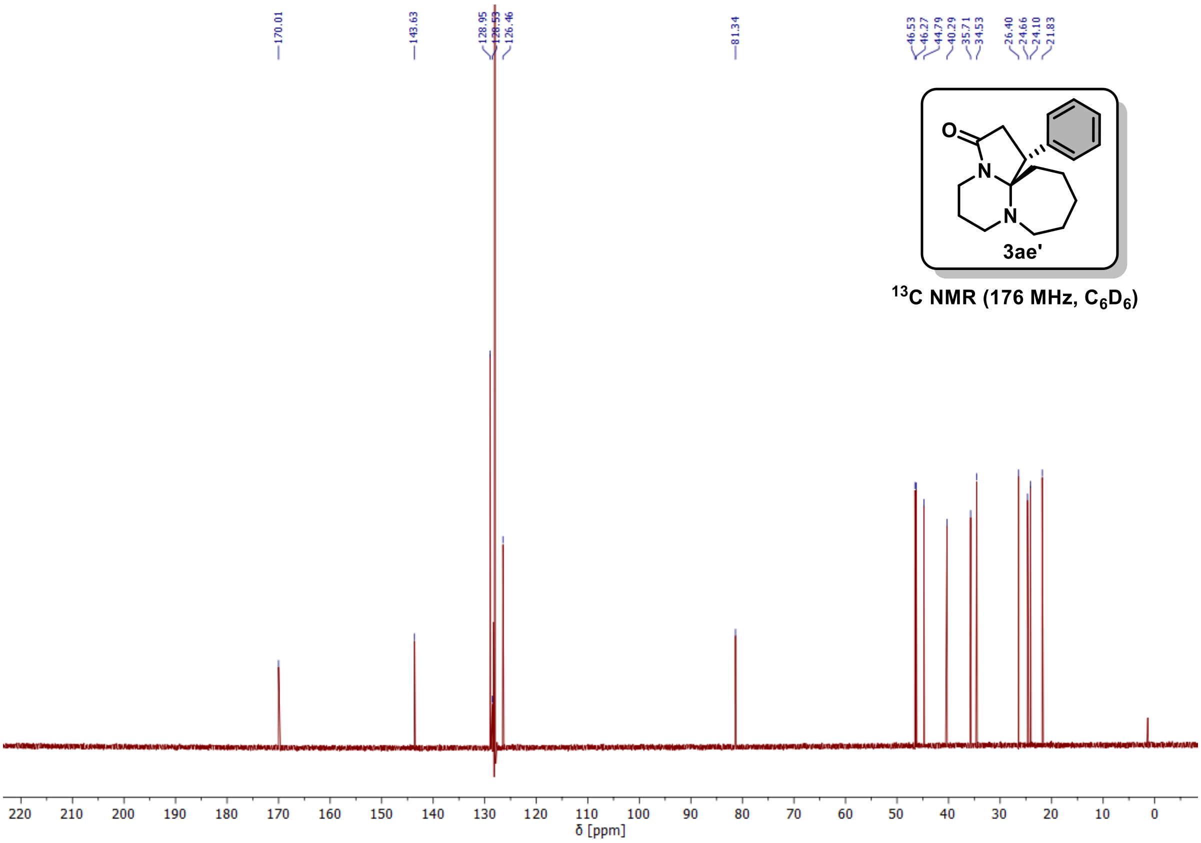

^{13}C NMR (176 MHz, C_6D_6)

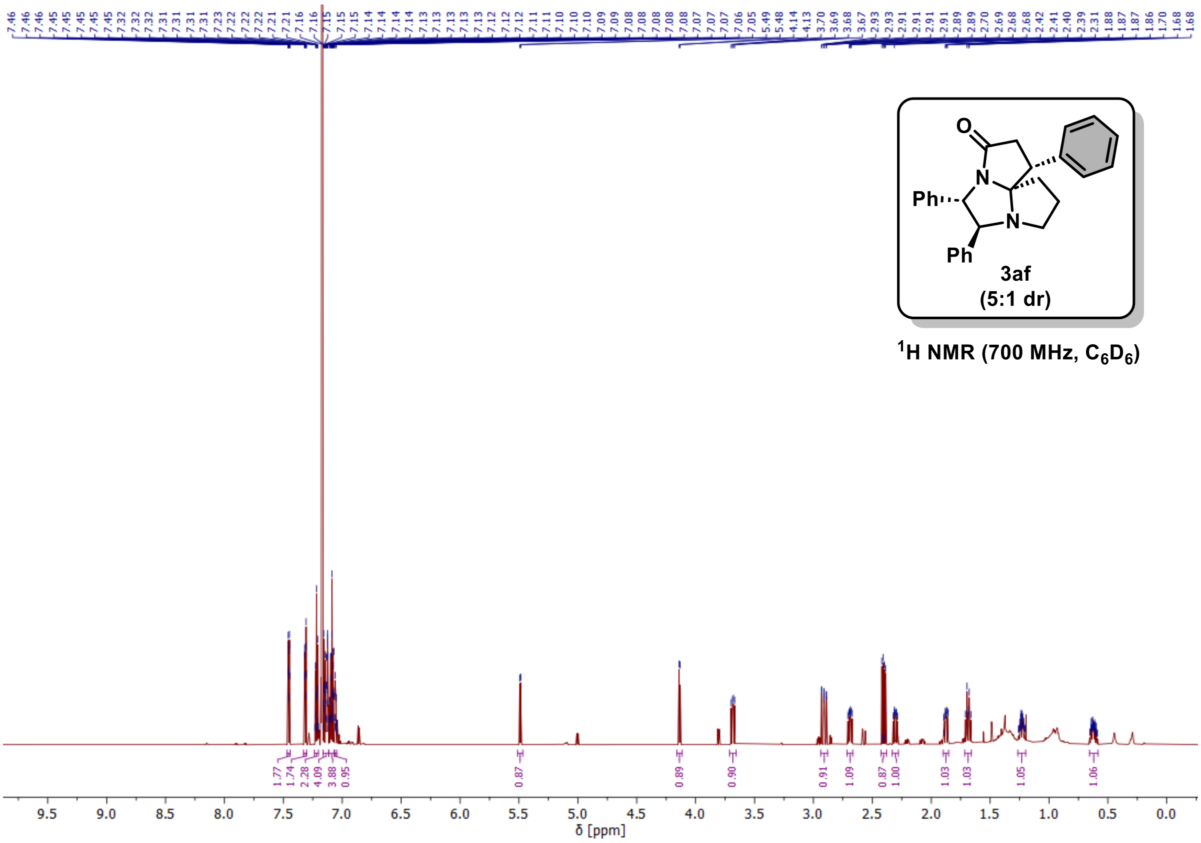
3af
(5:1 dr)
1H NMR (700 MHz, C6D6)
δ [ppm]

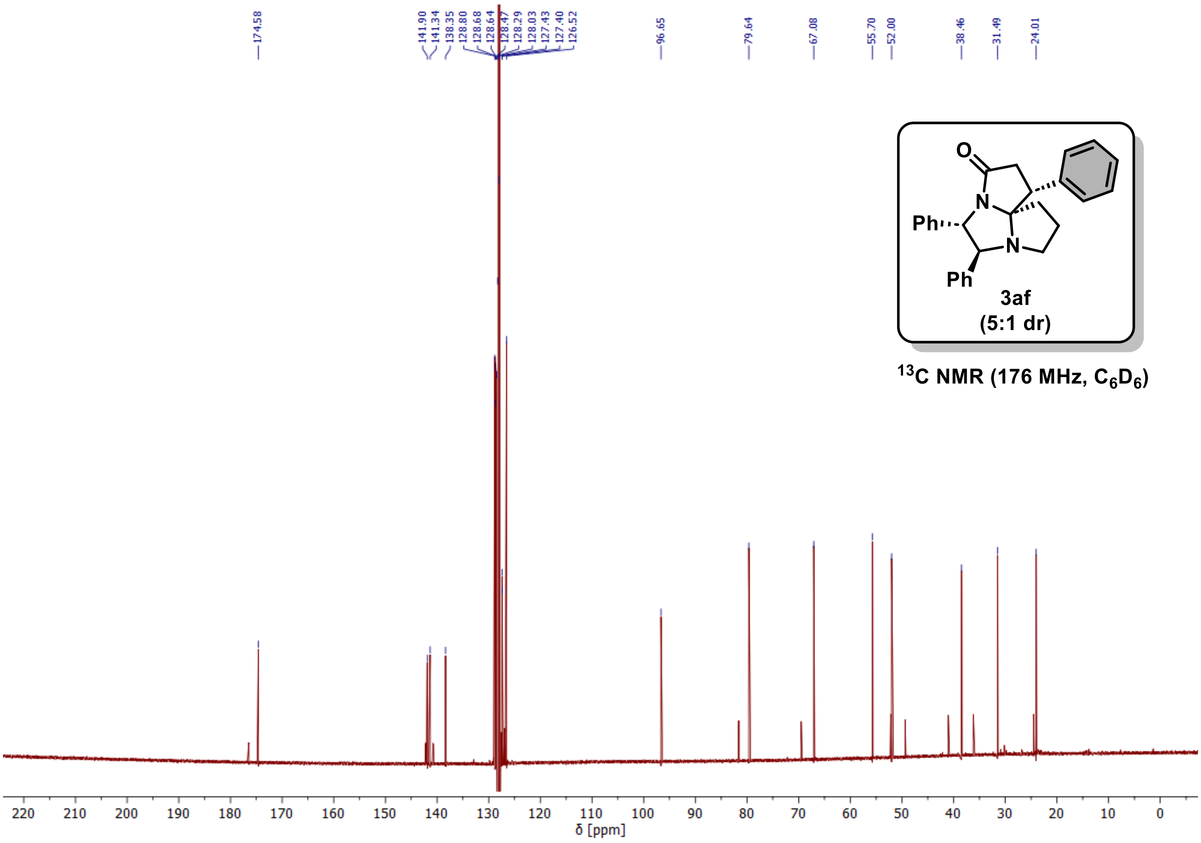
3af
(5:1 dr)
13C NMR (176 MHz, C6D6)
δ [ppm]

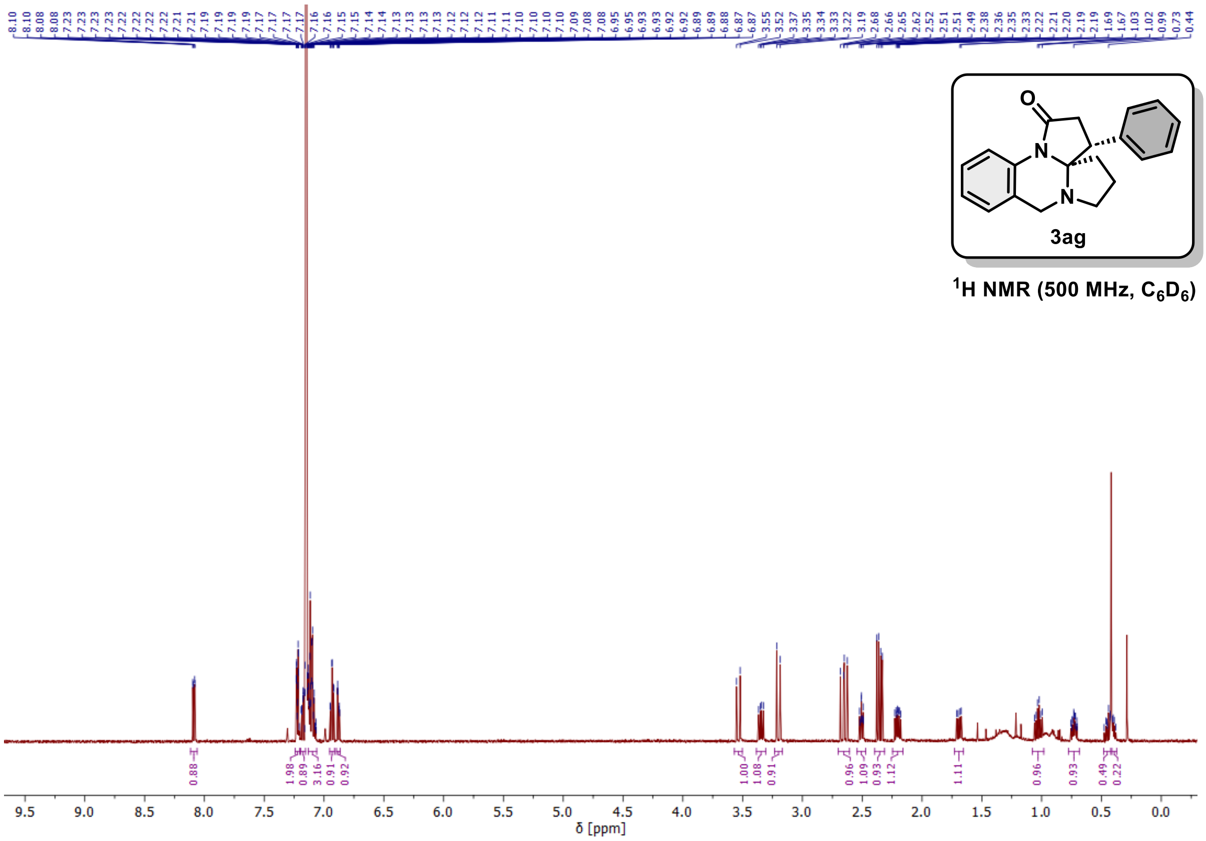
3ag
^{1}H NMR (500 MHz, C_6D_6)
δ [ppm]

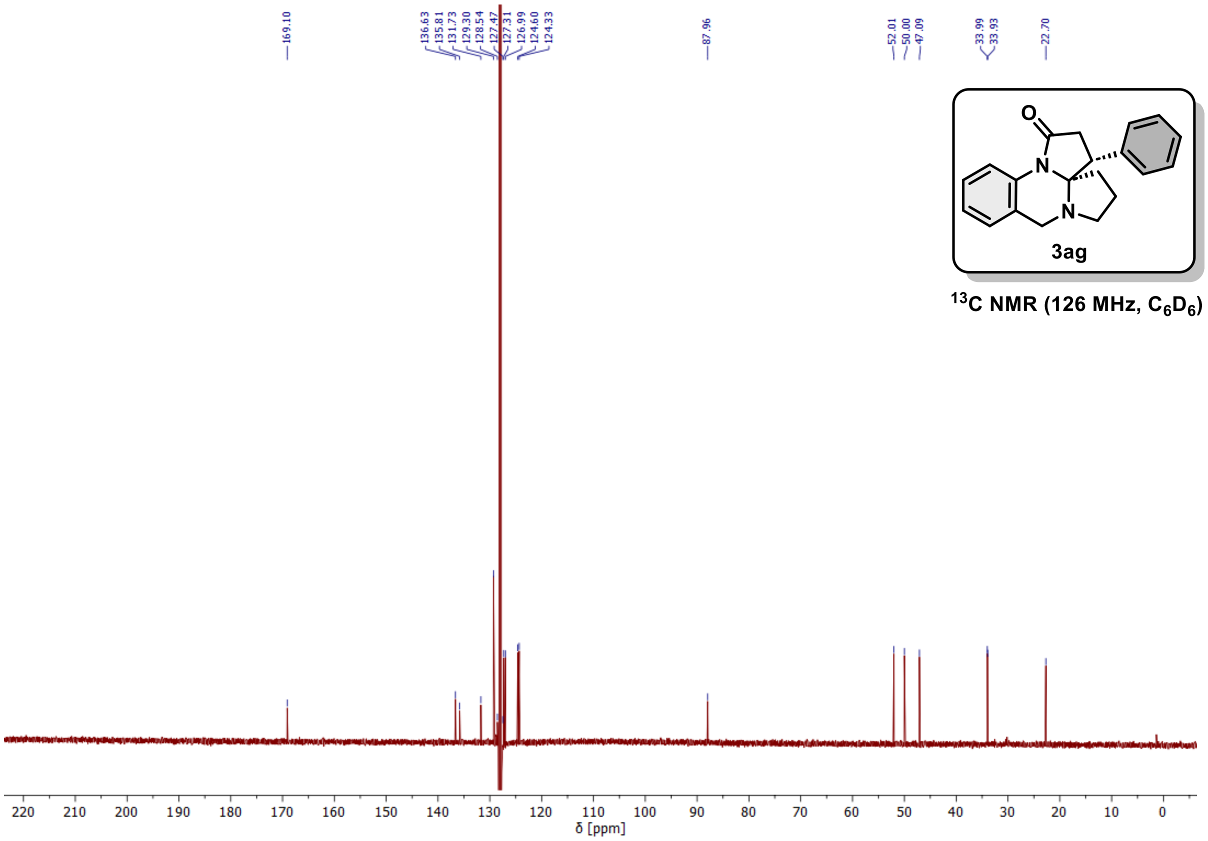
3ag
^{13}C NMR (126 MHz, C_6D_6)
δ [ppm]

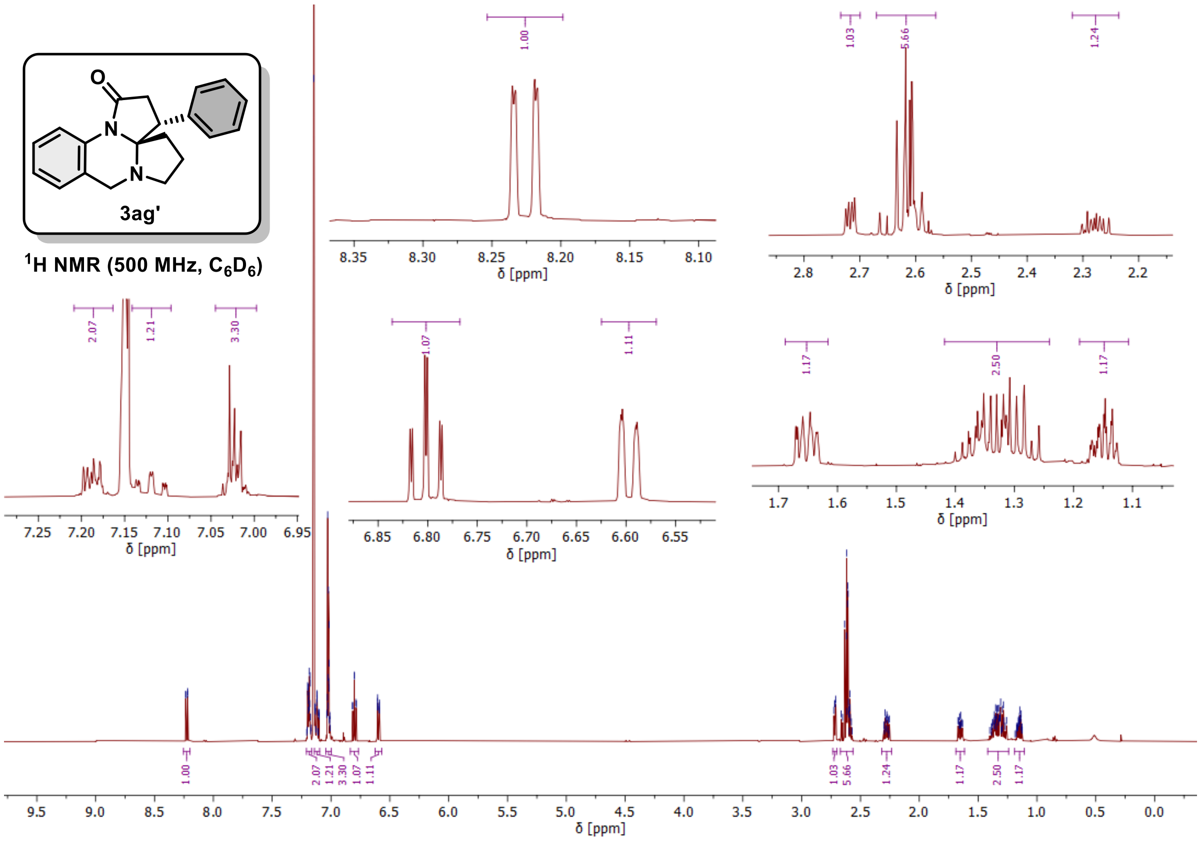

^{1}H NMR (500 MHz, C_6D_6)

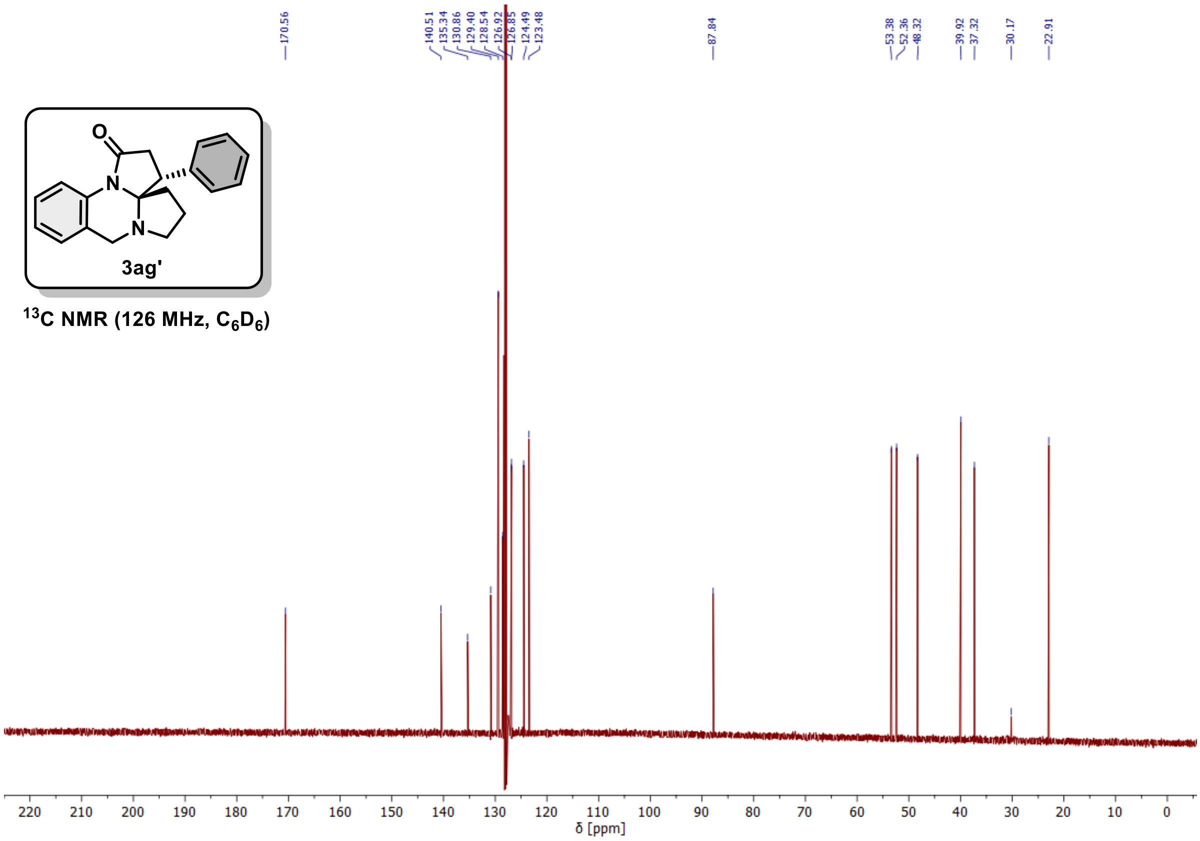

^{13}C NMR (126 MHz, C_6D_6)

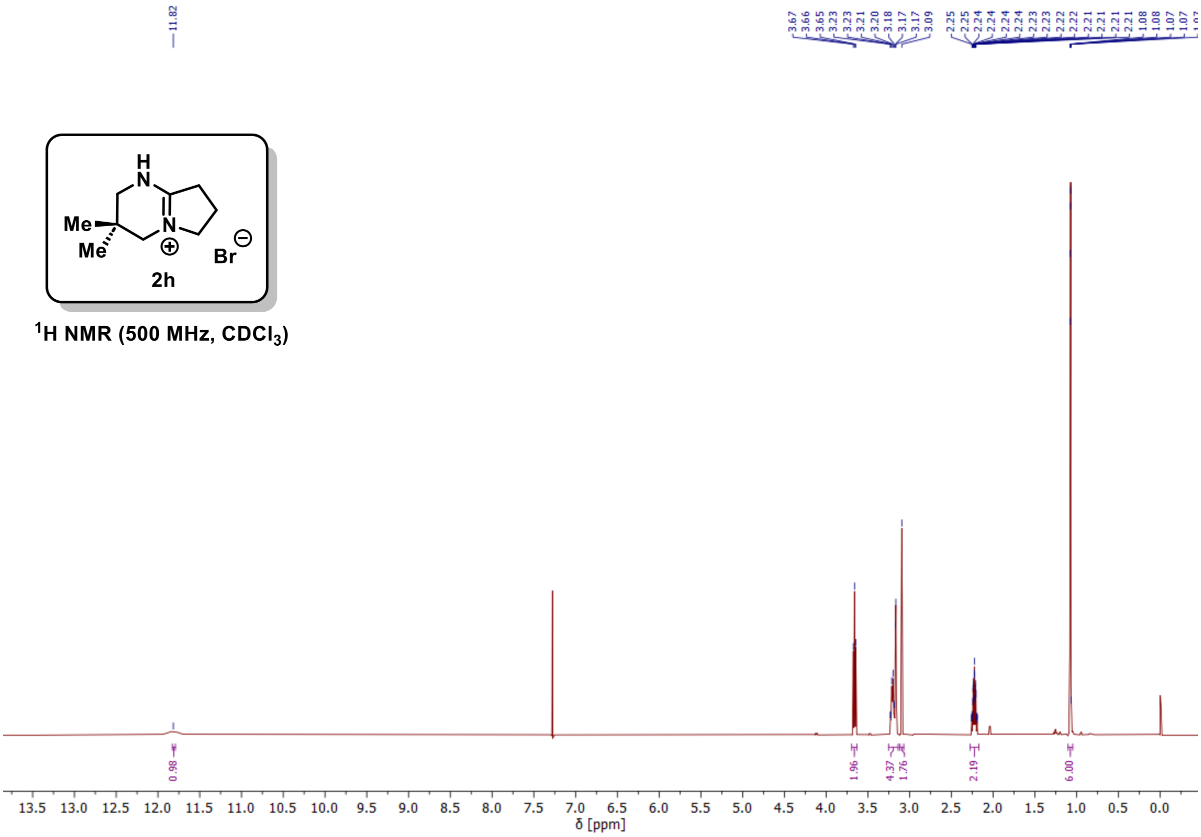

^{1}H NMR (500 MHz, $CDCl_3$)

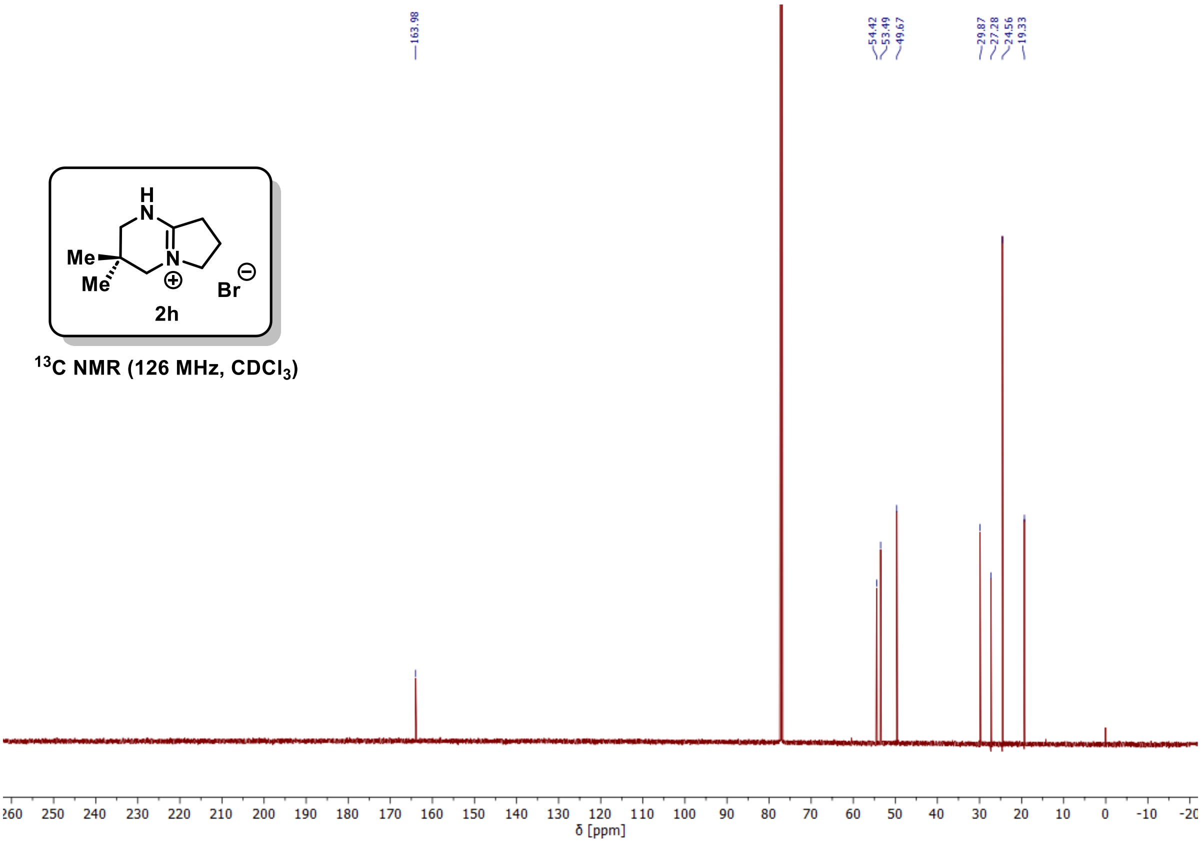

^{13}C NMR (126 MHz, $CDCl_3$)

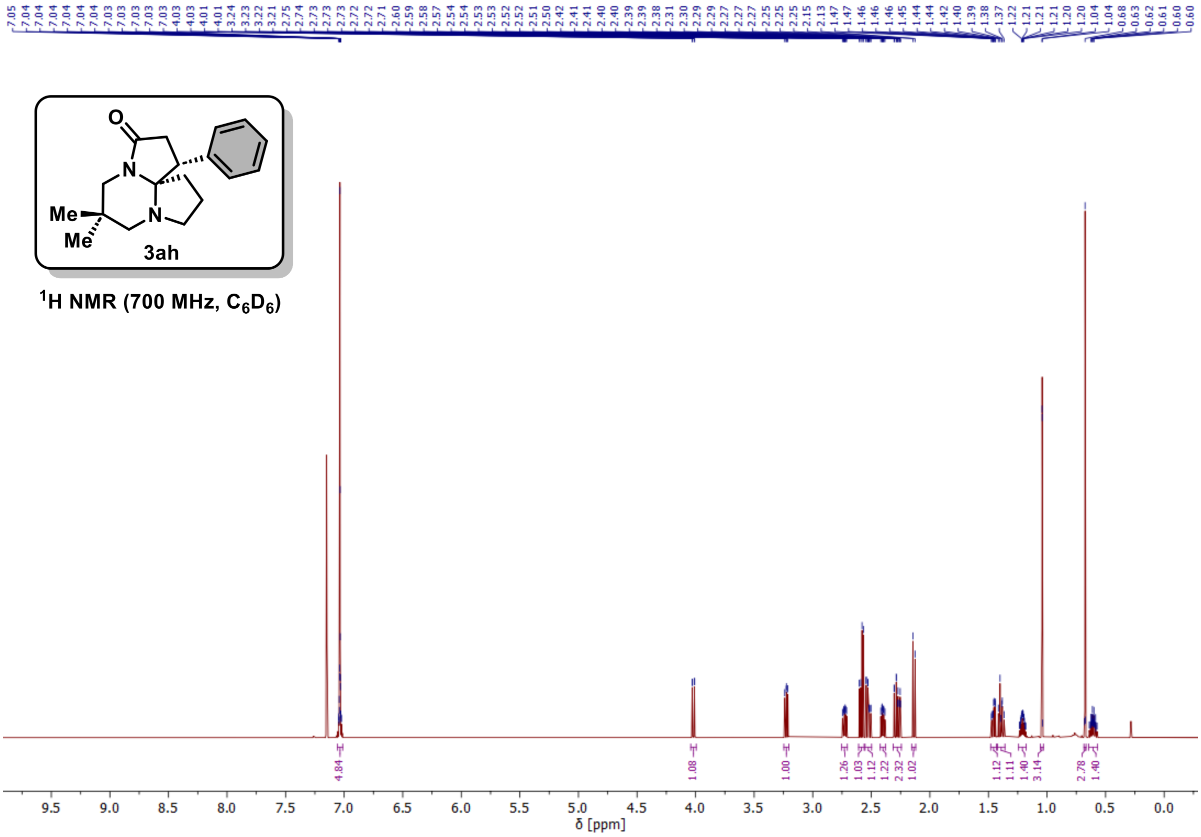
3ah
^{1}H NMR (700 MHz, C_6D_6)
δ [ppm]

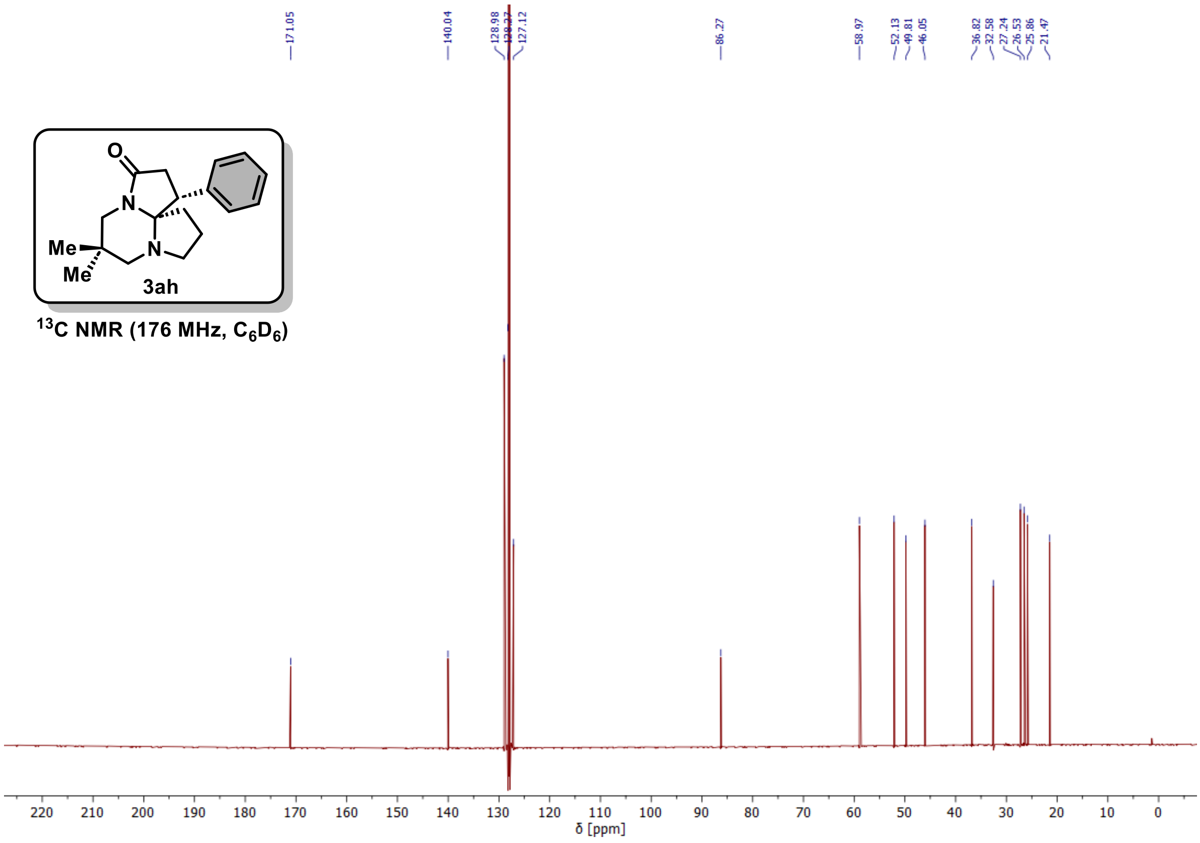
171.05
140.04
128.98
127.12
86.27
58.97
52.13
49.81
46.05
36.82
32.58
27.24
26.53
25.86
21.47
3ah
^{13}C NMR (176 MHz, C_6D_6)
δ [ppm]

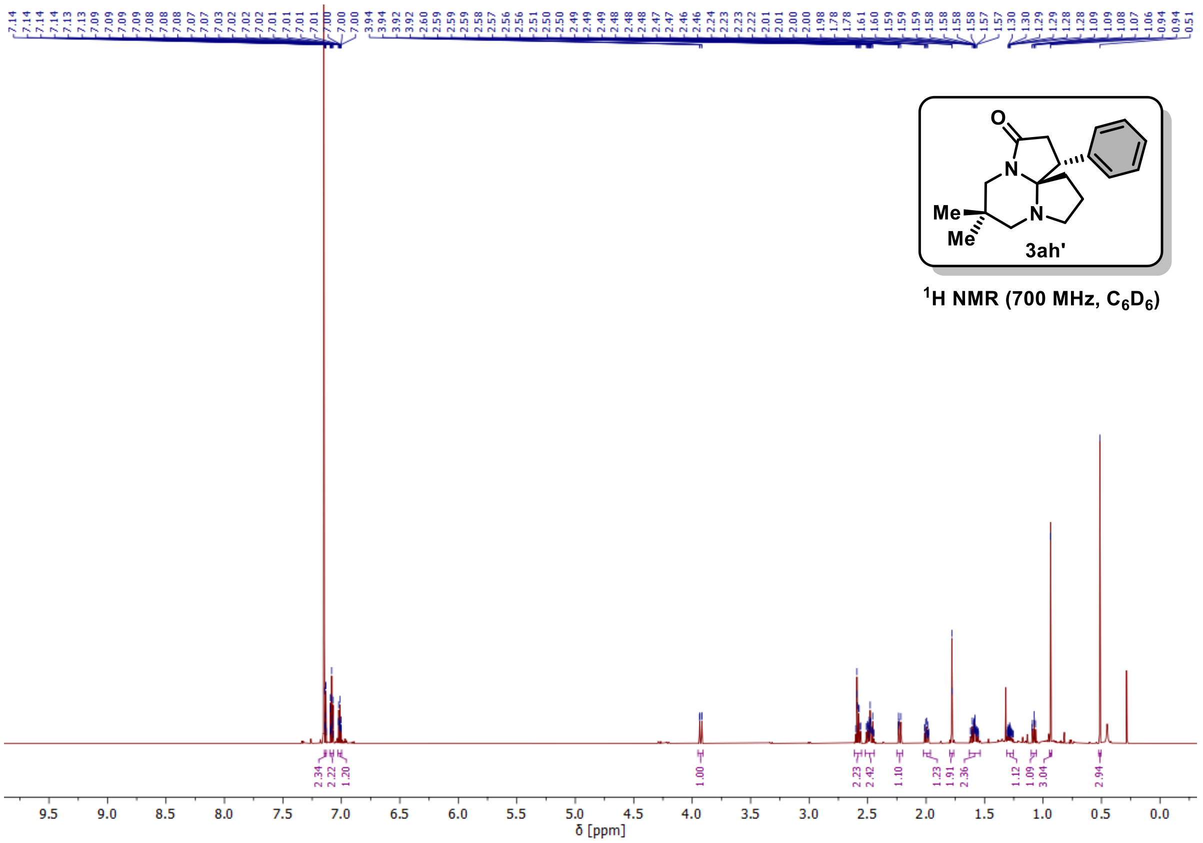
3ah'
^{1}H NMR (700 MHz, C_6D_6)
δ [ppm]

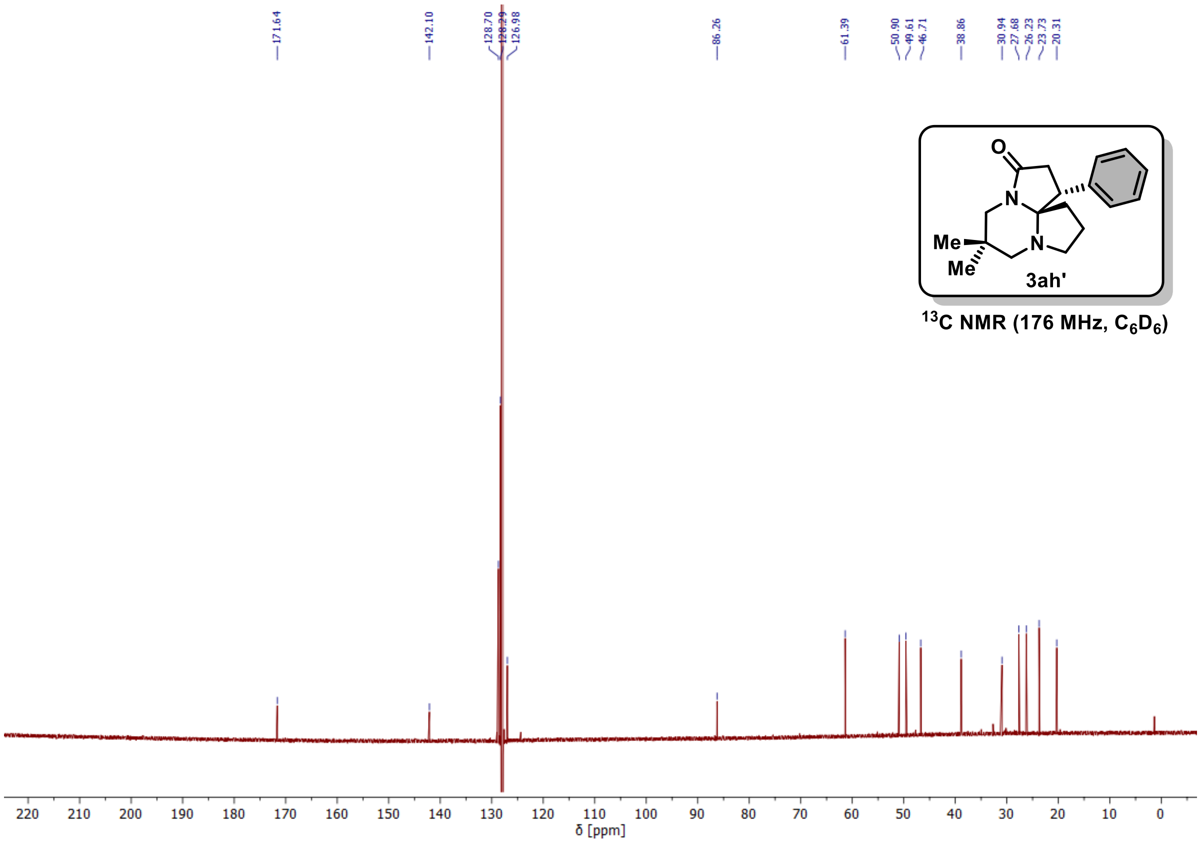
171.64
142.10
128.70
126.98
86.26
61.39
50.90
49.61
46.71
38.86
30.94
27.68
26.23
23.73
20.31
3ah'
^{13}C NMR (176 MHz, C_6D_6)
δ [ppm]

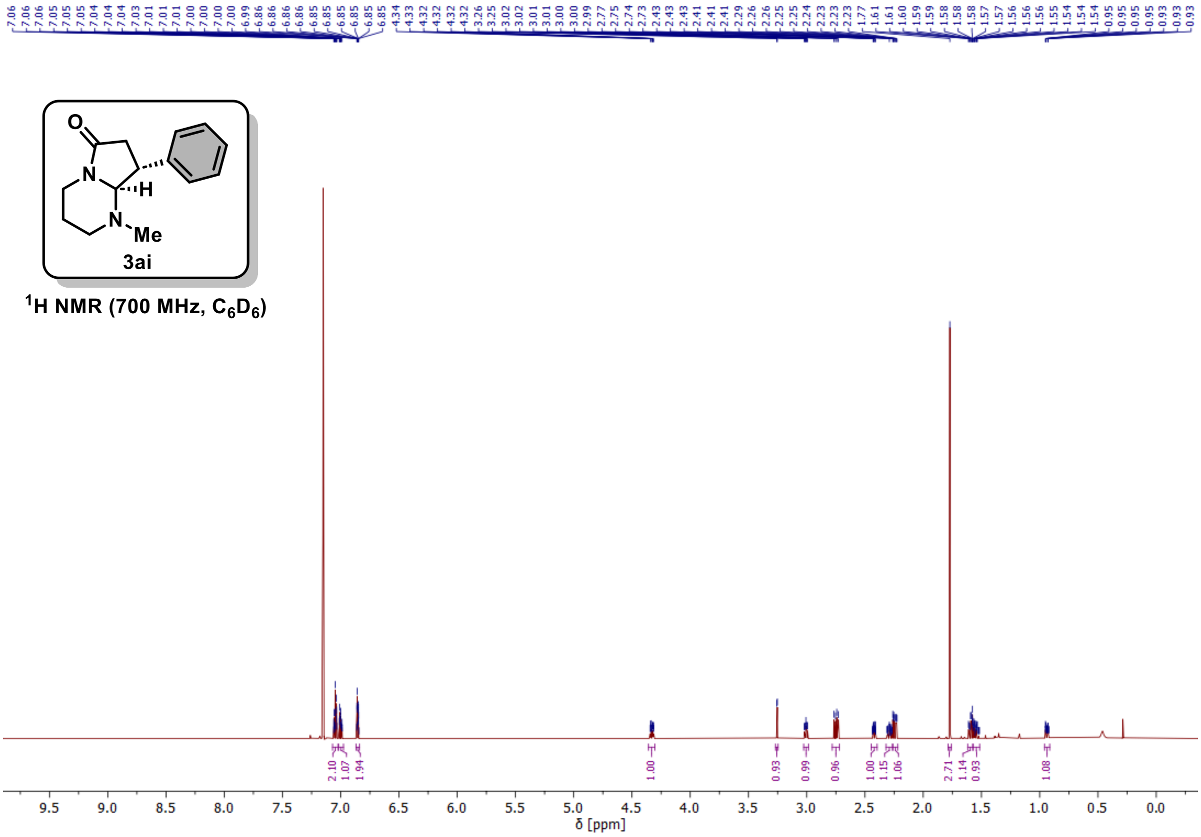

^{1}H NMR (700 MHz, C_6D_6)

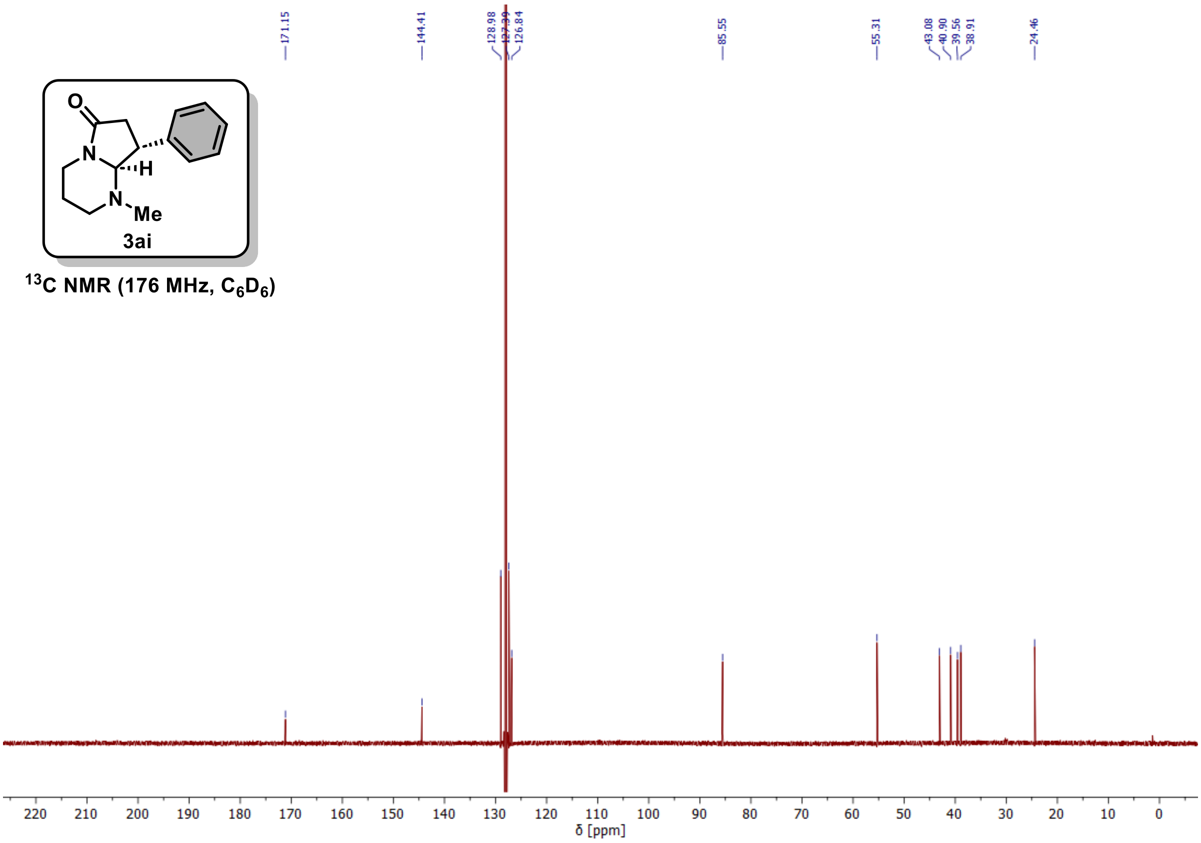

^{13}C NMR (176 MHz, C_6D_6)

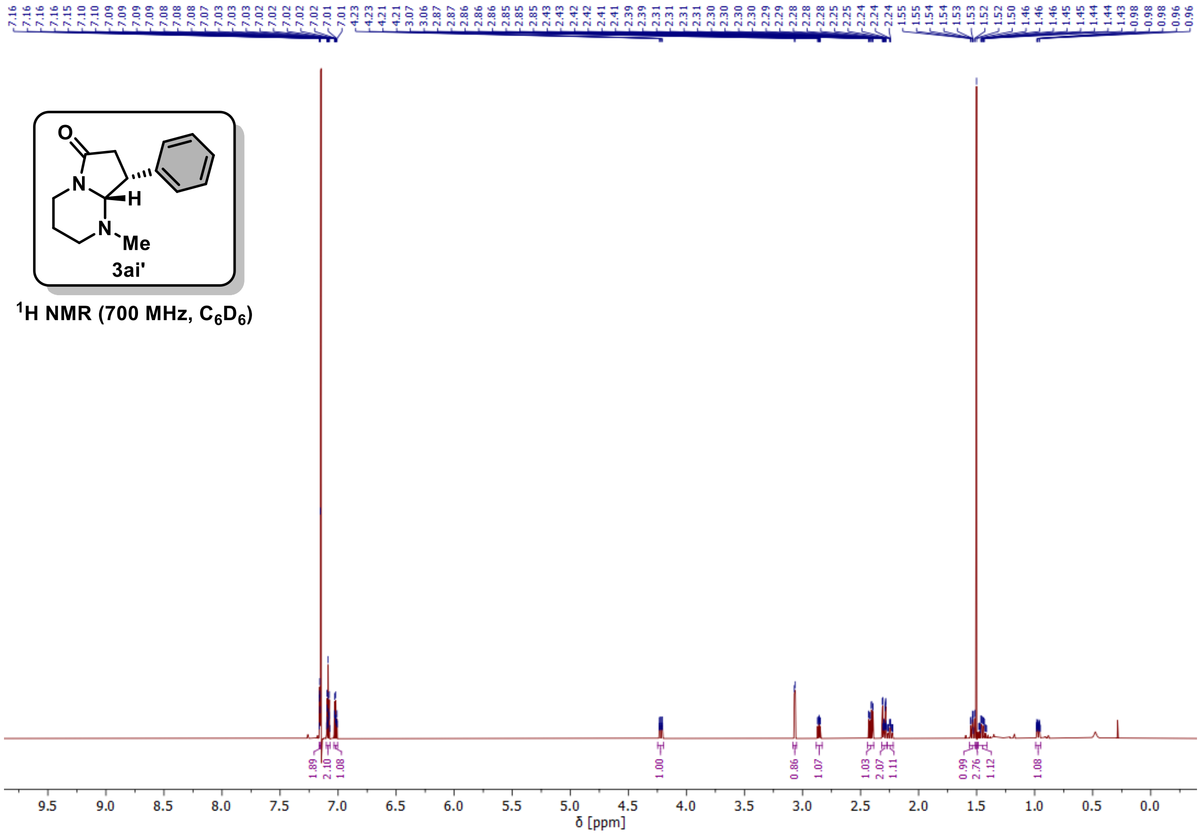

^{1}H NMR (700 MHz, C_6D_6)

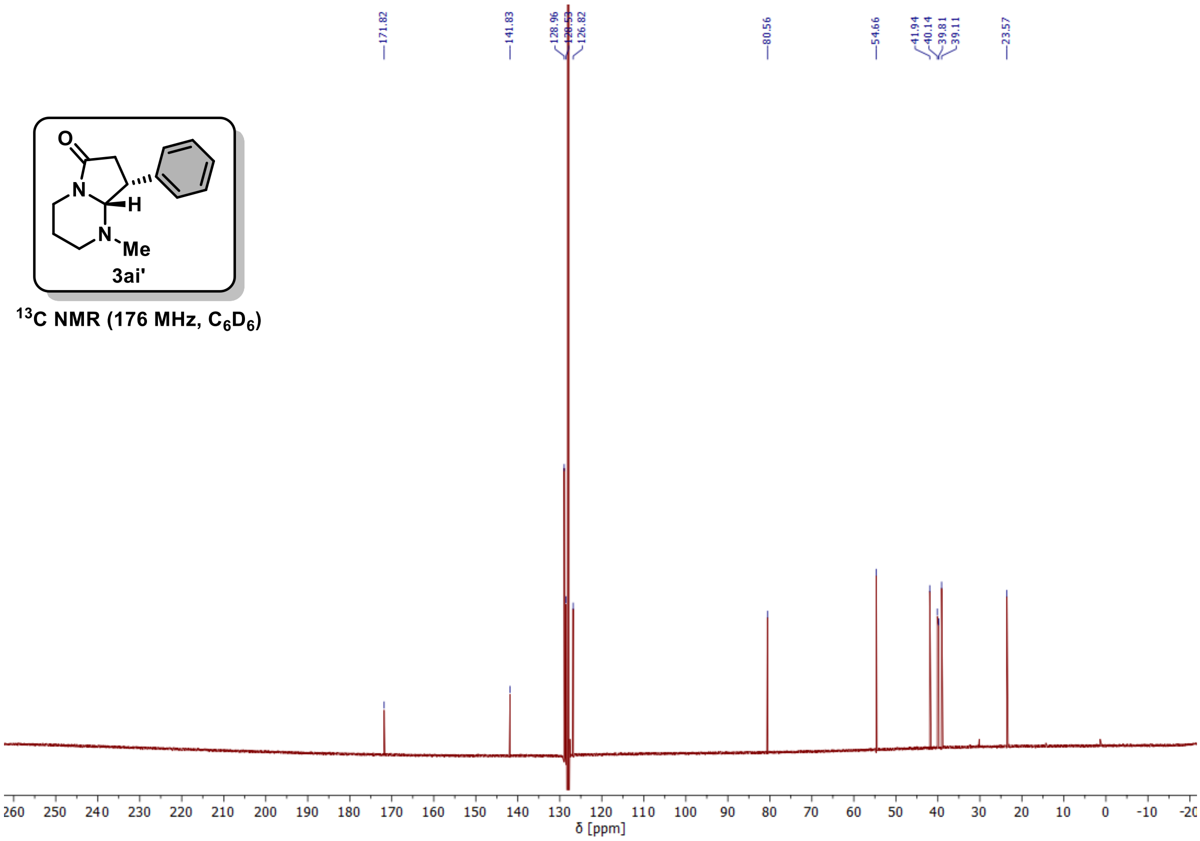

^{13}C NMR (176 MHz, C_6D_6)

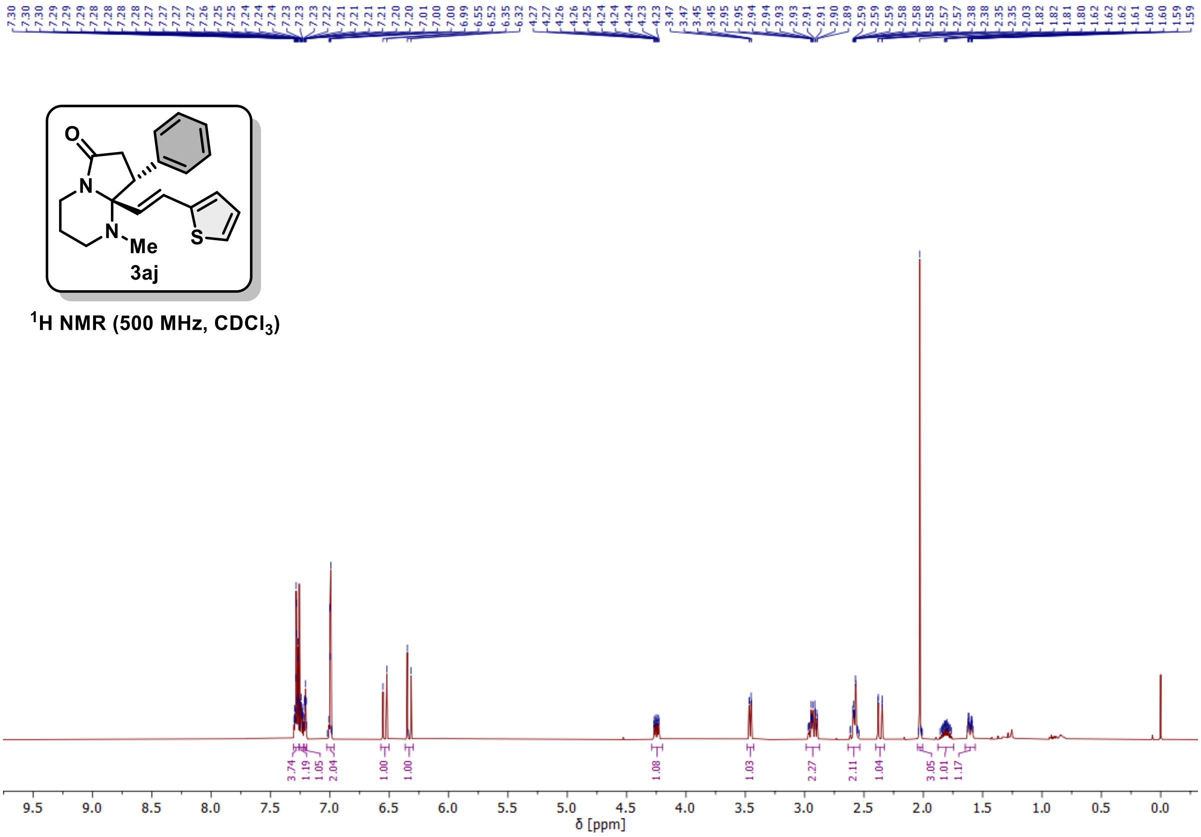
O
N
N
Me
S
3aj
^{1}H NMR (500 MHz, $CDCl_3$)
3.74
1.19
1.05
2.04
1.00
1.00
1.08
1.03
2.27
2.11
1.04
3.05
1.01
1.17
9.5 9.0 8.5 8.0 7.5 7.0 6.5 6.0 5.5 5.0 4.5 4.0 3.5 3.0 2.5 2.0 1.5 1.0 0.5 0.0
δ [ppm]

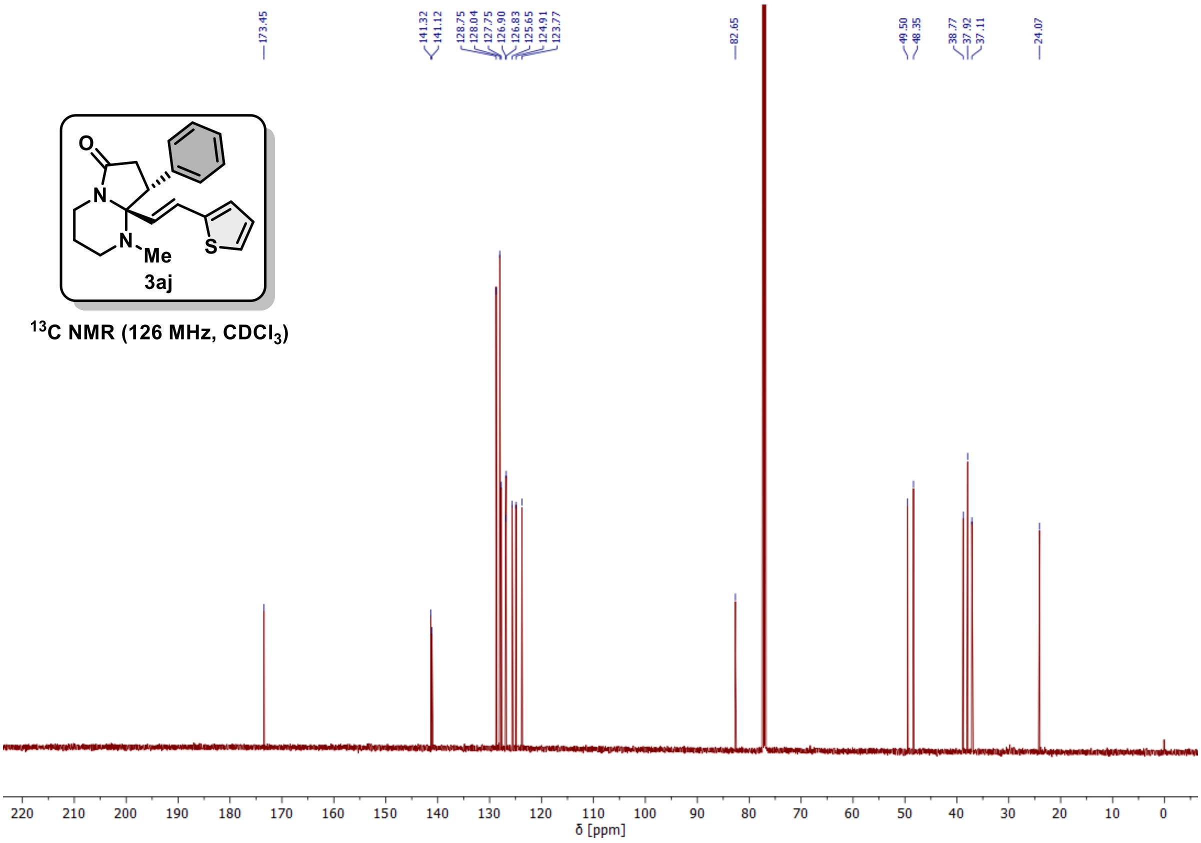
173.45
141.32
141.12
128.75
128.04
127.75
126.90
126.83
125.65
124.91
123.77
82.65
49.50
48.35
38.77
37.92
37.11
24.07
O
N
N
Me
S
3aj
^{13}C NMR (126 MHz, $CDCl_3$)
220 210 200 190 180 170 160 150 140 130 120 110 100 90 80 70 60 50 40 30 20 10 0
δ [ppm]

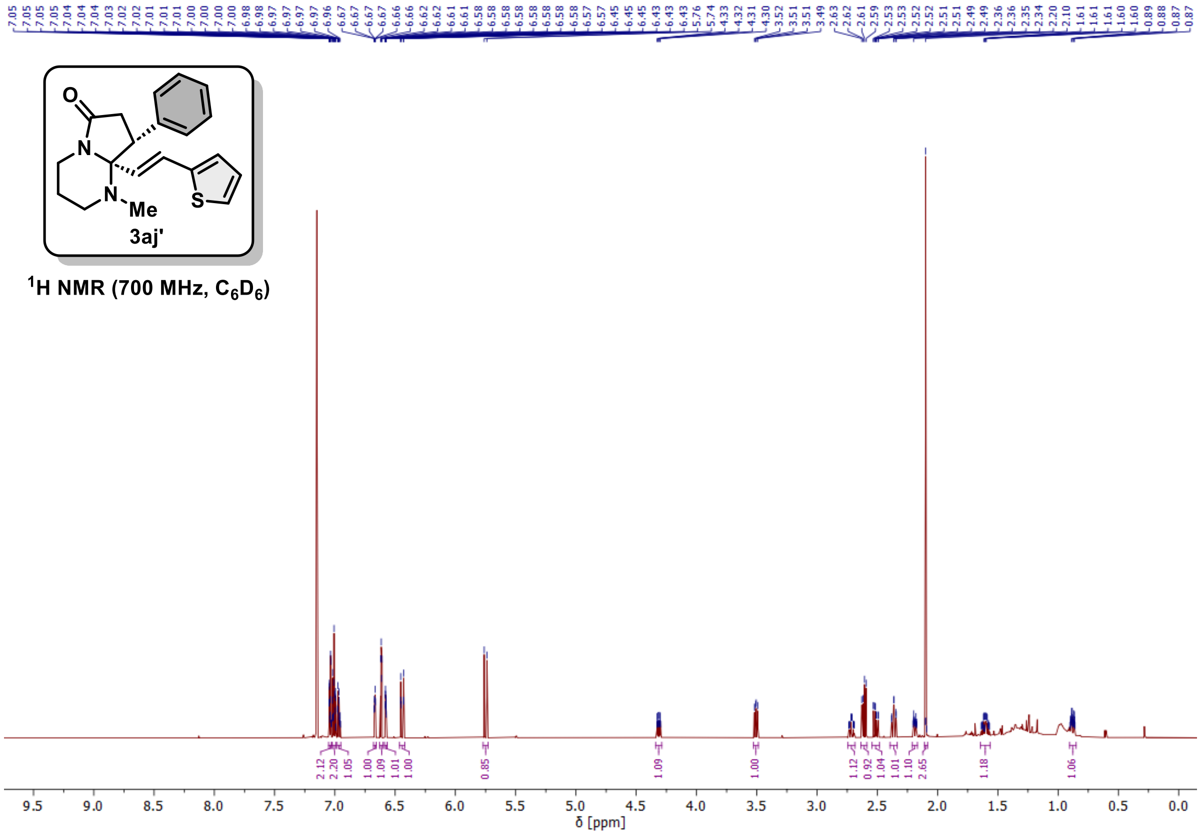
3aj'
^1H NMR (700 MHz, C_6D_6)
δ [ppm]

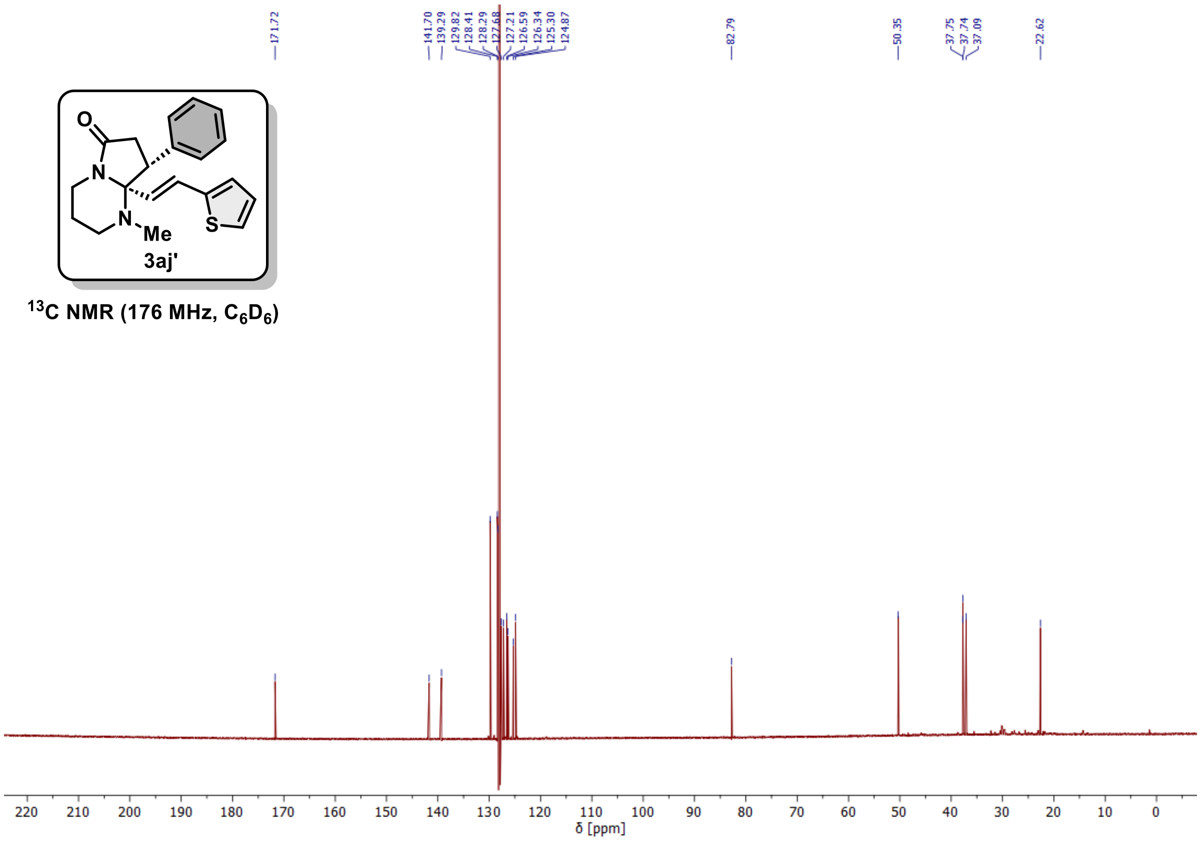
3aj'
^{13}C NMR (176 MHz, C_6D_6)
171.72
141.70
139.29
82.79
50.35
22.62
δ [ppm]

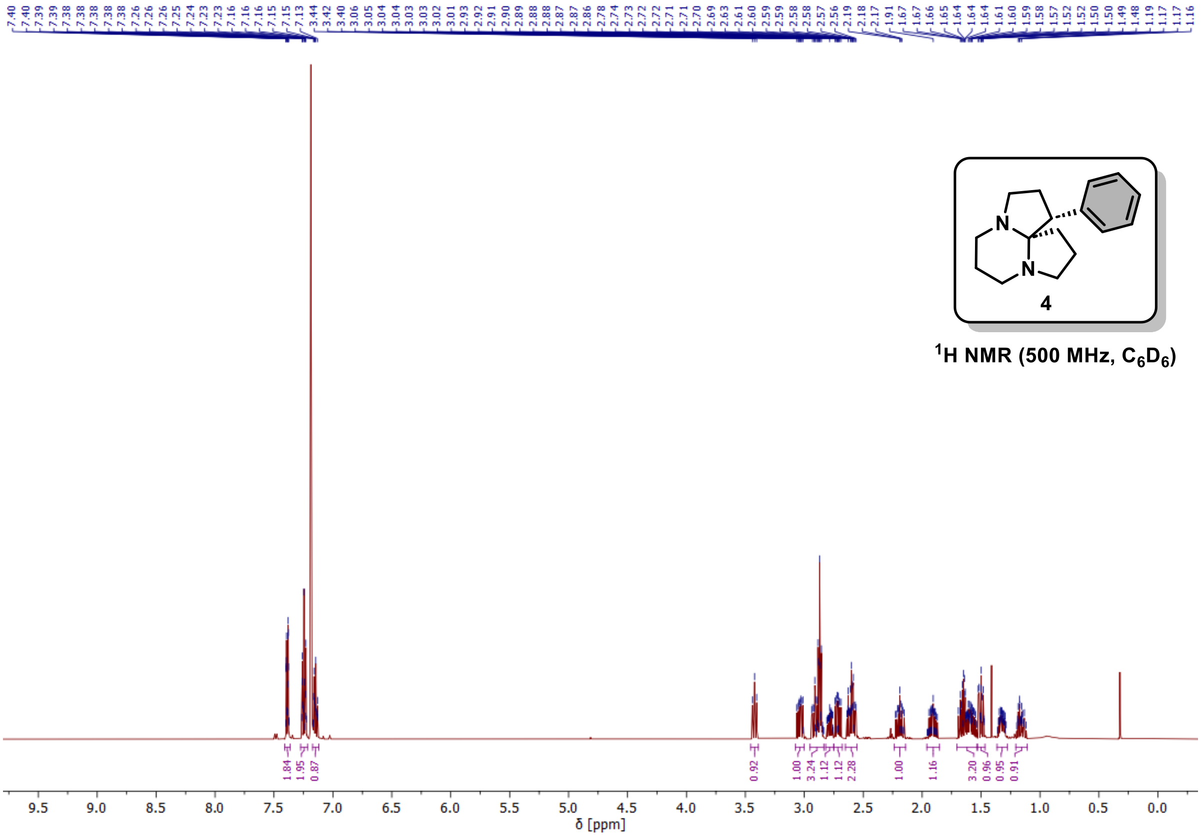

^{1}H NMR (500 MHz, C_6D_6)

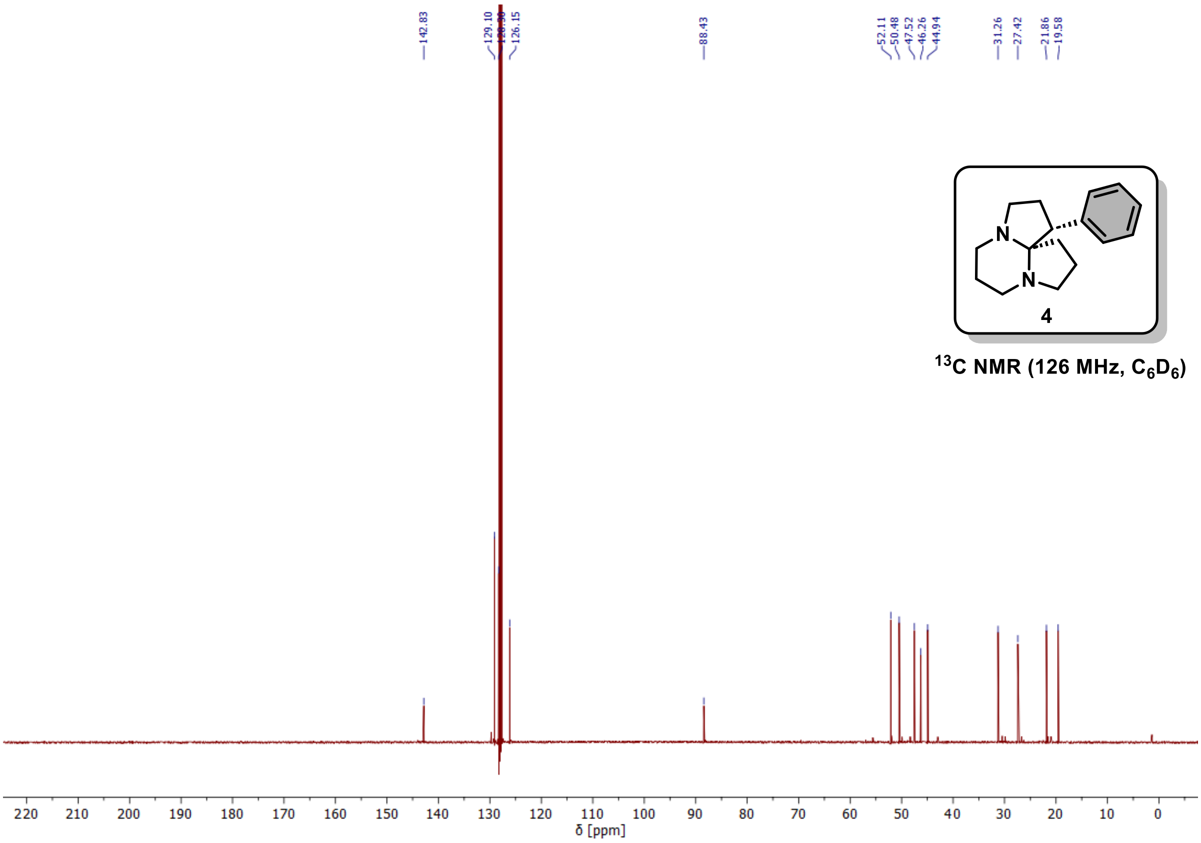

^{13}C NMR (126 MHz, C_6D_6)

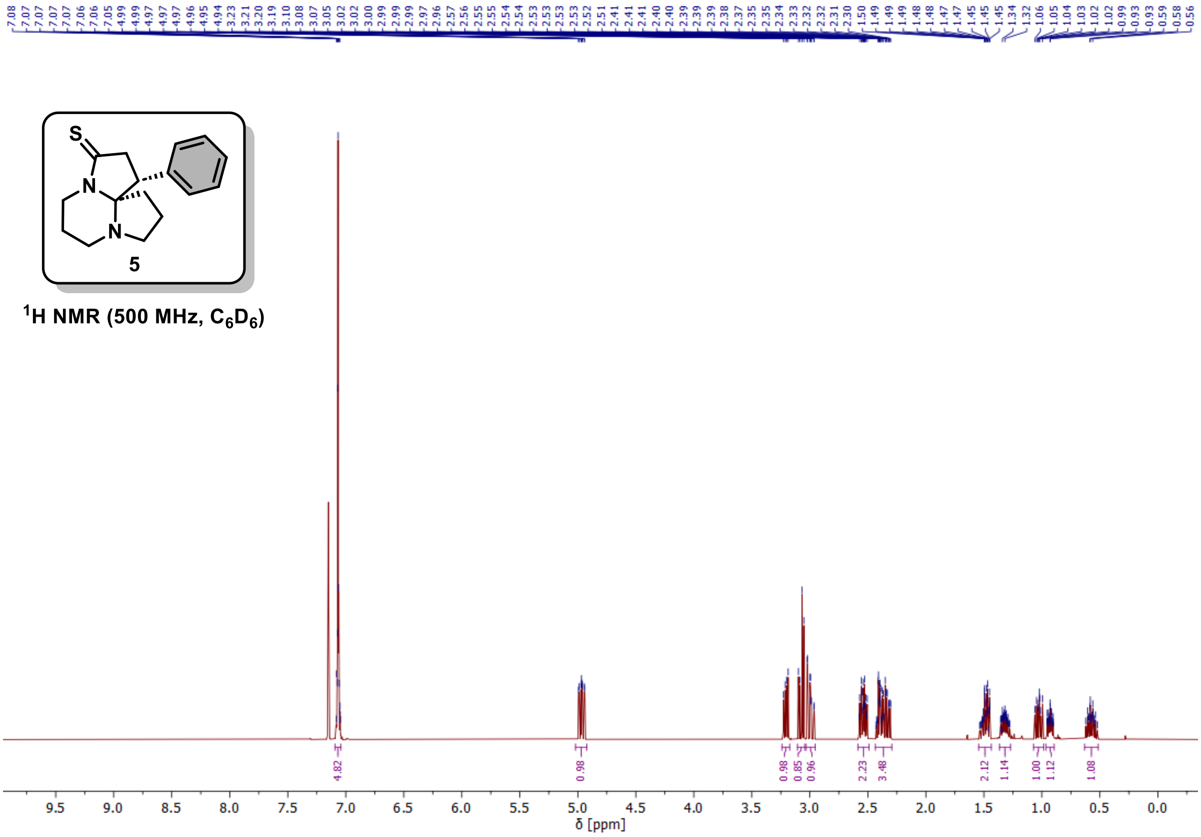
5
^{1}H NMR (500 MHz, C_6D_6)
δ [ppm]

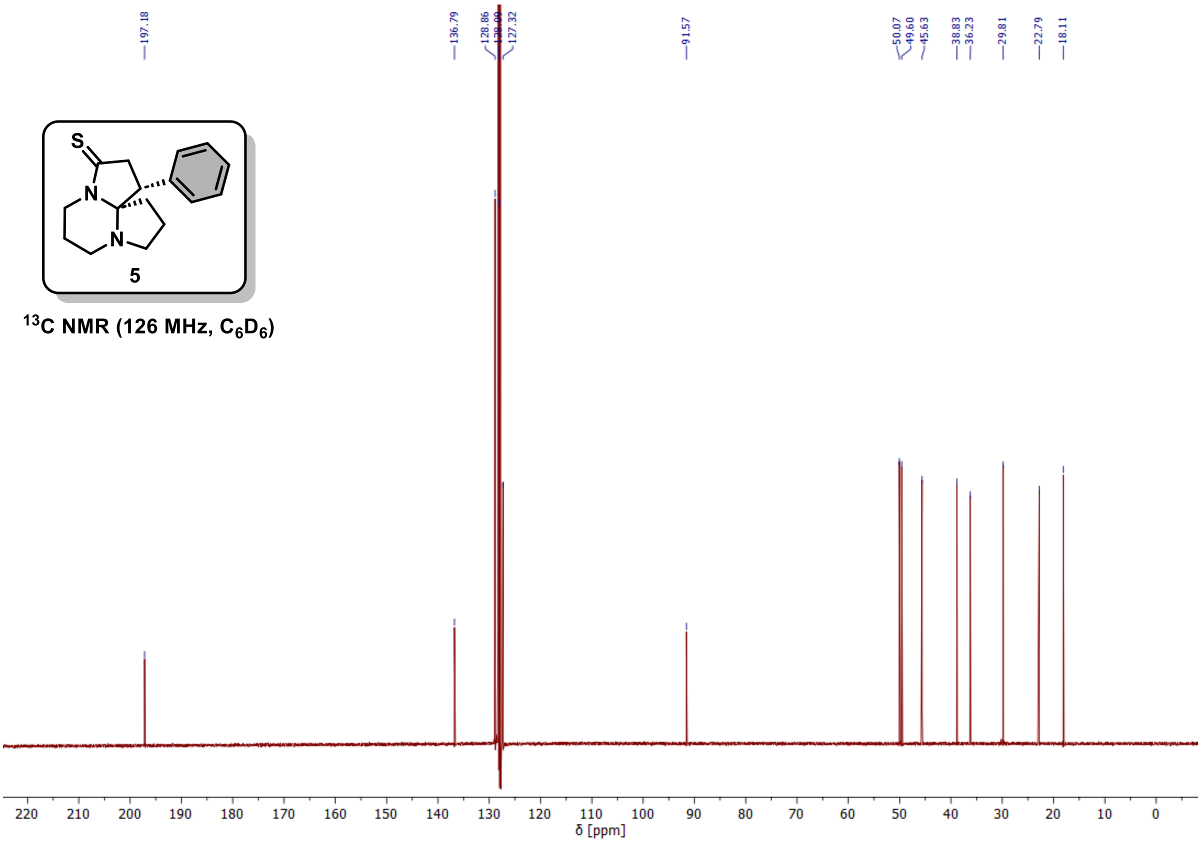
5
^{13}C NMR (126 MHz, C_6D_6)
δ [ppm]

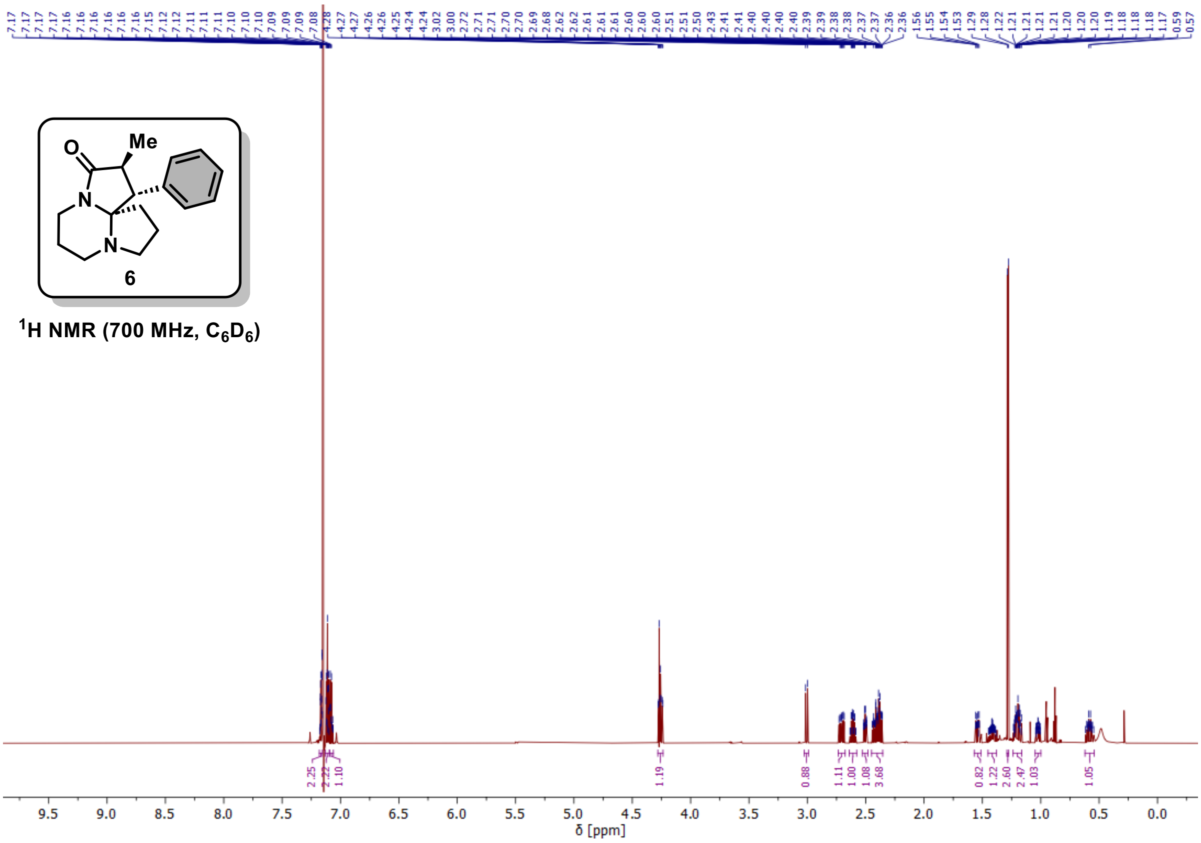

^{1}H NMR (700 MHz, C_6D_6)

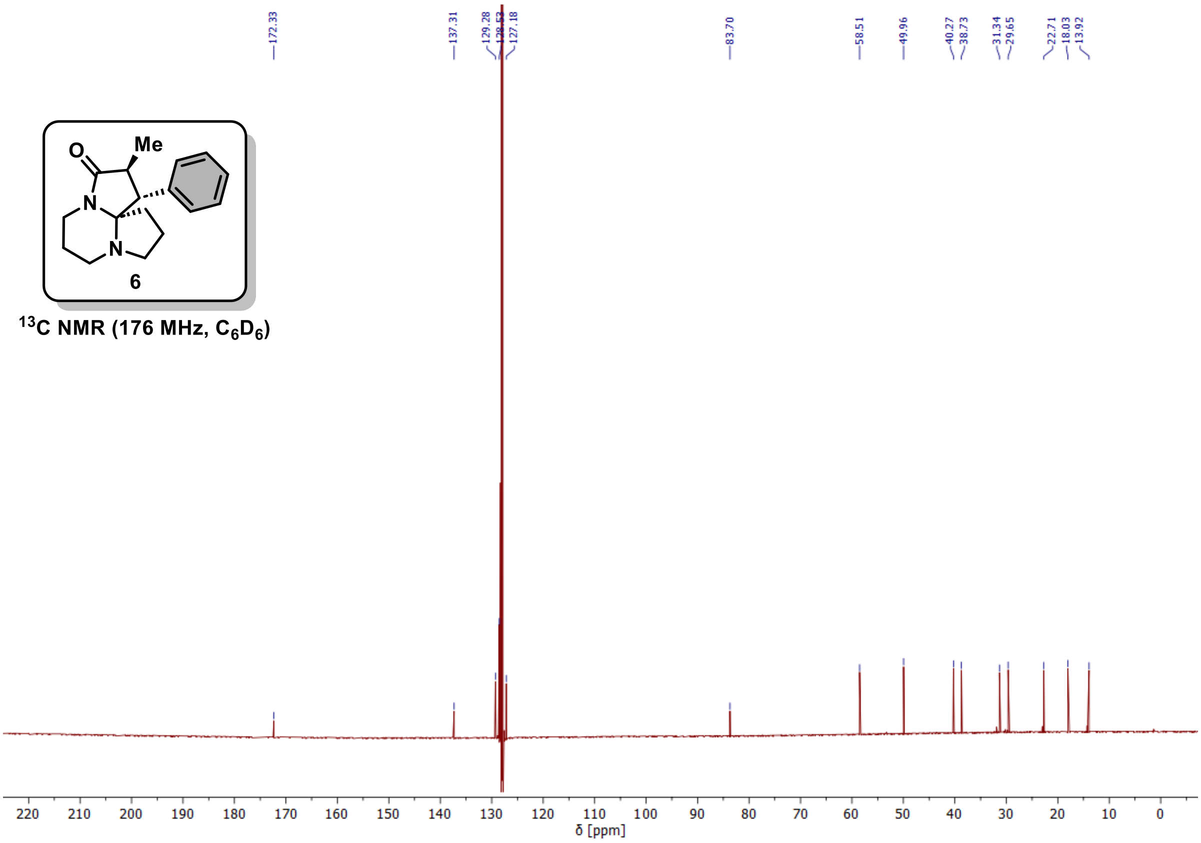

^{13}C NMR (176 MHz, C_6D_6)

8. X-Ray Data

11-(4-Fluorophenyl)hexahydro-1*H*,5*H*,9*H*-dipyrrolo[1,2-*a*:2',1'-*b*]pyrimidin-9-one (3ba)

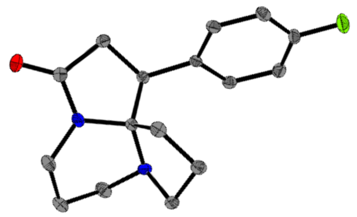

Figure 6: Structure of compound 3ba in the crystal. All atoms were drawn with anisotropic thermal ellipsoids at 50% probability level. H-atoms were omitted for clarity.

Crystallized by slowly removing the solvent. A colorless, block-shaped crystal was mounted on a MiTeGen micromount with perfluoroether oil. Data for Pna21_a were collected from a shock-cooled single crystal at 100(2) K on a Bruker APEX2 QUAZAR three-circle diffractometer with a microfocus sealed X-ray tube using a mirror optics as monochromator and a Bruker APEXII detector. The diffractometer was equipped with an Oxford Cryostream 800 low temperature device and used MoK_α radiation (λ = 0.71073 Å). All data were integrated with SAINT V8.40B and a multi-scan absorption correction using SADABS 2016/2 was applied.[28,29] The structure was solved by direct methods with SHELXT 2018/2 and refined by full-matrix least-squares methods against F^2 using SHELXL-2018/3.[30,31] All non-hydrogen atoms were refined with anisotropic displacement parameters. All hydrogen atoms were refined isotropic on calculated positions using a riding model with their U_{iso} values constrained to 1.5 times the U_{eq} of their pivot atoms for terminal sp^3 carbon atoms and 1.2 times for all other carbon atoms. Crystallographic data for the structures reported in this paper have been deposited with the Cambridge Crystallographic Data Centre.[32] CCDC 2457281 contain the supplementary crystallographic data for this paper. These data can be obtained free of charge from The Cambridge Crystallographic Data Centre via www.ccdc.cam.ac.uk/structures. This report and the CIF file were generated using FinalCif.[33]

Table 5: Crystal data and structure refinement for 3ba.

CCDC number	2457281
Empirical formula	$C_{16}H_{19}FN_2O$
Formula weight	274.33
Temperature [K]	100(2)
Crystal system	orthorhombic
Space group (number)	$Pna2_1$ (33)
a [Å]	15.496(8)
b [Å]	7.905(4)
c [Å]	22.785(11)
α [°]	90
β [°]	90
γ [°]	90
Volume [Å^3]	2791(2)
Z	8
ρ_{calc} [gcm^{-3}]	1.306
μ [mm^{-1}]	0.091
F(000)	1168
Crystal size [mm^3]	0.241×0.278×0.314
Crystal color	colorless
Crystal shape	block
Radiation	MoK_α (λ=0.71073 Å)
2θ range [°]	3.57 to 58.26 (0.73 Å)
Index ranges	−21 ≤ h ≤ 21<br>−10 ≤ k ≤ 10<br>−31 ≤ l ≤ 31
Reflections collected	195289
Independent reflections	7505<br>R_{int} = 0.0701<br>R_{sigma} = 0.0197
Completeness to θ = 25.242°	100.0 %
Data / Restraints / Parameters	7505 / 322 / 362
Absorption correction T_{min}/T_{max} (method)	0.6825 / 0.7461 (multi-scan)
Goodness-of-fit on F^2	1.043
Final *R* indexes [I≥2σ(I)]	R_1 = 0.0465<br>wR_2 = 0.1278
Final *R* indexes [all data]	R_1 = 0.0497<br>wR_2 = 0.1306
Largest peak/hole [eÅ^{-3}]	0.50/−0.22
Flack X parameter	0.0(10)

Refinement details for 3ba

Refined as a 2-component inversion twin.

Table 6: Atomic coordinates and Ueq [Å2] for 3ba.

Atom	*x*	*y*	*z*	U_{eq}
O1	−0.12520(13)	0.5838(3)	0.47477(9)	0.0208(4)
O2	0.15266(12)	0.4207(3)	0.53664(9)	0.0191(4)
N1	0.06545(14)	0.7371(3)	0.32833(10)	0.0150(4)
N2	−0.01868(14)	0.7014(3)	0.41760(10)	0.0168(4)
N3	0.35255(13)	0.2505(3)	0.67186(9)	0.0136(4)
N4	0.26580(14)	0.2938(3)	0.58457(10)	0.0156(4)
C1	−0.0504(2)	0.8754(4)	0.41529(13)	0.0232(6)
H1A	−0.011231	0.949701	0.437988	0.028
H1B	−0.108350	0.881261	0.433397	0.028
C2	−0.05531(19)	0.9381(4)	0.35125(13)	0.0221(5)
H2A	−0.022879	1.045431	0.347425	0.027
H2B	−0.116244	0.960423	0.340747	0.027
C3	−0.01730(17)	0.8054(3)	0.30878(12)	0.0187(5)
H3A	−0.058932	0.711226	0.304485	0.022
H3B	−0.009648	0.857984	0.269694	0.022
C4	0.13860(16)	0.8557(3)	0.32837(12)	0.0181(5)
H4A	0.121495	0.967561	0.344053	0.022
H4B	0.162622	0.870240	0.288428	0.022
C5	0.20297(17)	0.7679(3)	0.36888(12)	0.0188(5)
H5A	0.246910	0.847862	0.383869	0.023
H5B	0.232113	0.672365	0.348947	0.023
C6	0.14315(17)	0.7061(3)	0.41797(12)	0.0187(5)
H6A	0.131241	0.797788	0.446432	0.022
H6B	0.168910	0.608957	0.439062	0.022
C7	0.06016(16)	0.6530(3)	0.38560(11)	0.0137(4)
C8	0.04545(15)	0.4588(3)	0.37869(11)	0.0127(4)
H8	0.006086	0.443124	0.344344	0.015
C9	−0.00662(16)	0.4132(3)	0.43371(11)	0.0149(4)
H9A	−0.044681	0.314852	0.426402	0.018
H9B	0.031933	0.387361	0.467172	0.018
C10	−0.05913(17)	0.5725(3)	0.44524(11)	0.0154(5)
C11	0.23443(19)	0.1201(3)	0.58540(13)	0.0210(5)
H11A	0.273370	0.047367	0.561949	0.025
H11B	0.176165	0.115053	0.567666	0.025
C12	0.23073(19)	0.0547(4)	0.64942(13)	0.0230(6)
H12A	0.169915	0.033184	0.660445	0.028
H12B	0.262562	−0.053545	0.652265	0.028
C13	0.27016(17)	0.1831(4)	0.69220(13)	0.0186(5)
H13A	0.229152	0.277735	0.697767	0.022
H13B	0.278719	0.127839	0.730761	0.022
C14	0.42542(16)	0.1324(3)	0.67048(13)	0.0172(5)
H14A	0.407672	0.020527	0.655126	0.021
H14B	0.450959	0.117799	0.709988	0.021
C15	0.48840(17)	0.2200(3)	0.62880(12)	0.0189(5)
H15A	0.531248	0.139490	0.612729	0.023
H15B	0.518919	0.314453	0.648383	0.023

C16	0.42714(17)	0.2840(3)	0.58118(11)	0.0170(5)
H16A	0.413468	0.193275	0.552725	0.020
H16B	0.452580	0.381073	0.559849	0.020
C17	0.34621(15)	0.3381(3)	0.61524(11)	0.0127(4)
C18	0.33315(15)	0.5320(3)	0.62385(11)	0.0124(4)
H18	0.296254	0.545930	0.659535	0.015
C19	0.27757(16)	0.5837(3)	0.57136(11)	0.0158(5)
H19A	0.241192	0.683062	0.580733	0.019
H19B	0.313545	0.609617	0.536596	0.019
C20	0.22323(16)	0.4271(3)	0.56153(11)	0.0146(4)
F1_1	0.35354(10)	0.1134(3)	0.32389(8)	0.0267(4)
C1_1	0.27900(16)	0.1958(3)	0.33776(13)	0.0184(5)
C2_1	0.25306(17)	0.1989(3)	0.39593(12)	0.0178(5)
H2_1	0.286587	0.146759	0.425785	0.021
C3_1	0.17570(17)	0.2816(3)	0.40902(11)	0.0162(5)
H3_1	0.156164	0.284508	0.448526	0.019
C4_1	0.12654(16)	0.3597(3)	0.36567(11)	0.0131(4)
C5_1	0.15586(16)	0.3520(3)	0.30755(11)	0.0150(5)
H5_1	0.122827	0.404063	0.277419	0.018
C6_1	0.23254(17)	0.2695(3)	0.29297(12)	0.0182(5)
H6_1	0.252069	0.264332	0.253492	0.022
F1_2	0.64269(10)	0.8781(2)	0.67387(8)	0.0248(4)
C1_2	0.56814(16)	0.7932(3)	0.66098(12)	0.0165(5)
C2_2	0.52296(17)	0.7205(3)	0.70652(12)	0.0163(5)
H2_2	0.543557	0.726260	0.745734	0.020
C3_2	0.44612(16)	0.6383(3)	0.69305(11)	0.0144(5)
H3_2	0.413759	0.587308	0.723738	0.017
C4_2	0.41529(15)	0.6287(3)	0.63544(11)	0.0124(4)
C5_2	0.46374(17)	0.7062(3)	0.59125(11)	0.0157(5)
H5_2	0.443318	0.702470	0.551958	0.019
C6_2	0.54126(17)	0.7889(3)	0.60326(12)	0.0173(5)
H6_2	0.574213	0.840174	0.572936	0.021

U_{eq} is defined as 1/3 of the trace of the orthogonalized U_{ij} tensor.

Table 7: Anisotropic displacement parameters [Å2] for 3ba. The anisotropic displacement factor exponent takes the form: −2π2[h2(a*)2U11 + k2(b*)2U22 + … + 2hka*b*U12].

Atom	U_{11}	U_{22}	U_{33}	U_{23}	U_{13}	U_{12}
O1	0.0192(9)	0.0238(9)	0.0193(9)	−0.0033(7)	0.0048(7)	0.0002(8)
O2	0.0154(8)	0.0251(10)	0.0168(8)	−0.0031(7)	−0.0031(7)	0.0001(7)
N1	0.0145(10)	0.0138(9)	0.0166(10)	0.0032(8)	0.0000(8)	0.0010(7)
N2	0.0183(10)	0.0133(10)	0.0187(11)	−0.0016(8)	0.0032(8)	0.0025(8)
N3	0.0141(10)	0.0119(9)	0.0147(9)	0.0026(8)	0.0010(7)	0.0012(7)
N4	0.0166(10)	0.0140(10)	0.0160(10)	−0.0017(8)	−0.0024(8)	−0.0032(8)
C1	0.0290(14)	0.0153(12)	0.0252(14)	−0.0022(10)	0.0036(11)	0.0062(11)
C2	0.0204(12)	0.0178(12)	0.0282(14)	0.0046(10)	0.0022(11)	0.0051(10)
C3	0.0186(12)	0.0178(12)	0.0195(12)	0.0036(9)	−0.0023(9)	0.0014(9)
C4	0.0151(11)	0.0167(12)	0.0225(13)	0.0024(10)	0.0007(10)	−0.0011(9)
C5	0.0146(11)	0.0156(11)	0.0263(13)	0.0006(10)	−0.0019(10)	−0.0004(9)
C6	0.0225(12)	0.0150(12)	0.0186(12)	0.0000(9)	−0.0051(10)	−0.0027(9)
C7	0.0155(11)	0.0132(11)	0.0123(10)	−0.0001(8)	0.0012(8)	0.0009(8)
C8	0.0137(10)	0.0114(10)	0.0132(10)	−0.0001(8)	−0.0007(8)	−0.0011(8)

C9	0.0149(10)	0.0129(11)	0.0169(11)	0.0010(9)	0.0018(9)	−0.0012(9)
C10	0.0173(11)	0.0169(11)	0.0121(10)	−0.0015(9)	−0.0022(9)	−0.0002(9)
C11	0.0230(13)	0.0157(12)	0.0244(13)	−0.0029(10)	−0.0037(11)	−0.0052(10)
C12	0.0222(13)	0.0167(12)	0.0300(15)	0.0036(10)	−0.0013(11)	−0.0071(10)
C13	0.0185(12)	0.0166(11)	0.0208(12)	−0.0008(10)	0.0049(10)	−0.0023(9)
C14	0.0148(11)	0.0139(10)	0.0230(12)	0.0035(9)	−0.0003(9)	0.0016(9)
C15	0.0154(11)	0.0152(11)	0.0260(13)	0.0017(10)	0.0028(10)	0.0023(9)
C16	0.0177(12)	0.0170(11)	0.0164(11)	−0.0020(9)	0.0055(9)	0.0016(9)
C17	0.0123(10)	0.0114(10)	0.0143(11)	0.0011(8)	−0.0001(8)	−0.0011(8)
C18	0.0118(10)	0.0129(10)	0.0125(10)	0.0001(8)	0.0008(8)	−0.0008(8)
C19	0.0159(11)	0.0149(11)	0.0165(12)	0.0023(8)	−0.0005(9)	0.0003(9)
C20	0.0153(10)	0.0166(11)	0.0120(10)	−0.0011(9)	0.0015(9)	−0.0003(8)
F1_1	0.0152(7)	0.0335(10)	0.0315(9)	0.0011(7)	0.0036(7)	0.0096(7)
C1_1	0.0123(10)	0.0160(11)	0.0269(13)	−0.0022(10)	0.0010(9)	0.0012(9)
C2_1	0.0146(11)	0.0164(12)	0.0224(13)	0.0005(9)	−0.0060(9)	0.0015(9)
C3_1	0.0170(11)	0.0173(12)	0.0142(11)	−0.0028(9)	−0.0031(9)	0.0012(9)
C4_1	0.0144(10)	0.0108(10)	0.0141(10)	−0.0010(8)	−0.0007(8)	−0.0013(8)
C5_1	0.0170(11)	0.0133(11)	0.0148(11)	0.0012(9)	−0.0018(9)	0.0006(8)
C6_1	0.0182(12)	0.0188(12)	0.0176(12)	−0.0003(9)	0.0038(9)	−0.0012(9)
F1_2	0.0158(8)	0.0305(9)	0.0282(9)	0.0011(7)	−0.0026(6)	−0.0087(6)
C1_2	0.0114(10)	0.0145(10)	0.0235(12)	−0.0010(9)	−0.0010(9)	−0.0006(9)
C2_2	0.0167(11)	0.0165(11)	0.0157(11)	−0.0003(9)	−0.0024(9)	0.0004(9)
C3_2	0.0140(10)	0.0139(11)	0.0151(11)	0.0022(9)	0.0017(9)	0.0011(8)
C4_2	0.0122(10)	0.0099(10)	0.0152(11)	0.0004(8)	0.0006(8)	0.0003(8)
C5_2	0.0179(12)	0.0139(11)	0.0152(11)	0.0022(9)	0.0005(9)	−0.0015(9)
C6_2	0.0160(11)	0.0160(12)	0.0200(12)	0.0008(9)	0.0032(9)	−0.0022(9)

Table 8: Bond lengths and angles for 3ba.

Atom–Atom	Length [Å]
O1–C10	1.228(3)
O2–C20	1.233(3)
N1–C3	1.461(3)
N1–C7	1.467(3)
N1–C4	1.471(3)
N2–C10	1.352(3)
N2–C1	1.461(4)
N2–C7	1.473(3)
N3–C13	1.459(3)
N3–C14	1.466(3)
N3–C17	1.467(3)
N4–C20	1.350(3)
N4–C11	1.457(3)
N4–C17	1.471(3)
C1–C2	1.543(4)
C1–H1A	0.9900
C1–H1B	0.9900
C2–C3	1.544(4)
C2–H2A	0.9900
C2–H2B	0.9900
C3–H3A	0.9900
C3–H3B	0.9900
C4–C5	1.526(4)
C4–H4A	0.9900
C4–H4B	0.9900
C5–C6	1.533(4)
C5–H5A	0.9900
C5–H5B	0.9900
C6–C7	1.541(4)
C6–H6A	0.9900
C6–H6B	0.9900
C7–C8	1.560(4)
C8–C4_1	1.510(3)
C8–C9	1.534(3)
C8–H8	1.0000
C9–C10	1.522(4)
C9–H9A	0.9900
C9–H9B	0.9900
C11–C12	1.549(4)
C11–H11A	0.9900
C11–H11B	0.9900
C12–C13	1.534(4)
C12–H12A	0.9900
C12–H12B	0.9900
C13–H13A	0.9900
C13–H13B	0.9900
C14–C15	1.528(4)
C14–H14A	0.9900

C14–H14B	0.9900
C15–C16	1.528(4)
C15–H15A	0.9900
C15–H15B	0.9900
C16–C17	1.535(3)
C16–H16A	0.9900
C16–H16B	0.9900
C17–C18	1.559(4)
C18–C4_2	1.508(3)
C18–C19	1.529(3)
C18–H18	1.0000
C19–C20	1.514(4)
C19–H19A	0.9900
C19–H19B	0.9900
F1_1–C1_1	1.363(3)
C1_1–C6_1	1.378(4)
C1_1–C2_1	1.385(4)
C2_1–C3_1	1.398(4)
C2_1–H2_1	0.9500
C3_1–C4_1	1.392(3)
C3_1–H3_1	0.9500
C4_1–C5_1	1.402(3)
C5_1–C6_1	1.396(4)
C5_1–H5_1	0.9500
C6_1–H6_1	0.9500
F1_2–C1_2	1.368(3)
C1_2–C2_2	1.377(4)
C1_2–C6_2	1.380(4)
C2_2–C3_2	1.391(3)
C2_2–H2_2	0.9500
C3_2–C4_2	1.399(3)
C3_2–H3_2	0.9500
C4_2–C5_2	1.397(3)
C5_2–C6_2	1.395(4)
C5_2–H5_2	0.9500
C6_2–H6_2	0.9500

**Atom–Atom–Atom**	**Angle [°]**
C3–N1–C7	112.9(2)
C3–N1–C4	116.2(2)
C7–N1–C4	109.4(2)
C10–N2–C1	124.8(2)
C10–N2–C7	114.8(2)
C1–N2–C7	120.4(2)
C13–N3–C14	116.6(2)
C13–N3–C17	113.1(2)
C14–N3–C17	109.5(2)
C20–N4–C11	125.3(2)
C20–N4–C17	114.4(2)
C11–N4–C17	120.1(2)
N2–C1–C2	110.7(2)
N2–C1–H1A	109.5
C2–C1–H1A	109.5
N2–C1–H1B	109.5
C2–C1–H1B	109.5
H1A–C1–H1B	108.1
C1–C2–C3	110.8(2)
C1–C2–H2A	109.5
C3–C2–H2A	109.5
C1–C2–H2B	109.5
C3–C2–H2B	109.5
H2A–C2–H2B	108.1
N1–C3–C2	113.3(2)
N1–C3–H3A	108.9
C2–C3–H3A	108.9
N1–C3–H3B	108.9
C2–C3–H3B	108.9
H3A–C3–H3B	107.7
N1–C4–C5	102.4(2)
N1–C4–H4A	111.3
C5–C4–H4A	111.3
N1–C4–H4B	111.3
C5–C4–H4B	111.3
H4A–C4–H4B	109.2
C4–C5–C6	101.0(2)
C4–C5–H5A	111.6
C6–C5–H5A	111.6
C4–C5–H5B	111.6
C6–C5–H5B	111.6
H5A–C5–H5B	109.4
C5–C6–C7	104.0(2)
C5–C6–H6A	111.0
C7–C6–H6A	111.0
C5–C6–H6B	111.0
C7–C6–H6B	111.0
H6A–C6–H6B	109.0
N1–C7–N2	111.6(2)
N1–C7–C6	104.8(2)
N2–C7–C6	112.6(2)
N1–C7–C8	111.4(2)
N2–C7–C8	100.63(19)
C6–C7–C8	116.0(2)
C4_1–C8–C9	118.5(2)
C4_1–C8–C7	114.1(2)
C9–C8–C7	103.04(19)
C4_1–C8–H8	106.8
C9–C8–H8	106.8
C7–C8–H8	106.8
C10–C9–C8	103.2(2)
C10–C9–H9A	111.1
C8–C9–H9A	111.1
C10–C9–H9B	111.1
C8–C9–H9B	111.1
H9A–C9–H9B	109.1
O1–C10–N2	126.0(2)
O1–C10–C9	126.9(2)
N2–C10–C9	107.2(2)
N4–C11–C12	109.8(2)

N4–C11–H11A	109.7
C12–C11–H11A	109.7
N4–C11–H11B	109.7
C12–C11–H11B	109.7
H11A–C11–H11B	108.2
C13–C12–C11	111.3(2)
C13–C12–H12A	109.4
C11–C12–H12A	109.4
C13–C12–H12B	109.4
C11–C12–H12B	109.4
H12A–C12–H12B	108.0
N3–C13–C12	112.9(2)
N3–C13–H13A	109.0
C12–C13–H13A	109.0
N3–C13–H13B	109.0
C12–C13–H13B	109.0
H13A–C13–H13B	107.8
N3–C14–C15	102.5(2)
N3–C14–H14A	111.3
C15–C14–H14A	111.3
N3–C14–H14B	111.3
C15–C14–H14B	111.3
H14A–C14–H14B	109.2
C14–C15–C16	101.2(2)
C14–C15–H15A	111.5
C16–C15–H15A	111.5
C14–C15–H15B	111.5
C16–C15–H15B	111.5
H15A–C15–H15B	109.3
C15–C16–C17	103.9(2)
C15–C16–H16A	111.0
C17–C16–H16A	111.0
C15–C16–H16B	111.0
C17–C16–H16B	111.0
H16A–C16–H16B	109.0
N3–C17–N4	111.2(2)
N3–C17–C16	105.0(2)
N4–C17–C16	112.7(2)
N3–C17–C18	111.2(2)
N4–C17–C18	100.60(19)
C16–C17–C18	116.3(2)
C4_2–C18–C19	118.5(2)
C4_2–C18–C17	114.3(2)
C19–C18–C17	103.7(2)
C4_2–C18–H18	106.5
C19–C18–H18	106.5
C17–C18–H18	106.5
C20–C19–C18	102.2(2)
C20–C19–H19A	111.3
C18–C19–H19A	111.3
C20–C19–H19B	111.3
C18–C19–H19B	111.3
H19A–C19–H19B	109.2
O2–C20–N4	125.4(2)
O2–C20–C19	126.5(2)
N4–C20–C19	108.0(2)
F1_1–C1_1–C6_1	118.2(2)
F1_1–C1_1–C2_1	118.4(2)
C6_1–C1_1–C2_1	123.3(2)
C1_1–C2_1–C3_1	117.5(2)
C1_1–C2_1–H2_1	121.3
C3_1–C2_1–H2_1	121.3
C4_1–C3_1–C2_1	121.7(2)
C4_1–C3_1–H3_1	119.1
C2_1–C3_1–H3_1	119.1
C3_1–C4_1–C5_1	118.3(2)
C3_1–C4_1–C8	123.1(2)
C5_1–C4_1–C8	118.6(2)
C6_1–C5_1–C4_1	121.4(2)
C6_1–C5_1–H5_1	119.3
C4_1–C5_1–H5_1	119.3
C1_1–C6_1–C5_1	117.8(2)
C1_1–C6_1–H6_1	121.1
C5_1–C6_1–H6_1	121.1
F1_2–C1_2–C2_2	118.2(2)
F1_2–C1_2–C6_2	118.1(2)
C2_2–C1_2–C6_2	123.6(2)
C1_2–C2_2–C3_2	117.6(2)
C1_2–C2_2–H2_2	121.2
C3_2–C2_2–H2_2	121.2
C2_2–C3_2–C4_2	121.6(2)
C2_2–C3_2–H3_2	119.2
C4_2–C3_2–H3_2	119.2
C5_2–C4_2–C3_2	118.0(2)
C5_2–C4_2–C18	123.3(2)
C3_2–C4_2–C18	118.7(2)
C6_2–C5_2–C4_2	121.8(2)
C6_2–C5_2–H5_2	119.1
C4_2–C5_2–H5_2	119.1
C1_2–C6_2–C5_2	117.3(2)
C1_2–C6_2–H6_2	121.4
C5_2–C6_2–H6_2	121.4

Table 9: Torsion angles for 3ba.

Atom–Atom–Atom–Atom	Torsion Angle [°]
C10–N2–C1–C2	−126.6(3)
C7–N2–C1–C2	49.7(3)
N2–C1–C2–C3	−5.4(3)
C7–N1–C3–C2	59.9(3)
C4–N1–C3–C2	−67.6(3)
C1–C2–C3–N1	−46.5(3)
C3–N1–C4–C5	161.6(2)
C7–N1–C4–C5	32.3(3)

N1–C4–C5–C6	−42.3(3)	C20–N4–C17–C16	−108.1(2)
C4–C5–C6–C7	37.3(3)	C11–N4–C17–C16	77.3(3)
C3–N1–C7–N2	−17.4(3)	C20–N4–C17–C18	16.5(3)
C4–N1–C7–N2	113.6(2)	C11–N4–C17–C18	−158.2(2)
C3–N1–C7–C6	−139.6(2)	C15–C16–C17–N3	−19.4(3)
C4–N1–C7–C6	−8.6(3)	C15–C16–C17–N4	−140.6(2)
C3–N1–C7–C8	94.2(2)	C15–C16–C17–C18	104.0(2)
C4–N1–C7–C8	−134.8(2)	N3–C17–C18–C4_2	81.9(3)
C10–N2–C7–N1	138.0(2)	N4–C17–C18–C4_2	−160.2(2)
C1–N2–C7–N1	−38.7(3)	C16–C17–C18–C4_2	−38.2(3)
C10–N2–C7–C6	−104.4(3)	N3–C17–C18–C19	−147.7(2)
C1–N2–C7–C6	78.9(3)	N4–C17–C18–C19	−29.8(2)
C10–N2–C7–C8	19.8(3)	C16–C17–C18–C19	92.2(2)
C1–N2–C7–C8	−156.9(2)	C4_2–C18–C19–C20	160.4(2)
C5–C6–C7–N1	−18.5(3)	C17–C18–C19–C20	32.5(2)
C5–C6–C7–N2	−140.1(2)	C11–N4–C20–O2	−1.8(4)
C5–C6–C7–C8	104.8(2)	C17–N4–C20–O2	−176.2(2)
N1–C7–C8–C4_1	80.8(3)	C11–N4–C20–C19	178.5(2)
N2–C7–C8–C4_1	−160.7(2)	C17–N4–C20–C19	4.2(3)
C6–C7–C8–C4_1	−38.9(3)	C18–C19–C20–O2	156.9(2)
N1–C7–C8–C9	−149.4(2)	C18–C19–C20–N4	−23.4(3)
N2–C7–C8–C9	−30.9(2)	F1_1–C1_1–C2_1–C3_1	−178.9(2)
C6–C7–C8–C9	90.8(2)	C6_1–C1_1–C2_1–C3_1	0.0(4)
C4_1–C8–C9–C10	159.0(2)	C1_1–C2_1–C3_1–C4_1	−0.6(4)
C7–C8–C9–C10	31.9(2)	C2_1–C3_1–C4_1–C5_1	0.9(4)
C1–N2–C10–O1	−4.1(4)	C2_1–C3_1–C4_1–C8	−176.3(2)
C7–N2–C10–O1	179.4(2)	C9–C8–C4_1–C3_1	−26.9(3)
C1–N2–C10–C9	176.9(2)	C7–C8–C4_1–C3_1	94.7(3)
C7–N2–C10–C9	0.4(3)	C9–C8–C4_1–C5_1	155.9(2)
C8–C9–C10–O1	160.1(2)	C7–C8–C4_1–C5_1	−82.5(3)
C8–C9–C10–N2	−20.9(3)	C3_1–C4_1–C5_1–C6_1	−0.4(4)
C20–N4–C11–C12	−122.5(3)	C8–C4_1–C5_1–C6_1	176.9(2)
C17–N4–C11–C12	51.5(3)	F1_1–C1_1–C6_1–C5_1	179.3(2)
N4–C11–C12–C13	−6.3(3)	C2_1–C1_1–C6_1–C5_1	0.4(4)
C14–N3–C13–C12	−68.5(3)	C4_1–C5_1–C6_1–C1_1	−0.2(4)
C17–N3–C13–C12	59.8(3)	F1_2–C1_2–C2_2–C3_2	178.4(2)
C11–C12–C13–N3	−46.2(3)	C6_2–C1_2–C2_2–C3_2	0.1(4)
C13–N3–C14–C15	160.9(2)	C1_2–C2_2–C3_2–C4_2	0.1(4)
C17–N3–C14–C15	30.9(3)	C2_2–C3_2–C4_2–C5_2	−0.6(4)
N3–C14–C15–C16	−41.5(2)	C2_2–C3_2–C4_2–C18	177.8(2)
C14–C15–C16–C17	37.4(3)	C19–C18–C4_2–C5_2	−29.4(3)
C13–N3–C17–N4	−17.0(3)	C17–C18–C4_2–C5_2	93.4(3)
C14–N3–C17–N4	114.9(2)	C19–C18–C4_2–C3_2	152.3(2)
C13–N3–C17–C16	−139.1(2)	C17–C18–C4_2–C3_2	−84.9(3)
C14–N3–C17–C16	−7.3(3)	C3_2–C4_2–C5_2–C6_2	0.9(4)
C13–N3–C17–C18	94.3(2)	C18–C4_2–C5_2–C6_2	−177.4(2)
C14–N3–C17–C18	−133.8(2)	F1_2–C1_2–C6_2–C5_2	−178.1(2)
C20–N4–C17–N3	134.3(2)	C2_2–C1_2–C6_2–C5_2	0.2(4)
C11–N4–C17–N3	−40.3(3)	C4_2–C5_2–C6_2–C1_2	−0.7(4)

11-(*o*-Tolyl)hexahydro-1H,5H,9H-dipyrrolo[1,2-a:2',1'-b]pyrimidin-9-one (3ea)

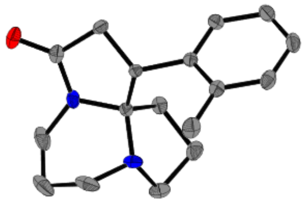

Figure 7: Structure of compound 3ea in the crystal. All atoms were drawn with anisotropic thermal ellipsoids at 50% probability level. H-atoms were omitted for clarity.

Crystallized by slowly removing the solvent. A colorless, block-shaped crystal was mounted on a MiTeGen micromount with perfluoroether oil. Data for **3ea** were collected from a shock-cooled single crystal at 100(2) K on a Bruker APEX2 QUAZAR three-circle diffractometer with a microfocus sealed X-ray tube using a mirror optics as monochromator and a Bruker APEXII detector. The diffractometer was equipped with an Oxford Cryostream 800 low temperature device and used MoKα radiation (λ = 0.71073 Å). All data were integrated with SAINT V8.40B and a multi-scan absorption correction using SADABS 2016/2 was applied.[28,29] The structure was solved by direct methods with SHELXT 2018/2 and refined by full-matrix least-squares methods against F2 using SHELXL-2018/3.[30,31] All non-hydrogen atoms were refined with anisotropic displacement parameters. All hydrogen atoms were refined isotropic on calculated positions using a riding model with their Uiso values constrained to 1.5 times the Ueq of their pivot atoms for terminal sp3 carbon atoms and 1.2 times for all other carbon atoms. Crystallographic data for the structures reported in this paper have been deposited with the Cambridge Crystallographic Data Centre.[32] CCDC 2457282 contain the supplementary crystallographic data for this paper. These data can be obtained free of charge from The Cambridge Crystallographic Data Centre via www.ccdc.cam.ac.uk/structures. This report and the CIF file were generated using FinalCif.[33]

Table 10: Crystal data and structure refinement for 3ea.

CCDC number	2457282
Empirical formula	$C_{17}H_{22}N_2O$
Formula weight	270.36
Temperature [K]	100(2)
Crystal system	orthorhombic
Space group (number)	$P2_12_12_1$ (19)
a [Å]	6.951(4)
b [Å]	13.847(7)
c [Å]	15.400(9)
α [°]	90
β [°]	90
γ [°]	90
Volume [Å^3]	1482.3(14)
Z	4
ρ_{calc} [gcm^{-3}]	1.211
μ [mm^{-1}]	0.076
F(000)	584
Crystal size [mm^3]	0.160×0.355×0.607
Crystal color	colorless
Crystal shape	block
Radiation	MoK_α (λ=0.71073 Å)
2θ range [°]	3.96 to 60.35 (0.71 Å)
Index ranges	−9 ≤ h ≤ 9<br>−19 ≤ k ≤ 19<br>−21 ≤ l ≤ 21
Reflections collected	44206
Independent reflections	4199<br>R_{int} = 0.0336<br>R_{sigma} = 0.0171
Completeness to θ = 25.242°	99.7 %
Data / Restraints / Parameters	4199 / 122 / 182
Absorption correction T_{min}/T_{max} (method)	0.7153 / 0.7460 (multi-scan)
Goodness-of-fit on F^2	1.081
Final *R* indexes [I≥2σ(I)]	R_1 = 0.0360<br>wR_2 = 0.0957
Final *R* indexes [all data]	R_1 = 0.0386<br>wR_2 = 0.0977
Largest peak/hole [eÅ^{-3}]	0.30/−0.16
Flack X parameter	0.2(3)

Table 11: Atomic coordinates and Ueq [Å2] for 3ea.

Atom	*x*	*y*	*z*	U_{eq}
O1	0.65487(17)	0.24132(9)	0.57033(7)	0.0256(3)
N1	0.6626(2)	0.37336(9)	0.48014(8)	0.0195(3)
N2	0.5371(2)	0.47955(9)	0.36895(9)	0.0211(3)
C1	0.6447(3)	0.45026(14)	0.54367(11)	0.0320(4)
H1A	0.759012	0.492765	0.540696	0.038
H1B	0.638263	0.422261	0.602753	0.038
C2	0.4620(3)	0.50973(14)	0.52587(14)	0.0375(5)
H2A	0.361795	0.492350	0.568794	0.045
H2B	0.491285	0.579271	0.532810	0.045
C3	0.3853(3)	0.49138(13)	0.43377(14)	0.0310(4)
H3A	0.304603	0.432466	0.434431	0.037
H3B	0.302234	0.546200	0.416513	0.037
C4	0.6506(3)	0.56469(11)	0.34646(12)	0.0283(4)
H4A	0.692706	0.600073	0.398996	0.034
H4B	0.577783	0.609041	0.308225	0.034
C5	0.8210(3)	0.51950(12)	0.29890(11)	0.0261(3)
H5A	0.931555	0.564436	0.295955	0.031
H5B	0.785343	0.499104	0.239425	0.031
C6	0.8646(2)	0.43271(11)	0.35699(10)	0.0190(3)
H6A	0.945071	0.451936	0.407145	0.023
H6B	0.932126	0.381510	0.324043	0.023
C7	0.6650(2)	0.39791(10)	0.38717(9)	0.0144(3)
C8	0.5887(2)	0.30169(10)	0.34754(9)	0.0133(2)
H8	0.445628	0.302791	0.354094	0.016
C9	0.6641(2)	0.22505(10)	0.41098(9)	0.0177(3)
H9A	0.579497	0.167550	0.411917	0.021
H9B	0.796506	0.204824	0.395785	0.021
C10	0.6596(2)	0.27800(11)	0.49747(9)	0.0185(3)
C1_1	0.2992(2)	0.36172(14)	0.21680(11)	0.0271(4)
H1A_1	0.235993	0.315806	0.255927	0.041
H1B_1	0.219387	0.371224	0.165043	0.041
H1C_1	0.316401	0.423609	0.246668	0.041
C2_1	0.4931(2)	0.32244(11)	0.19021(10)	0.0184(3)
C3_1	0.5389(3)	0.31615(12)	0.10180(10)	0.0231(3)
H3_1	0.449784	0.339841	0.060159	0.028
C4_1	0.7104(3)	0.27635(12)	0.07351(10)	0.0265(3)
H4_1	0.738210	0.273108	0.013187	0.032
C5_1	0.8413(3)	0.24127(12)	0.13352(10)	0.0249(3)
H5_1	0.958144	0.212670	0.114543	0.030
C6_1	0.8004(2)	0.24818(11)	0.22206(10)	0.0196(3)
H6_1	0.890854	0.224289	0.263027	0.024
C7_1	0.6294(2)	0.28950(10)	0.25157(9)	0.0150(3)

U_{eq} is defined as 1/3 of the trace of the orthogonalized U_{ij} tensor.

Table 12: Anisotropic displacement parameters [Å2] for 3ea. The anisotropic displacement factor exponent takes the form: −2π2[h2(a*)2U11 + k2(b*)2U22 + … + 2hka*b*U12].

Atom	U_{11}	U_{22}	U_{33}	U_{23}	U_{13}	U_{12}
O1	0.0220(5)	0.0366(6)	0.0182(5)	0.0095(5)	−0.0020(4)	−0.0019(5)
N1	0.0245(6)	0.0220(6)	0.0121(5)	−0.0017(4)	0.0007(5)	−0.0047(5)
N2	0.0209(6)	0.0131(5)	0.0293(7)	−0.0013(5)	0.0006(5)	0.0027(5)
C1	0.0442(11)	0.0327(9)	0.0190(7)	−0.0106(6)	0.0061(7)	−0.0140(8)
C2	0.0432(11)	0.0263(9)	0.0430(11)	−0.0176(8)	0.0231(9)	−0.0090(8)
C3	0.0236(8)	0.0192(7)	0.0503(11)	−0.0088(7)	0.0096(8)	0.0029(6)
C4	0.0357(9)	0.0141(6)	0.0350(9)	0.0011(6)	0.0003(8)	−0.0004(6)
C5	0.0350(9)	0.0198(7)	0.0235(7)	0.0029(6)	0.0032(7)	−0.0093(7)
C6	0.0171(7)	0.0204(7)	0.0195(7)	−0.0010(5)	0.0008(5)	−0.0048(5)
C7	0.0150(6)	0.0149(6)	0.0134(6)	−0.0008(5)	0.0000(5)	0.0000(5)
C8	0.0128(6)	0.0130(6)	0.0140(6)	0.0007(5)	−0.0006(5)	0.0008(5)
C9	0.0185(6)	0.0159(6)	0.0187(6)	0.0038(5)	−0.0007(5)	0.0021(5)
C10	0.0132(6)	0.0259(7)	0.0165(6)	0.0031(5)	−0.0013(5)	−0.0009(5)
C1_1	0.0186(8)	0.0392(9)	0.0235(7)	0.0039(7)	−0.0060(6)	0.0036(7)
C2_1	0.0197(7)	0.0178(6)	0.0175(6)	0.0005(5)	−0.0022(5)	−0.0040(5)
C3_1	0.0313(8)	0.0220(7)	0.0161(7)	0.0008(5)	−0.0050(6)	−0.0067(6)
C4_1	0.0409(9)	0.0230(7)	0.0156(6)	−0.0026(6)	0.0055(6)	−0.0069(7)
C5_1	0.0307(8)	0.0204(7)	0.0238(7)	−0.0035(6)	0.0091(6)	−0.0003(6)
C6_1	0.0220(7)	0.0174(6)	0.0195(7)	−0.0014(5)	0.0024(5)	0.0011(6)
C7_1	0.0172(6)	0.0126(6)	0.0151(6)	−0.0010(5)	−0.0001(5)	−0.0026(5)

Table 13: Bond lengths and angles for 3ea.

Atom–Atom	Length [Å]
O1–C10	1.2321(19)
N1–C10	1.347(2)
N1–C1	1.451(2)
N1–C7	1.4716(19)
N2–C4	1.460(2)
N2–C3	1.462(2)
N2–C7	1.466(2)
C1–C2	1.538(3)
C1–H1A	0.9900
C1–H1B	0.9900
C2–C3	1.536(3)
C2–H2A	0.9900
C2–H2B	0.9900
C3–H3A	0.9900
C3–H3B	0.9900
C4–C5	1.527(3)
C4–H4A	0.9900
C4–H4B	0.9900
C5–C6	1.529(2)
C5–H5A	0.9900
C5–H5B	0.9900
C6–C7	1.541(2)
C6–H6A	0.9900
C6–H6B	0.9900
C7–C8	1.5584(19)
C8–C7_1	1.514(2)
C8–C9	1.5347(19)
C8–H8	1.0000
C9–C10	1.521(2)
C9–H9A	0.9900
C9–H9B	0.9900
C1_1–C2_1	1.510(2)
C1_1–H1A_1	0.9800
C1_1–H1B_1	0.9800
C1_1–H1C_1	0.9800
C2_1–C3_1	1.401(2)
C2_1–C7_1	1.413(2)
C3_1–C4_1	1.384(3)
C3_1–H3_1	0.9500
C4_1–C5_1	1.385(3)
C4_1–H4_1	0.9500
C5_1–C6_1	1.396(2)
C5_1–H5_1	0.9500
C6_1–C7_1	1.396(2)
C6_1–H6_1	0.9500

Atom–Atom–Atom	Angle [°]
C10–N1–C1	125.74(14)
C10–N1–C7	114.79(12)
C1–N1–C7	119.17(13)
C4–N2–C3	117.48(14)
C4–N2–C7	109.89(14)
C3–N2–C7	113.19(13)

N1–C1–C2	110.09(16)
N1–C1–H1A	109.6
C2–C1–H1A	109.6
N1–C1–H1B	109.6
C2–C1–H1B	109.6
H1A–C1–H1B	108.2
C3–C2–C1	111.26(14)
C3–C2–H2A	109.4
C1–C2–H2A	109.4
C3–C2–H2B	109.4
C1–C2–H2B	109.4
H2A–C2–H2B	108.0
N2–C3–C2	113.47(16)
N2–C3–H3A	108.9
C2–C3–H3A	108.9
N2–C3–H3B	108.9
C2–C3–H3B	108.9
H3A–C3–H3B	107.7
N2–C4–C5	101.66(13)
N2–C4–H4A	111.4
C5–C4–H4A	111.4
N2–C4–H4B	111.4
C5–C4–H4B	111.4
H4A–C4–H4B	109.3
C4–C5–C6	101.28(13)
C4–C5–H5A	111.5
C6–C5–H5A	111.5
C4–C5–H5B	111.5
C6–C5–H5B	111.5
H5A–C5–H5B	109.3
C5–C6–C7	104.10(13)
C5–C6–H6A	110.9
C7–C6–H6A	110.9
C5–C6–H6B	110.9
C7–C6–H6B	110.9
H6A–C6–H6B	109.0
N2–C7–N1	110.95(12)
N2–C7–C6	104.33(12)
N1–C7–C6	112.08(12)
N2–C7–C8	112.21(12)
N1–C7–C8	100.35(11)
C6–C7–C8	117.11(12)
C7_1–C8–C9	118.72(12)
C7_1–C8–C7	114.46(11)
C9–C8–C7	103.04(11)
C7_1–C8–H8	106.6
C9–C8–H8	106.6
C7–C8–H8	106.6
C10–C9–C8	102.54(12)
C10–C9–H9A	111.3
C8–C9–H9A	111.3
C10–C9–H9B	111.3
C8–C9–H9B	111.3
H9A–C9–H9B	109.2
O1–C10–N1	125.79(15)
O1–C10–C9	126.83(15)
N1–C10–C9	107.38(12)
C2_1–C1_1–H1A_1	109.5
C2_1–C1_1–H1B_1	109.5
H1A_1–C1_1–H1B_1	109.5
C2_1–C1_1–H1C_1	109.5
H1A_1–C1_1–H1C_1	109.5
H1B_1–C1_1–H1C_1	109.5
C3_1–C2_1–C7_1	118.53(15)
C3_1–C2_1–C1_1	119.24(14)
C7_1–C2_1–C1_1	122.22(14)
C4_1–C3_1–C2_1	121.76(15)
C4_1–C3_1–H3_1	119.1
C2_1–C3_1–H3_1	119.1
C3_1–C4_1–C5_1	119.71(15)
C3_1–C4_1–H4_1	120.1
C5_1–C4_1–H4_1	120.1
C4_1–C5_1–C6_1	119.62(16)
C4_1–C5_1–H5_1	120.2
C6_1–C5_1–H5_1	120.2
C7_1–C6_1–C5_1	121.28(15)
C7_1–C6_1–H6_1	119.4
C5_1–C6_1–H6_1	119.4
C6_1–C7_1–C2_1	119.05(14)
C6_1–C7_1–C8	121.50(13)
C2_1–C7_1–C8	119.44(13)

Table 14: Torsion angles for 3ea.

Atom–Atom–Atom–Atom	Torsion Angle [°]
C10–N1–C1–C2	−117.03(19)
C7–N1–C1–C2	56.3(2)
N1–C1–C2–C3	−16.7(2)
C4–N2–C3–C2	−69.4(2)
C7–N2–C3–C2	60.38(19)
C1–C2–C3–N2	−37.4(2)
C3–N2–C4–C5	165.28(15)
C7–N2–C4–C5	33.96(17)
N2–C4–C5–C6	−42.72(16)
C4–C5–C6–C7	36.67(15)
C4–N2–C7–N1	110.09(15)
C3–N2–C7–N1	−23.46(18)
C4–N2–C7–C6	−10.80(16)
C3–N2–C7–C6	−144.34(13)
C4–N2–C7–C8	−138.55(13)
C3–N2–C7–C8	87.90(16)
C10–N1–C7–N2	138.45(14)
C1–N1–C7–N2	−35.6(2)
C10–N1–C7–C6	−105.36(15)
C1–N1–C7–C6	80.61(18)
C10–N1–C7–C8	19.68(17)
C1–N1–C7–C8	−154.36(15)
C5–C6–C7–N2	−16.85(15)
C5–C6–C7–N1	−136.98(13)
C5–C6–C7–C8	107.83(14)
N2–C7–C8–C7_1	80.15(15)
N1–C7–C8–C7_1	−162.00(12)
C6–C7–C8–C7_1	−40.47(17)
N2–C7–C8–C9	−149.51(12)
N1–C7–C8–C9	−31.66(13)
C6–C7–C8–C9	89.87(14)
C7_1–C8–C9–C10	160.76(12)
C7–C8–C9–C10	33.05(14)
C1–N1–C10–O1	−5.5(3)
C7–N1–C10–O1	−179.07(14)
C1–N1–C10–C9	174.80(16)
C7–N1–C10–C9	1.22(18)
C8–C9–C10–O1	158.23(15)
C8–C9–C10–N1	−22.07(16)
C7_1–C2_1–C3_1–C4_1	1.8(2)
C1_1–C2_1–C3_1–C4_1	−176.74(15)
C2_1–C3_1–C4_1–C5_1	0.2(2)
C3_1–C4_1–C5_1–C6_1	−1.3(2)
C4_1–C5_1–C6_1–C7_1	0.3(2)
C5_1–C6_1–C7_1–C2_1	1.8(2)
C5_1–C6_1–C7_1–C8	−176.91(14)
C3_1–C2_1–C7_1–C6_1	−2.7(2)
C1_1–C2_1–C7_1–C6_1	175.76(14)
C3_1–C2_1–C7_1–C8	175.95(13)
C1_1–C2_1–C7_1–C8	−5.5(2)
C9–C8–C7_1–C6_1	−34.23(19)
C7–C8–C7_1–C6_1	87.91(16)
C9–C8–C7_1–C2_1	147.11(13)
C7–C8–C7_1–C2_1	−90.75(16)

1-Methyl-8-phenyl-8a-((E)-2-(thienyl)vinyl)-hexahydropyrrolo[1,2-a]pyrimidin-6(2H)-one (3aj)

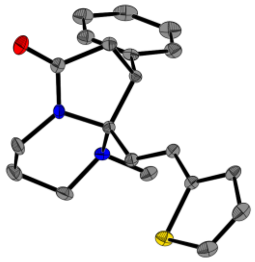

Figure 8: Structure of compound 3aj in the crystal. All atoms were drawn with anisotropic thermal ellipsoids at 50% probability level. H-atoms were omitted for clarity.

Crystallized by slowly removing the solvent. A colorless, block-shaped crystal was mounted on a MiTeGen micromount with perfluoroether oil. Data for **3aj** were collected from a shock-cooled single crystal at 100(2) K on a Bruker APEX2 QUAZAR three-circle diffractometer with a microfocus sealed X-ray tube using a mirror optics as monochromator and a Bruker APEXII detector. The diffractometer was equipped with an Oxford Cryostream 800 low temperature device and used MoK_α radiation (λ = 0.71073 Å). All data were integrated with SAINT V8.40B and a multi-scan absorption correction using SADABS 2016/2 was applied.[28,29] The structure was solved by direct methods with SHELXT 2018/2 and refined by full-matrix least-squares methods against F^2 using SHELXL-2018/3.[30,31] All non-hydrogen atoms were refined with anisotropic displacement parameters. All hydrogen atoms were refined isotropic on calculated positions using a riding model with their U_{iso} values constrained to 1.5 times the U_{eq} of their pivot atoms for terminal sp^3 carbon atoms and 1.2 times for all other carbon atoms. Crystallographic data for the structures reported in this paper have been deposited with the Cambridge Crystallographic Data Centre.[32] CCDC 2457283 contain the supplementary crystallographic data for this paper. These data can be obtained free of charge from The Cambridge Crystallographic Data Centre via www.ccdc.cam.ac.uk/structures. This report and the CIF file were generated using FinalCif.[33]

Table 15: Crystal data and structure refinement for 3aj.

CCDC number	2457283
Empirical formula	$C_{20}H_{22}N_2OS$
Formula weight	338.45
Temperature [K]	100(2)
Crystal system	orthorhombic
Space group (number)	$Pca2_1$ (29)
a [Å]	12.823(7)
b [Å]	7.519(3)
c [Å]	17.772(8)
α [°]	90
β [°]	90
γ [°]	90
Volume [Å^3]	1713.5(14)
Z	4
ρ_{calc} [gcm^{-3}]	1.312
μ [mm^{-1}]	0.198
F(000)	720
Crystal size [mm^3]	0.136×0.208×0.317
Crystal color	colorless
Crystal shape	block
Radiation	MoK_α (λ=0.71073 Å)
2θ range [°]	4.58 to 61.14 (0.70 Å)
Index ranges	−17 ≤ h ≤ 18<br>−10 ≤ k ≤ 10<br>−25 ≤ l ≤ 25
Reflections collected	50423
Independent reflections	5241<br>R_{int} = 0.0700<br>R_{sigma} = 0.0321
Completeness to θ = 25.242°	100.0 %
Data / Restraints / Parameters	5241 / 170 / 218
Absorption correction T_{min}/T_{max} (method)	0.6947 / 0.7461 (multi-scan)
Goodness-of-fit on F^2	1.083
Final R indexes [I≥2σ(I)]	R_1 = 0.0351<br>wR_2 = 0.0902
Final R indexes [all data]	R_1 = 0.0386<br>wR_2 = 0.0924
Largest peak/hole [eÅ^{-3}]	0.37/−0.23
Flack X parameter	0.01(2)

Table 16: Atomic coordinates and Ueq [Å2] for 3aj.

Atom	*x*	*y*	*z*	U_{eq}
O1	0.64296(13)	1.1496(2)	0.42350(10)	0.0229(3)
N1	0.60711(13)	0.8725(2)	0.47059(10)	0.0143(3)
N2	0.55829(14)	0.6638(2)	0.56492(10)	0.0145(3)
C1	0.70621(16)	0.7879(3)	0.45365(13)	0.0204(4)
H1A	0.759278	0.879488	0.441942	0.025
H1B	0.698663	0.709435	0.409260	0.025
C2	0.74031(17)	0.6800(3)	0.52123(14)	0.0232(5)
H2A	0.760069	0.760953	0.562791	0.028
H2B	0.802077	0.607571	0.507916	0.028
C3	0.65219(17)	0.5590(3)	0.54665(13)	0.0196(4)
H3A	0.635672	0.473015	0.506189	0.024
H3B	0.674487	0.491041	0.591569	0.024
C4	0.51868(15)	0.7686(3)	0.50108(11)	0.0122(3)
C5	0.44274(15)	0.9203(3)	0.52626(11)	0.0122(3)
H5	0.369017	0.880972	0.518541	0.015
C6	0.46900(16)	1.0701(3)	0.47010(12)	0.0150(4)
H6A	0.425310	1.060821	0.424337	0.018
H6B	0.458130	1.188404	0.493226	0.018
C7	0.58282(16)	1.0409(3)	0.45154(12)	0.0151(4)
C8	0.47858(18)	0.5481(3)	0.59697(13)	0.0191(4)
H8A	0.414703	0.616718	0.605609	0.029
H8B	0.503528	0.499449	0.644848	0.029
H8C	0.463943	0.450514	0.561998	0.029
C9	0.47543(15)	0.6528(3)	0.43804(11)	0.0138(4)
H9	0.521590	0.566108	0.418003	0.017
C10	0.38005(15)	0.6589(3)	0.40757(11)	0.0137(3)
H10	0.331691	0.741521	0.428025	0.016
C1_1	0.45751(16)	0.9792(3)	0.60752(11)	0.0143(4)
C2_1	0.37844(19)	0.9464(3)	0.65964(12)	0.0198(4)
H2_1	0.316859	0.886537	0.644074	0.024
C3_1	0.3888(2)	1.0006(3)	0.73448(14)	0.0257(5)
H3_1	0.334922	0.975413	0.769598	0.031
C4_1	0.4772(2)	1.0908(3)	0.75760(13)	0.0259(5)
H4_1	0.484205	1.128076	0.808427	0.031
C5_1	0.5562(2)	1.1265(3)	0.70552(13)	0.0233(5)
H5_1	0.616851	1.189315	0.720841	0.028
C6_1	0.54608(18)	1.0705(3)	0.63140(12)	0.0187(4)
H6_1	0.600351	1.094701	0.596489	0.022
S1_2	0.42171(4)	0.38416(7)	0.30512(3)	0.01952(12)
C1_2	0.34504(16)	0.5479(3)	0.34525(11)	0.0136(4)
C2_2	0.24753(16)	0.5582(3)	0.30991(12)	0.0159(4)
H2_2	0.193994	0.639375	0.323544	0.019
C3_2	0.23920(19)	0.4303(3)	0.25059(12)	0.0211(4)
H3_2	0.178766	0.417656	0.220141	0.025
C4_2	0.3261(2)	0.3287(3)	0.24198(13)	0.0227(4)
H4_2	0.332930	0.237657	0.205262	0.027

U_{eq} is defined as 1/3 of the trace of the orthogonalized U_{ij} tensor.

Table 17: Anisotropic displacement parameters [Å2] for 3aj. The anisotropic displacement factor exponent takes the form: −2π2[h2(a*)2U11 + k2(b*)2U22 + … + 2hka*b*U12].

Atom	U_{11}	U_{22}	U_{33}	U_{23}	U_{13}	U_{12}
O1	0.0228(8)	0.0246(8)	0.0214(8)	0.0027(7)	0.0035(6)	−0.0080(6)
N1	0.0106(7)	0.0170(8)	0.0154(8)	−0.0029(7)	0.0012(6)	−0.0018(6)
N2	0.0177(8)	0.0132(8)	0.0126(8)	−0.0009(6)	−0.0028(6)	0.0014(6)
C1	0.0115(9)	0.0271(11)	0.0227(10)	−0.0072(8)	0.0023(7)	0.0023(8)
C2	0.0150(9)	0.0266(11)	0.0279(11)	−0.0070(9)	−0.0042(8)	0.0055(8)
C3	0.0197(10)	0.0170(9)	0.0221(10)	−0.0048(8)	−0.0061(8)	0.0063(8)
C4	0.0122(8)	0.0121(8)	0.0124(8)	−0.0014(7)	0.0002(6)	−0.0006(6)
C5	0.0118(8)	0.0124(8)	0.0125(8)	−0.0014(7)	0.0001(7)	0.0009(6)
C6	0.0167(8)	0.0144(9)	0.0140(9)	0.0004(7)	−0.0002(7)	0.0005(7)
C7	0.0167(9)	0.0173(10)	0.0114(8)	−0.0017(7)	0.0001(7)	−0.0019(7)
C8	0.0248(10)	0.0142(9)	0.0184(10)	0.0017(7)	−0.0017(8)	−0.0023(8)
C9	0.0145(9)	0.0144(9)	0.0125(8)	−0.0028(7)	−0.0005(7)	−0.0007(7)
C10	0.0153(8)	0.0140(8)	0.0120(8)	−0.0006(7)	0.0008(7)	−0.0015(7)
C1_1	0.0183(9)	0.0125(8)	0.0121(8)	−0.0010(7)	−0.0001(7)	0.0032(7)
C2_1	0.0242(10)	0.0191(10)	0.0160(10)	−0.0031(8)	0.0040(8)	0.0004(9)
C3_1	0.0384(13)	0.0247(11)	0.0139(9)	−0.0017(8)	0.0064(9)	0.0031(10)
C4_1	0.0438(15)	0.0215(11)	0.0124(9)	−0.0037(8)	−0.0055(9)	0.0065(10)
C5_1	0.0293(11)	0.0216(11)	0.0190(10)	−0.0058(9)	−0.0087(9)	0.0025(9)
C6_1	0.0225(10)	0.0175(9)	0.0161(10)	−0.0035(8)	−0.0024(8)	0.0004(8)
S1_2	0.0237(2)	0.0196(2)	0.0153(2)	−0.0043(2)	−0.0022(2)	0.00263(19)
C1_2	0.0169(9)	0.0136(9)	0.0103(8)	−0.0007(6)	0.0002(7)	−0.0017(7)
C2_2	0.0215(9)	0.0146(8)	0.0117(8)	−0.0016(8)	0.0010(8)	−0.0044(7)
C3_2	0.0266(11)	0.0227(10)	0.0140(9)	−0.0004(8)	−0.0064(8)	−0.0053(9)
C4_2	0.0331(12)	0.0203(10)	0.0146(10)	−0.0056(8)	−0.0045(8)	−0.0016(9)

Table 18: Bond lengths and angles for 3aj.

Atom–Atom	Length [Å]
O1–C7	1.230(3)
N1–C7	1.347(3)
N1–C1	1.452(3)
N1–C4	1.480(3)
N2–C8	1.458(3)
N2–C4	1.472(3)
N2–C3	1.475(3)
C1–C2	1.514(3)
C1–H1A	0.9900
C1–H1B	0.9900
C2–C3	1.520(3)
C2–H2A	0.9900
C2–H2B	0.9900
C3–H3A	0.9900
C3–H3B	0.9900
C4–C9	1.523(3)
C4–C5	1.565(3)
C5–C1_1	1.522(3)
C5–C6	1.542(3)
C5–H5	1.0000
C6–C7	1.512(3)
C6–H6A	0.9900
C6–H6B	0.9900
C8–H8A	0.9800
C8–H8B	0.9800
C8–H8C	0.9800
C9–C10	1.338(3)
C9–H9	0.9500
C10–C1_2	1.458(3)
C10–H10	0.9500
C1_1–C6_1	1.393(3)
C1_1–C2_1	1.395(3)
C2_1–C3_1	1.397(3)
C2_1–H2_1	0.9500
C3_1–C4_1	1.384(4)
C3_1–H3_1	0.9500
C4_1–C5_1	1.398(4)
C4_1–H4_1	0.9500
C5_1–C6_1	1.389(3)
C5_1–H5_1	0.9500
C6_1–H6_1	0.9500
S1_2–C4_2	1.714(2)
S1_2–C1_2	1.730(2)
C1_2–C2_2	1.401(3)
C2_2–C3_2	1.431(3)
C2_2–H2_2	0.9500

C3_2–C4_2	1.359(4)
C3_2–H3_2	0.9500
C4_2–H4_2	0.9500

Atom–Atom–Atom	Angle [°]
C7–N1–C1	124.15(19)
C7–N1–C4	114.26(17)
C1–N1–C4	121.03(17)
C8–N2–C4	112.25(17)
C8–N2–C3	109.85(18)
C4–N2–C3	113.46(16)
N1–C1–C2	108.83(18)
N1–C1–H1A	109.9
C2–C1–H1A	109.9
N1–C1–H1B	109.9
C2–C1–H1B	109.9
H1A–C1–H1B	108.3
C1–C2–C3	110.00(18)
C1–C2–H2A	109.7
C3–C2–H2A	109.7
C1–C2–H2B	109.7
C3–C2–H2B	109.7
H2A–C2–H2B	108.2
N2–C3–C2	110.64(18)
N2–C3–H3A	109.5
C2–C3–H3A	109.5
N2–C3–H3B	109.5
C2–C3–H3B	109.5
H3A–C3–H3B	108.1
N2–C4–N1	107.48(16)
N2–C4–C9	112.73(16)
N1–C4–C9	108.14(16)
N2–C4–C5	112.62(16)
N1–C4–C5	101.36(15)
C9–C4–C5	113.59(16)
C1_1–C5–C6	112.00(17)
C1_1–C5–C4	113.94(16)
C6–C5–C4	102.20(15)
C1_1–C5–H5	109.5
C6–C5–H5	109.5
C4–C5–H5	109.5
C7–C6–C5	104.23(16)
C7–C6–H6A	110.9
C5–C6–H6A	110.9
C7–C6–H6B	110.9
C5–C6–H6B	110.9
H6A–C6–H6B	108.9
O1–C7–N1	125.6(2)
O1–C7–C6	126.7(2)
N1–C7–C6	107.74(17)
N2–C8–H8A	109.5
N2–C8–H8B	109.5
H8A–C8–H8B	109.5
N2–C8–H8C	109.5
H8A–C8–H8C	109.5
H8B–C8–H8C	109.5
C10–C9–C4	127.64(19)
C10–C9–H9	116.2
C4–C9–H9	116.2
C9–C10–C1_2	124.71(19)
C9–C10–H10	117.6
C1_2–C10–H10	117.6
C6_1–C1_1–C2_1	118.5(2)
C6_1–C1_1–C5	122.25(18)
C2_1–C1_1–C5	119.21(19)
C1_1–C2_1–C3_1	120.8(2)
C1_1–C2_1–H2_1	119.6
C3_1–C2_1–H2_1	119.6
C4_1–C3_1–C2_1	120.2(2)
C4_1–C3_1–H3_1	119.9
C2_1–C3_1–H3_1	119.9
C3_1–C4_1–C5_1	119.4(2)
C3_1–C4_1–H4_1	120.3
C5_1–C4_1–H4_1	120.3
C6_1–C5_1–C4_1	120.1(2)
C6_1–C5_1–H5_1	119.9
C4_1–C5_1–H5_1	119.9
C5_1–C6_1–C1_1	120.9(2)
C5_1–C6_1–H6_1	119.5
C1_1–C6_1–H6_1	119.5
C4_2–S1_2–C1_2	92.08(11)
C2_2–C1_2–C10	125.72(19)
C2_2–C1_2–S1_2	111.20(15)
C10–C1_2–S1_2	123.08(15)
C1_2–C2_2–C3_2	111.09(19)
C1_2–C2_2–H2_2	124.5
C3_2–C2_2–H2_2	124.5
C4_2–C3_2–C2_2	113.5(2)
C4_2–C3_2–H3_2	123.2
C2_2–C3_2–H3_2	123.2
C3_2–C4_2–S1_2	112.09(17)
C3_2–C4_2–H4_2	124.0
S1_2–C4_2–H4_2	124.0

Table 19: Torsion angles for 3aj.

Atom–Atom–Atom–Atom	Torsion Angle [°]
C7–N1–C1–C2	138.7(2)
C4–N1–C1–C2	−50.3(3)
N1–C1–C2–C3	50.9(2)
C8–N2–C3–C2	−173.79(18)
C4–N2–C3–C2	59.7(2)
C1–C2–C3–N2	−57.8(2)
C8–N2–C4–N1	−176.84(16)
C3–N2–C4–N1	−51.6(2)
C8–N2–C4–C9	−57.8(2)
C3–N2–C4–C9	67.5(2)
C8–N2–C4–C5	72.4(2)
C3–N2–C4–C5	−162.37(16)
C7–N1–C4–N2	−138.71(18)
C1–N1–C4–N2	49.5(2)
C7–N1–C4–C9	99.3(2)
C1–N1–C4–C9	−72.5(2)
C7–N1–C4–C5	−20.4(2)
C1–N1–C4–C5	167.79(17)
N2–C4–C5–C1_1	23.4(2)
N1–C4–C5–C1_1	−91.20(19)
C9–C4–C5–C1_1	153.07(17)
N2–C4–C5–C6	144.38(16)
N1–C4–C5–C6	29.81(18)
C9–C4–C5–C6	−85.92(19)
C1_1–C5–C6–C7	92.35(19)
C4–C5–C6–C7	−29.99(19)
C1–N1–C7–O1	−6.6(3)
C4–N1–C7–O1	−178.1(2)
C1–N1–C7–C6	172.84(18)
C4–N1–C7–C6	1.3(2)
C5–C6–C7–O1	−161.7(2)
C5–C6–C7–N1	18.9(2)
N2–C4–C9–C10	124.1(2)
N1–C4–C9–C10	−117.2(2)
C5–C4–C9–C10	−5.5(3)
C4–C9–C10–C1_2	177.63(19)
C6–C5–C1_1–C6_1	−46.4(3)
C4–C5–C1_1–C6_1	69.0(2)
C6–C5–C1_1–C2_1	131.3(2)
C4–C5–C1_1–C2_1	−113.3(2)
C6_1–C1_1–C2_1–C3_1	−1.3(3)
C5–C1_1–C2_1–C3_1	−179.2(2)
C1_1–C2_1–C3_1–C4_1	1.2(4)
C2_1–C3_1–C4_1–C5_1	−0.2(4)
C3_1–C4_1–C5_1–C6_1	−0.6(4)
C4_1–C5_1–C6_1–C1_1	0.4(4)
C2_1–C1_1–C6_1–C5_1	0.5(3)
C5–C1_1–C6_1–C5_1	178.3(2)
C9–C10–C1_2–C2_2	−176.5(2)
C9–C10–C1_2–S1_2	3.3(3)
C4_2–S1_2–C1_2–C2_2	−0.04(17)
C4_2–S1_2–C1_2–C10	−179.82(18)
C10–C1_2–C2_2–C3_2	179.71(19)
S1_2–C1_2–C2_2–C3_2	−0.1(2)
C1_2–C2_2–C3_2–C4_2	0.2(3)
C2_2–C3_2–C4_2–S1_2	−0.2(3)
C1_2–S1_2–C4_2–C3_2	0.1(2)

9. References

[1] C. M. Poteat, J. Yujin, M. Jung, J. D. Johnson, R. G. Williams, V. N. G.Lindsay, *Angew. Chem. Int. Ed.* **2020**, *59*, 18655–18661.

[2] M. Kotozaki, S. Chanthamath, I. Fujisawa, K. Shibatomi, S. Iwasa, *Chem. Commun.* **2017**, *53*, 12193–12196.

[3] M. A. Khanfar, L. Quinti, H. Wang, S. H. Choi, A. G. Kazantsev, R. B. Silverman, *Eur. J. Med. Chem.* **2014**, *76*, 414–426.

[4] W. C. Gao, J. J. Zhao, F. Hu, H. H. Chang, X. Li, W. L. Wei, *RSC Adv.* **2015**, *5*, 25222–25228.

[5] D. Dar'In, G. Kantin, O. Bakulina, M. Krasavin, *Synthesis* **2020**, *52*, 2259–2266.

[6] M. Kotozaki, S. Chanthamath, I. Fujisawa, K. Shibatomi, S. Iwasa, *Chem. Commun.* **2017**, *53*, 12193–12196.

[7] V. B. Birman, X. Li, Z. Han, *Org. Lett.* **2007**, *9*, 37–40.

[8] K. Murai, H. Komatsu, R. Nagao, H. Fujioka, *Org. Lett.* **2012**, *14*, 772–775.

[9] N. Aoyagi, Y. Furusho, Y. Sei, T. Endo, *Tetrahedron* **2013**, *69*, 5476–5480.

[10] D. Kalaitzakis, M. Triantafyllakis, M. Sofiadis, D. Noutsias, G. Vassilikogiannakis, *Angew. Chem. Int. Ed.* **2016**, *128*, 4681–4685.

[11] B. S. Pedersen, S. Scheibye, N. H. Nilsson, S.-O. Lawesson, *Bull. Soc. Chim. Belg.* **1978**, *87*, 223–228.

[12] T. Wakikawa, D. Sekine, Y. Murata, Y. Bunno, M. Kojima, Y. Nagashima, K. Tanaka, T. Yoshino, S. Matsunaga, *Angew. Chem. Int. Ed.* **2022**, *61*, e202213659.

[13] F. Neese, *WIREs Comput. Mol. Sci.* **2022**, *12*, e1606.

[14] F. Neese, *WIREs Comput. Mol. Sci.* **2012**, *2*, 73–78.

[15] F. Neese, F. Wennmohs, U. Becker, C. Riplinger, *J. Chem. Phys.* **2020**, *152*, 224108.

[16] C. Bannwarth, S. Ehlert, S. Grimme, *J. Chem. Theory Comput.* **2019**, *15*, 1652–1671.

[17] C. Bannwarth, E. Caldeweyher, S. Ehlert, A. Hansen, P. Pracht, J. Seibert, S. Spicher, S. Grimme, *WIREs Comput. Mol. Sci.* **2021**, *11*, e1493.

[18] S. Grimme, A. Hansen, S. Ehlert, J. M. Mewes, *J. Chem. Phys.* **2021**, *154*, 064103.

[19] T. Gasevic, J. B. Stückrath, S. Grimme, M. Bursch, *J. Phys. Chem. A* **2022**, *126*, 3826–3838.

[20] S. Grimme, *Chem. Eur. J.* **2012**, *18*, 9955–9964.

[21] Y. Zhao, D. G. Truhlar, *J. Phys. Chem. A* **2005**, *109*, 5656–5667.

[22] A. Hellweg, D. Rappoport, *Phys. Chem. Chem. Phys.* **2015**, *17*, 1010–1017.

[23] E. Caldeweyher, C. Bannwarth, S. Grimme, *J. Chem. Phys.* **2017**, *147*, 034112.

[24] E. Caldeweyher, S. Ehlert, A. Hansen, H. Neugebauer, S. Spicher, C. Bannwarth, S. Grimme, *J. Chem. Phys.* **2019**, *150*, 154122.

[25] E. Caldeweyher, J. M. Mewes, S. Ehlert, S. Grimme, *Phys. Chem. Chem. Phys.* **2020**, *22*, 8499–8512.

[26] A. V. Marenich, C. J. Cramer, D. G. Truhlar, *J. Phys. Chem. B* **2009**, *113*, 6378–6396.

[27] ***NBO 7.0.*** E. D. Glendening, J, K. Badenhoop, A. E. Reed, J. E. Carpenter, J. A. Bohmann, C. M. Morales, P. Karafiloglou, C. R. Landis, and F. Weinhold, Theoretical Chemistry Institute, University of Wisconsin, Madison (2018).

[28] Bruker, *SAINT*, *V8.40B*, Bruker AXS Inc., Madison, Wisconsin, USA.

[29] L. Krause, R. Herbst-Irmer, G. M. Sheldrick, D. Stalke, *J. Appl. Cryst.* **2015**, *48*, 3–10.

[30] G. M. Sheldrick, *Acta Cryst.* **2015**, *A71*, 3–8.

[31] G. M. Sheldrick, *Acta Cryst.* **2015**, *C71*, 3–8.

[32] C. R. Groom, I. J. Bruno, M. P. Lightfoot, S. C. Ward, *Acta Cryst.* **2016**, *B72*, 171–179.

[33] D. Kratzert, *FinalCif*, *V150*, https://dkratzert.de/finalcif.html.
